# Global, regional and national burden of skin and subcutaneous diseases: a systematic analysis of the Global Burden of Disease Study 2021

**DOI:** 10.1093/inthealth/ihaf070

**Published:** 2025-06-28

**Authors:** Deng Li, Siqi Fan, Haochen Zhao, Jiayi Song, Wei Li, Xuewen Xu

**Affiliations:** Department of Plastic and Burns Surgery, West China Hospital, Sichuan University, Chengdu, China; Department of Plastic and Burns Surgery, West China Hospital, Sichuan University, Chengdu, China; Department of Urology, West China Hospital, Sichuan University, Chengdu, China; Department of Plastic and Burns Surgery, West China Hospital, Sichuan University, Chengdu, China; Department of Plastic and Burns Surgery, West China Hospital, Sichuan University, Chengdu, China; Department of Plastic and Burns Surgery, West China Hospital, Sichuan University, Chengdu, China

**Keywords:** age-period-cohort, disability-adjusted life years, global burden of disease, skin and subcutaneous diseases, sociodemographic index

## Abstract

**Background:**

Skin and subcutaneous diseases (SSDs) represent a growing global health burden. This study aims to assess global, regional and national trends in the incidence, prevalence, mortality and disability-adjusted life years (DALYs) associated with 15 specific SSDs from 1990 to 2021, providing a comprehensive stratification by age, sex, sociodemographic index (SDI) and region.

**Methods:**

Data from the Global Burden of Disease (GBD) Study 2021, covering 204 countries, were analysed for age-standardized rates of incidence, prevalence, mortality and DALYs. Temporal trends were assessed using annual percentage change, age-period-cohort modelling and compositional analysis by SDI and GBD region.

**Results:**

In 2021, SSDs accounted for 4.7 billion incident cases, 2.0 billion prevalent cases, 119 129 deaths and 41.9 million DALYs globally. Incidence and prevalence have increased by >35% since 1990, with a higher burden among females and older adults. Immune-mediated and inflammatory SSDs have overtaken infectious conditions in high- and middle-SDI regions, while infections still dominate in low-SDI regions. Sub-Saharan Africa and tropical Latin America had the highest incidence and mortality burdens, respectively. A marked epidemiological shift was observed across most regions, with notable compositional transitions in SSD types over time.

**Conclusions:**

SSDs are increasing globally, with significant regional and socio-economic disparities. Targeted interventions and improved access to dermatologic care are critical for addressing the growing burden, especially in resource-limited regions.

## Introduction

Skin and subcutaneous diseases (SSDs) represent a formidable health burden, afflicting tens of millions of individuals worldwide, transcending geographic boundaries, racial lines and socio-economic strata.^1–3^ Their increasing incidence, prevalence, morbidity and mortality rates have catapulted SSDs into the forefront of global public health concerns.^4^ The intricate interplay between the epidemiology and burden of SSDs encompasses demographic shifts, socio-economic evolution, racial variations and exposure to risk factors, notably ultraviolet (UV) radiation.^5,6^ Early intervention strategies, encompassing identification of high-risk individuals, the emergence of innovative UV radiation protection technologies and products and the availability of diverse treatment modalities, hold promise in mitigating the SSD burden.^7–12^

However, the past 3 decades have witnessed profound transformations in global demographics, socio-economic landscapes and SSD risk factors.^13^ A pivotal development is the global aging trend, fuelled by declining fertility rates and lengthening life expectancy.^14,15^ Additionally, stratospheric ozone depletion has intensified surface ultraviolet B radiation exposure, significantly impacting SSD incidence and burden.^16^ Hence the need for tailored global and regional policies aimed at SSD prevention is paramount.

While studies focusing on SSD incidence and mortality trends in the USA, Australia and select European nations have illuminated aspects of this health challenge,^17–22^ often leveraging population-specific surveys or registries, the research landscape remains limited for regions characterized by low SSD incidence or resource constraints. Accurate and comprehensive epidemiological insights into the global SSD burden remain elusive.

In this regard, the Global Burden of Disease (GBD) study serves as an essential resource, bridging gaps where genuine disease burden data are absent or scarce across numerous countries and regions. Utilizing data from GBD 2021, this research aims to assess the worldwide incidence, prevalence, disability-adjusted life years (DALYs) and mortality associated with SSDs in the year 2021, while also exploring trends from 1990 to 2021. This initiative seeks to enhance the comprehensive understanding of the SSD burden and support the development of specific interventions to mitigate its global impact.

## Methods

### Study data

In this study we utilized data from the GBD database, an extensive online collection of current and historical epidemiological studies.^2,3,23^ Since its inception in 1991, the GBD initiative has undergone continuous expansion, spanning 3 decades of rigorous evaluations on the global health landscape. Each iteration has increased coverage, granularity and complexity to include an expanded range of diseases, risks and locations, while refining age group–specific analyses. Notably, GBD 2021 leveraged 328 938 unique data sources to generate >60.7 billion estimates, catering to 25 distinct age groups (ranging from birth to ≥95 y) across 204 countries and territories, categorized into 7 superregions and 21 regions, with gender-disaggregated and combined estimates.

The GBD's methods for data collection and estimation are detailed in this section, adhering to the Guidelines for Accurate and Transparent Health Estimates Reporting (GATHER) principles,^24^ as elaborated in prior GBD publications. Within the scope of this study, 15 SSD categories were identified based on their incidence, prevalence, data adequacy and standardized definitions. These categories include atopic dermatitis, contact dermatitis, seborrheic dermatitis, fungal SSDs, viral SSDs, acne vulgaris, alopecia areata, cellulitis, decubitus ulcer, pruritus, pyoderma, psoriasis, scabies, urticaria and other conditions. The underlying modelling methodology and severity stratification used for each SSD type are comprehensively described in the supplementary information. DALYs were employed as a metric to quantify the burden associated with each SSD, reflecting both years of life lost due to premature death and years lived with a disability.

Annually from 1990 to 2021, data pertaining to SSD incidence, prevalence, DALYs, deaths and corresponding age-standardized incidence rate (ASIR), age-standardized prevalence rate (ASPR), age-standardized DALYs rate (ASDR) and age-standardized mortality rate (ASMR) were systematically gathered using the Global Health Data Exchange Query Tool. These estimates were stratified by geography, gender, age and SSD subtype, partitioning the world into 21 regions and categorizing countries/territories into five sociodemographic index (SDI) strata: low, low-middle, middle, high and high SDI. Notably, our analysis focuses on the causes of the 15 SSDs defined by GBD 2021, which exclude skin neoplasms (e.g. melanoma and other skin cancers) and acute injuries such as burns. These excluded conditions are covered under separate GBD categories (neoplasms and injuries) and are outside the scope of this study.

Detailed descriptions of the comprehensive GBD 2021 methodology, including advanced analytical techniques and model specifications, have been published previously.^3,25^ Uncertainty was quantified by 95% uncertainty intervals (UIs) for each estimate, derived from the distribution of 1000 statistical draws in the GBD modelling process for each disease outcome. These UIs reflect the uncertainty from data sampling, model selection and other sources. The resultant measurements were subsequently stratified by location, gender and age group, providing a nuanced understanding of SSD burden at various levels.^26,27^

### Statistical analysis

We first reported the global and subtype-specific SSD-related incidence, prevalence, deaths and DALYs in 2021. These metrics were categorized by age, sex, SDI, region and country. For disease-specific burden estimation, morbidity metrics were calculated using DisMod-MR 2.1, a Bayesian meta-regression tool that synthesizes heterogeneous epidemiological data while adjusting for covariates such as age, sex and geography. Mortality estimates were generated via the Cause of Death Ensemble model (CODEm),^28^ which integrates vital registration, verbal autopsy and surveillance data through an ensemble of submodels to optimize predictive validity. DALYs were computed by combining years of life lost (YLLs) and years lived with disability (YLDs), with YLDs weighted by severity-specific disability weights from the GBD study. For diseases with varying severity grades, the GBD methodology incorporates severity by assigning disability weights to each severity level. In our study's DALY calculations, each condition's YLD component is already weighted by the proportion of cases in each severity level (as determined by the GBD; see Supplementary Information A for specific severity splits and weights used).

To assess temporal trends for SSDs, we employed a log-linear regression model. The natural logarithm of each age-standardized rate (ASR) was regressed against calendar year using the equation:


\begin{eqnarray*}
\ln \left( {{\mathrm{ASR}}} \right) = \alpha + \beta \times \left( {{\mathrm{Year}}} \right) + \varepsilon ,
\end{eqnarray*}

where α is the intercept, β is the slope coefficient and ε is the random error. The estimated annual percentage change (EAPC) in ASRs was calculated as 100×(expβ−1), capturing the average annual relative change in disease burden metrics over the study period. Methodological validity required three core assumptions: a log-linear association between ASRs and time, homoscedasticity of model residuals across the observation period and independence of residuals to minimize confounding from temporal autocorrelation. To quantify uncertainty, 95% UIs for EAPC estimates were generated using 1000 Monte Carlo simulations. These simulations drew iteratively from the posterior distribution of β, systematically accounting for variability inherent to input data and model parameterization.

To examine temporal patterns in SSD burden projections, the age-period-cohort (APC) model was applied to predict epidemiological trajectories spanning 2022–2046. This methodology disentangles three temporal dimensions: biological aging processes influencing disease susceptibility (age effects), temporal shifts in healthcare practices and diagnostic criteria (period effects) and generational exposure patterns shaped by historical antimicrobial utilization trends (cohort effects).^29^ The statistical framework employed a multiplicative risk structure expressed as: ln(Y)=μ+α (age)=β (period)+γ (cohort)+ε, where μ denotes baseline risk; α, β and γ coefficients quantify temporal dimension contributions; and ε addresses residual dispersion in Poisson-distributed event counts.^30^

Model robustness was assessed through comparative analysis with seasonal autoregressive integrated moving average specifications, optimized through autocorrelation function analysis to minimize prediction errors.

All statistical analyses adhered to rigorous scientific principles and were executed utilizing the R programming environment (version 3.5.3; R Foundation for Statistical Computing, Vienna, Austria). Statistical significance was ascertained at a two-sided p-value threshold of <0.05,^32^ ensuring the robustness and reliability of our findings.

## Results

### Global incidence, prevalence, mortality and DALYs of SSDs from 1990 to 2021

In 2021, an estimated 4.69 billion (95% UI 4.50 to 4.90 billion) new cases of SSDs occurred globally, with an ASIR of 59 092 per 100 000 population (95% UI 56 659 to 61 722), reflecting a modest increase over time (EAPC: +0.23% [95% confidence interval {CI} 0.21 to 0.24]). The number of prevalent cases reached 2.03 billion (95% UI 1.97 to 2.09 billion), corresponding to an ASPR of 25 568 per 100 000 (EAPC: +0.23% [95% CI 0.21 to 0.24]). Global SSD-related deaths totalled 119 129 (95% UI 108 214 to 126 524), with an ASMR of 1.47 per 100 000, showing a slight upward trend (EAPC: +0.15% [95% CI 0.02 to 0.28]). The overall burden measured by DALYs amounted to 41.94 million (95% UI 27.75 to 59.79 million), with an ASDR of 535.3 per 100 000 and a marginal increase over the period (EAPC: +0.05%; 95% CI 0.03 to 0.06) (Table 1, Supplementary Table S1, Supplementary Figures S1 and S2). Detailed information on 15 specific SSDs can be found in Supplementary Information B, with corresponding data presented in Supplementary Figures S21–S35.

### The global trends and burden of SSDs by sex and age from 1990 to 2021

Sex‐stratified analysis shows that from 1990 to 2021 both males and females experienced increasing ASIRs and ASPRs of SSDs, with females consistently reporting higher caseloads (ASIR in 2021 reached 2.3 billion cases in females and ASPR was 26 000 per 100 000 population versus 25 500 in males). The ASMR fluctuated more markedly in females and the ASDR rose to 560 per 100 000 in females compared with 520 in males (Supplementary Figure S3).

**Figure 1.**
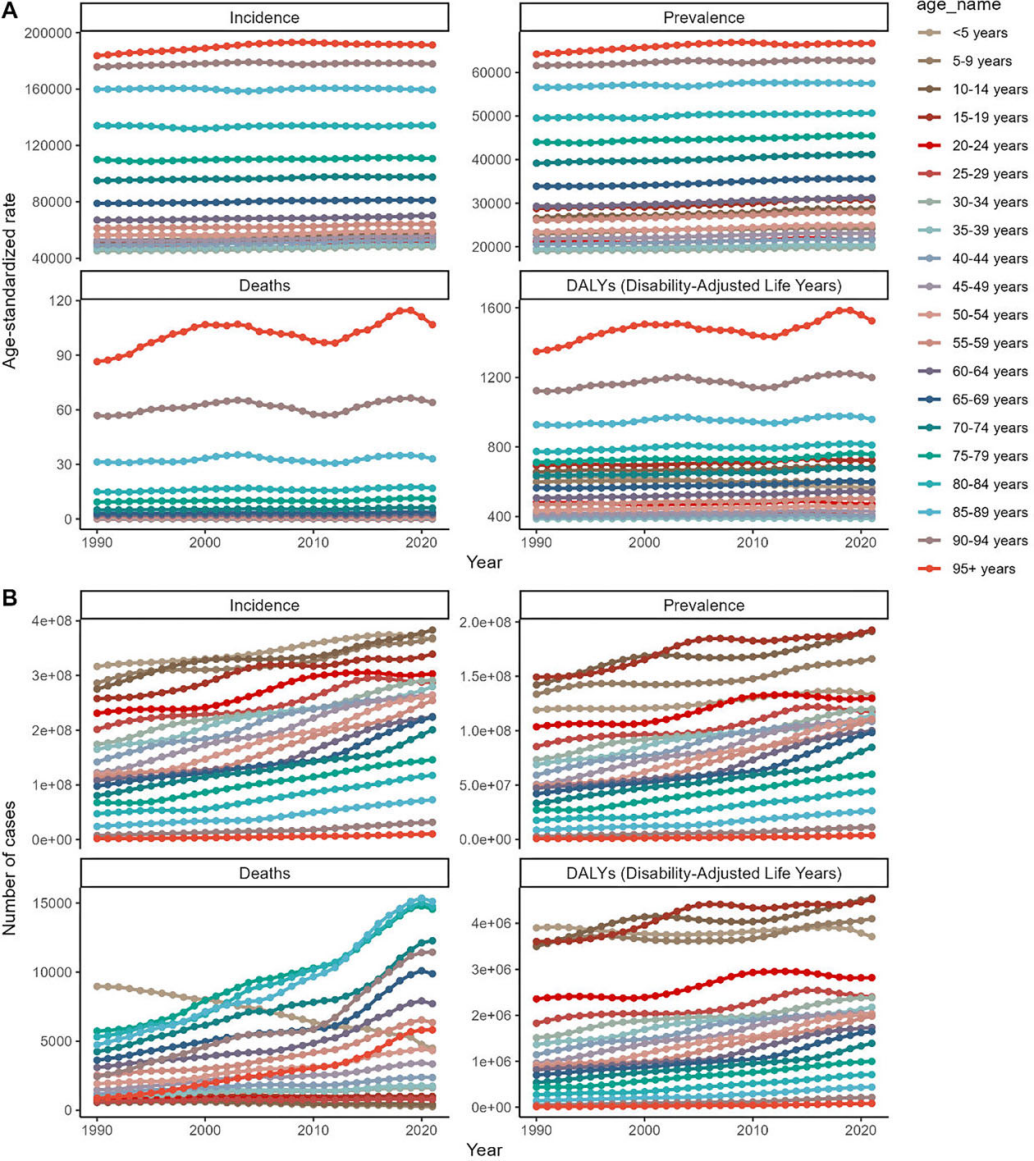
Global trends in **(A)** age-standardized rates and **(B)** incidence, prevalence, deaths and DALYs of SSDs by age from 1990 to 2021.

Age‐specific analysis (Figure 1, Supplementary Figure S4) identified the ≥95-y group as bearing the highest incidence, prevalence, mortality and DALY rates. The 10- to 14-y cohort exhibited the fastest increases in incidence (EAPC: +0.40% [95% CI 0.36 to 0.44]) and prevalence (EAPC: +0.26% [95% CI 0.24 to 0.28]), whereas the 85- to 89-y and 90- to 94-y cohorts showed minimal change (EAPC: 0.00% and +0.06%, respectively). Mortality declined in the <5 and 5- to 29-y age groups (EAPC: −2.42% [95% CI −2.59 to −2.25]) but rose in older cohorts.

### Analysis of SSD burden by SDI region

**Figure 2.**
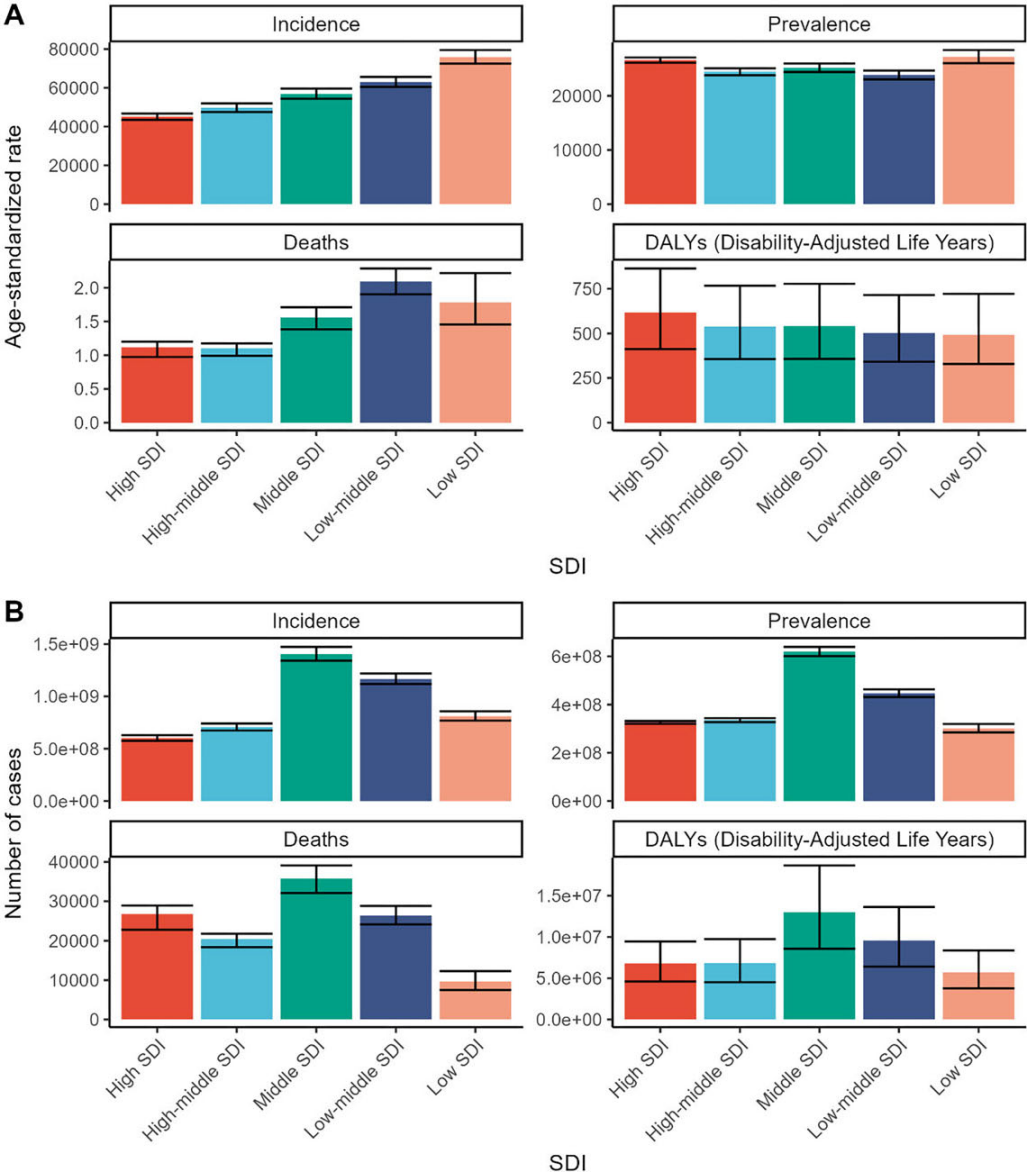
SDI-specific incidence, prevalence, deaths and DALYs of SSDs in 2021. **(A)** Age-standardized rates and **(B)** incidence, prevalence, deaths and DALYs across different SDI levels.

**Figure 3.**
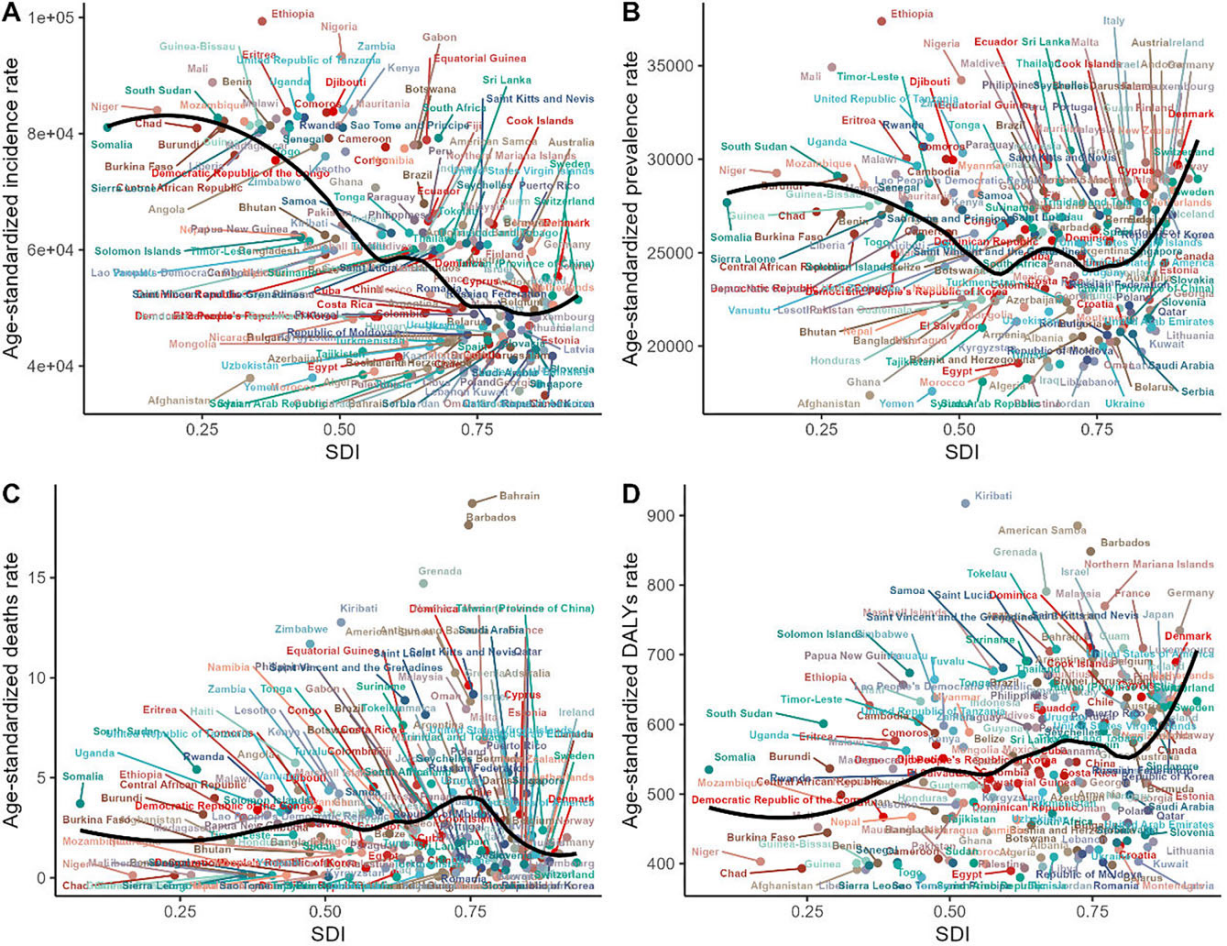
Age-standardized rates for **(A)** incidence, **(B)** prevalence, **(C)** deaths and **(D)** DALYs attributable to SSDs across countries and territories by SDI. An adaptive correlation modelled using Loess regression that adjusts according to all available data is depicted by the black line.

In 2021, the burden of SSDs showed distinct variation across SDI quintiles, both in terms of ASRs and absolute numbers (Figure 2, Table 1). ASIRs were highest in low-SDI and low-middle-SDI regions and progressively decreased with increasing SDI (Figure 3). For example, the ASIR was >75 000 per 100 000 in the lowest SDI quintile, while falling below 50 000 in high-SDI settings. Similarly, death rates were approximately twice as high in low-middle-SDI regions compared with high-SDI ones.

**Table 1.** The number and age-standardized rate of DALYs for SSDs globally and by GBD region in 1990 and 2021

	2021		1990–2021
Characteristics	DALYs, n (95% UI)	ASR, n×10^−5^ (95% UI)	ASDR (95% CI)
Global			
Both	41 944 109 (27 746 948 to 59 789 982)	535.3 (353.96 to 763.28)	0.05 (0.03 to 0.06)
Sex			
Female	21 816 985 (14 468 887 to 31 061 498)	558.08 (369.74 to 795.18)	0.01 (0 to 0.02)
Male	20 127 124 (13 310 320 to 28 728 484)	513.67 (340.21 to 733.03)	0.08 (0.07 to 0.1)
SDI region			
High SDI	6 833 944 (4 518 305 to 9 733 211)	538.2 (355.74 to 767.42)	0.23 (0.22 to 0.23)
High-middle SDI	6 788 696 (4 606 109 to 9 439 622)	616.79 (412 to 863.14)	0.07 (0.06 to 0.08)
Middle SDI	5 724 408 (3 776 365 to 8 363 296)	492.27 (328.38 to 721.54)	−0.1 (−0.12 to 0.07)
Low-middle SDI	13 000 854 (8 572 830 to 18 667 950)	541.29 (357.22 to 777.6)	0.12 (0.1 to 0.13)
Low SDI	9 562 236 (6 401 491 to 13 637 824)	502.49 (340.84 to 714.32)	0.02 (−0.01 to 0.05)
GBD region			
High-income Asia-Pacific	1 089 583 (705 963 to 1 554 152)	629.54 (402.36 to 912.19)	0.12 (0.11 to 0.13)
Central Asia	481 606 (308076 to 705 426)	508.93 (326.07 to 743.46)	0.14 (0.12 to 0.16)
East Asia	7 753 521 (5 013 710 to 11 387 131)	546.92 (353.7 to 799.02)	0.08 (0.07 to 0.09)
South Asia	8 888 988 (5 947 710 to 12 680 113)	489.15 (330.58 to 696.18)	−0.05 (−0.1 to 0)
Southeast Asia	4 290 673 (2 884 568 to 6 136 642)	628.54 (424.01 to 897.52)	0.22 (0.2 to 0.24)
Australasia	180 793 (120 993 to 257 154)	575.2 (379.53 to 814.38)	0.22 (0.21 to 0.24)
Caribbean	275 742 (189 520 to 385 094)	583.9 (400.57 to 816.36)	0.15 (0.13 to 0.16)
Central Europe	498 875 (331 067 to 713 363)	440.6 (289.7 to 628.75)	0.21 (0.18 to 0.24)
Eastern Europe	974 056 (671 140 to 1 349 426)	488.61 (331.84 to 681.33)	0.31 (0.28 to 0.34)
Western Europe	2 935 720 (2 028 590 to 4 081 413)	657.82 (443.63 to 925.26)	0.14 (0.12 to 0.15)
Andean Latin America	396 759 (258757 to 567 330)	607.58 (396.92 to 869.42)	0.14 (0.1 to 0.17)
Central Latin America	1 337 051 (924351 to 1 871 991)	534.7 (368.61 to 749.86)	0.33 (0.3 to 0.36)
Southern Latin America	435 327 (310 324 to 590 230)	632.73 (442.36 to 870.65)	0.53 (0.49 to 0.57)
Tropical Latin America	1 472 179 (1 005 626 to 2 098 254)	658.49 (445.66 to 940.12)	0.29 (0.25 to 0.34)
North Africa and Middle East	2 521 094 (1 683 311 to 3 545 537)	412.8 (276.01 to 579.97)	0.23 (0.22 to 0.24)
High-income North America	2 412 642 (1 675 356 to 3 283 744)	634.71 (433.62 to 880.36)	0.08 (0.06 to 0.1)
Oceania	92 567 (62 260 to 133 148)	668.61 (461.67 to 953.84)	0.06 (0.04 to 0.08)
Central SSA	658 283 (444 620 to 949 944)	483.9 (339.51 to 682.69)	−0.16 (−0.18 to 0.13)
Eastern SSA	2 539 974 (1 674 399 to 3 732 891)	578.69 (391.25 to 848.9)	−0.16 (−0.18 to 0.13)
Southern SSA	377 025 (260 607 to 533 231)	492.87 (344.43 to 694.7)	0.13 (0.06 to 0.19)
Western SSA	2 331 651 (1 495 672 to 3 505 882)	457.42 (290.11 to 697.9)	0.24 (0.23 to 0.26)

In contrast, the ASPR followed a U-shaped curve across SDI levels. Both low- and high-SDI regions showed relatively high ASPRs, while middle-SDI levels exhibited the lowest prevalence. This may reflect a dual burden: infectious diseases dominate in lower SDI settings, while chronic inflammatory conditions prolong disease duration in higher SDI groups.

However, DALYs showed a different pattern. ASDRs were highest in high-SDI and middle-SDI regions and lowest in low-SDI regions, contradicting the U-shaped trend of prevalence. The highest ASDR reached 616.8 per 100 000 in high-SDI regions compared with <500 per 100 000 in low-SDI areas.

Middle-SDI countries had the highest counts of incidence, prevalence, deaths and DALYs due to their large populations. For example, >1.4 billion incident SSD cases occurred in middle-SDI regions in 2021.

Trends from 1990 to 2021 (Supplementary Figures S5 and S6) further revealed that ASIRs and ASPRs increased across all SDI groups, particularly in high-middle- and middle-SDI countries, whereas low-SDI regions showed modest increases or plateauing trends. In contrast, the ASDR remained largely stable or declined slightly, with the most pronounced decrease observed in low-SDI regions (EAPC: −0.10% [95% CI −0.12 to −0.07). Notably, DALY rates diverged: while they decreased in low- and low-middle-SDI regions (EAPC: −0.83% and −0.18%, respectively), they rose significantly in high- and high-middle-SDI areas (EAPC: +0.58% and +0.84%, respectively), reflecting the increasing burden of chronic, non-fatal dermatoses in more developed settings.

### Temporal shifts in SSD composition across SDI strata

**Figure 4.**
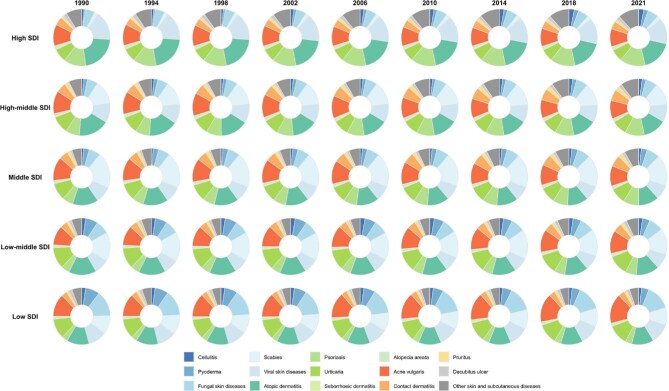
SDI-stratified composition of 15 SSD-related DALYs, 1990–2021.

Across 1990–2021 the composition of SSD burden measured by the ASDR changed substantially with sociodemographic development. The SDI-stratified pie charts show that high-SDI regions (top row) saw an increasing proportion of immune-mediated and inflammatory skin diseases: the green (atopic dermatitis, psoriasis, urticaria, alopecia, seborrheic) and orange/yellow (acne, pruritus, contact dermatitis) segments expanded over time, while the blue infectious disease segments contracted. Conversely, low- and low-middle-SDI regions (bottom rows) remain dominated by infectious SSDs, though even here the pie charts indicate a modest decline in their share from 1990 to 2021. Non-infectious categories (green/orange) in these regions have grown. Middle-SDI countries lie in between: in 1990 their burden was mostly infection-driven, but by 2021 the slices for immune and exposure-related diseases had visibly increased. The most dramatic shifts are seen in the highest and lowest SDI strata. For example, the high-SDI pies show a large increase in psoriasis (dark green) and acne (orange), while the low-SDI pies show growing segments for acne and atopic dermatitis (Figure 4). In sum, by 2021 infectious SSDs still dominate low-SDI settings, but immune/inflammatory and other causes had risen in prominence across all SDI levels.

Other SDI-stratified composition trends are detailed in Supplementary Figures S7–S11. Notably, Supplementary Figure S7 demonstrates a shift from infectious to immune-mediated SSDs in DALY cases across the SDI gradient from 1990 to 2021. Supplementary Figures S8 and S9 reveal that incidence and prevalence burdens of fungal infections and scabies decreased in high-SDI countries, with corresponding increases in urticaria and psoriasis. Supplementary Figures S10 and S11, which assess ASIR and ASPR, indicate a persistent and widening burden of chronic SSDs in higher SDI strata.

## Region distribution of SSDs in 2021

Geographically, sub-Saharan Africa (SSA) accounted for the highest SSD incidence in 2021, with the Central (98.5 million cases [95% UI 92.5–106.0 million]), Eastern (87.3 million [95% UI 83.3–92.0 million]), Western (86.5 million [95% UI 82.3–91.0 million]) and Southern (78.6 million [95% UI 75.2–82.1 million]) subregions leading globally (Supplementary Table S1; Supplementary Figure S12). East Asia also recorded a substantial incidence (781.9 million [95% UI 741.0–821.5 million]), while Oceania reported 8.3 million cases (95% UI 7.9–8.9 million). Prevalence was highest in tropical Latin America (64.8 million [95% UI 62.8–67.1 million]), central Latin America (59.3 million [95% UI 57.6–61.0 million]) and Australasia (9.7 million [95% UI 9.6–9.9 million]). The greatest DALY burdens occurred in western (144.1 million [95% UI 135.1–154.4 million]) and eastern (133.3 million [95% CI 125.7–141.7 million]) SSA, with central SSA contributing 35.3 million DALYs (95% UI 31.9–39.0 million). Mortality was highest in tropical Latin America, followed by Australasia and high-income North America (7 668 deaths [95% UI 6589–8657]) (Table 1, Supplementary Figure S12).

Regional analyses revealed heterogeneous trends. Most regions saw 0–200% increases in incidence and prevalence, while mortality changes ranged from declines (e.g. Eastern Europe) to dramatic increases of up to 1400% in parts of South America and Central Africa. DALYs surged notably in select African, South American and Asian regions (Supplementary Figure S13). Hierarchical clustering of ASR EAPCs (incidence, prevalence, mortality, DALYs) identified four ‘high‐increase’ regions (Eastern Europe, Western Africa, tropical Latin America and South Asia) and four ‘decrease’ regions (Central Europe, Australia, Central Asia and Latin America and Caribbean) (Supplementary Figure S14).

**Figure 5.**
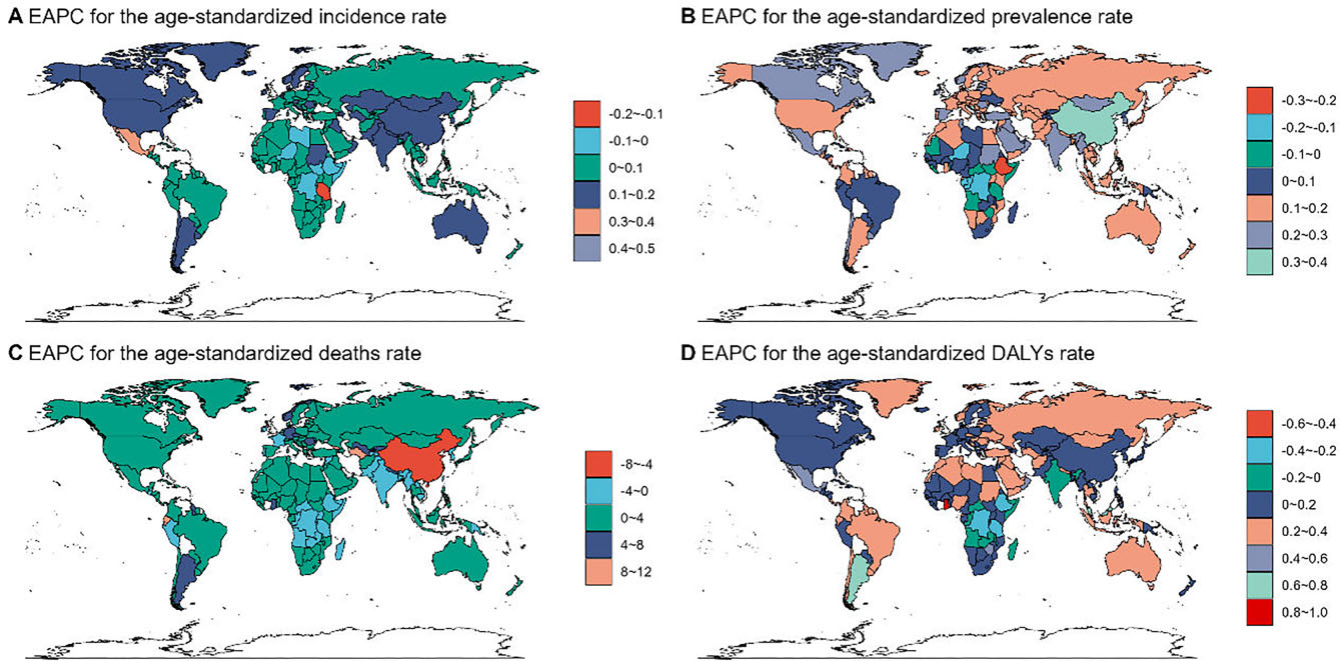
The global distribution of EAPCs for age-standardized rates for **(A)** incidence, **(B)** prevalence, **(C)** deaths and **(D)** DALYs of SSDs from 1990 to 2021.

Figure 5 summarizes global EAPC patterns for ASRs, showing a general upward trajectory in incidence and prevalence rates, with minor decreases in certain European and Asian locales. Mortality and DALY rate trends diverge: several Asian and South American regions exhibited reductions, whereas others continued to experience increasing SSD‐related mortality and disability.

### Burden of SSDs by hierarchical disease categories in 2021

**Figure 6.**
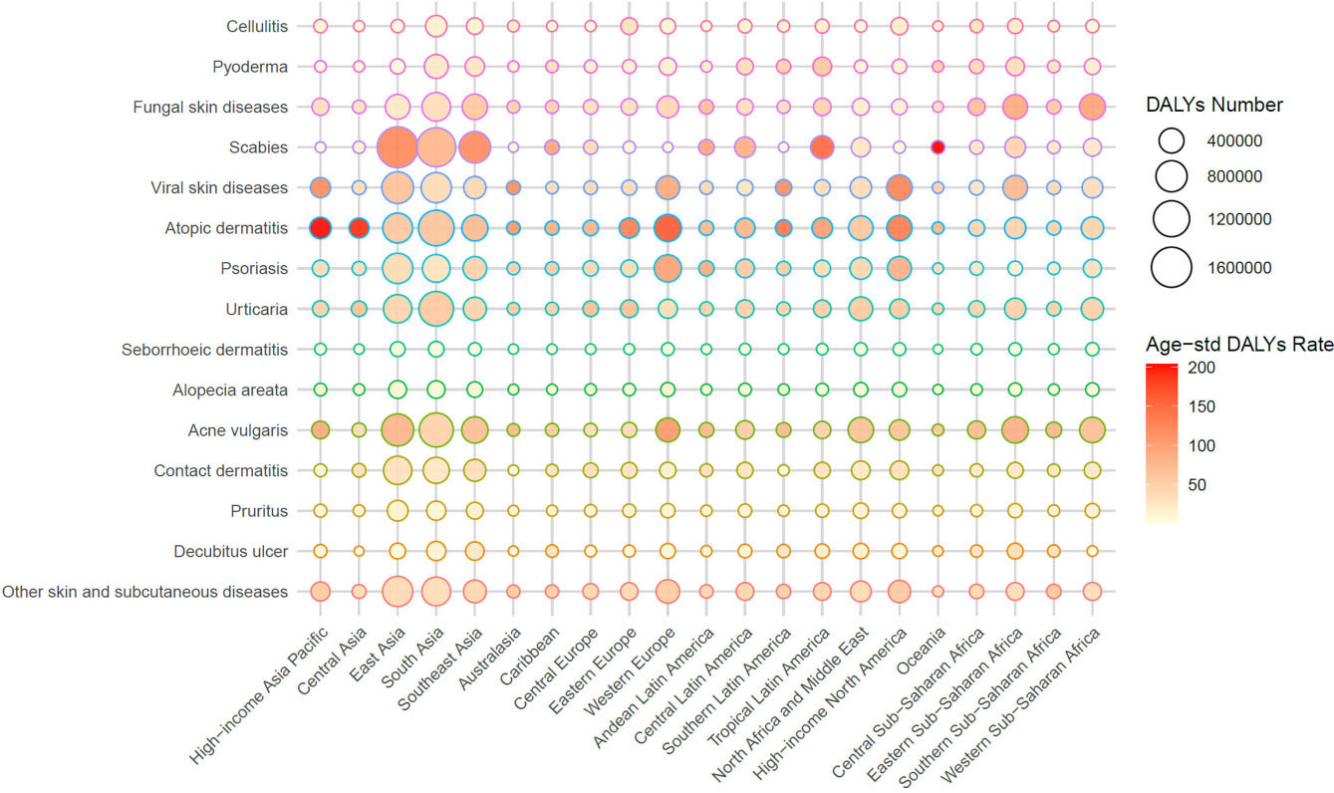
Geographic distribution of 2021 DALYs for 15 SSDs.

In 2021, the burden of SSDs, as measured by DALYs, exhibited substantial geographic variation across regions and disease types (Figure 6). Several tropical and low- to middle-income regions, including South Asia, tropical Latin America and western SSA, showed the largest total DALYs for multiple conditions, particularly for infectious and parasitic dermatoses such as fungal skin diseases, scabies and pyoderma. These diseases presented with both large absolute DALY numbers (larger bubble size) and elevated age-standardized DALY rates (darker red colour), reflecting high disease prevalence, severity and inadequate access to treatment.

In contrast, in high-SDI regions such as high-income North America, Western Europe and Australasia, the DALY burden was dominated by non-communicable and immune-mediated skin conditions, including atopic dermatitis, psoriasis and acne vulgaris. Although the overall DALY burden was lower than in tropical regions, these conditions showed significant ASDRs, indicating their persistent impact on quality of life. For instance, atopic dermatitis in high-income Asia-Pacific areas and acne vulgaris in North America contributed large, brightly coloured bubbles, highlighting substantial per capita burden.

Notably, the burden of pruritus and contact dermatitis was relatively evenly distributed across regions, suggesting that these conditions are widespread but not necessarily region specific. Pressure-related skin conditions such as decubitus ulcers also contributed to the DALY burden in both aging and low-resource populations, including Central and Eastern Europe and SSA.

Supplementary Figures S15 and S16 map the 2021 geographic distributions of SSD incidence and prevalence by subtype, highlighting distinct epidemiological patterns such as the concentration of scabies and fungal SSDs in SSA and the prevalence of psoriasis and acne in Europe and high-SDI Asia-Pacific areas.

### Regional composition shifts in SSDs

**Figure 7.**
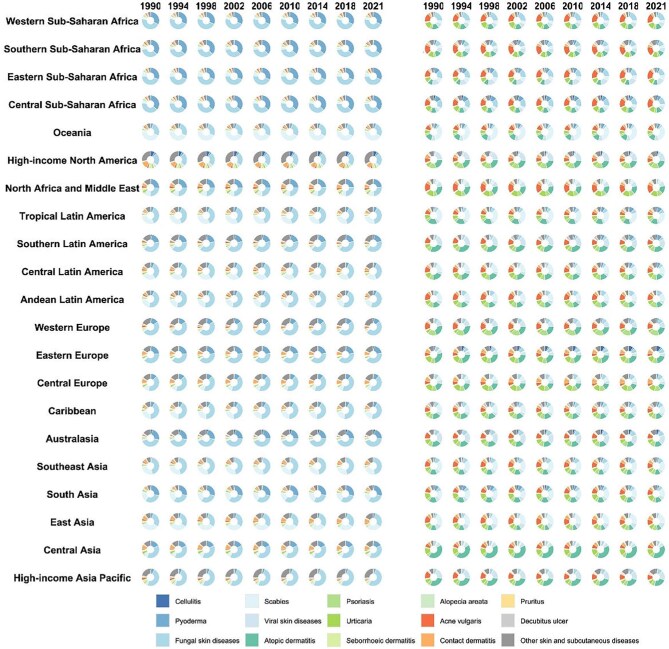
Geographic region–stratified composition of SSD-related incidence and DALYs, 1990–2021.

Figure 7 illustrates compositional changes in ASDR and ASIR attributable to SSDs across 21 GBD regions from 1990 to 2021. A clear global shift is observed: high-SDI regions transitioned from infectious to immune-mediated and inflammatory diseases, while low-SDI regions remain dominated by infections.

In SSA, all subregions (Western, Eastern, Central, Southern) remained heavily burdened by infectious diseases, particularly fungal infections, pyoderma and scabies. Only minor increases were seen in acne and dermatitis. By 2021, incidence and DALY compositions in these regions were still dominated by infectious causes, reflecting limited transition despite global trends. In contrast, high-income regions such as Western Europe, North America and Australasia showed substantial shifts. By 2021, infectious SSDs had nearly disappeared from the disease composition, replaced by acne, atopic dermatitis, psoriasis and contact dermatitis. DALY pies in these regions were largely composed of non-communicable conditions, highlighting lifestyle-driven burden patterns. Central and Eastern Europe followed a moderate trajectory. While starting with significant fungal and bacterial burdens in 1990, these regions saw increased shares of acne and dermatitis by 2021, particularly in Central Europe. Eastern Europe retained a relatively higher infectious share but still trended toward a more inflammatory profile. In Latin America, subregional variation was evident. Tropical and Andean Latin America maintained higher infectious burdens, whereas southern and central Latin America experienced visible growth in non-infectious diseases, especially acne, psoriasis and dermatitis, suggesting a socio-economic gradient in disease transition. North Africa and the Middle East showed relatively stable SSD composition. Dermatitis remained predominant, while infectious diseases declined modestly. Acne and urticaria gradually increased but did not dramatically alter the overall profile. South and Southeast Asia experienced gradual transitions. South Asia remained dominated by fungal infections and pyoderma, although acne and dermatitis gained slightly. Southeast Asia showed more noticeable growth in atopic dermatitis and urticaria, although infections still contributed substantially to the burden in 2021. East Asia showed a more pronounced transition. Inflammatory conditions like acne, atopic dermatitis and psoriasis became dominant by 2021, replacing earlier infectious burdens. Central Asia followed a similar trend, although infectious diseases persisted longer before declining. Oceania and Australasia presented a sharp contrast. In Oceania, scabies and parasitic skin diseases continued to dominate throughout the period. In contrast, Australasia aligned with high-income patterns, with inflammatory SSDs accounting for most of the burden by 2021.

Across the 21 GBD regions, Supplementary Figures S17–S20 demonstrate marked differences in incidence, DALYs, prevalent cases and the corresponding ASPR from 1990 to 2021. In regions such as western SSA, South Asia and Southeast Asia, fungal infections, scabies and acne vulgaris consistently represented the largest proportions of DALYs and incidence. In contrast, Western Europe, high-income North America and Australasia showed greater contributions from chronic inflammatory conditions such as atopic dermatitis, psoriasis and urticaria. Similar patterns were observed in age-standardized prevalence rates, with immune-mediated disorders comprising a substantial burden in developed regions.

### Predicted trends of SSDs by sex from 2022 to 2046

**Figure 8.**
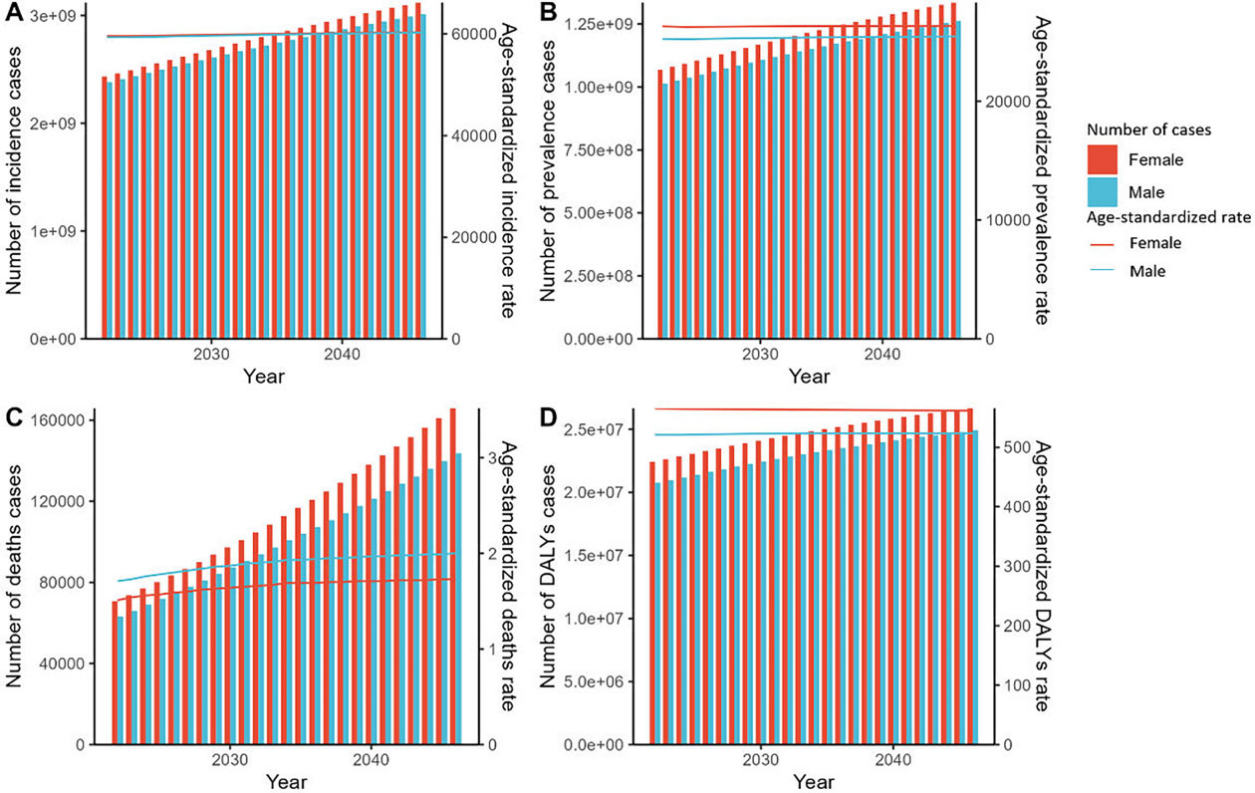
The predicted results in SSD numbers and age-standardized rates for incidence, prevalence, deaths and DALYs by sex globally from 2022 to 2046 by the APC model.

Based on the APC model projections for the period 2022–2046, both genders are anticipated to experience an increase in the incidence, prevalence, deaths and DALY cases, accompanied by relatively stable ASRs across all categories (Figure 8). Additionally, females are expected to consistently exhibit higher numbers and ASRs in all categories compared with males over the forecasting period.

## Discussion

Our analysis of the global, regional and local patterns in the incidence, mortality and DALYs of SSDs from 1990 to 2021, segmented by age, sex and subtype, uncovered intricate dynamics in the disease burden. Notwithstanding promising declines in SSD cases and related healthcare expenses in specific areas and nations, largely due to primary and secondary prevention measures such as UV protection and early self-diagnosis, the overall global burden of SSDs continues to increase, as indicated by increasing ASIRs and ASMRs.^18,21,33,34^

Globally, a gradual reduction in the ASMR and DALYs of SSDs from 1990 to 2021 has been observed and can be attributed to the proactive implementation of preventive measures, which include self-examination initiatives, public education campaigns and the introduction of innovative treatment modalities over the past few decades. However, the global ASIR of SSDs has exhibited a notable upward trend over this period, likely influenced by the aging of the global population.^35,36^

From 1990 to 2021, an upsurge in ASRs of SSDs can be observed in both sexes, while the caseloads have always been higher in women. Hormonal differences can be a significant aetiological reason, which can influence skin conditions such as acne and autoimmune disorders like lupus erythematosus. Additionally, psychosocial stressors, prevalent among women due to societal roles and responsibilities, may exacerbate conditions like eczema and psoriasis, indicating the need for gender-sensitive healthcare policies aimed at reducing morbidity and improving quality of life.

Age-wise, the highest incidence rates are observed in the oldest age group. Aging is inherently associated with weakened immune responses and skin barrier function, making older adults more prone to infections, chronic inflammatory conditions like psoriasis and malignancies such as skin cancer. Additionally, comorbidities and polypharmacy prevalent in this demographic can exacerbate skin conditions, complicating management and increasing morbidity.

Furthermore, our analysis uncovers regional disparities in SSD-related disease burden. The relationship between latitudinal gradient and SSDs emerges as a pivotal aetiological factor, complementing the well-established role of UV radiation exposure. Previous studies have revealed that individuals with higher melanin pigmentation, who naturally possess enhanced UV protection via their epidermal melanin, exhibit a reduced risk of SSDs compared with those with lighter Caucasian skin tones,^37–39^ consistent with our findings. Notable elevations can be observed in regions further from the equator. This spatial distribution mirrors the latitudinal gradient in human skin pigmentation, where darker skin tones are prevalent near the equator and lighter skin tones are more common at higher latitudes.^40^ While constitutive melanin pigmentation provides intrinsic photoprotection, congenital and acquired pigmentation disorders such as albinism and vitiligo markedly amplify photodamage risks. In albinism, melanin deficiency disrupts UV filtration, leading to cumulative DNA damage.^41^ Similarly, vitiligo-associated depigmentation diminishes epidermal antioxidant capacity, exacerbating oxidative stress and inflammatory responses to UV radiation.^42^ These vulnerabilities disproportionately affect populations in tropical latitudes, where intense UV exposure intersects with limited access to photoprotective resources.^43^ For instance, our data revealed elevated SSD mortality in southern Latin America and southern SSA, likely reflecting both environmental and biological risk stratification. This underscores the necessity of integrating dermatological care with environmental health policies, particularly for genetically predisposed subgroups in low-resource, high-UV settings.

Despite the encouraging global decline in SSD-related DALYs and mortality over the past 3 decades, particularly in high-income North America, underdeveloped regions with rapid population growth continue to grapple with adverse trends, which highlights the impact of poverty in disease burden, in addition to climate. Moreover, regions like Eastern Europe have demonstrated a decrease in SSD incidence rates, potentially attributable to effective public health interventions and improved socio-economic conditions.^44,45^ Nevertheless, the scenario in southern SSA remains dire, with an exceptionally high ASR of incidence in 2021 relative to other regions globally. In southern SSA, the lack of effective treatment for SSD patients is a complex issue. Contributing factors include limited healthcare infrastructure, low socio-economic conditions, poor disease awareness and difficulties in identifying pigment lesions on darker skin. Additionally, delays in early detection and timely treatment are exacerbated by a significant immunosuppression burden due to the prevalence of human immunodeficiency virus/acquired immunodeficiency syndrome and other socio-cultural barriers.^46^

Emerging evidence highlights the significant role of environmental pollutants in the pathogenesis of chronic skin diseases. Ambient ozone and particulate matter pollution have been linked to oxidative stress and systemic inflammation, which may exacerbate skin conditions such as eczema and psoriasis.^47,48^ Indoor air pollution, particularly from biomass combustion and volatile organic compounds, further compounds these effects by increasing direct dermal exposure and impairing skin barrier function.^49^ Notably, studies suggest that indoor pollutants may pose a greater risk than ambient pollution in certain settings, such as hospitals and households.^50^ While limited data directly connect nitrous oxide or lead poisoning to skin diseases, their systemic pro-inflammatory effects could indirectly contribute to dermatological pathologies.^51^ The interplay between these pollutants and skin health underscores the need for integrated mitigation strategies targeting both indoor and ambient sources to reduce the burden of environmentally driven skin diseases.

The APC analysis indicates that incidence, prevalence, deaths and DALYs attributable to SSDs are expected to continue increasing, coupled with stable ASRs, suggesting that while the risk per individual may not be changing significantly, the growing and aging population will likely lead to an increased burden on healthcare systems. This could result in greater demand for dermatologic services, preventive measures and treatment resources.

The gender disparity highlighted by these projections is particularly noteworthy. The consistently higher numbers in females could be influenced by a combination of biological, behavioural and societal factors. For instance, females might have a greater awareness and reporting of skin conditions, or there could be intrinsic biological susceptibilities that result in a higher incidence and burden of these diseases. Future research endeavours should meticulously evaluate the efficacy of diverse prevention technologies and therapeutic options in mitigating the SSD burden. This includes examining the impact of UV protection products, early screening initiatives and public health campaigns aimed at mitigating risk factors, notably UV radiation exposure. Additionally, delving deeper into the socio-economic determinants of SSD burden is crucial. Investigating how advancements in healthcare infrastructure, education and economic status can contribute to reducing SSD incidence and mortality rates can inform the formulation of more effective global health policies.

Our study builds upon and complements previous research, notably Chu et al.’s^22^ examination of SSD burden in Europe from 1990 to 2017, which emphasized morbidity and DALYs, and Urban et al.’s^18^ focus on Asia, particularly the association between SSD-related DALYs and socio-economic status over the same time frame.

Although our study drawing on the GBD 2021 database to quantify global incidence, prevalence, DALYs and mortality for SSDs provides valuable insights into temporal and regional patterns, it is subject to several important limitations. First, the absence of histological subtype classifications and risk factor–specific data within the GBD 2021 prevents more granular subgroup analyses of SSD burden. Second, data heterogeneity and underreporting may undermine the precision of our estimates, and the observational nature of our analysis precludes causal inference while leaving unmeasured confounders such as disparities in healthcare access and quality, socio-economic status, cultural practices and environmental exposures unaccounted for. Third, by focusing exclusively on the SSD categories defined by the GBD, we inadvertently omit significant sources of skin‐related mortality and disability, namely severe burn injuries, malignant skin cancers and endemic infections (e.g. leprosy, cutaneous leishmaniasis, onchocerciasis), which disproportionately affect regional DALY burdens. Finally, because the GBD 2021 does not provide population-attributable fractions for SSDs nor allow linkage with country‐level Healthcare Access and Quality (HAQ) Index scores, our study cannot apportion disease outcomes to specific environmental or behavioural risks or assess how health system performance mitigates SSD burden. To overcome these gaps, future burden‐of‐disease research should integrate detailed exposure datasets (such as satellite‐derived UV indices, air pollutant concentrations and meteorological records), calculate population attributable fractions, incorporate independently sourced HAQ metrics and include region‐specific endemic disease profiles, thereby enabling stronger causal insights, more targeted intervention prioritization (e.g. UV‐protection campaigns, air quality improvements, climate adaptation measures) and evidence‐based health system strengthening to reduce the global SSD burden.

## Conclusions

In conclusion, our research underscores the considerable differences in the burden of SSDs across countries and regions, establishing SSDs as a vital international public health concern. Our findings suggest that the prevalence of SSD cases will continue to increase over the next 23 y, highlighting the persistent importance of addressing SSDs as a public health challenge. This emphasizes the urgent need for tailored, region-specific strategies to combat SSDs and tackle their root causes on a global scale.

## Supplementary Material

ihaf070_Supplemental_Files

## Data Availability

Datasets that are publicly accessible were analysed. The data can be found at https://vizhub.healthdata.org/gbd-results/.
