# Supplementary material for: Global, regional and national burden of skin and subcutaneous diseases: a systematic analysis of the Global Burden of Disease Study 2021
Source: Int Health. 2025 Jun 28;18(2):183–96. doi: 10.1093/inthealth/ihaf070 (PMC13017215; doi:10.1093/inthealth/ihaf070)
Supplement: ihaf070_Supplemental_Files [file ihaf070_supplemental_files.zip › Supplementary Figures.docx]

**Supplementary Figures**


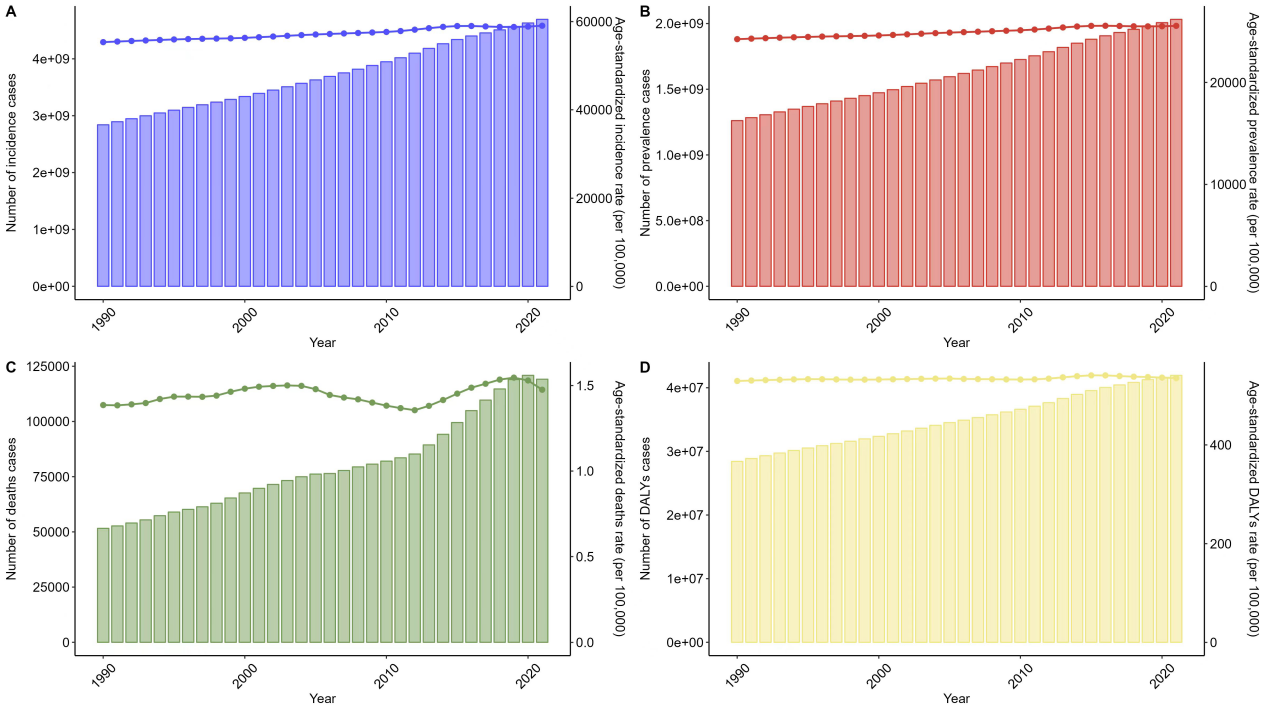


**Supplementary** Figure S1. Global trends in numbers and age-standardized rates for (A) incidence, (B) prevalence, (C) deaths and (D) DALYs of SSDs from 1990 to 2021. Abbreviations: DALYs, disability-adjusted life years; SSDs, skin and subcutaneous diseases.


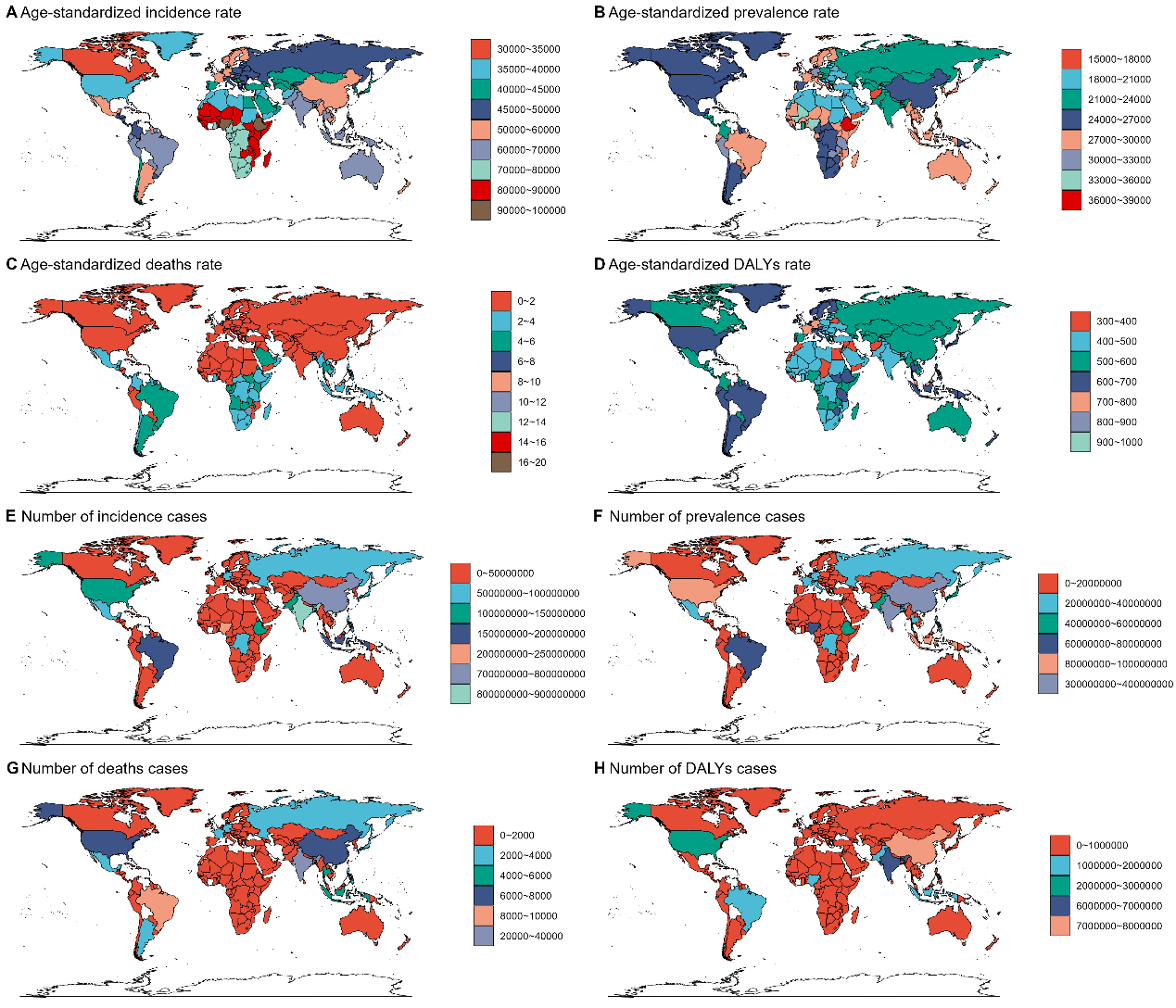


**Supplementary Figure S2.** The global distribution of age-standardized rates of SSDs-related incidence, prevalence, deaths, and DALYs in 2021. Abbreviations: DALYs, disability-adjusted life years; SSDs, skin and subcutaneous diseases.


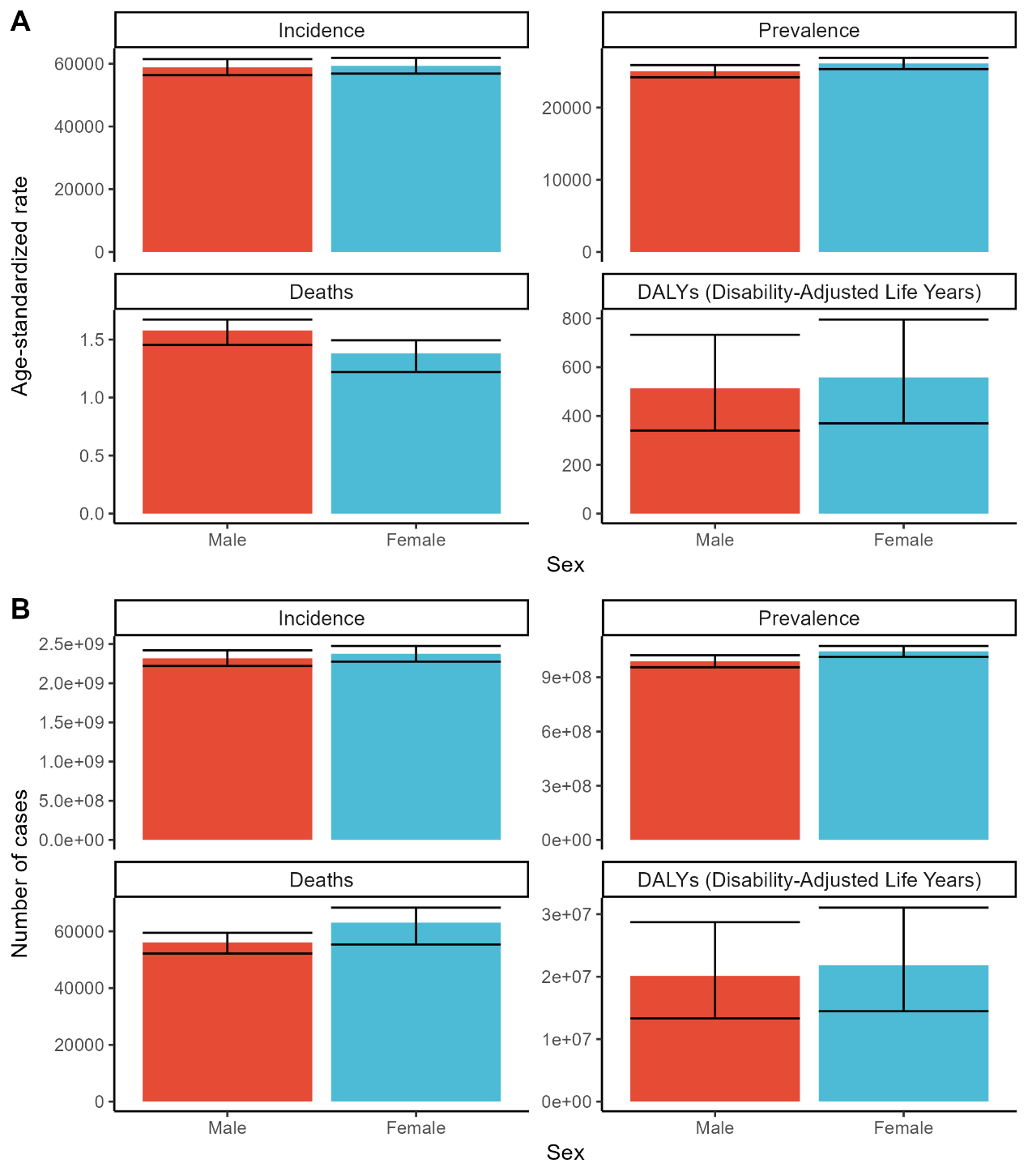


**Supplementary Figure S3.** The age-standardized rates and numbers of SSDs in incidence, prevalence, deaths and DALYs by sex in 2021. Abbreviations: DALYs, disability-adjusted life years; SSDs, skin and subcutaneous diseases.


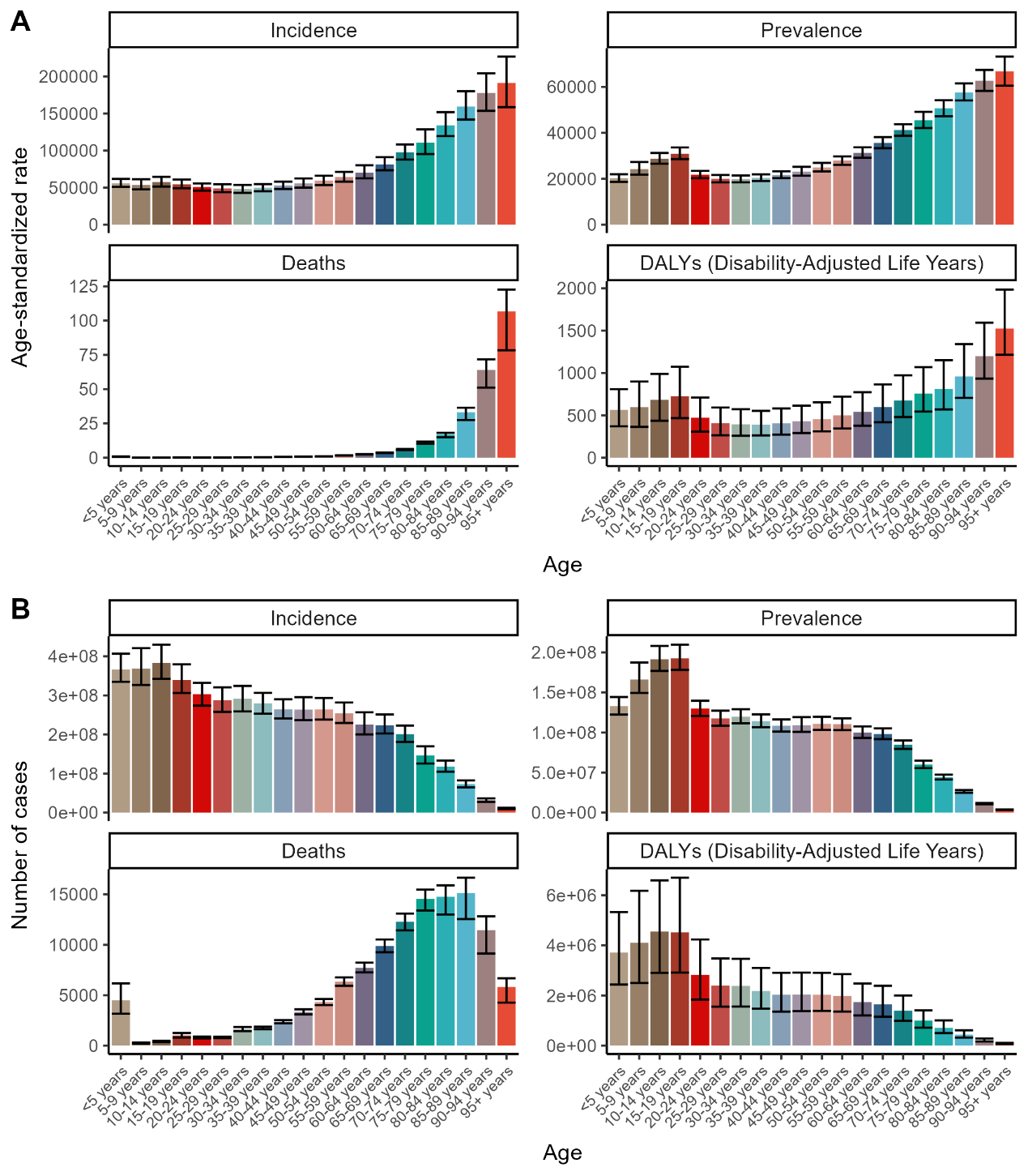


**Supplementary Figure S4.** The age-standardized rates and numbers of SSDs in incidence, prevalence, deaths and DALYs by age in 2021. Abbreviations: DALYs, disability-adjusted life years; SSDs, skin and subcutaneous diseases.


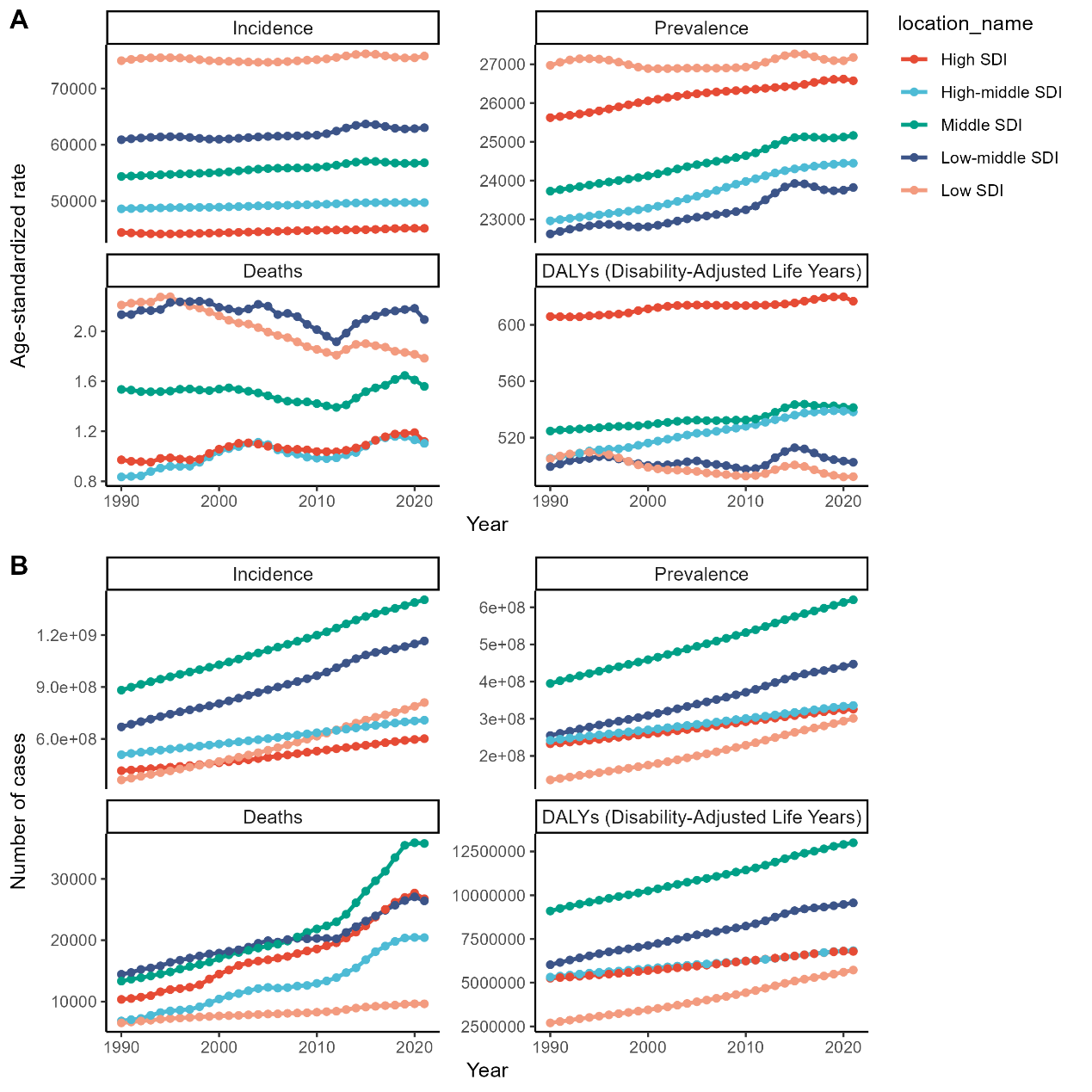


**Supplementary Figure S5.** Global trends in numbers and age-standardized rates for **(A)** incidence, **(B)** prevalence, **(C)** deaths and **(D)** DALYs of SSDs by SDI level from 1990 to 2021. Abbreviations: DALYs, disability-adjusted life years; SDI, socio-demographic index; SSDs, skin and subcutaneous diseases.


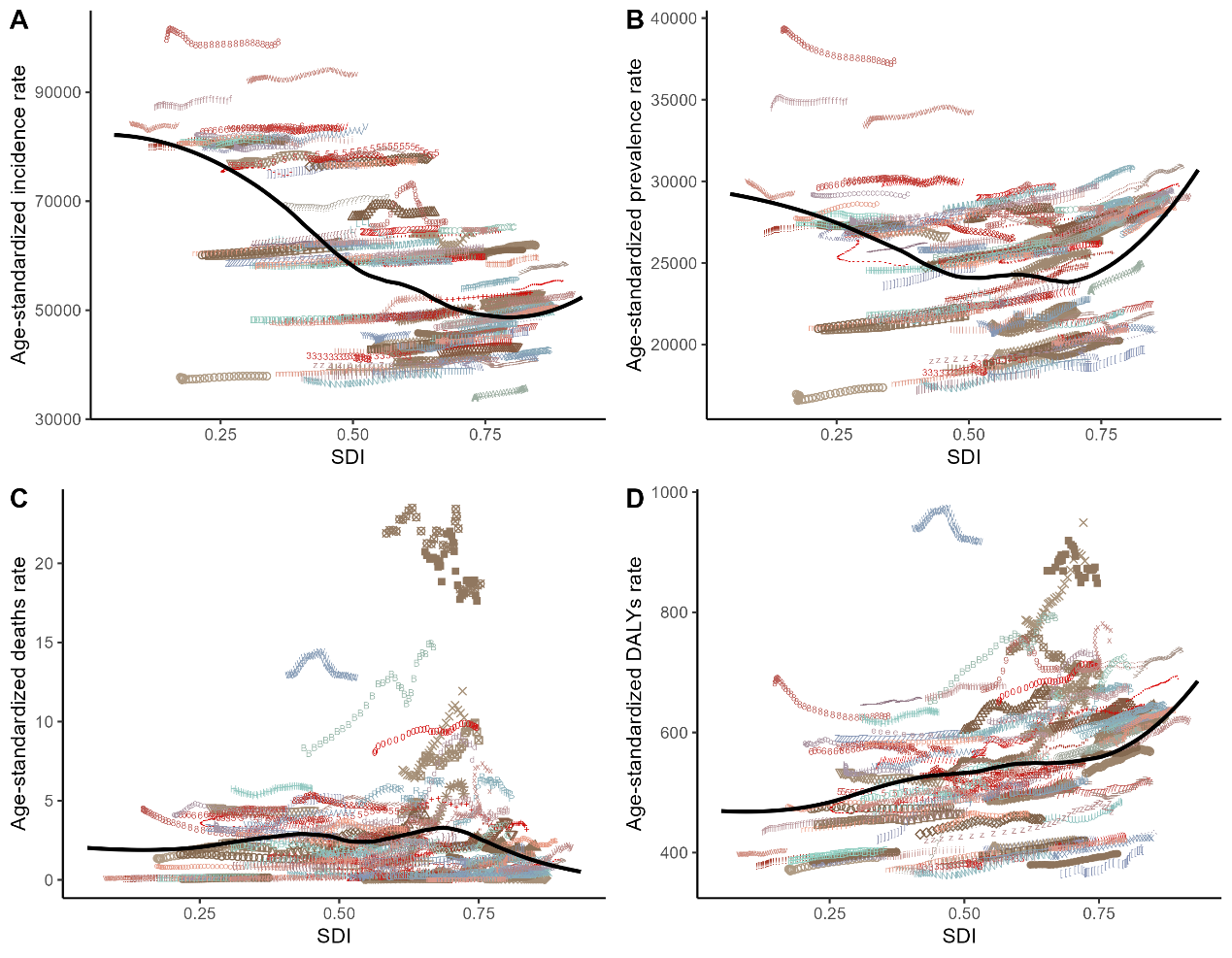


**Supplementary Figure S6.** Relationship between SDI and age-standardized rates of SSDs from 1990 to 2021. Abbreviations: SDI, socio-demographic index; SSDs, skin and subcutaneous diseases.

**
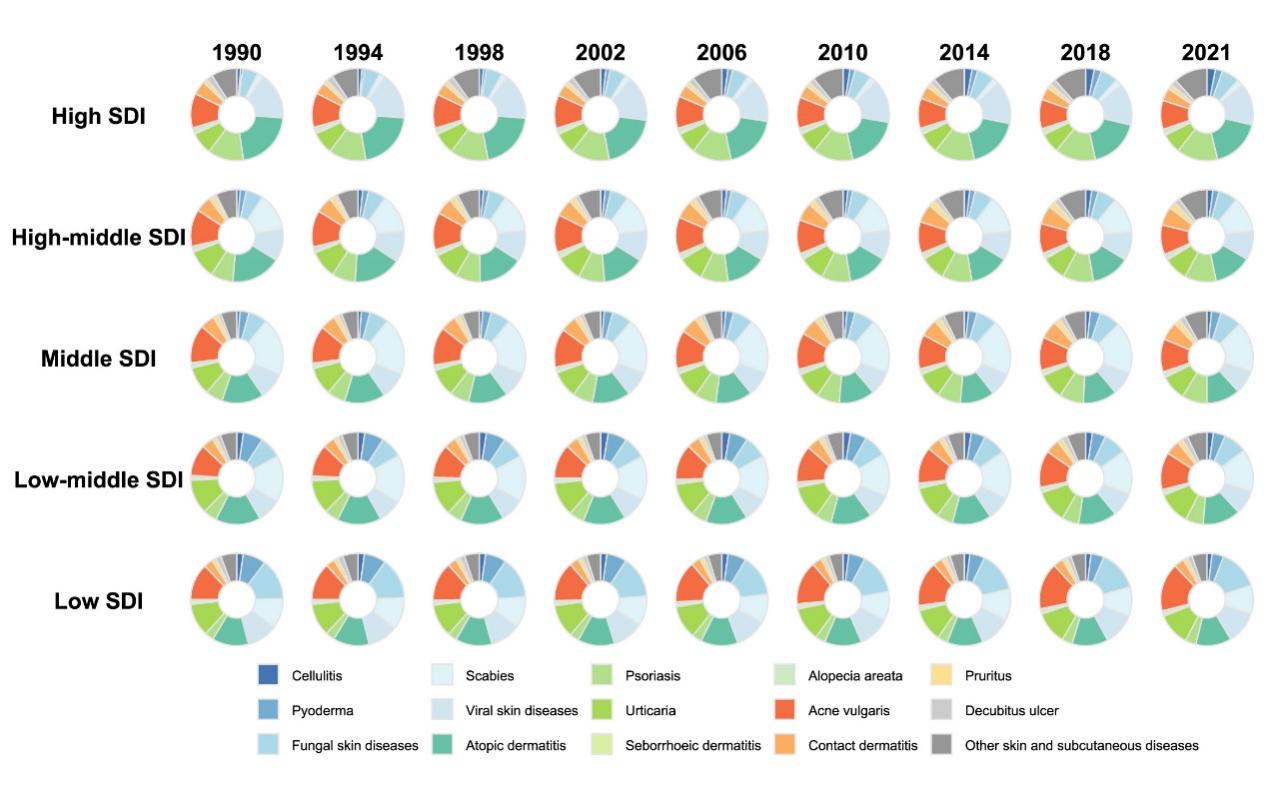
**

**Supplementary Figure S7.** SDI-Stratified Composition of 15 SSDs-related DALYs cases, 1990–2021. Abbreviations: SSDs, skin and subcutaneous diseases; SDI, socio-demographic index; DALY, disability-adjusted life years.


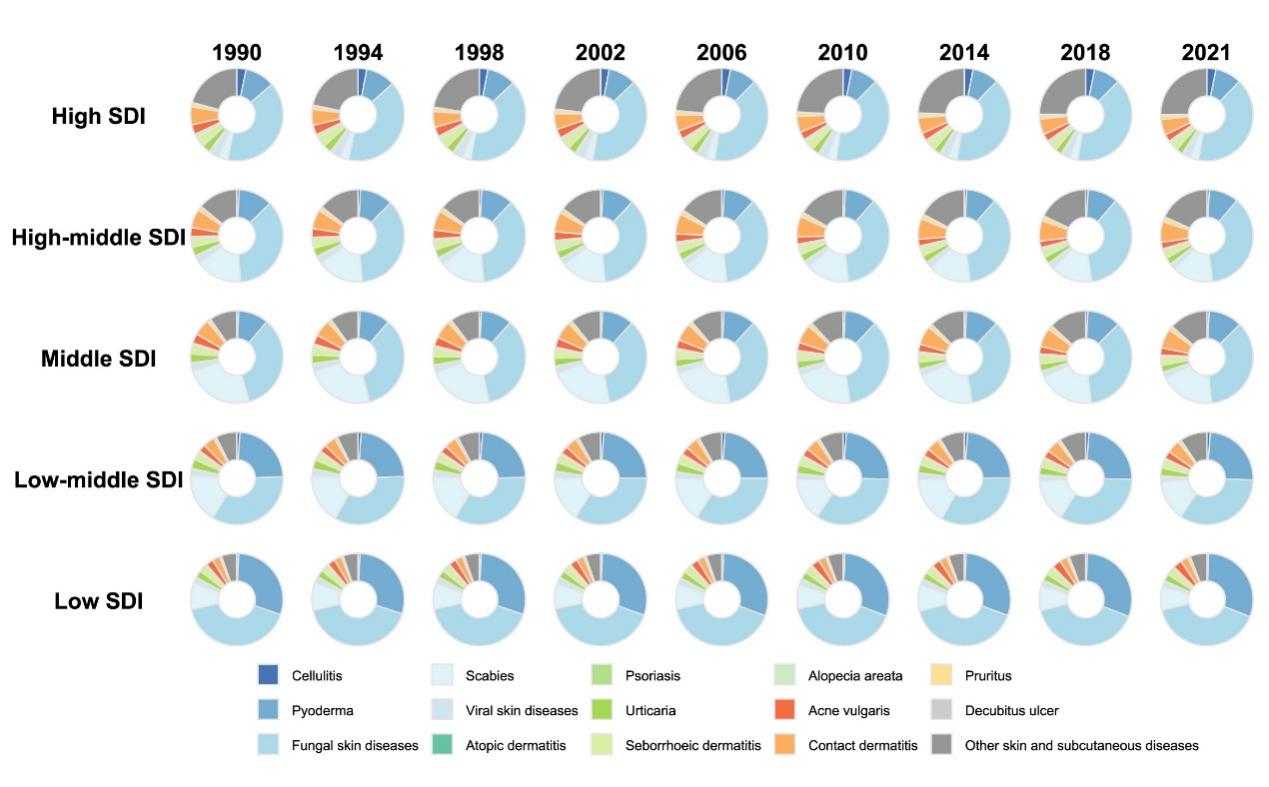


**Supplementary Figure S8.** SDI-Stratified Composition of 15 SSDs-related incidence cases, 1990–2021. Abbreviations: SSDs, skin and subcutaneous diseases; SDI, socio-demographic index.


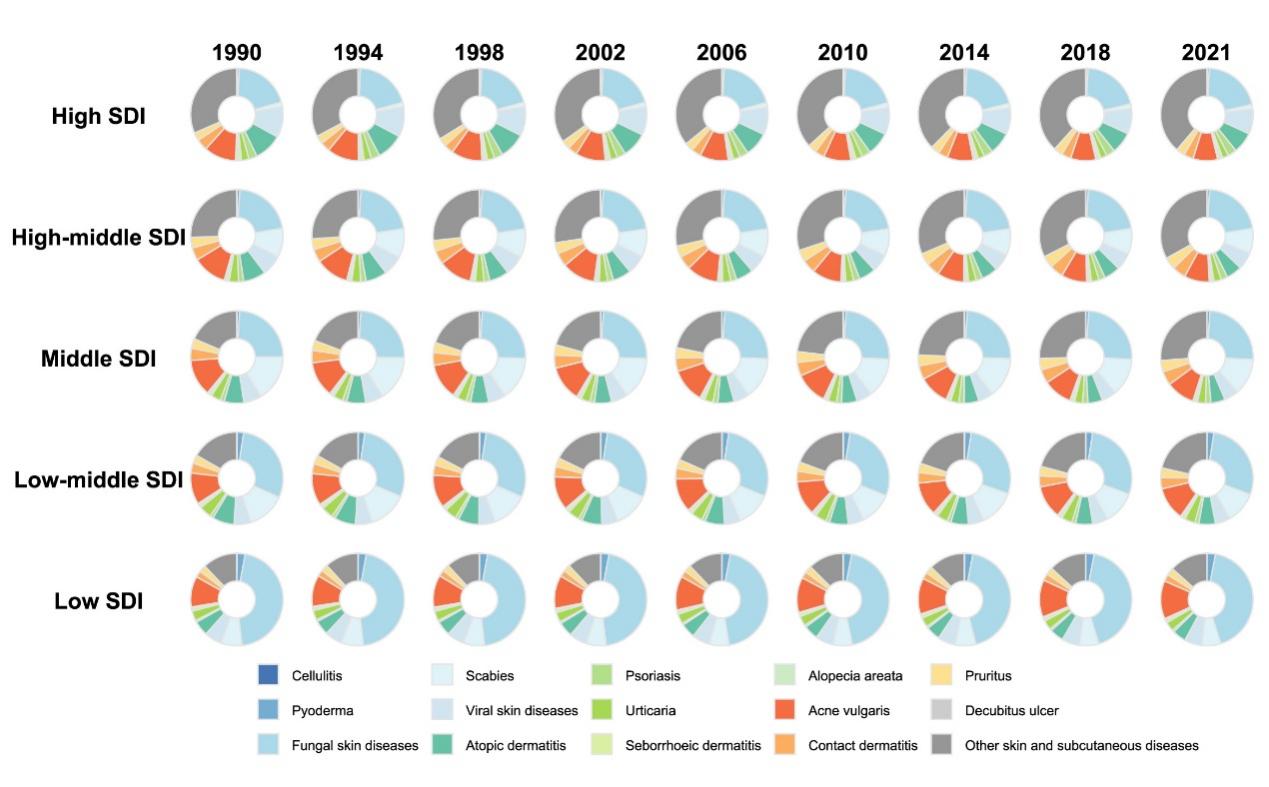


**Supplementary Figure S9.** SDI-Stratified Composition of 15 SSDs-related prevalent cases, 1990–2021. Abbreviations: SSDs, skin and subcutaneous diseases; SDI, socio-demographic index.


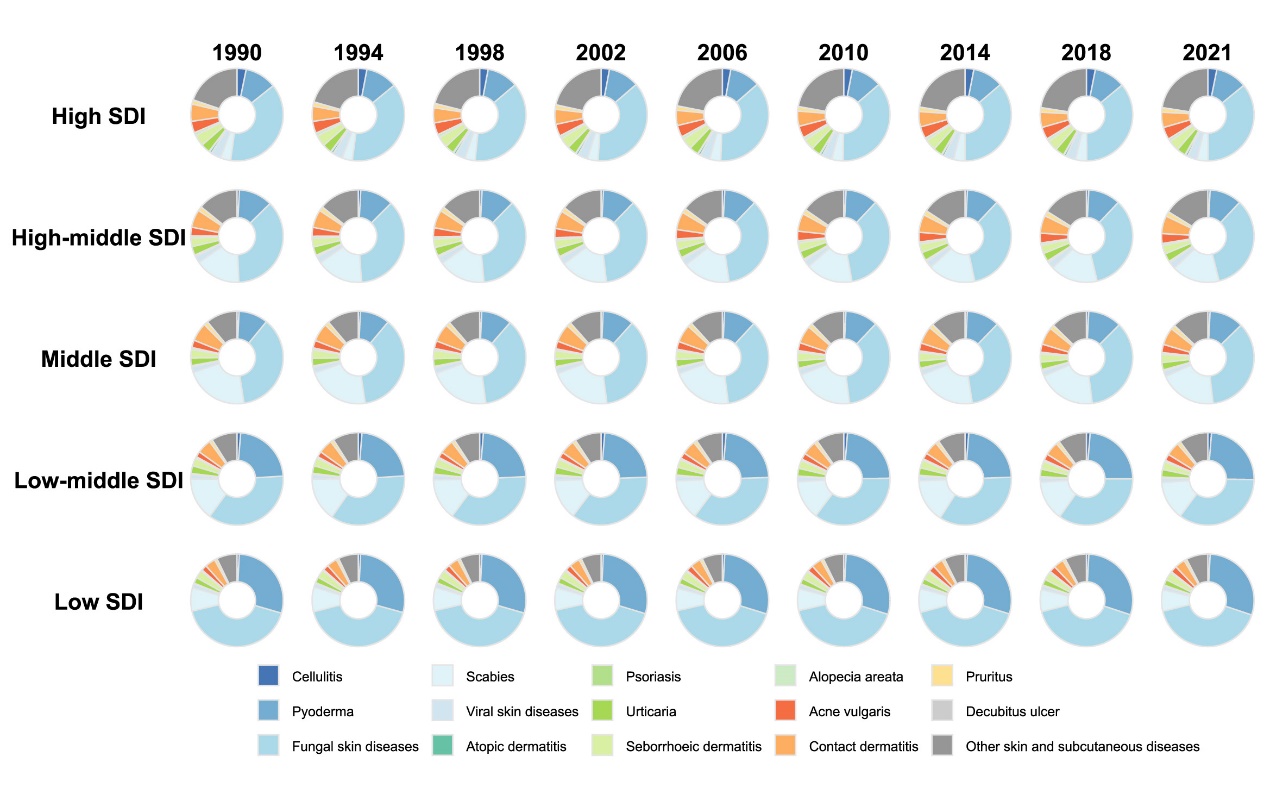


**Supplementary Figure S10.** SDI-Stratified Composition of 15 SSDs-related ASIR, 1990–2021. Abbreviations: SSDs, skin and subcutaneous diseases; SDI, socio-demographic index; ASIR, age-standardized incidence rate.


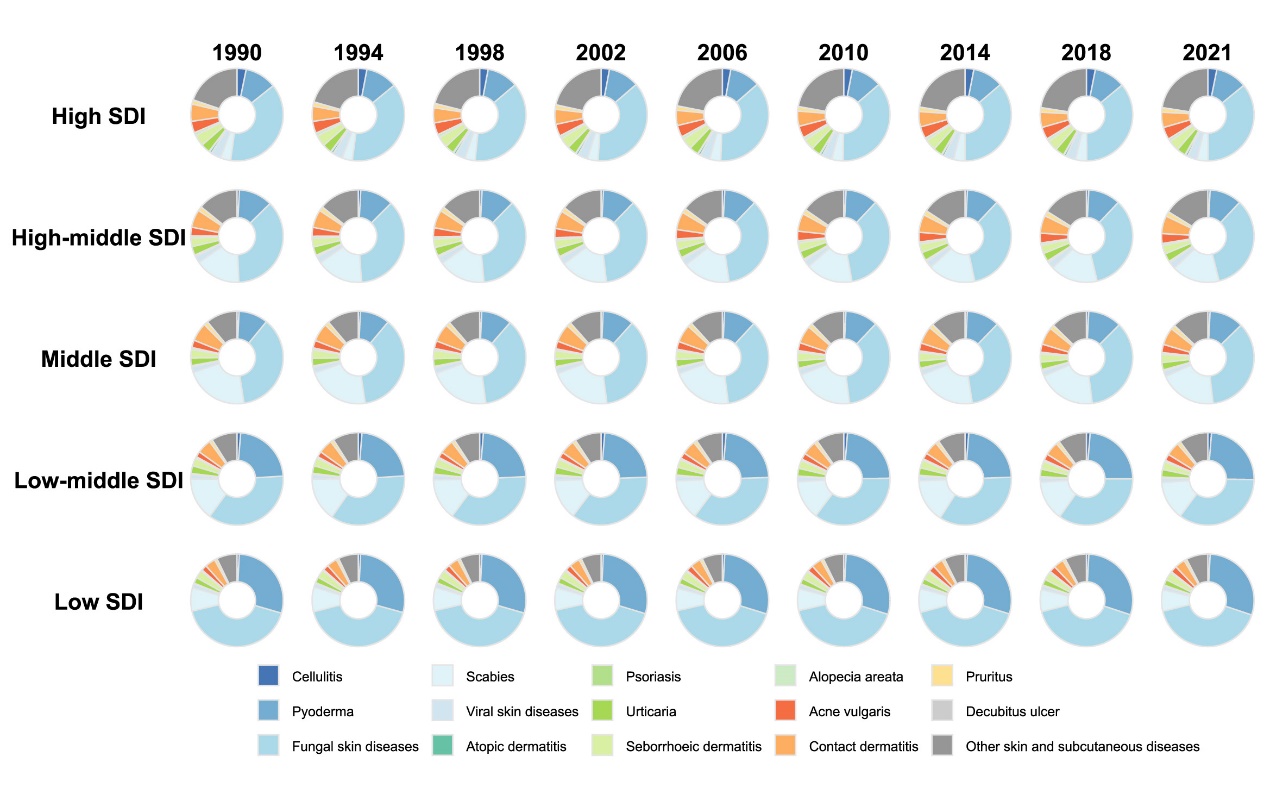


**Supplementary Figure S11.** SDI-Stratified Composition of 15 SSDs-related ASPR, 1990–2021. Abbreviations: SSDs, skin and subcutaneous diseases; SDI, socio-demographic index; ASPR, age-standardized prevalence rate.


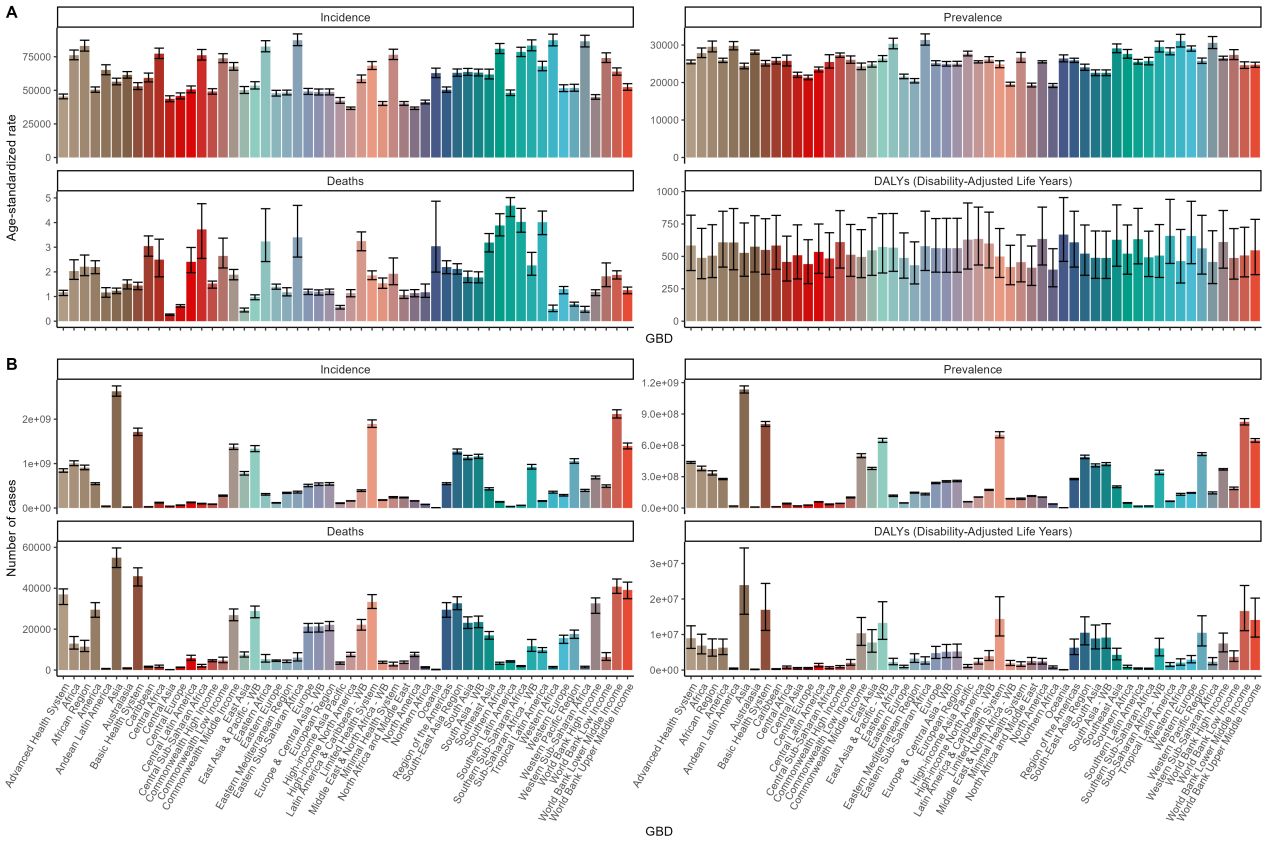
**Supplementary** Figure S12. GBD region-specific analysis of SSDs burden in 2021. Age-standardized rates (A) and number of cases (B) of incidence, prevalence, deaths, and DALYs across different GBD regions. Abbreviations: DALYs, disability-adjusted life years; GBD, global burden of disease; SSDs, skin and subcutaneous diseases.


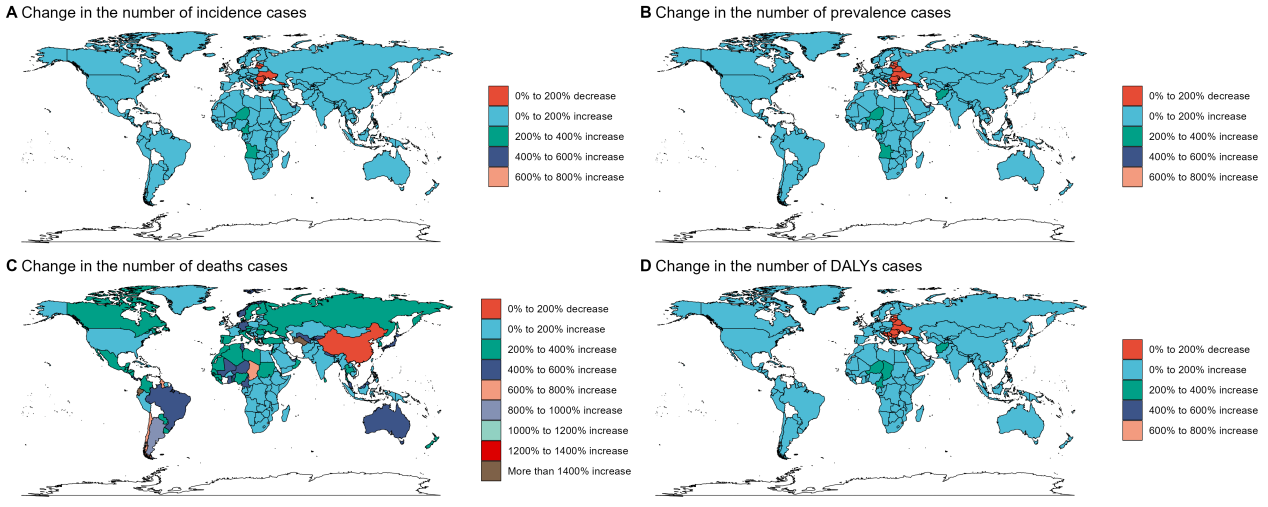


**Supplementary** Figure S13. Changes in SSDs-related number of (A) incidence, (B) prevalence, (C) deaths and (D)DALYs cases across countries and territories from 1990 to 2021. Abbreviations: DALYs, disability-adjusted life years; SSDs, skin and subcutaneous diseases.


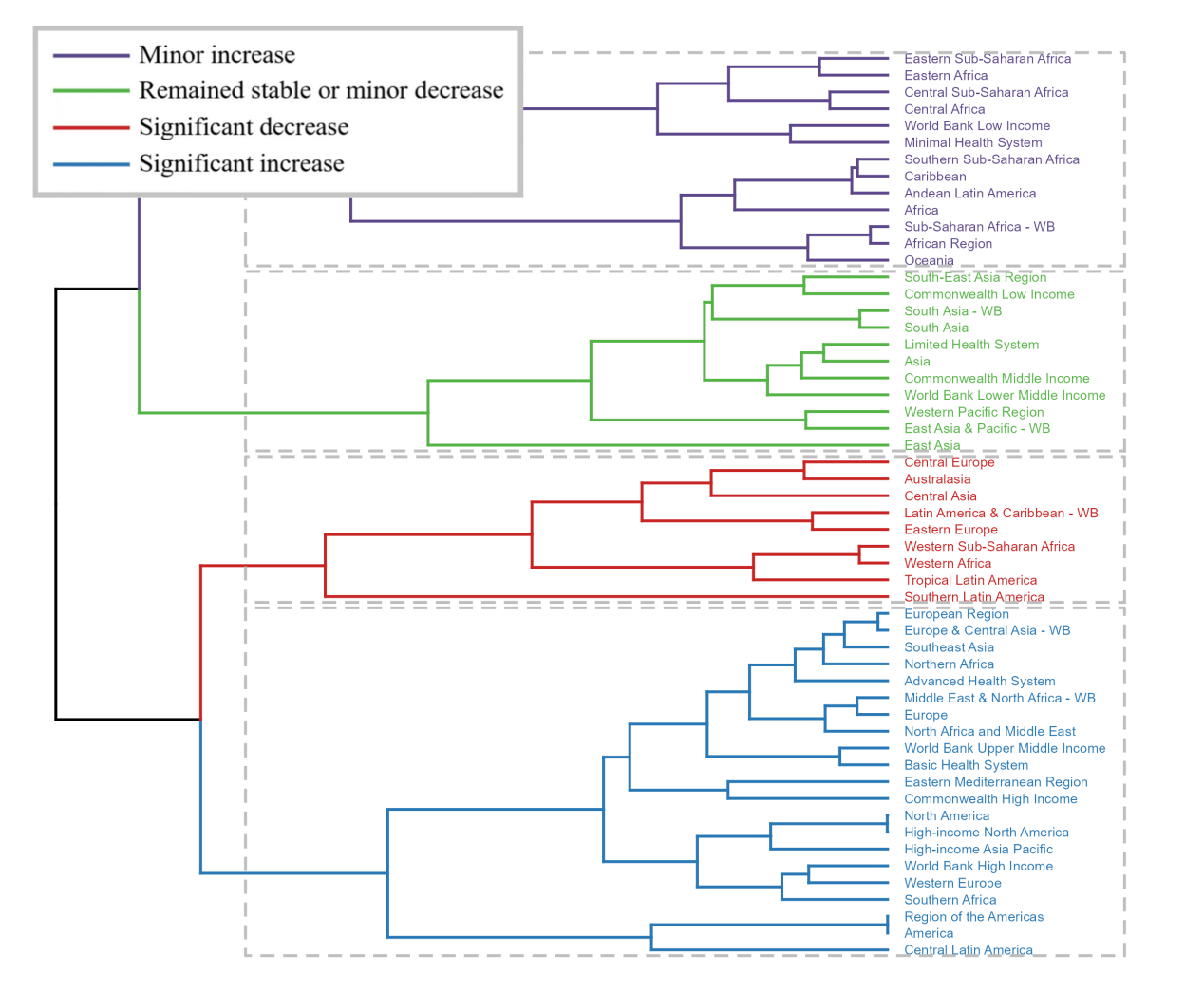


**Supplementary** Figure S14. The outcomes of cluster analysis, on account of the EAPC values of the age-standardized rates of SSDs from 1990 to 2021. Trend patterns categorize regions: significant increase (blue), minor increase (purple), stable or minor decrease (green), and considerable decrease (red). Abbreviations: EAPC, estimated annual percentage change; SSDs, skin and subcutaneous disease.


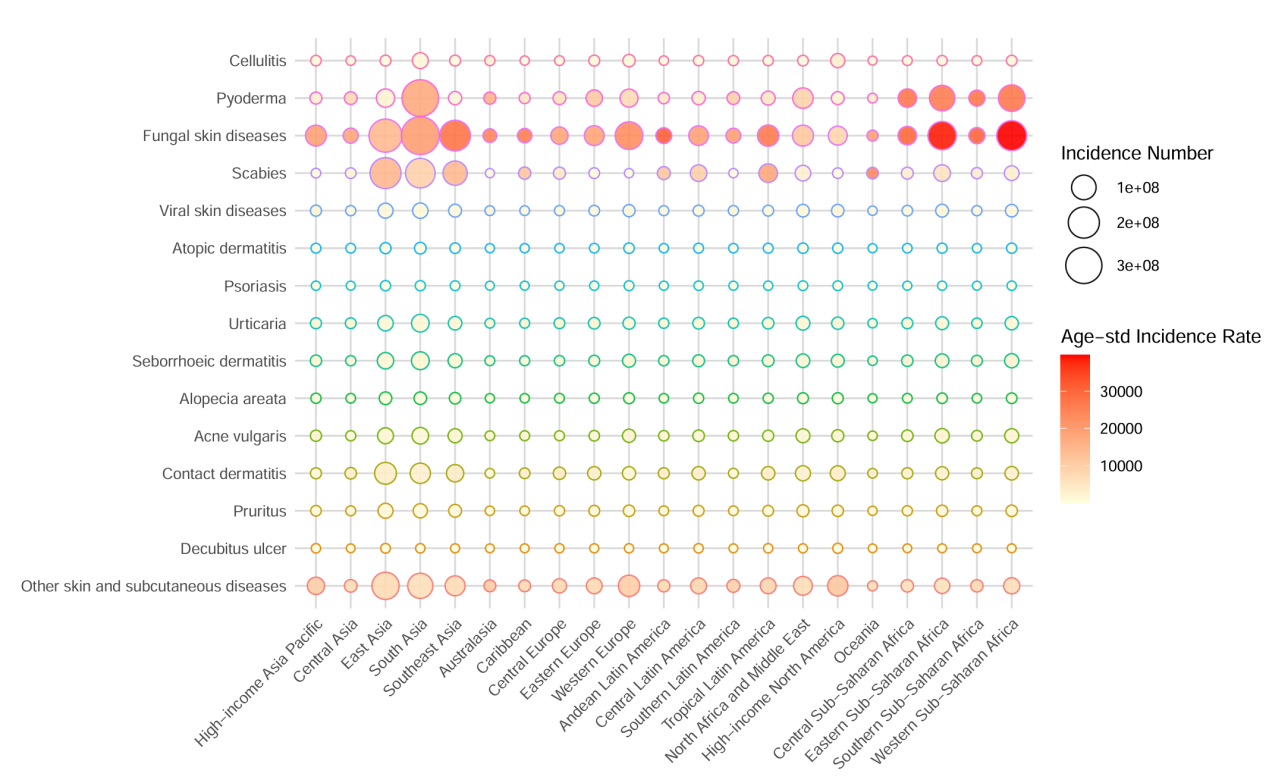


**Supplementary Figure S15.** Geographic Distribution of 2021 incidence for 15 SSDs. Abbreviations: SSDs, skin and subcutaneous diseases.


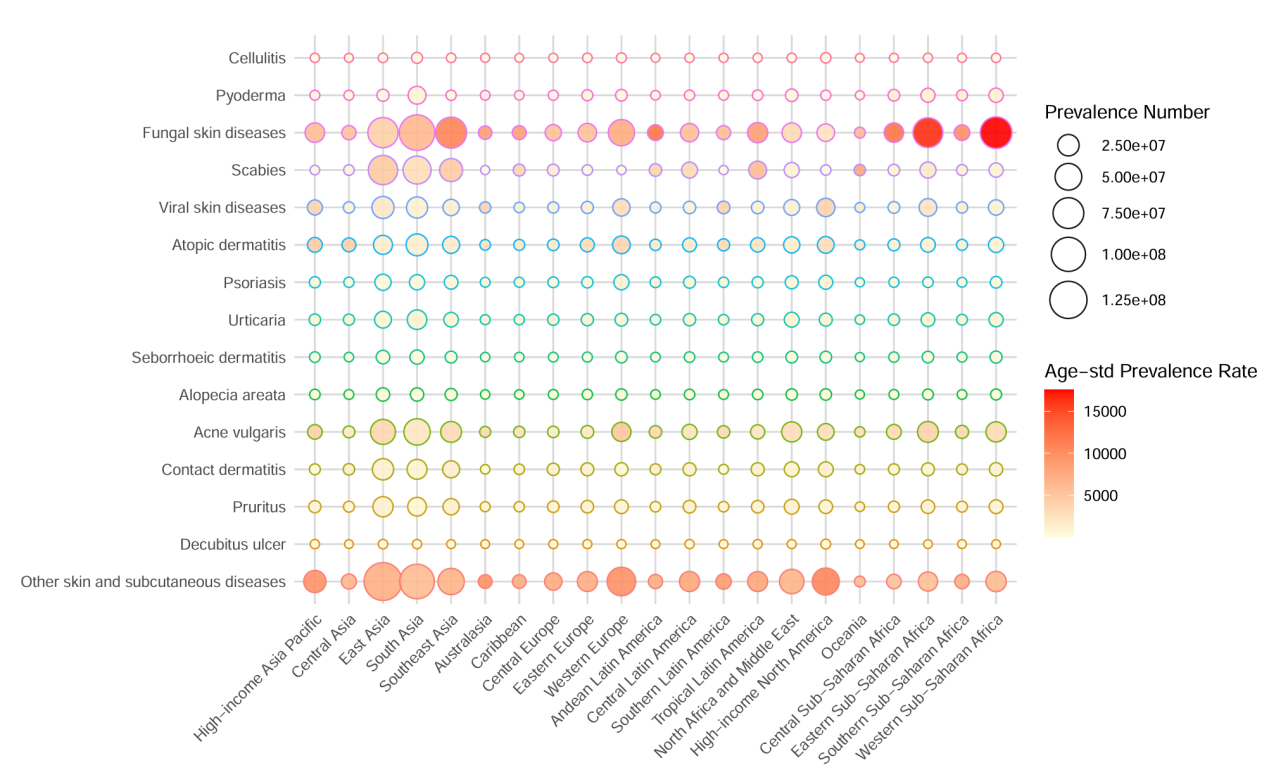


**Supplementary Figure S16.** Geographic Distribution of 2021 prevalence for 15 SSDs. Abbreviations: SSDs, skin and subcutaneous diseases.


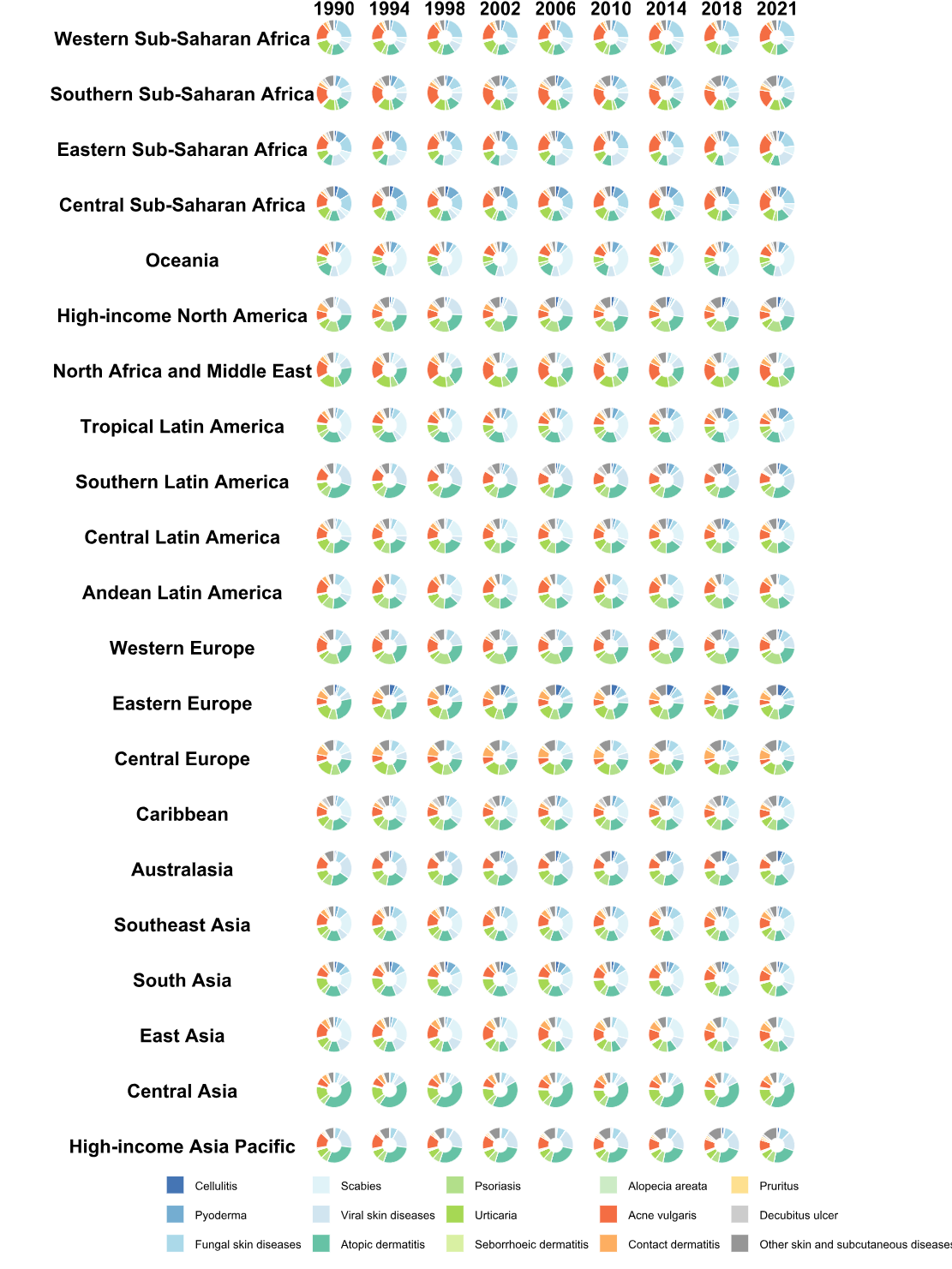


**Supplementary Figure S17.** 21 geographical regions-Stratified Composition of 15 SSDs-related DALYs cases, 1990–2021. Abbreviations: SSDs, skin and subcutaneous diseases; DALY, disability-adjusted life years.


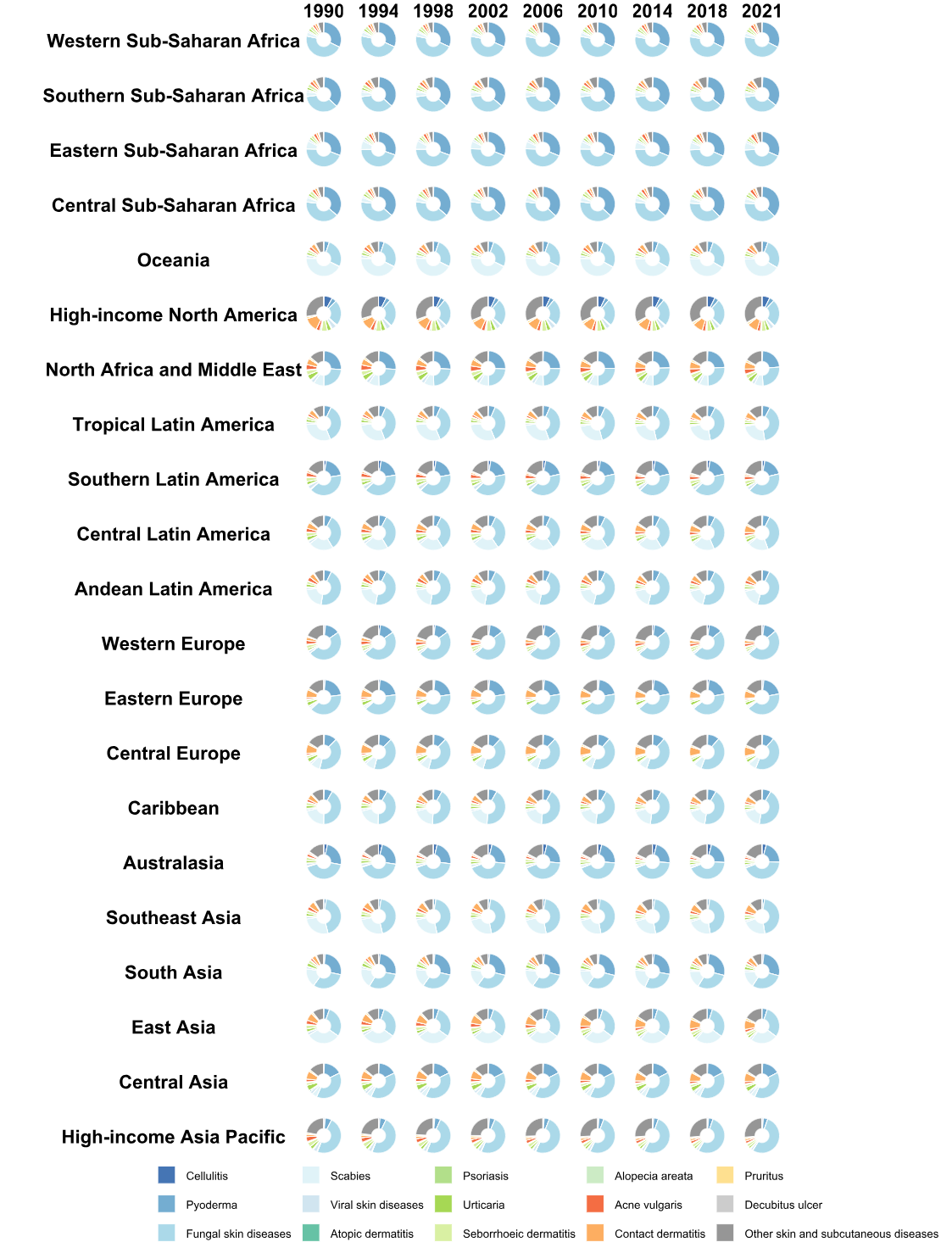


**Supplementary Figure S18.** 21 geographical regions-Stratified Composition of 15 SSDs-related incidence cases, 1990–2021. Abbreviations: SSDs, skin and subcutaneous diseases.


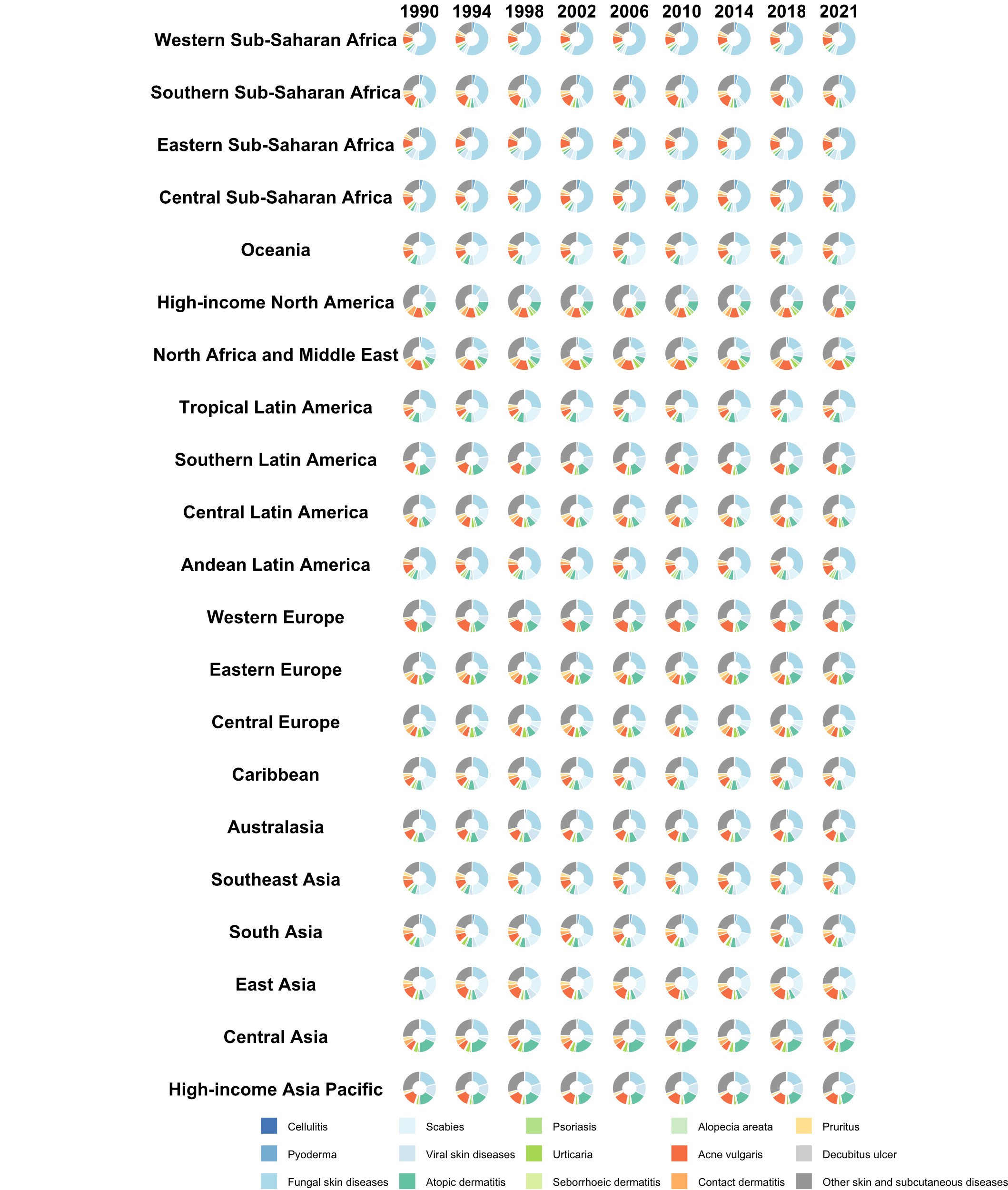


**Supplementary Figure S19.** 21 geographical regions-Stratified Composition of 15 SSDs-related ASPR, 1990–2021. Abbreviations: SSDs, skin and subcutaneous diseases; ASPR, age-standardized prevalence rate.


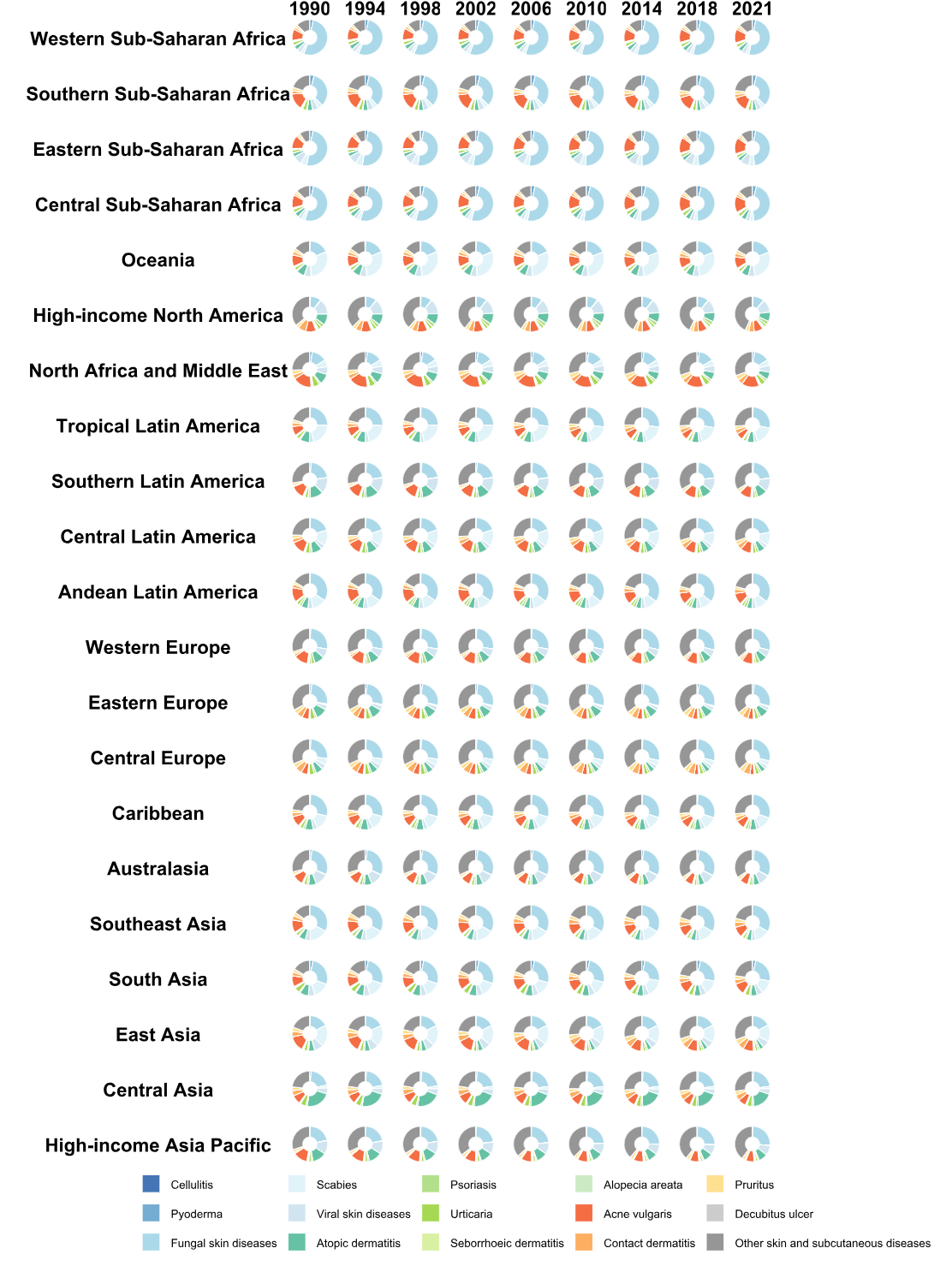


**Supplementary Figure S20.** 21 geographical regions-Stratified Composition of 15 SSDs-related prevalent cases, 1990–2021. Abbreviations: SSDs, skin and subcutaneous diseases.
