# Supplementary material for: Global, regional and national burden of skin and subcutaneous diseases: a systematic analysis of the Global Burden of Disease Study 2021
Source: Int Health. 2025 Jun 28;18(2):183–96. doi: 10.1093/inthealth/ihaf070 (PMC13017215; doi:10.1093/inthealth/ihaf070)
Supplement: ihaf070_Supplemental_Files [file ihaf070_supplemental_files.zip › Supplementary Information (A).docx]

**Supplementary Information(A)**

*1. Atopic Dermatitis*

1) Case Definition & Diagnostic Criteria

GBD defines atopic dermatitis (AD) as a relapsing inflammatory skin disorder characterized by epidermal barrier dysfunction, presenting with erythema, scaling or lichenification (ICD-10 L20). Diagnosis in GBD relies on clinical examination of typical eczema lesions, often accompanied by elevated IgE and other immunologic markers.

2) Input Data Sources

Data were drawn from epidemiological literature and insurance–claims datasets. GBD 2010 systematically reviewed publications (1980–2012) on AD incidence/prevalence; subsequent updates covered 2013–2016. Key non–survey inputs included US commercial insurance claims (MarketScan, 2000 & 2010–2016) and registry data from Poland and Russia. Hospital admission records and self-reported survey data (e.g., NHANES, NHIS) without clinical exam confirmation were excluded. In all, 113 countries contributed AD data.

3) Modeling Tool & Strategy

We used DisMod-MR 2.1 to estimate age–sex–year–location–specific prevalence. Priors assumed no excess mortality and remission rates ranging from 0.0 to 0.2 (implying disease durations from ~5 years to lifelong). A 20-year time window was applied. Beginning in GBD 2019, MR-BRT replaced DisMod’s internal crosswalks to adjust non–clinical survey data to a clinical–examination reference definition. Socio-demographic index (SDI) was included as a covariate.

4) Data Processing & Bias Adjustment

MR-BRT bias corrections aligned non-clinical (“self-report“ or “claims-only“) data to the clinical exam reference standard. For example, US MarketScan 2000 data were up-adjusted by a factor of ~1.8, based on logit bias coefficients. Outlier data points (e.g., implausible age patterns or region-level anomalies) were excluded.

5) Severity Splitting

AD was split into three severity levels with corresponding disability weights (DWs):

Mild: DW 0.027 (skin lesions ± mild itch)

Moderate: DW 0.188 (pronounced lesions, itch interfering with sleep/attention)

Severe: DW 0.576 (widespread, intense itch/pain, psychosocial impact)

Proportions in each category were drawn from clinical studies and expert opinion.

6) Uncertainty Estimation

Uncertainty intervals (UIs) were derived from 1,000 posterior draws of the DisMod model, combined with the DW uncertainty distributions, yielding 95% UIs for prevalence, YLDs, and DALYs.

*2. Contact Dermatitis*

1) Case Definition

An inflammatory skin condition caused by contact with irritants or allergens (ICD-10 L23–L25), diagnosed by lesion location and history, often confirmed by patch testing.

2) Data Sources

Literature surveys (1980–2016) and US MEPS and MarketScan claims (2000–2014). Seventeen countries contributed 69 prevalence data points.

3) Modeling

DisMod-MR 2.1 with priors of no excess mortality, remission rate prior 0.1–4.0 (0.25–10 years), and zero incidence under age 6. A 25-year window ensured adequate data.

4) Bias Adjustment

MR-BRT calibrated non-clinical data to the clinical reference. US claims and survey data were up-adjusted ~2× to match examination‐based case definitions. Region-level random effects were constrained to avoid extreme fluctuations.

5) Severity Splitting

Two strata:

Mild (DW 0.027)

Moderate (DW 0.188)

No “severe“ category was defined, as cases rarely progress beyond moderate disability.

6) Uncertainty

Derived from 1,000 draws sampling prevalence and DW uncertainty, yielding 95% UIs.

3. Seborrheic Dermatitis

1) Definition

Inflammatory disease of sebaceous‐gland–rich areas (ICD-10 L21), manifesting with greasy scales and erythema.

2) Data

Prevalence literature (1980–2017), US/Scandinavian outpatient registries; 23 countries, 73 data points.

3) Modeling

DisMod-MR 2.1 with remission prior 0.1–12 (1 month–10 years), minimal incidence under 5 years & above 60 years, zero excess mortality, 25-year window.

4) Bias Adjustment

MR-BRT aligned administrative and survey data to the reference. Region-specific random effects were relaxed for data-sparse regions.

5) Severity

Single category (DW 0.027); all cases treated equally.

6) Uncertainty

Prevalence uncertainty plus DW uncertainty propagated via 1,000 draws.

4. Psoriasis

1) Definition

Chronic immune‐mediated skin disease (ICD-10 L40), typified by well-demarcated erythematous plaques with silver scales.

2) Data

Systematic reviews (1980–2016), national surveys (MEPS, NHANES, Australia), claims data (US, Taiwan, Poland, Russia); 30 countries, 117 prevalence sources.

3) Modeling

DisMod-MR 2.1 with remission prior 0.05–0.15 (6.6–20 years), no excess mortality, 10-year window. SDI and absolute latitude used as covariates. MR-BRT bias‐corrected claims vs. survey data.

4) Bias Adjustment

Claims and administrative data were down-adjusted ~25–60% to align with clinical exam prevalence estimates. Outliers excluded.

5) Severity Splitting

Three levels:

Mild (DW 0.027)

Moderate (DW 0.188)

Severe (DW 0.576)

Severity proportions drawn from meta‐analyses of clinical cohorts.

6) Uncertainty

1,000‐draw posterior sampling of DisMod estimates and DWs; 95% UIs reported.

*5. Acne Vulgaris*

1) Definition

Chronic inflammatory disease of pilosebaceous units (ICD-10 L70), excluding rosacea (L71).

2) Data

Literature review (1980–2016), claims (MarketScan 2000 & 2010–2016), registries (Poland, Taiwan, Norway), 34 countries, 108 sources.

3) Modeling

DisMod-MR 2.1 with no excess mortality, remission prior 0.38–0.6 (2–3 years), zero incidence under 6 years & above 60 years, 5-year data window. MR-BRT adjusted non‐clinical data; SDI, sugar consumption, HAQ index used as covariates.

4) Bias Adjustment

MR-BRT crosswalks: clinical exam & MarketScan 2010–2014 as reference; “no exam“ data up-adjusted ~1.2×, older MarketScan up-adjusted ~2×, outpatient data up-adjusted ~12×.

5) Severity

Three strata:

Mild (DW 0.011)

Moderate (DW 0.067)

Severe (DW 0.405)

6) Uncertainty

Combined sampling of DisMod outputs, severity proportions, and DWs; 95% UIs from 1,000 draws.

*6. Alopecia Areata*

1) Definition

Autoimmune hair‐follicle disorder causing patchy hair loss (ICD-10 L63).

2) Data

Literature (1980–2016) & MarketScan claims; 16 countries, 41 prevalence points + some incidence data.

3) Modeling

DisMod-MR 2.1 with no excess mortality, remission prior 0.0–0.2 (~7 months minimum), 20-year window. MR-BRT bias‐corrected claims; gender effect constrained to avoid overestimation of female effect.

4) Bias Adjustment

US claims data (2000 & 2010–2016) down-adjusted ~67%; sex ratio bias removed by fixing sex coefficient to zero.

5) Severity

Two levels:

Mild (DW 0.011)

Severe (DW 0.067)

~90% of cases assumed mild; remaining severe.

6) Uncertainty

Prevalence + DW sampling via 1,000 draws; 95% UIs reflect data and DW uncertainty.

*7. Scabies*

1) Definition

Infestation by Sarcoptes scabiei mites (ICD-10 B86), causing intense itch and papular rash.

2) Data

Literature (1980–2013), MarketScan claims (2000 & 2010–2016), outpatient data; 36 countries, 147 prevalence + 5 incidence points.

3) Modeling

DisMod-MR 2.1 with no excess mortality, remission prior 2.5–3.5 (~4–5 months), 5-year window. MR-BRT adjusted survey vs. clinical data; region random effects constrained ±0.25–0.5.

4) Bias Adjustment

US 2000 claims down-adjusted ~23%; self-report lightly adjusted (~4% difference). Region constraints applied to limit extreme outliers.

5) Severity

Single acute‐infection level (DW 0.027).

6) Uncertainty

Prevalence sampling + DW uncertainty via 1,000 draws; 95% UIs reported.

*8. Fungal Skin Diseases*

1) Definition

Superficial dermatophytoses (tinea capitis, corporis, pedis, etc.; ICD-10 B35–B36). Tinea capitis modeled separately.

2) Data

Literature (1980–2017), MEPS (2000–2009), claims (MarketScan 2000 & 2010–2016); 31 countries, 137 prevalence points.

3) Modeling

Two DisMod models: tinea capitis (remission 0.5–4; age cutoff >20 years low incidence) & other fungal diseases (remission 0.33–4; no excess mortality), both 20-year windows, MR-BRT bias correction, SDI & HAQ covariates, region effect constraints.

4) Bias Adjustment

Outpatient data down-adjusted ~90% for tinea capitis; MEPS and early claims down-adjusted ~50% for other fungal.

5) Severity

Single acute‐infection level (DW 0.006).

6) Uncertainty

Prevalence + DW sampling; wide 95% UIs reflecting data heterogeneity.

*9. Viral Skin Diseases*

1) Definition

Viral warts (HPV) & molluscum contagiosum (ICD-10 B07 & B08.1), modeled separately.

2) Data

Sparse literature; US outpatient & MarketScan (2000 & 2010–2016); 35 countries, 61 prevalence + 7 incidence points.

3) Modeling

Two DisMod models: warts (remission 0.25–2; incidence cap ~0.1%/year) & molluscum (remission 0.5–2), 25-year windows, MR-BRT bias corrections (~60–70% down-adjustment of claims), region effect constraints.

4) Bias Adjustment

MarketScan 2000 & 2010–2016 both down-adjusted ~68–69% to match clinical references.

5) Severity

Two levels:

Mild acute infection (DW 0.006)

Moderate disfigurement (DW 0.067) for ~10% of wart cases; molluscum all mild.

6) Uncertainty

1,000 draws sampling prevalence, severity proportions, and DWs; 95% UIs reflect combined uncertainty.

*10. Pruritus*

1) Definition

Primary chronic itch disorder without clear skin lesions (ICD-10 L29).

2) Data

Very limited—some symptom surveys and outpatient records. GBD likely extrapolates from skin disease surveys.

3) Modeling

DisMod-MR 2.1 with no mortality, chronic course prior, using analogous diseases (e.g., dermatitis) to inform covariates. High uncertainty.

4) Bias Adjustment

Self-report vs. clinical data adjusted via MR-BRT; outpatient data up-adjusted for unreported mild cases; comorbidity corrections applied to avoid double counting with eczema.

5) Severity

Assumed moderate acute‐infection DW (~0.027–0.051), no further splits.

6) Uncertainty

Wide UIs from limited data and DW assumptions; sampled via 1,000 draws.

*11. Urticaria*

1) Definition

Transient wheals from skin mast-cell degranulation (ICD-10 L50), including chronic spontaneous and inducible subtypes.

2) Data

Sparse survey and claims data; global burden internally estimated (GBD 2019: ~65.1 million cases).

3) Modeling

DisMod-MR 2.1 with no mortality, high remission rate to reflect episodic nature, incidence from survey recall (“ever had urticaria“) converted to point prevalence.

4) Bias Adjustment

Survey recall vs. clinical case definitions crosswalked by MR-BRT; outpatient vs. self-report adjusted to a common reference scale.

5) Severity

Single moderate‐severity DW (~0.050–0.067), reflecting impact of itch and transient impairment.

6) Uncertainty

Limited inputs → wide 95% UIs from 1,000 posterior draws of prevalence and DW.

*12. Decubitus Ulcer*

1) Definition

Pressure injury from prolonged compression (ICD-10 L89), staged I–IV.

2) Data

Hospital admission, long-term care facility reports, outpatient follow-up, and death registry data.

3) Modeling

DisMod-MR 2.1 for incidence/prevalence; CODEm for mortality. Age, HAQ, and care‐facility covariates used; excess mortality reconciled via DisMod’s EMR.

4) Bias Adjustment

MR-BRT corrects underreporting in outpatient settings; duplicate admissions and coding inconsistencies adjusted. CODEm ensemble uses care capacity covariates to model mortality.

5) Severity

Three categories:

Stage I–II (DW 0.006)

Stage III (DW 0.051)

Stage IV (DW 0.133)

6) Uncertainty

Combined YLD (DisMod) and YLL (CODEm) sampling—1,000 draws for incidence, severity DWs, and mortality—to derive DALY UIs.

*13. Cellulitis*

(Modeled under “Bacterial skin diseases“ – parameters parallel to pyoderma but with distinct ICD codes and epidemiology.)

*14. Pyoderma*

1) Definition

Purulent bacterial skin infections (e.g., impetigo, abscesses; ICD-10 L08 family).

2) Data

High incidence in children reported in tropical‐region surveys; hospital and outpatient records. GBD 2020 reports >10^9 incident cases.

3) Modeling

DisMod-MR 2.1 modeling of incidence with rapid remission (days–weeks), zero excess mortality; pediatric age focus; interactions with cellulitis/scabies covariates.

4) Bias Adjustment

Crosswalks to align community survey vs. hospital‐only data; transform point prevalence to incidence via assumed average disease duration.

5) Severity

Acute mild infection DW ∼0.027. No further splits.

6) Uncertainty

1,000‐draw sampling of incidence priors, remission priors, and DW uncertainty.

*15. Other Skin & Subcutaneous Diseases*
