# Supplementary material for: Global, regional and national burden of skin and subcutaneous diseases: a systematic analysis of the Global Burden of Disease Study 2021
Source: Int Health. 2025 Jun 28;18(2):183–96. doi: 10.1093/inthealth/ihaf070 (PMC13017215; doi:10.1093/inthealth/ihaf070)
Supplement: ihaf070_Supplemental_Files [file ihaf070_supplemental_files.zip › Supplementary Information (B).docx]

**Supplementary Information (B)**

Burden of 15 Skin and Subcutaneous Diseases (1990–2021)


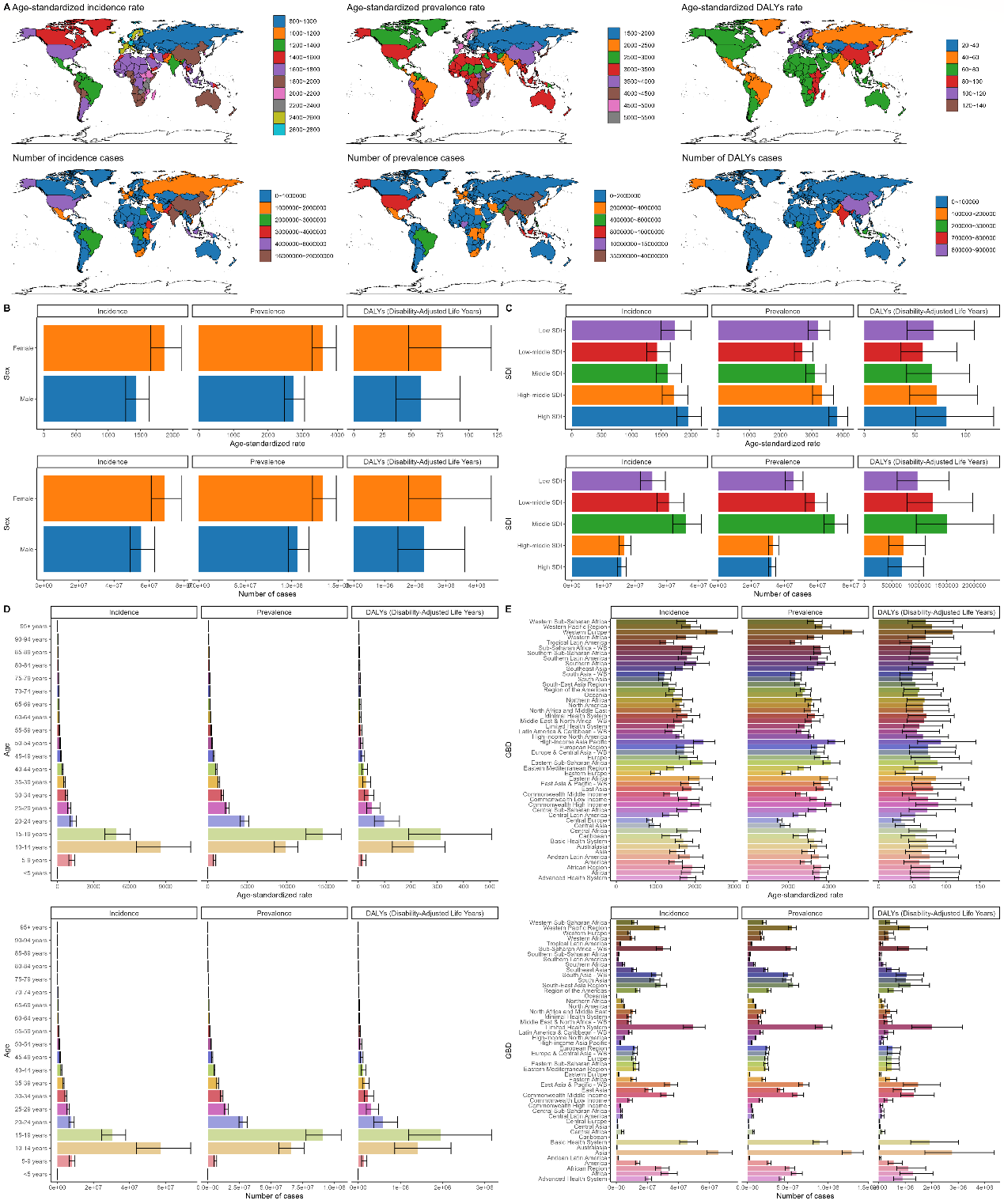


**Supplementary** Figure S21. Global distribution and trends in Acne vulgaris burden, illustrating the differences in incidence, prevalence, DALYs, and number of cases across regions and demographics.

(A) Global distribution of ASRs for SSD incidence, prevalence, and DALYs. (B) Bar plots comparing Acne vulgaris incidence, prevalence, and DALYs by sex and age group. (C) Bar plots showing Acne vulgaris incidence, prevalence, and DALYs by SDI level. (D) Age-specific distribution of Acne vulgaris burden. (E) Stratified analysis of Acne vulgaris burden by GBD regions. Abbreviations: SSDs, Skin and subcutaneous diseases; DALYs: Disability-adjusted life years; ASRs, Age-standardized rates; SDI, Socio-demographic index; GBD, Global Burden of Disease.


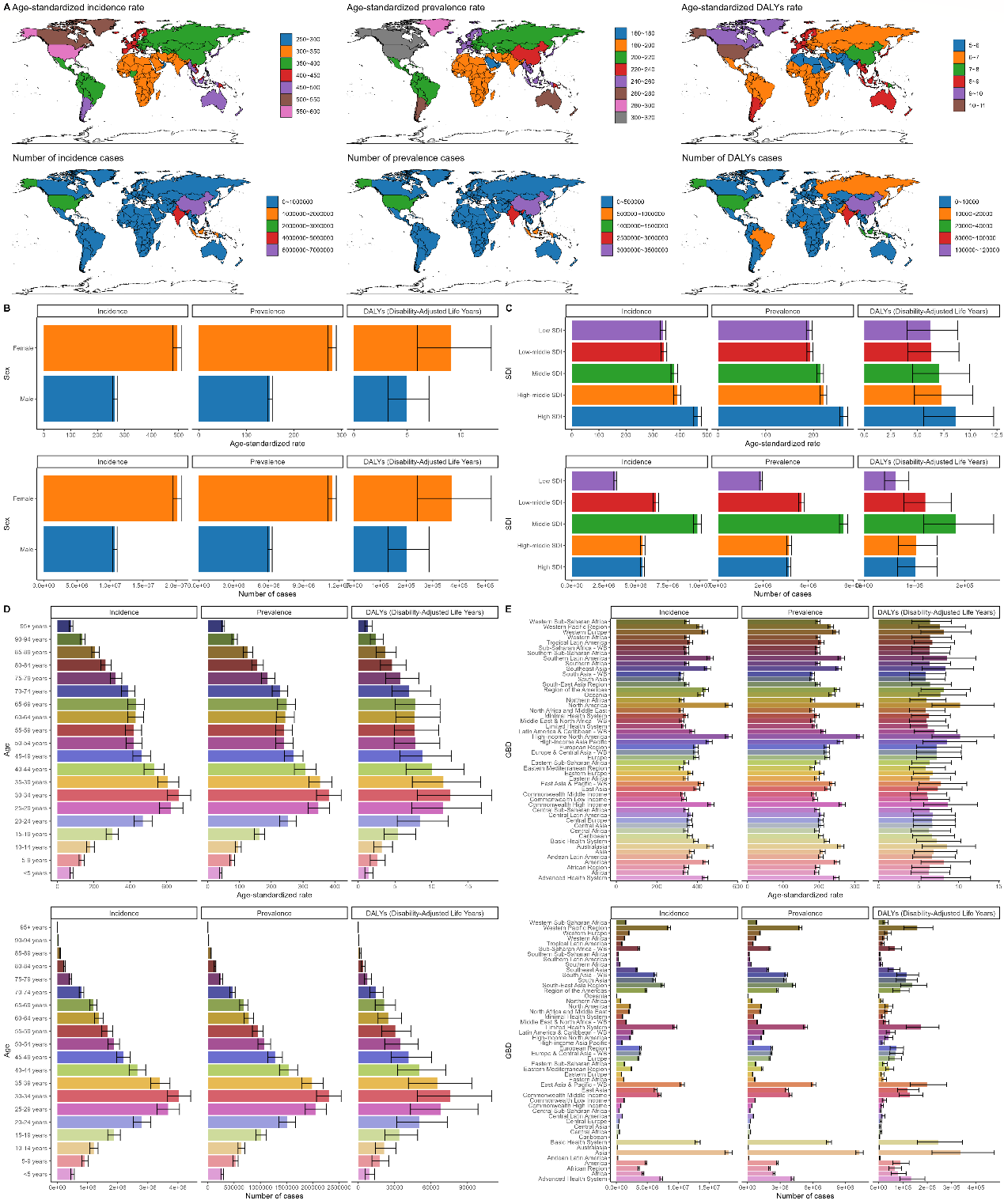


**Supplementary** Figure S22. Global distribution and trends in Alopecia areata burden, illustrating the differences in incidence, prevalence, DALYs, and number of cases across regions and demographics.

(A) Global distribution of ASRs for SSD incidence, prevalence, and DALYs. (B) Bar plots comparing Alopecia areata incidence, prevalence, and DALYs by sex and age group. (C) Bar plots showing Alopecia areata incidence, prevalence, and DALYs by SDI level. (D) Age-specific distribution of Alopecia areata burden. (E) Stratified analysis of Alopecia areata burden by GBD regions. Abbreviations: SSDs, Skin and subcutaneous diseases; DALYs: Disability-adjusted life years; ASRs, Age-standardized rates; SDI, Socio-demographic index; GBD, Global Burden of Disease.


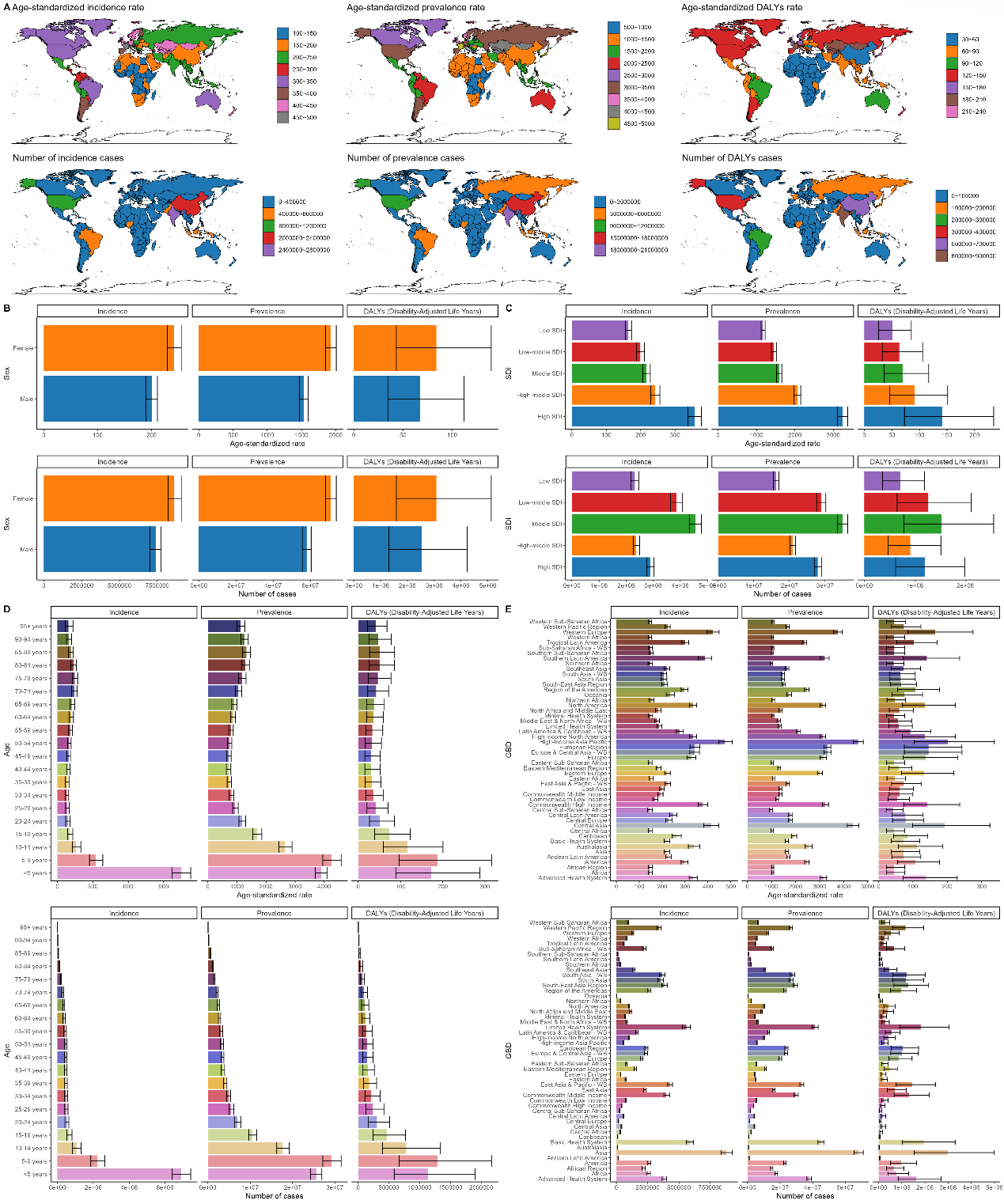


**Supplementary** Figure S23. Global distribution and trends in Atopic dermatitis burden, illustrating the differences in incidence, prevalence, DALYs, and number of cases across regions and demographics.

(A) Global distribution of ASRs for SSD incidence, prevalence, and DALYs. (B) Bar plots comparing Atopic dermatitis incidence, prevalence, and DALYs by sex and age group. (C) Bar plots showing Atopic dermatitis incidence, prevalence, and DALYs by SDI level. (D) Age-specific distribution of Atopic dermatitis burden. (E) Stratified analysis of Atopic dermatitis burden by GBD regions. Abbreviations: SSDs, Skin and subcutaneous diseases; DALYs: Disability-adjusted life years; ASRs, Age-standardized rates; SDI, Socio-demographic index; GBD, Global Burden of Disease.


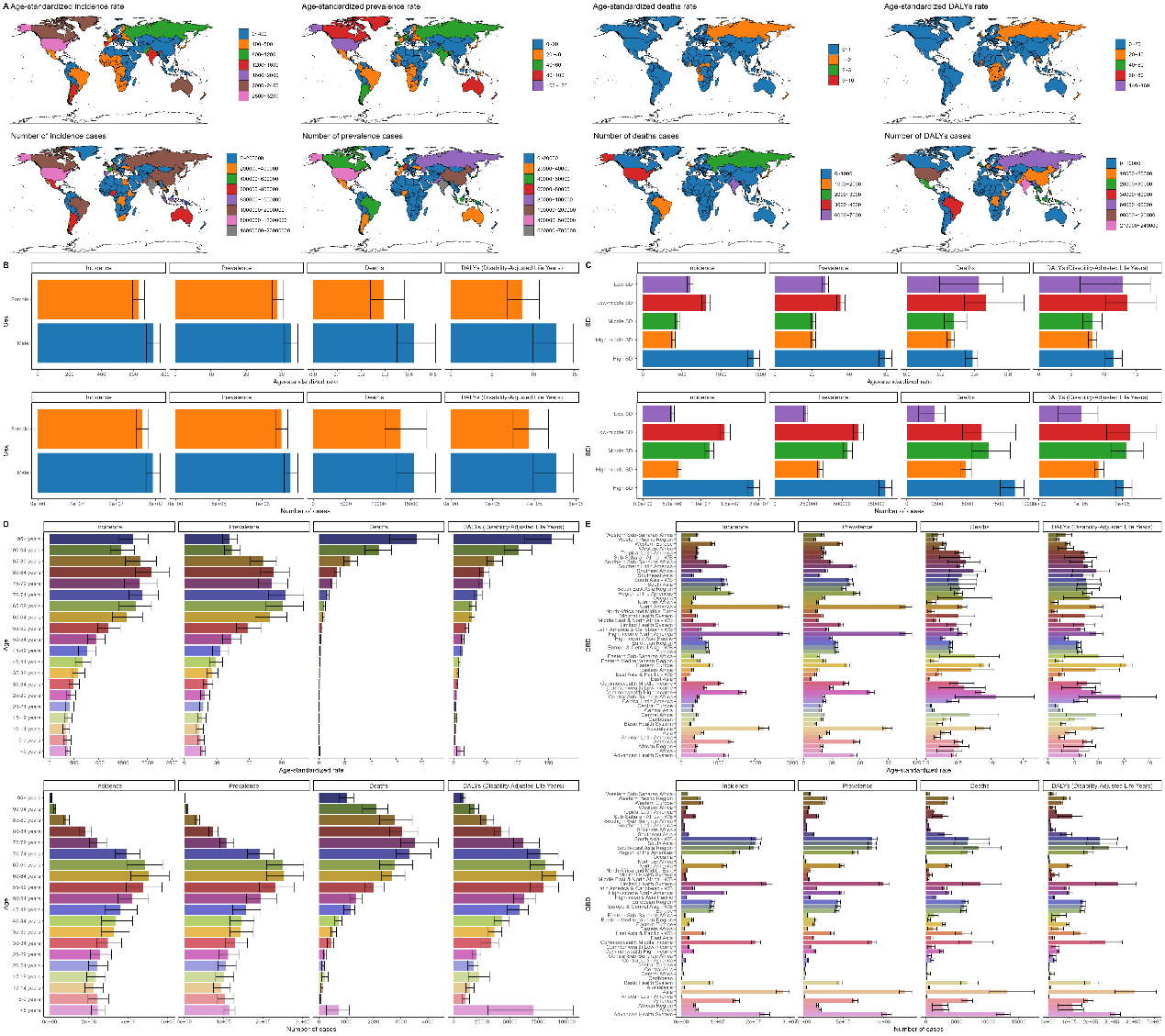


**Supplementary** Figure S24. Global distribution and trends in Cellulitis burden, illustrating the differences in incidence, prevalence, mortality and DALYs, and number of cases across regions and demographics.

(A) Global distribution of ASRs for SSD incidence, prevalence, mortality and DALYs. (B) Bar plots comparing Cellulitis incidence, prevalence, mortality and DALYs by sex and age group. (C) Bar plots showing Cellulitis incidence, prevalence, mortality and DALYs by SDI level. (D) Age-specific distribution of Cellulitis burden. (E) Stratified analysis of Cellulitis burden by GBD regions. Abbreviations: SSDs, Skin and subcutaneous diseases; DALYs: Disability-adjusted life years; ASRs, Age-standardized rates; SDI, Socio-demographic index; GBD, Global Burden of Disease.


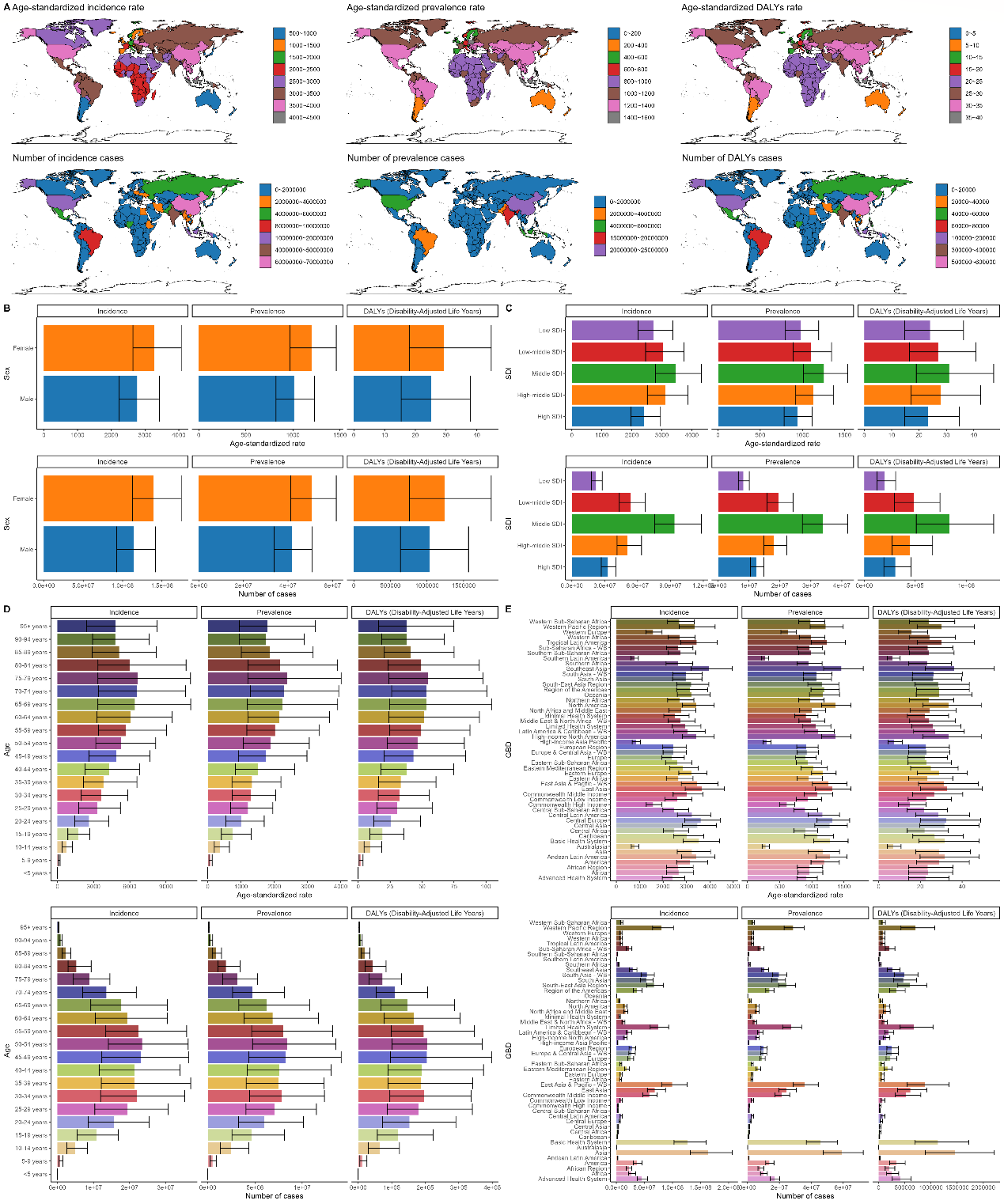


**Supplementary** Figure S25. Global distribution and trends in Contact dermatitis burden, illustrating the differences in incidence, prevalence, DALYs, and number of cases across regions and demographics.

(A) Global distribution of ASRs for SSD incidence, prevalence, and DALYs. (B) Bar plots comparing Contact dermatitis incidence, prevalence, and DALYs by sex and age group. (C) Bar plots showing Contact dermatitis incidence, prevalence, and DALYs by SDI level. (D) Age-specific distribution of Contact dermatitis burden. (E) Stratified analysis of Contact dermatitis burden by GBD regions. Abbreviations: SSDs, Skin and subcutaneous diseases; DALYs: Disability-adjusted life years; ASRs, Age-standardized rates; SDI, Socio-demographic index; GBD, Global Burden of Disease.


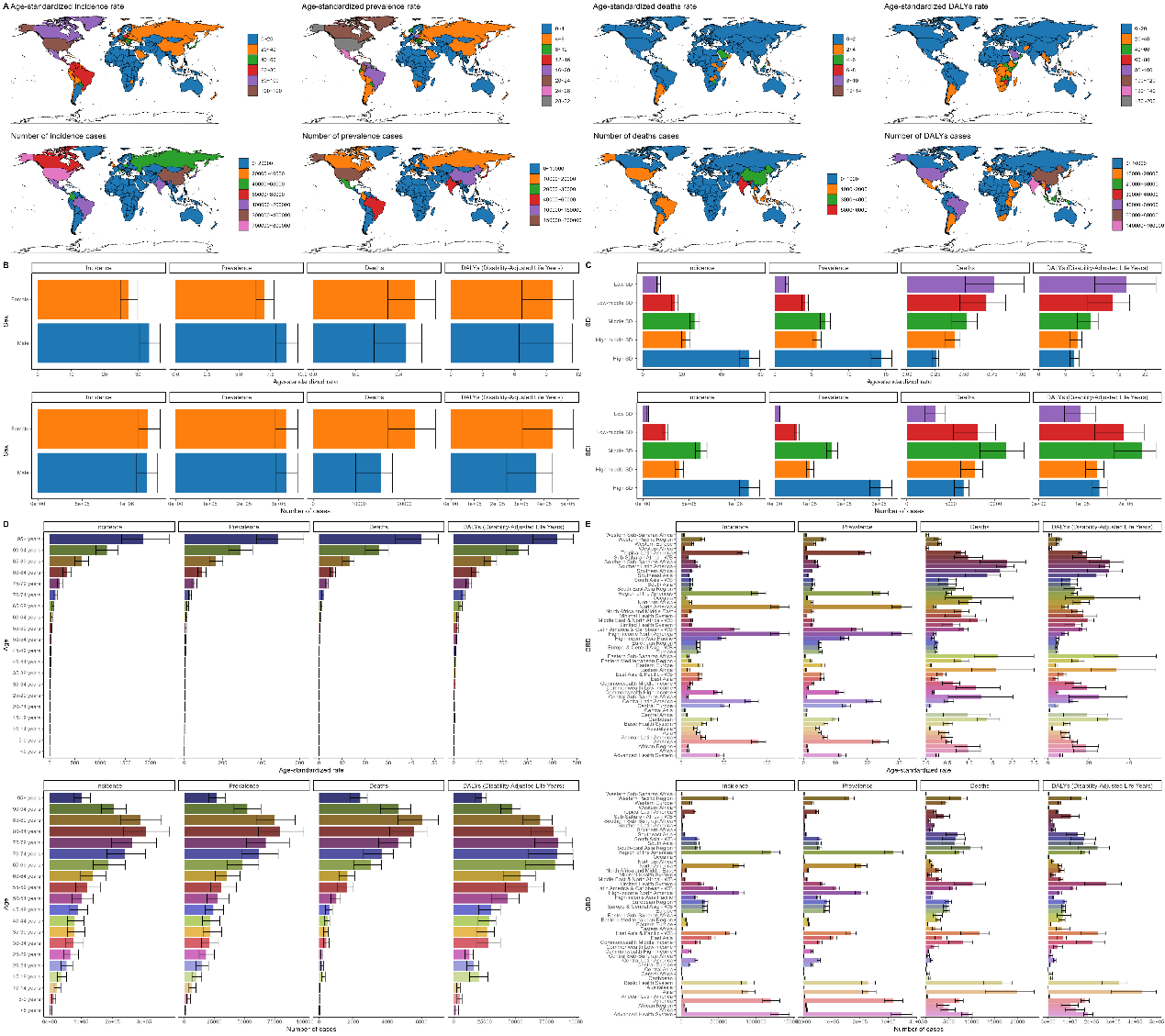


**Supplementary** Figure S26. Global distribution and trends in Decubitus ulcer burden, illustrating the differences in incidence, prevalence, mortality and DALYs, and number of cases across regions and demographics.

(A) Global distribution of ASRs for SSD incidence, prevalence, mortality and DALYs. (B) Bar plots comparing Decubitus ulcer incidence, prevalence, mortality and DALYs by sex and age group. (C) Bar plots showing Decubitus ulcer incidence, prevalence, mortality and DALYs by SDI level. (D) Age-specific distribution of Decubitus ulcer burden. (E) Stratified analysis of Decubitus ulcer burden by GBD regions. Abbreviations: SSDs, Skin and subcutaneous diseases; DALYs: Disability-adjusted life years; ASRs, Age-standardized rates; SDI, Socio-demographic index; GBD, Global Burden of Disease.


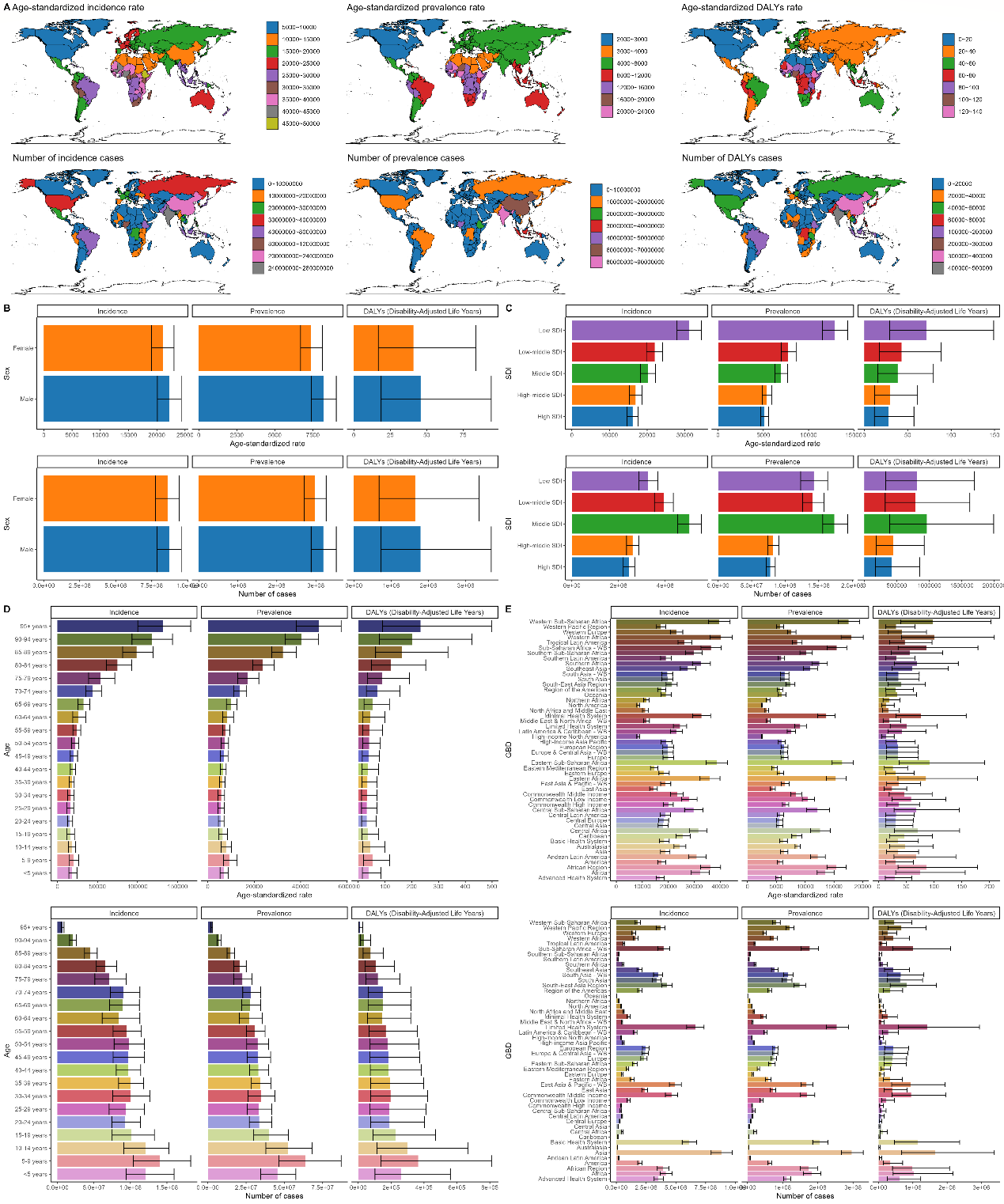


**Supplementary** Figure S27. Global distribution and trends in Fungal skin diseases burden, illustrating the differences in incidence, prevalence, DALYs, and number of cases across regions and demographics.

(A) Global distribution of ASRs for SSD incidence, prevalence, and DALYs. (B) Bar plots comparing Fungal skin diseases incidence, prevalence, and DALYs by sex and age group. (C) Bar plots showing Fungal skin diseases incidence, prevalence, and DALYs by SDI level. (D) Age-specific distribution of Fungal skin diseases burden. (E) Stratified analysis of Fungal skin diseases burden by GBD regions. Abbreviations: SSDs, Skin and subcutaneous diseases; DALYs: Disability-adjusted life years; ASRs, Age-standardized rates; SDI, Socio-demographic index; GBD, Global Burden of Disease.


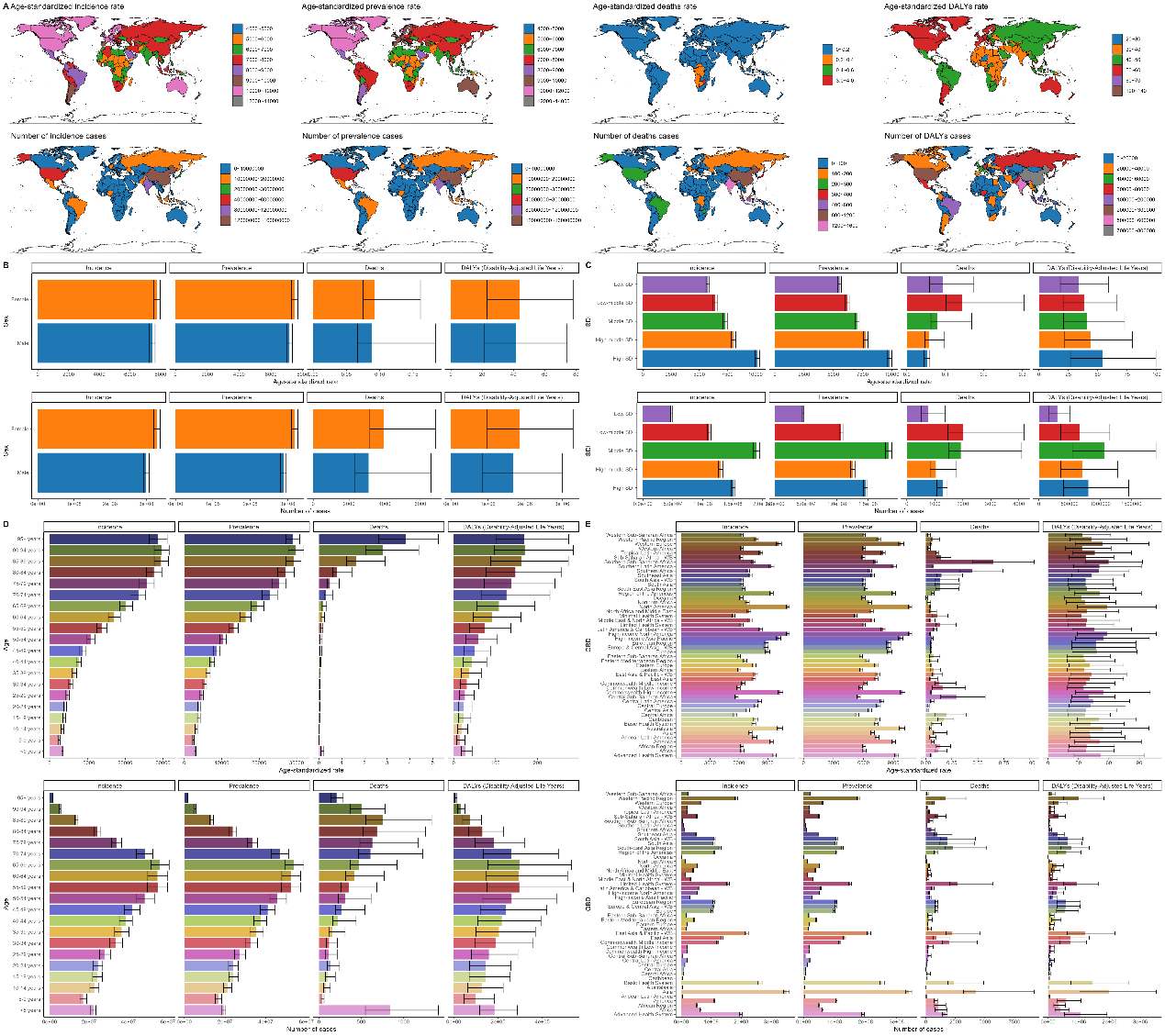


**Supplementary** Figure S28. Global distribution and trends in Other skin and subcutaneous diseases burden, illustrating the differences in incidence, prevalence, mortality and DALYs, and number of cases across regions and demographics.

(A) Global distribution of ASRs for SSD incidence, prevalence, mortality and DALYs. (B) Bar plots comparing Cellulitis incidence, prevalence, mortality and DALYs by sex and age group. (C) Bar plots showing Cellulitis incidence, prevalence, mortality and DALYs by SDI level. (D) Age-specific distribution of Other skin and subcutaneous diseases burden. (E) Stratified analysis of Other skin and subcutaneous diseases burden by GBD regions. Abbreviations: SSDs, Skin and subcutaneous diseases; DALYs: Disability-adjusted life years; ASRs, Age-standardized rates; SDI, Socio-demographic index; GBD, Global Burden of Disease.


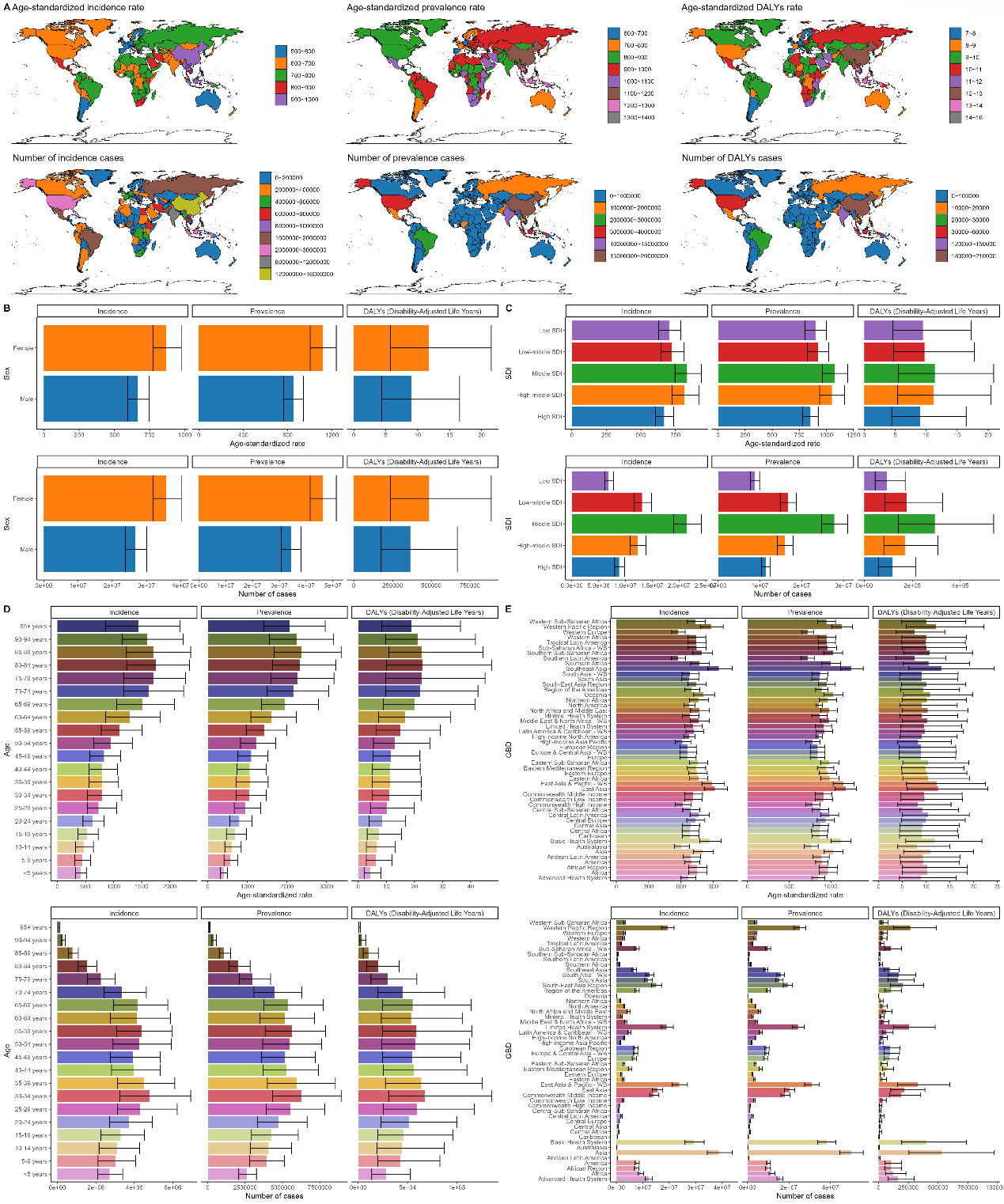


**Supplementary** Figure S29. Global distribution and trends in Pruritus burden, illustrating the differences in incidence, prevalence, DALYs, and number of cases across regions and demographics.

(A) Global distribution of ASRs for SSD incidence, prevalence, and DALYs. (B) Bar plots comparing Pruritus incidence, prevalence, and DALYs by sex and age group. (C) Bar plots showing Pruritus incidence, prevalence, and DALYs by SDI level. (D) Age-specific distribution of Pruritus burden. (E) Stratified analysis of Pruritus burden by GBD regions. Abbreviations: SSDs, Skin and subcutaneous diseases; DALYs: Disability-adjusted life years; ASRs, Age-standardized rates; SDI, Socio-demographic index; GBD, Global Burden of Disease.


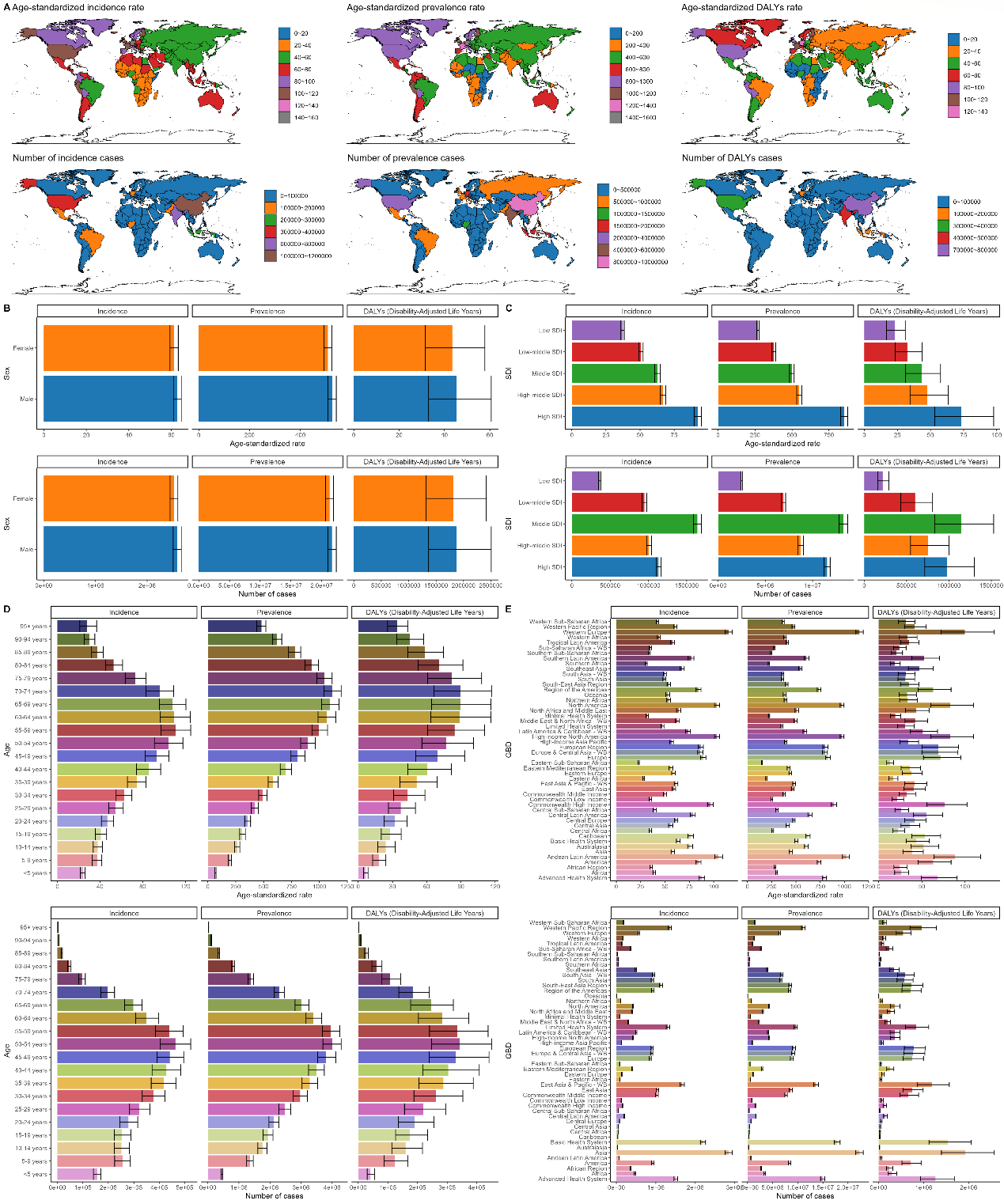


**Supplementary** Figure S30. Global distribution and trends in Psoriasis burden, illustrating the differences in incidence, prevalence, DALYs, and number of cases across regions and demographics.

(A) Global distribution of ASRs for SSD incidence, prevalence, and DALYs. (B) Bar plots comparing Psoriasis incidence, prevalence, and DALYs by sex and age group. (C) Bar plots showing Psoriasis incidence, prevalence, and DALYs by SDI level. (D) Age-specific distribution of Psoriasis burden. (E) Stratified analysis of Psoriasis burden by GBD regions. Abbreviations: SSDs, Skin and subcutaneous diseases; DALYs: Disability-adjusted life years; ASRs, Age-standardized rates; SDI, Socio-demographic index; GBD, Global Burden of Disease.


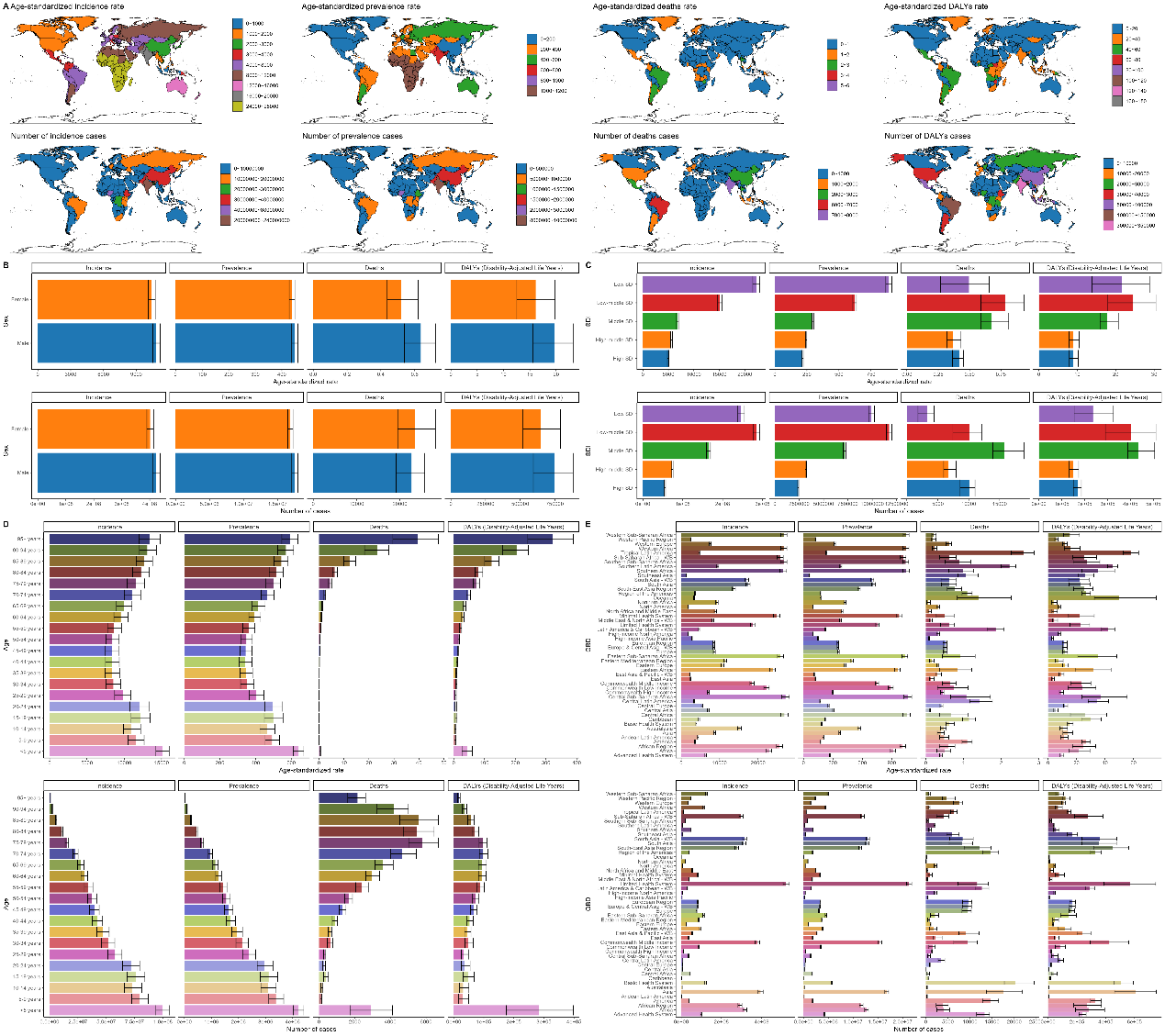


**Supplementary** Figure S31. Global distribution and trends in Pyoderma, illustrating the differences in incidence, prevalence, mortality and DALYs, and number of cases across regions and demographics.

(A) Global distribution of ASRs for SSD incidence, prevalence, mortality and DALYs. (B) Bar plots comparing Cellulitis incidence, prevalence, mortality and DALYs by sex and age group. (C) Bar plots showing Cellulitis incidence, prevalence, mortality and DALYs by SDI level. (D) Age-specific distribution of Pyoderma. (E) Stratified analysis of Pyoderma burden by GBD regions. Abbreviations: SSDs, Skin and subcutaneous diseases; DALYs: Disability-adjusted life years; ASRs, Age-standardized rates; SDI, Socio-demographic index; GBD, Global Burden of Disease.


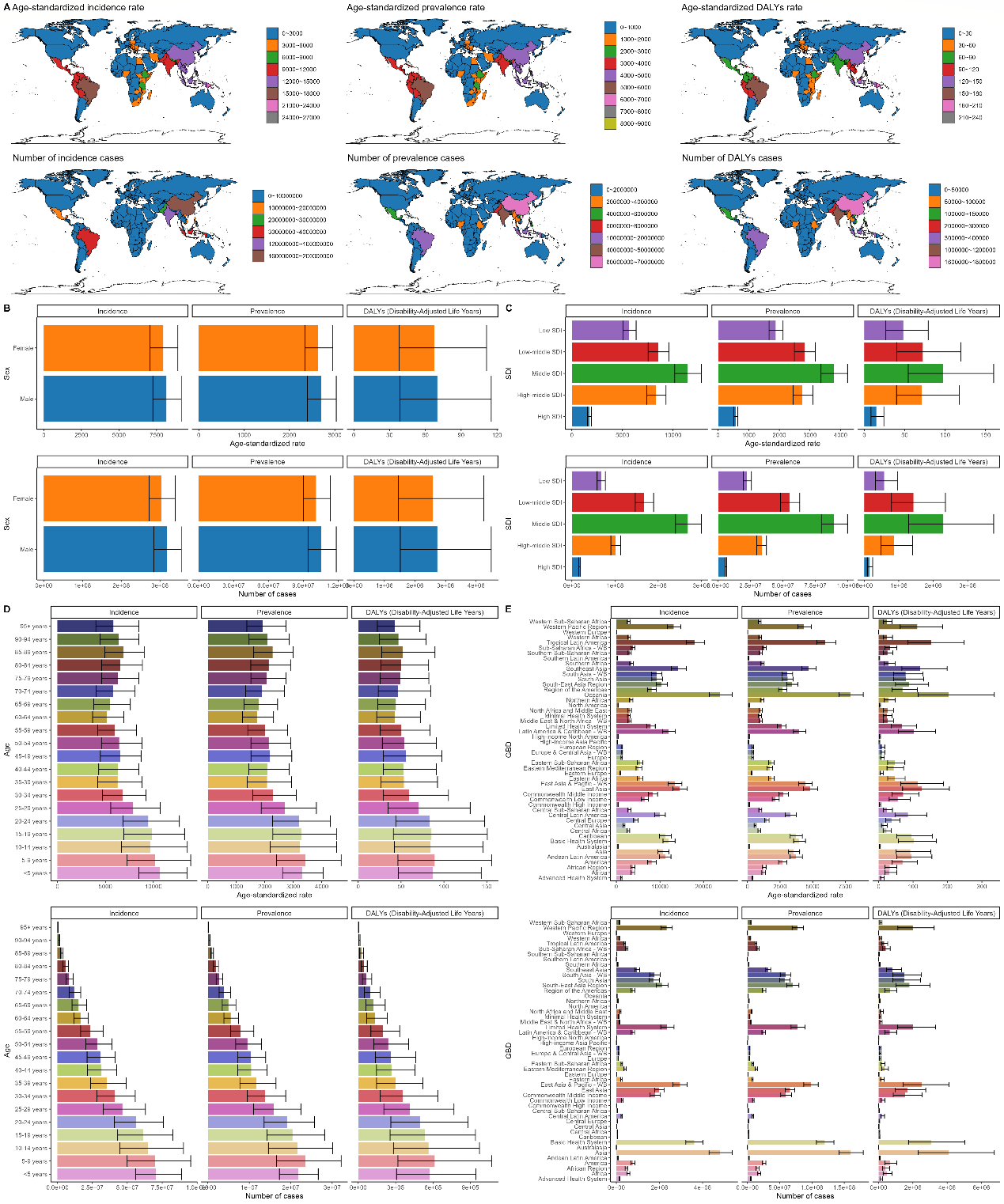


**Supplementary** Figure S32. Global distribution and trends in Scabies burden, illustrating the differences in incidence, prevalence, DALYs, and number of cases across regions and demographics.

(A) Global distribution of ASRs for SSD incidence, prevalence, and DALYs. (B) Bar plots comparing Scabies incidence, prevalence, and DALYs by sex and age group. (C) Bar plots showing Scabies incidence, prevalence, and DALYs by SDI level. (D) Age-specific distribution of Scabies burden. (E) Stratified analysis of Scabies burden by GBD regions. Abbreviations: SSDs, Skin and subcutaneous diseases; DALYs: Disability-adjusted life years; ASRs, Age-standardized rates; SDI, Socio-demographic index; GBD, Global Burden of Disease.


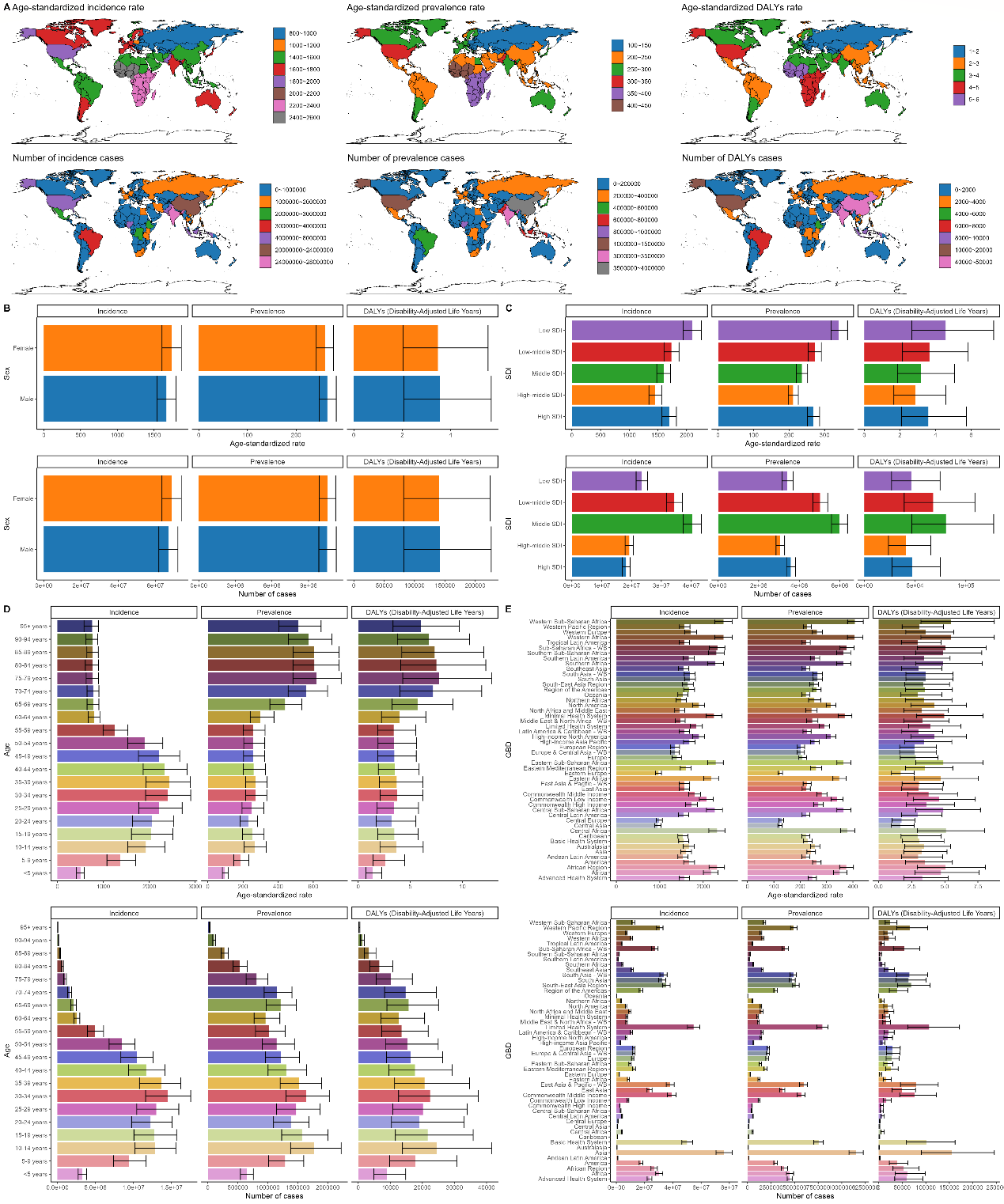


**Supplementary** Figure S33. Global distribution and trends in Seborrheic dermatitis burden, illustrating the differences in incidence, prevalence, DALYs, and number of cases across regions and demographics.

(A) Global distribution of ASRs for SSD incidence, prevalence, and DALYs. (B) Bar plots comparing Seborrheic dermatitis incidence, prevalence, and DALYs by sex and age group. (C) Bar plots showing Seborrheic dermatitis incidence, prevalence, and DALYs by SDI level. (D) Age-specific distribution of Seborrheic dermatitis burden. (E) Stratified analysis of Seborrheic dermatitis burden by GBD regions. Abbreviations: SSDs, Skin and subcutaneous diseases; DALYs: Disability-adjusted life years; ASRs, Age-standardized rates; SDI, Socio-demographic index; GBD, Global Burden of Disease.


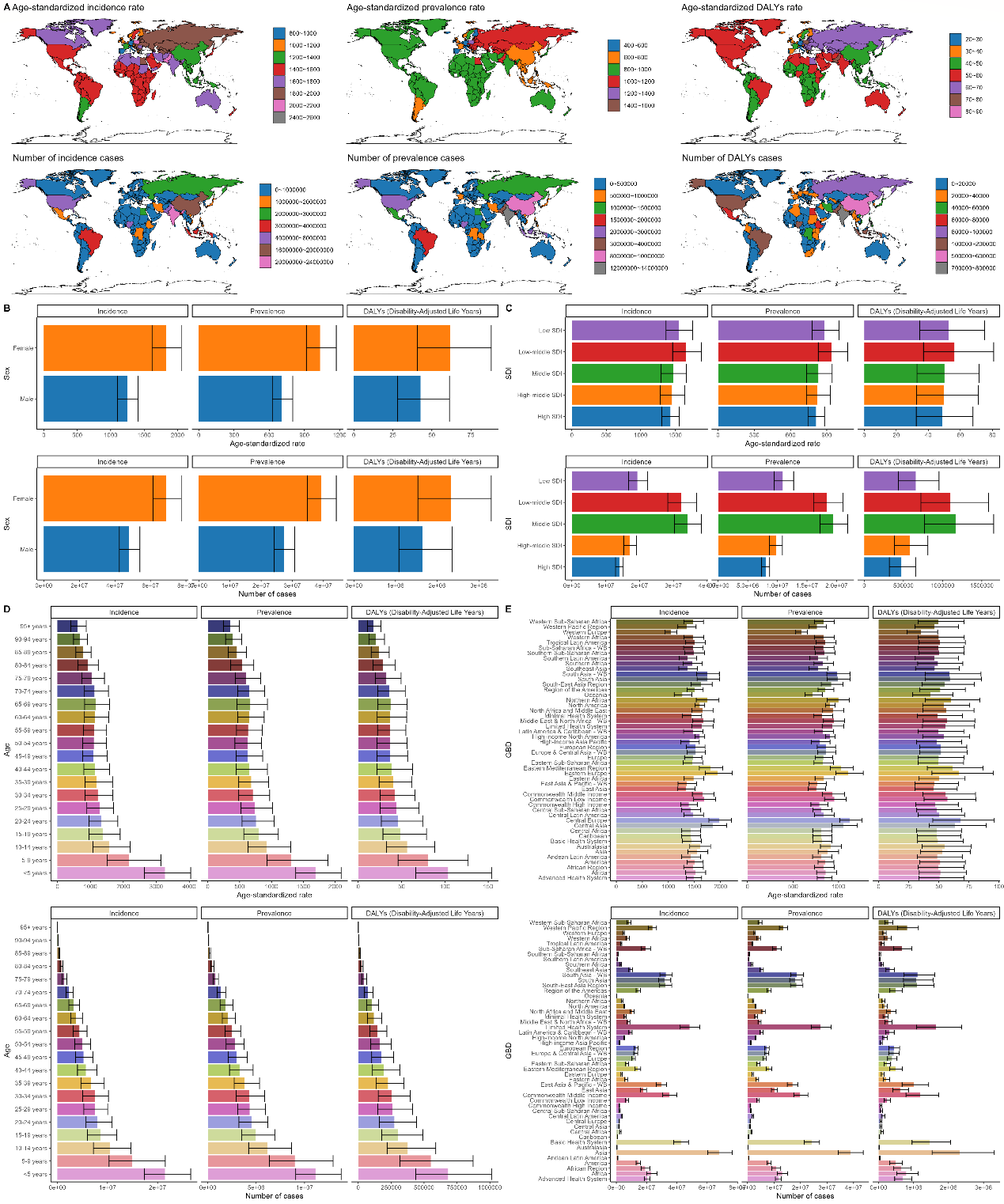


**Supplementary** Figure S34. Global distribution and trends in Urticaria burden, illustrating the differences in incidence, prevalence, DALYs, and number of cases across regions and demographics.

(A) Global distribution of ASRs for SSD incidence, prevalence, and DALYs. (B) Bar plots comparing Urticaria incidence, prevalence, and DALYs by sex and age group. (C) Bar plots showing Urticaria incidence, prevalence, and DALYs by SDI level. (D) Age-specific distribution of Urticaria burden. (E) Stratified analysis of Urticaria burden by GBD regions. Abbreviations: SSDs, Skin and subcutaneous diseases; DALYs: Disability-adjusted life years; ASRs, Age-standardized rates; SDI, Socio-demographic index; GBD, Global Burden of Disease.


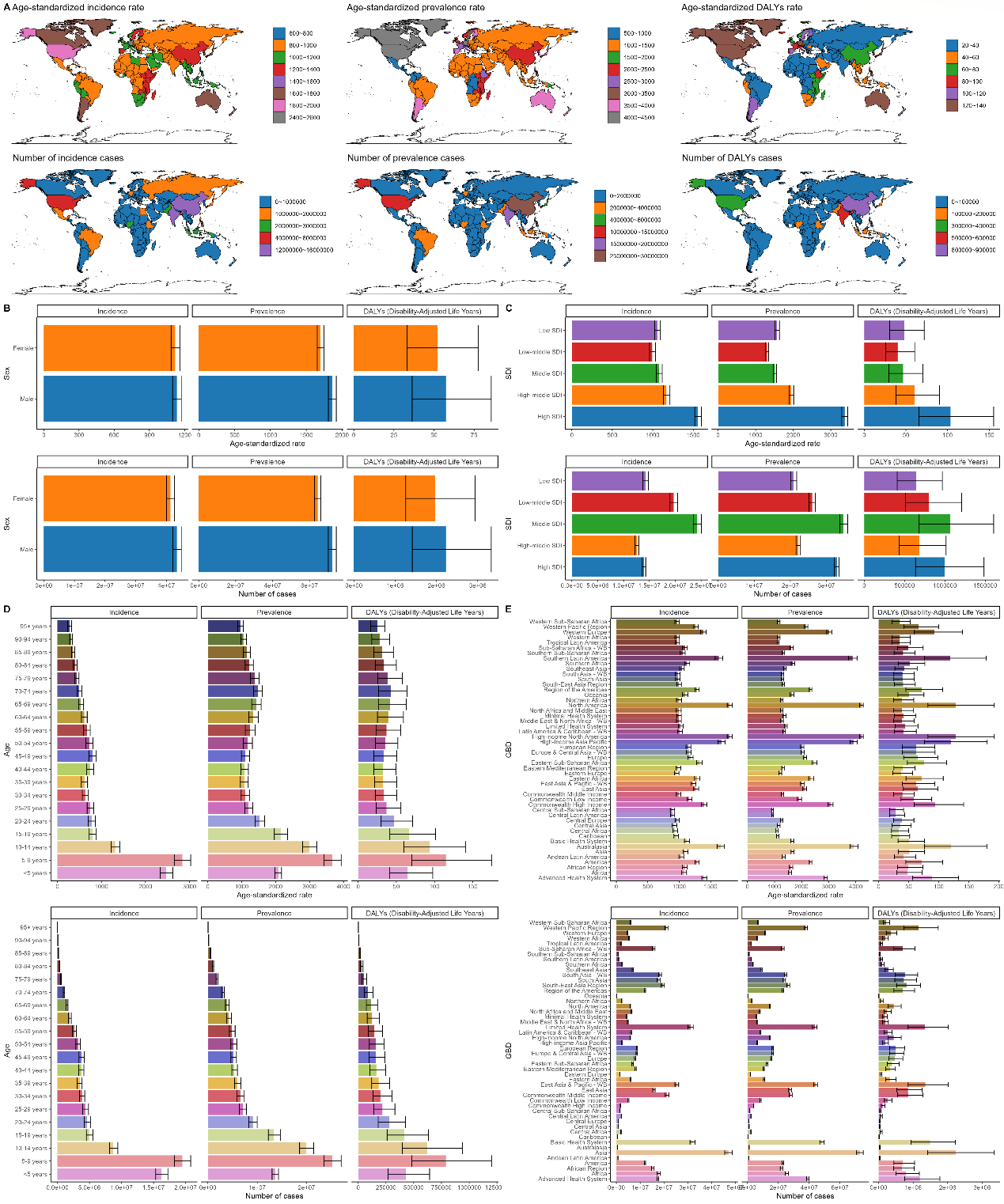


**Supplementary** Figure S35. Global distribution and trends in Viral skin diseases burden, illustrating the differences in incidence, prevalence, DALYs, and number of cases across regions and demographics.

(A) Global distribution of ASRs for SSD incidence, prevalence, and DALYs. (B) Bar plots comparing Viral skin diseases incidence, prevalence, and DALYs by sex and age group. (C) Bar plots showing Viral skin diseases incidence, prevalence, and DALYs by SDI level. (D) Age-specific distribution of Viral skin diseases burden. (E) Stratified analysis of Viral skin diseases burden by GBD regions. Abbreviations: SSDs, Skin and subcutaneous diseases; DALYs: Disability-adjusted life years; ASRs, Age-standardized rates; SDI, Socio-demographic index; GBD, Global Burden of Disease.
