# Supplementary material for: Global, regional and national burden of skin and subcutaneous diseases: a systematic analysis of the Global Burden of Disease Study 2021
Source: Int Health. 2025 Jun 28;18(2):183–96. doi: 10.1093/inthealth/ihaf070 (PMC13017215; doi:10.1093/inthealth/ihaf070)
Supplement: ihaf070_Supplemental_Files [file ihaf070_supplemental_files.zip › Supplementary Table.docx]

**Table S1** Global, all age, regional data on SSDs-related prevalence, incidence, DALYs and mortality, and temporal trends from 1990 to 2021.

|  | 1990 |  |  |  |  |  |  |  | 2021 |  |  |  |  |  |  |  | 1990-2021 | |  |  |
| --- | --- | --- | --- | --- | --- | --- | --- | --- | --- | --- | --- | --- | --- | --- | --- | --- | --- | --- | --- | --- |
|  | Prevalence |  | incidence |  | DALYs |  | Death |  | Prevalence |  | Incidence |  | DALYs |  | Death |  | ASPR | ASIR | ASDR | ASMR |
| Characteristics | Numbers (95% UI) | ASR | Numbers (95% UI) | ASR | Numbers (95% UI) | ASR | Numbers (95% UI) | ASR | Numbers (95% UI) | ASR | Numbers (95% UI) | ASR | Numbers (95% UI) | ASR | Numbers (95% UI) | ASR | (95% CI) | (95% CI) | (95% CI) | (95% CI) |
|  |  | No. ×10-5 (95%UI) |  | No. ×10-5 (95%UI) |  | No. ×10-5 (95%UI) |  | No. ×10-5 (95%UI) |  | No. ×10-5 (95%UI) |  | No. ×10-5 (95%UI) |  | No. ×10-5 (95%UI) |  | No. ×10-5 (95%UI) | EAPC_CI | EAPC_CI | EAPC_CI | EAPC_CI |
| Global | 1261208676 (1220122078-1302296796) | 24262.38 (23517.19-25004.78) | 2840135563 (2717363229-2977067277) | 55371.82 (52979.87-57837.46) | 28425933 (18945310-40409003) | 529.54 (352.59-752.08) | 51591 (46232-57621) | 1.39 (1.22-1.56) | 2031912754 (1968859875-2094361908) | 25567.9 (24782.89-26381.91) | 4693912471 (4497714609-4895691752) | 59091.81 (56658.66-61721.71) | 41944109 (27746948-59789982) | 535.3 (353.96-763.28) | 119129 (108214-126524) | 1.47 (1.34-1.57) | 0.19 (0.18-0.2) | 0.23 (0.21-0.24) | 0.05 (0.03-0.06) | 0.15 (0.02-0.28) |
| Sex |  |  |  |  |  |  |  |  |  |  |  |  |  |  |  |  |  |  |  |  |
| Female | 650730489 (630654269-670857953) | 24976.9 (24243.11-25706.61) | 1431180423 (1368840482-1497050659) | 55605.69 (53226.61-58041.32) | 14957936 (9972908-21204670) | 558.31 (371.6-791.25) | 26847 (24074-30483) | 1.3 (1.15-1.48) | 1043532481 (1013123409-1073475231) | 26122.8 (25334.67-26881.99) | 2375522781 (2275734303-2476446145) | 59325.23 (56866.83-61844.26) | 21816985 (14468887-31061498) | 558.08 (369.74-795.18) | 63045 (55363-68357) | 1.38 (1.22-1.49) | 0.16 (0.15-0.17) | 0.22 (0.21-0.24) | 0.01 (0-0.02) | 0.17 (0.04-0.31) |
| Male | 610478187 (588932365-631700955) | 23579.8 (22803.04-24350.33) | 1408955139 (1347869578-1480427397) | 55151.11 (52730.83-57629.21) | 13467997 (8972402-19204333) | 502.02 (334.95-714.5) | 24744 (21478-27769) | 1.5 (1.3-1.69) | 988380273 (955536443-1021727108) | 25040.73 (24211.14-25892.96) | 2318389690 (2221377457-2420225033) | 58869 (56342.79-61452.86) | 20127124 (13310320-28728484) | 513.67 (340.21-733.03) | 56084 (52177-59461) | 1.58 (1.45-1.67) | 0.21 (0.2-0.22) | 0.23 (0.22-0.25) | 0.08 (0.07-0.1) | 0.07 (-0.06-0.21) |
| Age |  |  |  |  |  |  |  |  |  |  |  |  |  |  |  |  |  |  |  |  |
| <5 years | 118981039 (110192338-128798720) | 19192.41 (17774.74-20776.07) | 316574930 (289303324-350362646) | 51065.59 (46666.5-56515.76) | 3901319 (2727271-5456582) | 629.31 (439.93-880.18) | 8971 (6780-11308) | 1.45 (1.09-1.82) | 132866249 (122345820-144322808) | 20187.17 (18588.74-21927.84) | 366534739 (334979125-406762969) | 55689.84 (50895.4-61801.95) | 3709927 (2437405-5327260) | 563.67 (370.33-809.4) | 4503 (3166-6170) | 0.68 (0.48-0.94) | 0.11 (0.09-0.13) | 0.21 (0.17-0.26) | -0.41 (-0.43--0.38) | -2.42 (-2.59--2.25) |
| 5-9 years | 133561936 (120869597-149368008) | 22888.59 (20713.49-25597.28) | 286264566 (252656676-326485014) | 49057.33 (43297.92-55949.93) | 3494768 (2139915-5255888) | 598.9 (366.72-900.7) | 598 (484-723) | 0.1 (0.08-0.12) | 166188104 (149428907-187265101) | 24188.52 (21749.24-27256.26) | 368549202 (326609785-420920329) | 53641.99 (47537.75-61264.56) | 4098943 (2499812-6174641) | 596.6 (363.85-898.71) | 259 (206-322) | 0.04 (0.03-0.05) | 0.19 (0.16-0.22) | 0.31 (0.27-0.35) | -0.04 (-0.05--0.02) | -3.13 (-3.47--2.79) |
| 10-14 years | 142158641 (131461771-154480070) | 26537.85 (24540.98-28837.98) | 275290189 (245033468-309218948) | 51390.54 (45742.28-57724.28) | 3511561 (2244493-5098419) | 655.53 (419-951.76) | 561 (462-672) | 0.1 (0.09-0.13) | 191416854 (176904260-208117776) | 28713.85 (26536.86-31219.1) | 383111511 (342198678-429944951) | 57469.37 (51332.17-64494.71) | 4548450 (2903621-6591185) | 682.3 (435.56-988.72) | 404 (344-483) | 0.06 (0.05-0.07) | 0.26 (0.24-0.28) | 0.4 (0.36-0.44) | 0.12 (0.1-0.14) | -1.63 (-1.84--1.42) |
| 15-19 years | 149356516 (138170744-162254939) | 28754.29 (26600.79-31237.51) | 257813296 (231360531-289747907) | 49634.51 (44541.8-55782.6) | 3603812 (2335489-5365346) | 693.81 (449.63-1032.94) | 929 (747-1168) | 0.18 (0.14-0.22) | 192630170 (178142826-209700399) | 30871.22 (28549.45-33606.92) | 339120805 (306302732-379614500) | 54348.04 (49088.56-60837.62) | 4519581 (2912573-6698923) | 724.32 (466.77-1073.58) | 1001 (806-1248) | 0.16 (0.13-0.2) | 0.25 (0.23-0.28) | 0.33 (0.28-0.37) | 0.17 (0.15-0.2) | -0.58 (-0.71--0.45) |
| 20-24 years | 103584609 (96077822-111593290) | 21050.02 (19524.52-22677.51) | 231077042 (206278642-255939915) | 46958.49 (41919.06-52011.02) | 2356369 (1536351-3527709) | 478.85 (312.21-716.89) | 760 (622-935) | 0.15 (0.13-0.19) | 129824837 (120592618-139666755) | 21740.45 (20194.42-23388.57) | 303214113 (273797648-332131350) | 50776.18 (45850.11-55618.66) | 2817871 (1835101-4236039) | 471.88 (307.31-709.37) | 800 (714-896) | 0.13 (0.12-0.15) | 0.15 (0.12-0.19) | 0.29 (0.26-0.33) | 0.03 (-0.02-0.07) | -0.79 (-1--0.59) |
| 25-29 years | 85394055 (78860413-92614216) | 19292.87 (17816.74-20924.11) | 201455484 (179725099-226169156) | 45514.36 (40604.86-51097.86) | 1826491 (1192360-2652673) | 412.65 (269.39-599.31) | 623 (525-747) | 0.14 (0.12-0.17) | 117520698 (108365939-127285679) | 19974.85 (18418.83-21634.6) | 287697977 (257949460-320552710) | 48899.68 (43843.36-54483.96) | 2399687 (1555418-3480311) | 407.87 (264.37-591.54) | 803 (738-881) | 0.14 (0.13-0.15) | 0.14 (0.12-0.16) | 0.25 (0.23-0.28) | 0 (-0.03-0.04) | -0.28 (-0.46--0.1) |
| 30-34 years | 73356031 (68196241-79149654) | 19032.63 (17693.89-20535.82) | 174309930 (154989753-194789585) | 45225.68 (40212.95-50539.24) | 1511330 (990931-2183356) | 392.12 (257.1-566.48) | 981 (807-1206) | 0.25 (0.21-0.31) | 119794375 (111422234-129127933) | 19817.75 (18432.74-21361.81) | 291316554 (259058223-324291487) | 48192.9 (42856.36-53647.99) | 2380977 (1559934-3461370) | 393.89 (258.06-572.62) | 1623 (1441-1846) | 0.27 (0.24-0.31) | 0.14 (0.13-0.16) | 0.24 (0.22-0.26) | 0.01 (0-0.03) | 0.16 (0.05-0.27) |
| 35-39 years | 68649201 (63891507-73768590) | 19489.08 (18138.4-20942.44) | 164989930 (148848448-181244810) | 46839.61 (42257.14-51454.27) | 1361179 (915607-1934885) | 386.43 (259.94-549.3) | 1034 (889-1218) | 0.29 (0.25-0.35) | 114202561 (106574703-122710644) | 20361.82 (19001.81-21878.78) | 279507953 (253128323-306534546) | 49835.06 (45131.68-54653.78) | 2177763 (1474002-3100301) | 388.29 (262.81-552.77) | 1753 (1635-1878) | 0.31 (0.29-0.33) | 0.17 (0.16-0.18) | 0.24 (0.22-0.25) | 0.03 (0.02-0.05) | 0.12 (-0.04-0.28) |
| 40-44 years | 59050452 (55009622-63364316) | 20612.3 (19201.8-22118.11) | 141905044 (129174003-155929688) | 49533.73 (45089.8-54429.21) | 1141914 (760059-1628713) | 398.6 (265.31-568.52) | 1154 (1001-1371) | 0.4 (0.35-0.48) | 108409524 (101218295-116319779) | 21671.03 (20233.51-23252.29) | 264442622 (240843753-290195638) | 52862.01 (48144.6-58010.03) | 2029157 (1357311-2906279) | 405.63 (271.33-580.96) | 2369 (2209-2537) | 0.47 (0.44-0.51) | 0.2 (0.19-0.21) | 0.24 (0.22-0.25) | 0.09 (0.07-0.11) | 0.22 (-0.01-0.44) |
| 45-49 years | 50573763 (46620626-55216550) | 21780.68 (20078.18-23780.2) | 122429378 (109775677-137174917) | 52726.85 (47277.26-59077.34) | 962752 (647365-1374476) | 414.63 (278.8-591.95) | 1466 (1251-1780) | 0.63 (0.54-0.77) | 109217918 (100788194-119185864) | 23065.86 (21285.58-25171) | 263660677 (236831549-295439821) | 55682.81 (50016.73-62394.28) | 2035269 (1376971-2911878) | 429.83 (290.8-614.96) | 3351 (3099-3606) | 0.71 (0.65-0.76) | 0.22 (0.21-0.24) | 0.22 (0.19-0.24) | 0.14 (0.13-0.16) | 0.19 (0.02-0.37) |
| 50-54 years | 49643791 (46111920-53778580) | 23353.93 (21692.43-25299.06) | 119811588 (107507752-133428734) | 56362.97 (50574.87-62768.88) | 922063 (627301-1312243) | 433.77 (295.1-617.32) | 1922 (1675-2286) | 0.9 (0.79-1.08) | 110874074 (103249844-119681691) | 24919.84 (23206.23-26899.42) | 264223420 (238041203-293534683) | 59386.33 (53501.66-65974.27) | 2028252 (1384174-2905310) | 455.87 (311.1-652.99) | 4318 (4025-4630) | 0.97 (0.9-1.04) | 0.24 (0.22-0.25) | 0.19 (0.17-0.22) | 0.18 (0.16-0.19) | 0.17 (0.07-0.26) |
| 55-59 years | 48340970 (45016535-51707983) | 26102.04 (24306.99-27920.08) | 113609059 (102208222-126500580) | 61344 (55188.04-68304.86) | 869273 (593659-1243822) | 469.37 (320.55-671.61) | 2541 (2177-3054) | 1.37 (1.18-1.65) | 110470383 (103035164-117625224) | 27915.74 (26036.86-29723.76) | 254132273 (229439672-281724341) | 64218.93 (57979.13-71191.41) | 1979234 (1359653-2852559) | 500.15 (343.58-720.84) | 6349 (5935-6765) | 1.6 (1.5-1.71) | 0.25 (0.24-0.26) | 0.17 (0.15-0.19) | 0.23 (0.22-0.25) | 0.56 (0.36-0.76) |
| 60-64 years | 47039881 (43579910-50982820) | 29288.4 (27134.12-31743.39) | 107830323 (95138962-123737163) | 67138.31 (59236.3-77042.37) | 813881 (564088-1175669) | 506.75 (351.22-732.01) | 3096 (2673-3607) | 1.93 (1.66-2.25) | 100052242 (93225078-107687760) | 31261.65 (29128.48-33647.39) | 225029015 (200061754-256683572) | 70311.05 (62509.95-80201.62) | 1735057 (1205383-2474084) | 542.12 (376.63-773.04) | 7714 (7261-8227) | 2.41 (2.27-2.57) | 0.24 (0.23-0.25) | 0.14 (0.13-0.15) | 0.24 (0.23-0.25) | 0.54 (0.38-0.71) |
| 65-69 years | 41873797 (38927694-45166572) | 33875.88 (31492.49-36539.74) | 97583639 (87689785-109885545) | 78945.12 (70941-88897.36) | 697570 (480491-1024971) | 564.33 (388.72-829.2) | 3624 (3123-4255) | 2.93 (2.53-3.44) | 98188410 (91775766-105107402) | 35595.87 (33271.12-38104.18) | 223848803 (202516133-251211129) | 81151.05 (73417.4-91070.61) | 1649803 (1152579-2388408) | 598.1 (417.84-865.86) | 9886 (9264-10533) | 3.58 (3.36-3.82) | 0.2 (0.19-0.22) | 0.11 (0.1-0.12) | 0.24 (0.22-0.26) | 0.67 (0.55-0.79) |
| 70-74 years | 33165818 (30989242-35350201) | 39174.78 (36603.85-41754.92) | 80507201 (72042571-89890355) | 95093.44 (85095.19-106176.62) | 537328 (372215-786201) | 634.68 (439.65-928.64) | 4244 (3692-4976) | 5.01 (4.36-5.88) | 84766173 (79567905-90071342) | 41180.77 (38655.37-43758.1) | 200827704 (180694120-222741325) | 97565.32 (87784.11-108211.31) | 1390433 (986616-2001573) | 675.49 (479.31-972.4) | 12279 (11427-13101) | 5.97 (5.55-6.36) | 0.17 (0.16-0.18) | 0.1 (0.09-0.11) | 0.22 (0.2-0.25) | 0.66 (0.55-0.77) |
| 75-79 years | 27104749 (24932774-29521111) | 44033.01 (40504.53-47958.51) | 67714696 (57772418-79632147) | 110005.9 (93854.17-129366.39) | 438274 (307937-625691) | 712 (500.26-1016.47) | 5734 (5057-6715) | 9.31 (8.21-10.91) | 59956272 (55500414-64801531) | 45461.23 (42082.62-49135.1) | 146067893 (125750889-169829584) | 110754.48 (95349.32-128771.54) | 997508 (717991-1410672) | 756.35 (544.41-1069.63) | 14565 (13396-15471) | 11.04 (10.16-11.73) | 0.13 (0.12-0.13) | 0.07 (0.06-0.08) | 0.17 (0.14-0.21) | 0.44 (0.32-0.57) |
| 80-84 years | 17526527 (16264661-18839163) | 49543.65 (45976.64-53254.19) | 47419847 (42080305-53959806) | 134045.52 (118951.81-152532.56) | 273968 (192447-388928) | 774.45 (544.01-1099.41) | 5306 (4607-6119) | 15 (13.02-17.3) | 44391857 (41324113-47435117) | 50685.44 (47182.77-54160.15) | 117484399 (104680216-133038567) | 134140.56 (119521.08-151899.89) | 710175 (498948-1007515) | 810.86 (569.69-1150.35) | 14760 (13002-15898) | 16.85 (14.85-18.15) | 0.08 (0.07-0.09) | 0.02 (0-0.04) | 0.16 (0.13-0.19) | 0.4 (0.28-0.52) |
| 85-89 years | 8553631 (8000029-9170336) | 56605.05 (52941.5-60686.2) | 24151742 (21373501-27299824) | 159828.11 (141442.65-180661.06) | 140204 (102132-197714) | 927.82 (675.88-1308.41) | 4731 (4097-5358) | 31.31 (27.11-35.46) | 26292672 (24729185-28128654) | 57505.78 (54086.21-61521.33) | 72906155 (64892112-82346770) | 159456.03 (141928.19-180103.99) | 438380 (322860-612914) | 958.8 (706.14-1340.53) | 15124 (12549-16659) | 33.08 (27.45-36.43) | 0.06 (0.05-0.08) | 0 (-0.01-0.02) | 0.14 (0.09-0.18) | 0.22 (0.07-0.38) |
| 90-94 years | 2639384 (2439040-2844781) | 61593.19 (56917.92-66386.37) | 7527911 (6501404-8647420) | 175672.84 (151718.06-201797.93) | 48148 (36640-64719) | 1123.58 (855.03-1510.3) | 2438 (2032-2760) | 56.88 (47.41-64.42) | 11213111 (10419467-12055359) | 62680.29 (58243.89-67388.38) | 31811513 (27495870-36569889) | 177823.51 (153699.46-204422.41) | 214521 (166995-285253) | 1199.16 (933.49-1594.54) | 11447 (9138-12833) | 63.99 (51.08-71.74) | 0.06 (0.05-0.07) | 0.03 (0.02-0.05) | 0.19 (0.12-0.26) | 0.29 (0.12-0.45) |
| 95+ years | 653887 (591507-717012) | 64227.03 (58099.85-70427.38) | 1869769 (1547646-2197967) | 183655.19 (152015.15-215891.92) | 13731 (10845-17680) | 1348.66 (1065.28-1736.59) | 880 (676-1024) | 86.45 (66.37-100.62) | 3636268 (3298563-3987580) | 66716.73 (60520.67-73162.47) | 10425143 (8645561-12365385) | 191276.2 (158625.15-226874.95) | 83120 (66267-108163) | 1525.05 (1215.84-1984.53) | 5819 (4268-6681) | 106.77 (78.31-122.59) | 0.12 (0.09-0.14) | 0.14 (0.1-0.17) | 0.32 (0.21-0.42) | 0.53 (0.33-0.73) |
| SDI region |  |  |  |  |  |  |  |  |  |  |  |  |  |  |  |  |  |  |  |  |
| High-middle SDI | 241941458 (235196949-248591564) | 22961.43 (22332.73-23558.29) | 508389984 (484861568-532917033) | 48592.05 (46402.6-50883.23) | 5318323 (3496631-7580336) | 505.32 (332.45-720.01) | 6854 (6256-7464) | 0.84 (0.75-0.92) | 335817804 (327169206-343809739) | 24448.5 (23784.48-25067.34) | 708130196 (674072488-741776043) | 49709.51 (47542.34-51945.13) | 6833944 (4518305-9733211) | 538.2 (355.74-767.42) | 20427 (18379-21773) | 1.1 (0.99-1.18) | 0.23 (0.22-0.24) | 0.08 (0.08-0.09) | 0.23 (0.22-0.23) | 0.84 (0.62-1.06) |
| High SDI | 232682367 (227842798-237373379) | 25624.45 (25083.06-26134.49) | 415866543 (397929008-432871382) | 44385.95 (42610.29-46107.27) | 5254867 (3512309-7348798) | 605.93 (402.74-852.41) | 10358 (9403-10947) | 0.97 (0.88-1.03) | 325874100 (320016554-331844074) | 26577.69 (26103.82-27054.73) | 601244525 (575468954-628640312) | 45122.45 (43432.12-46728.4) | 6788696 (4606109-9439622) | 616.79 (412-863.14) | 26755 (22796-28930) | 1.12 (0.97-1.2) | 0.12 (0.12-0.13) | 0.08 (0.07-0.08) | 0.07 (0.06-0.08) | 0.58 (0.45-0.72) |
| Low-middle SDI | 254990307 (244177007-266283183) | 22623.13 (21818.37-23465.3) | 669132700 (638016846-705276390) | 60903.27 (58335.94-63567.32) | 6027110 (4096477-8516396) | 499.36 (341.98-704.59) | 14457 (12351-16357) | 2.13 (1.79-2.5) | 447197727 (431479426-463256024) | 23821.66 (23035.71-24648.43) | 1166336604 (1118403687-1218435180) | 63035.03 (60474.43-65657.37) | 9562236 (6401491-13637824) | 502.49 (340.84-714.32) | 26411 (24143-28847) | 2.09 (1.9-2.29) | 0.19 (0.16-0.21) | 0.13 (0.1-0.16) | 0.02 (-0.01-0.05) | -0.18 (-0.32--0.05) |
| Low SDI | 135430972 (126547424-144567825) | 26975.7 (25710.04-28329.02) | 362405374 (342742275-384645793) | 74976.29 (71529.48-78636.76) | 2701404 (1813033-3912689) | 504.9 (339.71-734.05) | 6533 (5139-8249) | 2.21 (1.66-3.16) | 301231209 (284702105-319452038) | 27178.26 (26004.55-28429.07) | 810131490 (768785318-856739596) | 75800.46 (72471.98-79452.07) | 5724408 (3776365-8363296) | 492.27 (328.38-721.54) | 9638 (7510-12296) | 1.78 (1.46-2.22) | 0.01 (0-0.03) | 0.03 (0.01-0.05) | -0.1 (-0.12--0.07) | -0.83 (-0.92--0.73) |
| Middle SDI | 394997304 (381537884-408542383) | 23727.72 (22959.06-24519.89) | 881746010 (840842243-930192268) | 54361.88 (51867.84-57137.95) | 9098983 (5983346-13091489) | 524.63 (346.7-753.5) | 13337 (11845-15295) | 1.53 (1.34-1.82) | 620162774 (600539569-639173949) | 25164.17 (24369.93-25951.66) | 1404380477 (1341496115-1473122864) | 56798.67 (54352.01-59558.36) | 13000854 (8572830-18667950) | 541.29 (357.22-777.6) | 35766 (32115-39124) | 1.56 (1.38-1.71) | 0.21 (0.2-0.22) | 0.17 (0.15-0.18) | 0.12 (0.1-0.13) | 0.01 (-0.14-0.17) |
| GBD region |  |  |  |  |  |  |  |  |  |  |  |  |  |  |  |  |  |  |  |  |
| Advanced Health System | 332581482 (325400791-340414300) | 24430.42 (23886.94-24987.4) | 627373664 (599211697-655104919) | 44869.98 (43008.84-46723.61) | 7276213 (4844688-10217785) | 559.96 (372.18-788.88) | 12830 (11766-13500) | 0.85 (0.77-0.89) | 436366541 (427831258-445418679) | 25502.43 (24996.17-26012.17) | 842336707 (805440303-883761179) | 45468.5 (43696.33-47240.27) | 8965587 (6094257-12452959) | 584.13 (390.72-818.13) | 37011 (32047-39677) | 1.18 (1.04-1.25) | 0.16 (0.15-0.17) | 0.06 (0.06-0.07) | 0.16 (0.15-0.17) | 1.1 (0.95-1.26) |
| Africa | 168796647 (158646815-179563390) | 27252.77 (26036.41-28595.84) | 446205258 (422914134-472837309) | 74359.55 (70993.71-78065.9) | 3161307 (2109902-4619188) | 479.13 (318.75-704.08) | 6704 (5341-8459) | 2.02 (1.54-2.85) | 377536555 (357120880-400667691) | 27859.02 (26655.32-29214.03) | 1004866089 (953890330-1062921111) | 76182.17 (72767.34-80003.15) | 6893280 (4549051-10089292) | 488.81 (327.87-716.07) | 12928 (10249-16391) | 2.03 (1.7-2.49) | 0.07 (0.06-0.07) | 0.07 (0.06-0.08) | 0.07 (0.06-0.08) | 0.03 (-0.03-0.1) |
| African Region | 147478266 (137791745-157747452) | 29366.62 (27940.39-30971.94) | 398704754 (376904510-423951152) | 82268.8 (78425.83-86452.59) | 2681796 (1792539-3948590) | 502.13 (332.45-741.92) | 6141 (4922-7717) | 2.26 (1.75-3.2) | 334547097 (315567886-356696820) | 29535.44 (28178.46-31064.77) | 910438775 (862372657-965501873) | 83065.75 (79202.18-87304.76) | 5963084 (3913101-8772174) | 505.5 (336.69-744.61) | 11434 (9040-14538) | 2.21 (1.85-2.68) | 0.01 (0-0.02) | 0.02 (0.01-0.03) | 0.02 (0.01-0.03) | -0.13 (-0.21--0.04) |
| America | 175382512 (171281394-179646352) | 24958.55 (24384.56-25534.56) | 335510516 (322254650-349827678) | 48203.49 (46287.76-50182.78) | 4170399 (2789797-5904420) | 584.02 (391.08-823.76) | 7419 (6877-7726) | 1.24 (1.14-1.29) | 277466104 (271522544-283275652) | 25917.86 (25342.53-26485.36) | 547766076 (526863101-569507055) | 50484.71 (48545.78-52523.94) | 6296149 (4352641-8729409) | 608.68 (417.49-848.49) | 29514 (25822-32962) | 2.2 (1.93-2.45) | 0.13 (0.12-0.14) | 0.17 (0.16-0.19) | 0.19 (0.17-0.21) | 2.4 (2.19-2.6) |
| Andean Latin America | 10431028 (10005015-10883621) | 29058.44 (27961.15-30187.04) | 22482329 (21167149-23928407) | 64644.82 (61026.32-68485.69) | 223568 (145249-321600) | 577.87 (374.67-835.08) | 161 (135-186) | 0.79 (0.67-0.94) | 19371957 (18634885-20134882) | 29797.05 (28711.45-30923.63) | 42114879 (39834644-44578229) | 65172.46 (61594.01-69048.92) | 396759 (258757-567330) | 607.58 (396.92-869.42) | 657 (548-767) | 1.17 (0.97-1.36) | 0.09 (0.08-0.1) | 0.03 (0.03-0.04) | 0.14 (0.1-0.17) | 0.05 (-1.13-1.25) |
| Asia | 714957243 (690413577-739995573) | 23209.05 (22462.37-23981.54) | 1639628939 (1565339174-1726774921) | 54215.35 (51836.1-56953.57) | 16884829 (11244577-24084729) | 526.43 (351.54-749.99) | 29607 (26072-33668) | 1.6 (1.39-1.87) | 1134861839 (1100512598-1168695481) | 24401.54 (23669.9-25159.99) | 2628351075 (2517507291-2748958419) | 56461.17 (54108.79-59044.96) | 23910129 (15707052-34374248) | 527.37 (346.74-758.13) | 54940 (50138-59712) | 1.24 (1.12-1.34) | 0.18 (0.17-0.2) | 0.16 (0.14-0.18) | 0.01 (-0.01-0.02) | -1.09 (-1.22--0.96) |
| Australasia | 5569235 (5460555-5678905) | 26759.26 (26237.6-27311.74) | 12627431 (12102320-13138012) | 59924.44 (57490.52-62388.87) | 108041 (70891-154885) | 541.01 (354.43-771.82) | 162 (147-174) | 0.76 (0.68-0.81) | 9713771 (9558283-9870376) | 28085.95 (27551.5-28651.27) | 22140112 (21190319-23185039) | 61522.04 (59062.36-63958.81) | 180793 (120993-257154) | 575.2 (379.53-814.38) | 952 (809-1054) | 1.52 (1.31-1.68) | 0.18 (0.17-0.19) | 0.1 (0.1-0.11) | 0.22 (0.21-0.24) | 2.35 (2.09-2.6) |
| Basic Health System | 528686569 (511836063-546053933) | 23669.16 (22946.07-24391.91) | 1126341844 (1071387657-1186893837) | 51679.08 (49235.48-54356.17) | 12226588 (8008008-17610137) | 528.49 (346.91-760.03) | 16572 (14662-19400) | 1.42 (1.24-1.7) | 804576921 (780710242-828176832) | 25161.26 (24414.45-25884.54) | 1712642082 (1632105075-1798669186) | 53030.6 (50593.18-55728.85) | 16968123 (11155847-24390088) | 550.03 (361.86-790.81) | 45899 (41114-49989) | 1.44 (1.28-1.58) | 0.21 (0.21-0.22) | 0.09 (0.08-0.1) | 0.14 (0.13-0.15) | 0.13 (-0.01-0.27) |
| Caribbean | 8604280 (8281496-8904726) | 25348.91 (24452.91-26165.11) | 19538402 (18507100-20736427) | 58814.8 (55829.21-62518.79) | 195920 (132081-279307) | 560.64 (380.48-795.49) | 677 (621-735) | 2.86 (2.62-3.12) | 12482657 (12063771-12881935) | 25816.63 (24929.49-26671.52) | 28887857 (27436815-30654147) | 59272.21 (56268.54-62847.54) | 275742 (189520-385094) | 583.9 (400.57-816.36) | 1638 (1419-1866) | 3.05 (2.64-3.46) | 0.07 (0.07-0.08) | 0.03 (0.03-0.04) | 0.15 (0.13-0.16) | 0.23 (0.1-0.36) |
| Central Africa | 18101296 (16266930-20161936) | 26758.57 (24995.14-28720.64) | 49423469 (46353495-53236404) | 77694.63 (73943.1-81971.1) | 333117 (224985-479343) | 475.08 (324.59-679.8) | 1033 (793-1322) | 2.9 (2.29-3.74) | 42707455 (39417678-46304152) | 25771.96 (24423.57-27306.72) | 121209101 (114269242-129324557) | 77321.19 (73683.94-81417.95) | 775687 (510487-1131717) | 457.68 (309.94-656.29) | 1694 (1242-2408) | 2.5 (1.8-3.33) | -0.11 (-0.13--0.1) | 0 (-0.02-0.01) | -0.12 (-0.15--0.1) | -0.63 (-0.7--0.55) |
| Central Asia | 14414041 (13997923-14914975) | 21465.13 (20816.33-22190.46) | 27080952 (25802092-28507286) | 42919.57 (40750.87-45159.38) | 363359 (226672-544477) | 492.35 (311.51-726.21) | 59 (50-68) | 0.11 (0.09-0.13) | 20443344 (19825386-21106981) | 22029 (21356.7-22750.84) | 40013179 (38207204-42199546) | 43706.42 (41560.52-45941.88) | 481606 (308076-705426) | 508.93 (326.07-743.46) | 217 (192-243) | 0.26 (0.23-0.29) | 0.13 (0.09-0.17) | 0.09 (0.07-0.12) | 0.14 (0.12-0.16) | 3.48 (2.92-4.05) |
| Central Europe | 26096009 (25330718-26965625) | 20294.6 (19726.43-20958.77) | 58509198 (55449053-61586699) | 44870.29 (42665.78-47088.39) | 511700 (333422-732015) | 418.28 (273.14-599.87) | 470 (452-488) | 0.36 (0.34-0.37) | 28095556 (27236664-29000335) | 21313.77 (20757.91-21972.62) | 65010013 (61326604-68703582) | 45878.37 (43665.47-48090.26) | 498875 (331067-713363) | 440.6 (289.7-628.75) | 1338 (1223-1450) | 0.62 (0.57-0.67) | 0.19 (0.18-0.2) | 0.09 (0.08-0.1) | 0.21 (0.18-0.24) | 2.96 (1.62-4.32) |
| Central Latin America | 34435343 (33340697-35545438) | 22361.34 (21742.93-23002.91) | 71631633 (68424341-75630594) | 48363.59 (46149.15-50804.83) | 821621 (547723-1172949) | 499.38 (335.61-707.41) | 1565 (1505-1613) | 2 (1.9-2.07) | 59323977 (57551975-60990289) | 23494 (22825.41-24137.06) | 127551342 (121345795-134078332) | 50695.35 (48242.81-53257.23) | 1337051 (924351-1871991) | 534.7 (368.61-749.86) | 5807 (4730-7231) | 2.41 (1.97-2.99) | 0.19 (0.17-0.2) | 0.2 (0.17-0.22) | 0.33 (0.3-0.36) | 1.35 (0.94-1.76) |
| Central Sub-Saharan Africa | 15133879 (13438365-17036984) | 26624.6 (24604.16-28884.07) | 40353471 (37690465-43792326) | 76543.96 (72967.86-81208.4) | 290853 (202783-411010) | 506.51 (356.02-713.11) | 1156 (870-1456) | 4.21 (3.22-5.33) | 35250637 (31856356-38978166) | 25487.4 (23858.15-27419.42) | 98536239 (92473369-105988918) | 76137.95 (72537.26-80412.95) | 658283 (444620-949944) | 483.9 (339.51-682.69) | 2012 (1483-2766) | 3.72 (2.55-4.76) | -0.14 (-0.15--0.13) | 0 (-0.02-0.02) | -0.16 (-0.18--0.13) | -0.5 (-0.57--0.43) |
| Commonwealth High Income | 30186019 (29517506-30840343) | 25921.37 (25336.63-26502.11) | 57689906 (54997984-60349015) | 47667.89 (45527.05-49722.15) | 634448 (424472-889174) | 577.14 (383.63-812.8) | 1427 (1306-1493) | 0.95 (0.87-1) | 44624330 (43708244-45551199) | 27280.94 (26703.96-27885.84) | 86493850 (82535141-90732504) | 49214.24 (47141.18-51206.52) | 911950 (625869-1262904) | 610.55 (410.05-853.24) | 4706 (4067-5037) | 1.53 (1.34-1.63) | 0.18 (0.17-0.19) | 0.12 (0.12-0.13) | 0.2 (0.18-0.22) | 1.8 (1.5-2.1) |
| Commonwealth Low Income | 51535874 (48841492-54205744) | 24738.95 (23807.79-25716.82) | 142833406 (135956119-150011080) | 70945.31 (67898.87-74195.09) | 1138984 (758742-1636578) | 502.8 (341.22-722) | 2428 (1907-3275) | 2.6 (1.96-3.61) | 100824714 (96617038-105049260) | 26071.06 (25132.23-27091.27) | 277924757 (265130831-291859205) | 73853.87 (70634.93-77293.5) | 2039534 (1349914-2976956) | 512.95 (346.05-746.38) | 4643 (3605-6301) | 2.65 (2.16-3.37) | 0.16 (0.14-0.17) | 0.12 (0.1-0.13) | 0.04 (0.02-0.06) | -0.39 (-0.6--0.19) |
| Commonwealth Middle Income | 261660609 (249129579-274499222) | 22857.58 (21980.44-23781.3) | 729049553 (694028760-769272235) | 65210.27 (62404.23-68240.13) | 6065424 (4119580-8557616) | 499.14 (342.02-701.65) | 15938 (13665-17937) | 2.23 (1.87-2.57) | 500706688 (481780507-519881724) | 24296.31 (23440.2-25196.99) | 1375830679 (1318379249-1438423981) | 67733.5 (64937.91-70713.98) | 10360957 (6947864-14799458) | 496.08 (335.96-706.71) | 26866 (24119-29868) | 1.89 (1.68-2.1) | 0.23 (0.19-0.26) | 0.16 (0.11-0.2) | -0.01 (-0.06-0.03) | -0.74 (-0.89--0.59) |
| East Asia | 272520330 (263648230-281675845) | 22770.49 (22059.85-23480.6) | 571746764 (541514847-605718095) | 48491.46 (45991.87-51165.19) | 6451671 (4256967-9317286) | 531.82 (352.1-766.59) | 7105 (6218-8061) | 1.3 (1.12-1.5) | 378585061 (368583208-388914877) | 24841.08 (24114.77-25545.32) | 781862178 (740979492-821530394) | 50175.29 (47664.49-52836.27) | 7753521 (5013710-11387131) | 546.92 (353.7-799.02) | 7535 (6348-8873) | 0.45 (0.37-0.53) | 0.3 (0.29-0.31) | 0.11 (0.1-0.12) | 0.08 (0.07-0.09) | -4.16 (-4.55--3.77) |
| East Asia & Pacific - WB | 445141394 (430784867-460243256) | 24376.45 (23623.17-25133.74) | 915542100 (870138140-966825380) | 51055.43 (48597.84-53812.3) | 10277715 (6738223-14806094) | 550.7 (361.01-792.65) | 13666 (11817-16424) | 1.32 (1.13-1.61) | 647702856 (628233719-666653652) | 26404.86 (25612.43-27190.87) | 1335958939 (1269485099-1405235470) | 53556.47 (50986.52-56436.81) | 13275072 (8637722-19234902) | 572.82 (371.9-829.81) | 28890 (25573-31293) | 0.99 (0.87-1.07) | 0.27 (0.27-0.28) | 0.16 (0.16-0.16) | 0.13 (0.13-0.14) | -1.15 (-1.28--1.02) |
| Eastern Africa | 54918959 (51150392-58851166) | 30622.66 (29188.34-32211.08) | 141455234 (133219896-150673140) | 82347.43 (78387.76-86761.95) | 1091575 (744988-1584215) | 582.39 (400.79-850.51) | 3242 (2235-4729) | 3.56 (2.37-6.2) | 116956799 (110640227-123968778) | 30324.22 (29002.61-31816.29) | 307309833 (290430408-325849895) | 82604.04 (78693.7-86960.49) | 2255662 (1494262-3307481) | 567.46 (384.72-831.06) | 5367 (3882-7603) | 3.24 (2.42-4.56) | -0.06 (-0.08--0.05) | -0.03 (-0.05--0.01) | -0.12 (-0.15--0.09) | -0.35 (-0.4--0.29) |
| Eastern Europe | 48648326 (47343198-50257957) | 20964.98 (20398.26-21615.72) | 112558239 (107188172-118172733) | 47303.81 (45168.33-49472.53) | 971141 (640618-1371735) | 444.04 (292.27-627.68) | 1298 (1245-1343) | 0.49 (0.47-0.51) | 48644573 (47367394-50165984) | 21582.16 (21022.29-22241.83) | 116168826 (110309311-122360542) | 47888.39 (45734.78-50016.18) | 974056 (671140-1349426) | 488.61 (331.84-681.33) | 4576 (4236-4929) | 1.4 (1.3-1.5) | 0.15 (0.11-0.19) | 0.07 (0.05-0.1) | 0.31 (0.28-0.34) | 2.69 (2.25-3.12) |
| Eastern Mediterranean Region | 68710761 (66527939-70923368) | 19308.59 (18742.16-19867.93) | 160933755 (154982070-167680458) | 46585.43 (44876.38-48386.08) | 1597792 (1063292-2270610) | 407.89 (271.23-577.49) | 1816 (1418-2269) | 1.05 (0.78-1.44) | 146741181 (142108510-151346484) | 20468.59 (19886.92-21060.91) | 341398873 (329095827-354413314) | 48367.83 (46586.55-50147.99) | 3233606 (2145527-4588900) | 432.02 (287.35-612.24) | 4214 (3699-4878) | 1.17 (1.03-1.36) | 0.22 (0.21-0.23) | 0.14 (0.13-0.15) | 0.21 (0.2-0.22) | 0.47 (0.42-0.51) |
| Eastern Sub-Saharan Africa | 62027244 (57622500-66622600) | 31782.75 (30295.92-33490.85) | 163008899 (153389378-173638262) | 87348.31 (83055.35-92093.42) | 1230729 (844420-1790340) | 600.02 (413.46-878.38) | 3877 (2768-5380) | 3.85 (2.65-6.53) | 133302707 (125699887-141646870) | 31366.99 (29978.46-32970.47) | 357055983 (337452353-378691275) | 87336.81 (83281.41-92012.18) | 2539974 (1674399-3732891) | 578.69 (391.25-848.9) | 6093 (4428-8473) | 3.4 (2.6-4.7) | -0.08 (-0.09--0.07) | -0.04 (-0.06--0.03) | -0.16 (-0.18--0.13) | -0.49 (-0.54--0.44) |
| Europe | 199842574 (194826797-205541357) | 23932.67 (23310.82-24585.6) | 413797168 (392916627-434605524) | 48083.63 (45842.26-50359.89) | 4161383 (2772193-5881451) | 526.91 (350.56-741.8) | 7719 (7102-8094) | 0.83 (0.76-0.88) | 239235401 (233329478-245918109) | 25187.02 (24576.18-25819.25) | 506462406 (479933597-534462001) | 49171.32 (46951.18-51428.52) | 4784813 (3262578-6658604) | 564.16 (378.35-792.05) | 21265 (18490-22786) | 1.22 (1.08-1.3) | 0.2 (0.18-0.21) | 0.09 (0.08-0.09) | 0.24 (0.22-0.25) | 1.09 (0.89-1.29) |
| Europe & Central Asia - WB | 209886672 (204625497-215843952) | 23845.61 (23223.55-24496.63) | 432448963 (411181621-453985059) | 47822.89 (45588.09-50113.83) | 4420879 (2943244-6239949) | 527.74 (350.48-745.54) | 7741 (7124-8118) | 0.81 (0.74-0.85) | 254541978 (248213184-261519578) | 24979.33 (24371.92-25616.09) | 536002462 (507950903-564977036) | 48741.14 (46547.21-50994.47) | 5155238 (3496056-7202551) | 563.37 (377.49-793.73) | 21348 (18577-22874) | 1.19 (1.06-1.27) | 0.18 (0.17-0.19) | 0.08 (0.07-0.08) | 0.22 (0.21-0.23) | 1.12 (0.92-1.32) |
| European Region | 211824182 (206523482-217829399) | 23867.85 (23245.37-24517.66) | 436120833 (414708382-457791180) | 47848.32 (45613.71-50133.16) | 4461654 (2970969-6297788) | 527.9 (350.67-745.71) | 7901 (7271-8285) | 0.82 (0.75-0.86) | 258220844 (251834494-265250567) | 25027.17 (24422.67-25662.09) | 543007391 (514684419-572366822) | 48798.85 (46599.68-51047.77) | 5233903 (3551414-7310073) | 563.84 (377.97-794.1) | 22136 (19237-23738) | 1.22 (1.08-1.3) | 0.18 (0.17-0.19) | 0.08 (0.07-0.08) | 0.22 (0.21-0.23) | 1.16 (0.98-1.35) |
| High-income Asia Pacific | 46130703 (45063387-47403211) | 26263.75 (25668.85-26955.24) | 73609890 (69640479-77837598) | 40899.08 (38846.02-43149.7) | 1006429 (648131-1442007) | 607.81 (390.36-882.72) | 739 (658-796) | 0.45 (0.39-0.48) | 60027880 (58712254-61543392) | 27700.46 (27104.4-28372.33) | 109153762 (102216698-116595917) | 42360.87 (40350.08-44630.68) | 1089583 (705963-1554152) | 629.54 (402.36-912.19) | 3503 (2839-3913) | 0.58 (0.49-0.64) | 0.18 (0.16-0.2) | 0.12 (0.11-0.12) | 0.12 (0.11-0.13) | 1.03 (0.69-1.37) |
| High-income North America | 71429290 (70416074-72562457) | 24570.69 (24204.52-25001.43) | 108586328 (105031648-112268819) | 36258.78 (35139.3-37473.54) | 1767937 (1206897-2459029) | 629.69 (426.52-882.17) | 3137 (2811-3318) | 0.86 (0.78-0.91) | 104996769 (103843518-106187305) | 25505.59 (25224.8-25810.67) | 160600824 (156269731-164615111) | 36676.18 (35792.48-37564.64) | 2412642 (1675356-3283744) | 634.71 (433.62-880.36) | 7668 (6589-8657) | 1.14 (0.99-1.28) | 0.13 (0.12-0.14) | 0.11 (0.08-0.14) | 0.08 (0.06-0.1) | 1.57 (1.22-1.92) |
| Latin America & Caribbean - WB | 104952754 (101648598-108421088) | 25200.59 (24456.7-25966.47) | 229165649 (218208554-242145905) | 56877.26 (54242.95-59820.1) | 2423718 (1595002-3478932) | 550.81 (363.73-787.51) | 4368 (4136-4527) | 1.78 (1.67-1.86) | 173573774 (168559476-178527652) | 26150.94 (25388.65-26901.37) | 389747209 (371954002-409311083) | 58476.23 (55778.07-61425.43) | 3907320 (2690008-5474539) | 599.53 (411.03-841.6) | 22182 (19522-24734) | 3.25 (2.86-3.62) | 0.13 (0.12-0.14) | 0.09 (0.07-0.11) | 0.32 (0.3-0.35) | 2.39 (2.16-2.61) |
| Limited Health System | 362052809 (343947175-379989868) | 23508.36 (22615.1-24475.01) | 984828738 (936195628-1039138184) | 65747.28 (62903.16-68809.36) | 8232476 (5609645-11662000) | 502.78 (345.24-711.01) | 20514 (17454-23582) | 2.26 (1.88-2.71) | 701049798 (672181114-730536677) | 24852.19 (23957.91-25808.96) | 1894394493 (1814116357-1984275057) | 68390.47 (65547.69-71421.49) | 14384822 (9595377-20637494) | 499.35 (337.38-715.13) | 33327 (30121-36886) | 1.86 (1.68-2.04) | 0.2 (0.17-0.22) | 0.14 (0.11-0.18) | -0.03 (-0.07-0) | -0.93 (-1.09--0.77) |
| Middle East & North Africa - WB | 44122841 (42956434-45482741) | 18563.73 (18096.43-19065.65) | 90434111 (87554179-93546065) | 39516.67 (38143.56-40933.68) | 1020422 (681476-1454298) | 391.26 (261.94-550.55) | 1126 (909-1519) | 1.21 (0.94-1.71) | 88714434 (86316461-91290118) | 19561.67 (19063.13-20091.42) | 179814838 (173743388-186477920) | 40213.61 (38777.66-41635.83) | 1924038 (1288456-2702784) | 417.98 (281.22-585.82) | 3763 (3310-4315) | 1.54 (1.33-1.75) | 0.21 (0.2-0.22) | 0.08 (0.07-0.09) | 0.24 (0.23-0.25) | 0.93 (0.74-1.13) |
| Minimal Health System | 36721548 (33819354-39903590) | 27135.81 (25694.44-28683.05) | 98996365 (93065166-105874185) | 76650.3 (73012-80702.3) | 665410 (448144-970692) | 461.21 (308.34-670.25) | 1623 (1283-1995) | 2.03 (1.53-2.66) | 88290353 (82388768-95099139) | 26653.18 (25385.43-28034.55) | 240850009 (227494795-256847272) | 76489.95 (72930.81-80617.5) | 1591607 (1046995-2335529) | 455.63 (303.49-665.67) | 2760 (2017-3863) | 1.92 (1.48-2.57) | -0.05 (-0.07--0.03) | 0.01 (-0.01-0.02) | -0.02 (-0.04--0.01) | -0.19 (-0.24--0.15) |
| North Africa and Middle East | 57843157 (56231805-59634926) | 18302.31 (17836.85-18815.15) | 120570158 (116703436-124560766) | 39643.65 (38217.52-41029.45) | 1346877 (898117-1917656) | 387.94 (258.13-548.27) | 1232 (967-1684) | 0.9 (0.68-1.31) | 115174776 (111980118-118582526) | 19306.3 (18807.36-19840.1) | 236277686 (228062970-245033446) | 40218.77 (38825.69-41630.55) | 2521094 (1683311-3545537) | 412.8 (276.01-579.97) | 3743 (3260-4402) | 1.06 (0.92-1.25) | 0.21 (0.2-0.22) | 0.07 (0.06-0.08) | 0.23 (0.22-0.24) | 0.75 (0.65-0.86) |
| North America | 71431535 (70418328-72564645) | 24571.4 (24205.2-25002.18) | 108602796 (105047214-112284900) | 36264.15 (35144.26-37479.09) | 1767908 (1206859-2458998) | 629.68 (426.51-882.16) | 3137 (2812-3318) | 0.86 (0.78-0.91) | 105001294 (103847694-106191924) | 25506.05 (25225.24-25811.21) | 160625460 (156293105-164641508) | 36680.42 (35796.6-37569.07) | 2412652 (1675349-3283773) | 634.7 (433.62-880.35) | 7670 (6591-8659) | 1.14 (0.99-1.28) | 0.13 (0.12-0.14) | 0.11 (0.08-0.14) | 0.08 (0.06-0.1) | 1.57 (1.22-1.92) |
| Northern Africa | 20730056 (20136234-21419514) | 18329.97 (17853.29-18841.76) | 44751722 (43336074-46273633) | 40773.78 (39373.95-42185.43) | 463195 (303618-661094) | 375.84 (250.39-530.52) | 401 (296-608) | 0.89 (0.62-1.44) | 38600791 (37507942-39768051) | 19192.44 (18674.29-19727.79) | 81893022 (79228249-84851851) | 41310.69 (39904.25-42747.92) | 822701 (543858-1159033) | 398.25 (265.37-559.32) | 1260 (1028-1614) | 1.17 (0.97-1.51) | 0.18 (0.16-0.19) | 0.07 (0.05-0.08) | 0.21 (0.2-0.22) | 1.38 (1.23-1.52) |
| Oceania | 1641491 (1574439-1716040) | 25993.21 (25100.9-26973.88) | 3858091 (3626424-4127479) | 62423.13 (58905.64-66191.45) | 43325 (28686-62899) | 656.36 (439-939.91) | 93 (59-153) | 3.01 (1.84-5.3) | 3547417 (3417504-3696044) | 26417.21 (25516.6-27376.99) | 8330362 (7859155-8895278) | 62864.31 (59461.07-66512.16) | 92567 (62260-133148) | 668.61 (461.67-953.84) | 233 (151-362) | 3.04 (1.99-4.87) | 0.04 (0.03-0.05) | 0.01 (0-0.03) | 0.06 (0.04-0.08) | 0.08 (0.05-0.11) |
| Region of the Americas | 175382512 (171281394-179646352) | 24958.55 (24384.56-25534.56) | 335510516 (322254650-349827678) | 48203.49 (46287.76-50182.78) | 4170399 (2789797-5904420) | 584.02 (391.08-823.76) | 7419 (6877-7726) | 1.24 (1.14-1.29) | 277466104 (271522544-283275652) | 25917.86 (25342.53-26485.36) | 547766076 (526863101-569507055) | 50484.71 (48545.78-52523.94) | 6296149 (4352641-8729409) | 608.68 (417.49-848.49) | 29514 (25822-32962) | 2.2 (1.93-2.45) | 0.13 (0.12-0.14) | 0.17 (0.16-0.19) | 0.19 (0.17-0.21) | 2.4 (2.19-2.6) |
| South-East Asia Region | 291206401 (278849767-303761349) | 22990.98 (22099.88-23880.22) | 759262440 (722756334-801312362) | 61334.55 (58644.89-64203.62) | 6985140 (4749061-9870493) | 523.63 (359.43-737.87) | 17920 (15264-20419) | 2.41 (2.02-2.84) | 489331386 (472426454-506069022) | 24053.87 (23237.59-24901.62) | 1272509436 (1218465732-1328413698) | 63067.87 (60466.24-65896.35) | 10513684 (7094011-14988302) | 521.56 (354.26-742.38) | 32656 (29528-35837) | 2.12 (1.91-2.33) | 0.17 (0.14-0.21) | 0.13 (0.08-0.17) | -0.02 (-0.06-0.03) | -0.69 (-0.85--0.53) |
| South Asia | 229008701 (218356978-240286425) | 21387.43 (20580.99-22246.19) | 644280575 (612369480-680697573) | 61807.38 (59160.8-64638.03) | 5642059 (3844386-7911936) | 495.45 (342.14-692.96) | 15061 (12951-16935) | 2.33 (1.94-2.67) | 408436790 (394178246-422330208) | 22596.96 (21827.66-23378.25) | 1137040755 (1091353105-1184975257) | 63675.55 (61116.27-66304.83) | 8888988 (5947710-12680113) | 489.15 (330.58-696.18) | 23122 (20338-26018) | 1.79 (1.57-2.03) | 0.21 (0.17-0.25) | 0.13 (0.08-0.18) | -0.05 (-0.1-0) | -1.23 (-1.44--1.03) |
| South Asia - WB | 235604518 (224821610-246973257) | 21481.82 (20684.55-22332.33) | 657768197 (625380928-694806444) | 61571.49 (58967.41-64384.53) | 5777741 (3934783-8111970) | 495.34 (341.75-693.5) | 15201 (13064-17082) | 2.29 (1.91-2.63) | 420525535 (406127078-434633783) | 22598.76 (21834.7-23369.05) | 1161645288 (1115540534-1210294658) | 63203.16 (60712.05-65804.23) | 9144516 (6110363-13045001) | 488.2 (329.73-694.86) | 23488 (20722-26395) | 1.77 (1.55-2) | 0.2 (0.16-0.24) | 0.13 (0.08-0.17) | -0.05 (-0.1-0) | -1.19 (-1.39--0.99) |
| Southeast Asia | 124410213 (118964388-130441527) | 27805.54 (26651.09-29014.65) | 264238420 (248768104-283563364) | 60484.29 (57196.38-64490.2) | 2766041 (1820834-3999355) | 593.22 (397.23-855.62) | 5633 (4489-7657) | 2.42 (1.87-3.45) | 203100121 (194920364-211703443) | 29118.55 (27958.35-30293.31) | 429388588 (406181212-457315842) | 61774.87 (58486.42-65776.73) | 4290673 (2884568-6136642) | 628.54 (424.01-897.52) | 16942 (15069-18848) | 3.19 (2.81-3.55) | 0.16 (0.15-0.16) | 0.08 (0.08-0.08) | 0.22 (0.2-0.24) | 1.14 (0.98-1.3) |
| Southern Africa | 24046120 (22766435-25423101) | 26705.22 (25595.79-27901.04) | 69302058 (65811633-72864052) | 79393.41 (75725.82-83070.11) | 487600 (338013-695372) | 508.64 (352.28-727.84) | 1717 (1490-2013) | 3.68 (3.01-4.65) | 48808256 (46278226-51534839) | 27627.53 (26485.46-28815.43) | 138730659 (132073297-145944504) | 80968.14 (77380.85-84868.12) | 939314 (639974-1355441) | 521.43 (362.61-742.48) | 3273 (2778-3832) | 3.88 (3.46-4.36) | 0.13 (0.12-0.14) | 0.07 (0.06-0.08) | 0.1 (0.06-0.13) | 0.11 (-0.17-0.4) |
| Southern Latin America | 11858676 (11533547-12191086) | 24110.84 (23494.07-24761.32) | 22454000 (21464568-23526167) | 46320.61 (44237.38-48529.65) | 273708 (181604-389069) | 549.27 (365.41-778.2) | 536 (493-582) | 1.29 (1.18-1.4) | 18007774 (17594172-18446983) | 25532.26 (24914.07-26182.92) | 35085965 (33471645-36777120) | 48109.32 (46132.49-50269.65) | 435327 (310324-590230) | 632.73 (442.36-870.65) | 4254 (3845-4556) | 4.69 (4.25-5.01) | 0.2 (0.19-0.21) | 0.13 (0.13-0.14) | 0.53 (0.49-0.57) | 5.08 (4.47-5.69) |
| Southern Sub-Saharan Africa | 12315389 (11751860-12902336) | 25147.74 (24154.8-26168.38) | 37390102 (35654884-39217692) | 77772.14 (74291.94-81460.43) | 242619 (166193-346575) | 473.95 (327.03-674.15) | 884 (718-1141) | 3.64 (2.91-4.79) | 19737224 (18914422-20592062) | 25707.34 (24719.56-26706.79) | 59802488 (57231598-62547292) | 78608.83 (75156.45-82085.23) | 377025 (260607-533231) | 492.87 (344.43-694.7) | 1889 (1672-2171) | 4.03 (3.61-4.57) | 0.08 (0.07-0.09) | 0.04 (0.03-0.04) | 0.13 (0.06-0.19) | 0.25 (-0.13-0.64) |
| Sub-Saharan Africa - WB | 148504408 (138642592-158966115) | 29403.87 (27956.81-31025.38) | 402702359 (380560727-428379103) | 82657.46 (78776.37-86865.44) | 2704262 (1810074-3979762) | 503.4 (333.83-743.98) | 6299 (5015-7988) | 2.3 (1.77-3.27) | 339737080 (320172563-362466278) | 29514.95 (28154.66-31043.83) | 925319800 (876168664-980999791) | 83334.1 (79451.34-87572.49) | 6082704 (3995835-8942274) | 506.4 (337.42-745.48) | 11654 (9139-14954) | 2.26 (1.86-2.79) | 0 (0-0.01) | 0.02 (0.01-0.03) | 0.02 (0.01-0.03) | -0.09 (-0.17--0.01) |
| Tropical Latin America | 39931212 (38442602-41494174) | 27649.29 (26756.65-28689.45) | 93757916 (88955831-99784879) | 67062.03 (63792.03-70863.13) | 915860 (590391-1334256) | 602.35 (392.19-877.99) | 1452 (1362-1517) | 1.74 (1.6-1.84) | 64831485 (62775492-67061194) | 28276.93 (27388.37-29266.64) | 157134868 (149370897-165557351) | 67852.33 (64588.58-71628.39) | 1472179 (1005626-2098254) | 658.49 (445.66-940.12) | 9884 (8661-10987) | 4.02 (3.51-4.47) | 0.06 (0.04-0.09) | 0.01 (-0.03-0.05) | 0.29 (0.25-0.34) | 2.99 (2.72-3.25) |
| Western Africa | 51000216 (47575587-54760678) | 30484.94 (28866.22-32265) | 141272776 (132951642-149870808) | 86129.48 (81894.77-90616.12) | 785820 (498055-1200573) | 435.14 (275.05-668.58) | 311 (249-400) | 0.25 (0.19-0.34) | 130463254 (122087123-139975140) | 31084.9 (29478.5-32853.56) | 355723475 (335721404-377905822) | 87323.1 (83124.16-91823.75) | 2099916 (1347898-3163827) | 464.04 (294.69-708.07) | 1333 (1013-1741) | 0.5 (0.39-0.65) | 0.06 (0.05-0.07) | 0.05 (0.04-0.07) | 0.26 (0.24-0.27) | 3.05 (2.63-3.48) |
| Western Europe | 113021894 (110265071-116024067) | 27663.33 (26997.12-28409.68) | 216804260 (204759960-229084043) | 50096.88 (47608.3-52625.8) | 2390672 (1618606-3347611) | 627.88 (422.09-887.06) | 5971 (5374-6314) | 1.04 (0.93-1.1) | 144725394 (141248234-148638143) | 29038.12 (28365.47-29769.1) | 286360314 (269569379-303894329) | 51594.81 (49085.75-54155.92) | 2935720 (2028590-4081413) | 657.82 (443.63-925.26) | 15654 (13046-17045) | 1.3 (1.11-1.41) | 0.15 (0.14-0.17) | 0.09 (0.08-0.1) | 0.14 (0.12-0.15) | 0.74 (0.6-0.88) |
| Western Pacific Region | 359406376 (348392176-370795873) | 23785.47 (23090.16-24482.83) | 734156871 (696721062-775657394) | 49410.63 (47040.24-52021.6) | 8355826 (5474436-12029659) | 542.73 (355.53-780.79) | 9746 (8427-11281) | 1.15 (0.99-1.36) | 516111692 (502040473-529897610) | 25815.37 (25096.56-26532.73) | 1057908424 (1005529665-1110419340) | 51795.93 (49328.37-54517.74) | 10499513 (6788327-15279361) | 562.23 (363.34-817.07) | 17654 (15492-19541) | 0.7 (0.61-0.77) | 0.28 (0.28-0.28) | 0.15 (0.15-0.16) | 0.12 (0.11-0.13) | -1.9 (-2.06--1.75) |
| Western Sub-Saharan Africa | 55738233 (51988885-59855690) | 30109.89 (28502.72-31860.38) | 155048504 (145937326-164551028) | 85498.76 (81310.58-89930.51) | 861804 (546203-1316653) | 430.48 (272.36-661.32) | 323 (261-416) | 0.24 (0.18-0.32) | 144112882 (135093086-154365165) | 30588.89 (29055.46-32264.17) | 395396251 (373539497-419617478) | 86517.85 (82323.52-90991.25) | 2331651 (1495672-3505882) | 457.42 (290.11-697.9) | 1413 (1075-1856) | 0.46 (0.36-0.6) | 0.05 (0.04-0.06) | 0.05 (0.03-0.06) | 0.24 (0.23-0.26) | 3.01 (2.59-3.43) |
| World Bank High Income | 272320246 (266515305-278108296) | 25502.56 (24952.98-26044.22) | 489612018 (467373903-511341751) | 44375.37 (42570.34-46184.27) | 5897777 (3946911-8244564) | 595.07 (395.72-837.92) | 11640 (10571-12274) | 0.94 (0.85-0.99) | 369762598 (363011466-377048396) | 26526.94 (26032.13-27026.85) | 687122216 (656287327-720507516) | 45111.57 (43374.22-46809.53) | 7495357 (5112973-10410110) | 611.21 (409.29-854.19) | 32622 (27738-35280) | 1.19 (1.04-1.28) | 0.13 (0.13-0.14) | 0.07 (0.07-0.08) | 0.1 (0.1-0.11) | 0.94 (0.8-1.07) |
| World Bank Low Income | 86429208 (80195140-92849493) | 27662.84 (26305.94-29130.27) | 219387319 (207206209-233817835) | 73192.8 (69729.55-77064.57) | 1757480 (1174393-2564219) | 497.54 (333.72-726.64) | 4085 (3198-5151) | 2.11 (1.59-2.94) | 187900269 (176110523-200375430) | 27382.51 (26119.02-28770.97) | 490608126 (464765530-520439729) | 74050.9 (70724.21-77915.56) | 3702898 (2426808-5418929) | 488.07 (324.53-714.59) | 6401 (4710-8525) | 1.82 (1.4-2.36) | -0.04 (-0.06--0.03) | 0.02 (0.01-0.04) | -0.08 (-0.1--0.06) | -0.6 (-0.67--0.53) |
| World Bank Lower Middle Income | 450203143 (431211012-468835022) | 23300.61 (22446.06-24179.1) | 1157540660 (1104852703-1218897225) | 61357.53 (58736.09-64104.94) | 9986336 (6737374-14158909) | 501.74 (341.33-710.84) | 22208 (18756-25764) | 1.97 (1.64-2.39) | 825198375 (794967173-855919290) | 24634.3 (23759.83-25524.91) | 2116747164 (2026050347-2212006276) | 63968.77 (61290.85-66712.11) | 16626968 (11077977-23805271) | 507.47 (341.95-724.81) | 40792 (37509-44524) | 1.88 (1.71-2.04) | 0.2 (0.18-0.22) | 0.16 (0.13-0.19) | 0.04 (0.01-0.07) | -0.32 (-0.44--0.2) |
| World Bank Upper Middle Income | 451083724 (437411117-464730152) | 23208.07 (22532.48-23920.67) | 970986610 (924597936-1022501747) | 51041.45 (48722.18-53608.4) | 10756005 (7035415-15485556) | 520.02 (340.11-748.09) | 13605 (12335-15208) | 1.14 (1.02-1.29) | 647416300 (629661673-664488330) | 24743.53 (24052.65-25428.3) | 1395731861 (1330539191-1463290413) | 52415.95 (50050.98-54977.89) | 14101521 (9274833-20252608) | 546.73 (359.31-785.9) | 39181 (34897-43000) | 1.26 (1.11-1.38) | 0.23 (0.22-0.23) | 0.09 (0.08-0.1) | 0.17 (0.17-0.18) | 0.28 (0.09-0.47) |
| Country |  |  |  |  |  |  |  |  |  |  |  |  |  |  |  |  |  |  |  |  |
| Afghanistan | 1639598 (1587960-1697557) | 16964.56 (16482.03-17453.72) | 3468740 (3350346-3599634) | 37684.33 (36222.39-39084.23) | 38882 (26182-55101) | 372.5 (253.08-525.12) | 79 (52-116) | 1.35 (0.83-2.2) | 4988566 (4816994-5177600) | 17368.6 (16880.4-17907.44) | 10229568 (9900626-10629287) | 37888.71 (36463.31-39367.81) | 124136 (82923-175992) | 391.59 (268.73-544.18) | 148 (106-193) | 1.79 (1.22-2.61) | 0.13 (0.09-0.17) | 0.05 (0.03-0.07) | 0.22 (0.19-0.25) | 1.24 (1.1-1.39) |
| Albania | 602614 (584336-622233) | 19364.78 (18766.15-20051.7) | 1297560 (1235476-1366555) | 43500.07 (41266-45871.64) | 13762 (9078-19678) | 407.7 (268.05-585.06) | 9 (7-11) | 0.35 (0.27-0.43) | 599526 (581158-620296) | 20466.87 (19900.57-21152.73) | 1380045 (1303196-1459689) | 44689.59 (42476.07-47019.4) | 10702 (6939-15399) | 416.96 (269.78-597.83) | 7 (5-9) | 0.18 (0.13-0.24) | 0.22 (0.21-0.24) | 0.11 (0.1-0.12) | 0.11 (0.09-0.13) | -1.77 (-2.25--1.29) |
| Algeria | 4339053 (4201341-4485423) | 18450.86 (17937.01-18997.97) | 8817090 (8510342-9128798) | 39161.07 (37720.24-40661.36) | 99474 (65623-142761) | 380.42 (252.22-539.39) | 59 (44-84) | 0.71 (0.53-0.99) | 8137351 (7896264-8374313) | 19212.37 (18631.37-19774.5) | 16682799 (16076416-17303032) | 39589 (38133.46-41071.26) | 175363 (117896-246063) | 408.76 (276.89-571.37) | 281 (218-372) | 1.2 (0.95-1.53) | 0.18 (0.16-0.19) | 0.07 (0.06-0.08) | 0.27 (0.26-0.29) | 2.47 (2.17-2.77) |
| American Samoa | 12765 (12266-13283) | 27611.21 (26697.86-28555.17) | 29233 (27601-31167) | 64099.68 (60747.06-67719.19) | 359 (253-513) | 787.07 (578.16-1092.29) | 2 (1-2) | 6.95 (5.55-8.81) | 13894 (13427-14375) | 27638.89 (26730.27-28550.25) | 31897 (30119-33820) | 64213.59 (60797.67-67940.74) | 440 (328-588) | 885.21 (662.14-1178.64) | 4 (4-6) | 10.24 (8.22-12.71) | -0.03 (-0.06--0.01) | -0.07 (-0.12--0.02) | 0.54 (0.46-0.62) | 1.97 (1.73-2.21) |
| Andorra | 15307 (14877-15759) | 27914.46 (27154.65-28726.13) | 27897 (26402-29432) | 49874.04 (47200.76-52551.42) | 319 (213-450) | 602 (399.05-851.84) | 0 (0-0) | 0.14 (0.1-0.19) | 27549 (26810-28331) | 29080.79 (28332.67-29889.69) | 52636 (49656-55820) | 51021.49 (48447.04-53722.02) | 517 (349-734) | 623.77 (415.93-885.75) | 0 (0-0) | 0.1 (0.07-0.14) | 0.15 (0.14-0.17) | 0.09 (0.08-0.1) | 0.13 (0.12-0.14) | -0.58 (-0.77--0.39) |
| Angola | 2852736 (2526767-3218050) | 27042.31 (24870.08-29420.99) | 7553054 (7054467-8189216) | 76984.36 (73114.47-81673.79) | 58640 (41999-82708) | 534.08 (384.43-739.67) | 260 (195-330) | 4.57 (3.66-5.71) | 8824814 (7932666-9878374) | 26662.17 (24880.7-28672.97) | 23998898 (22415414-25988619) | 77702.36 (74019.44-82111.01) | 168424 (115790-244409) | 517 (370.54-722.03) | 556 (412-749) | 4.38 (3.31-5.56) | -0.03 (-0.04--0.01) | 0.05 (0.03-0.08) | -0.09 (-0.1--0.08) | -0.13 (-0.17--0.09) |
| Antigua and Barbuda | 15530 (15001-16033) | 26167.21 (25303.91-27006.19) | 35062 (33304-37088) | 59728.94 (56840.18-63270.12) | 356 (243-504) | 592.24 (406.53-835.4) | 2 (2-3) | 4.18 (3.65-4.74) | 24760 (23995-25551) | 27050.01 (26196.12-27895.25) | 56134 (53239-59658) | 60633.75 (57666.01-64234.52) | 614 (446-826) | 695.13 (498.99-941.66) | 8 (7-8) | 8.82 (8.05-9.48) | 0.1 (0.09-0.11) | 0.04 (0.04-0.05) | 0.56 (0.47-0.65) | 2.6 (2.14-3.06) |
| Argentina | 8015359 (7802317-8241636) | 24226.27 (23602.11-24886.68) | 15768697 (15090822-16483552) | 48217.11 (46119.67-50436.41) | 183737 (121518-260862) | 549.3 (363.76-778.9) | 346 (315-382) | 1.19 (1.08-1.31) | 12010014 (11722835-12301528) | 25504.85 (24889.79-26175.5) | 24182342 (23146745-25264926) | 50042.5 (48061.9-52195.58) | 300015 (215287-405112) | 647.55 (456.57-885.24) | 3153 (2856-3400) | 5.44 (4.94-5.86) | 0.18 (0.17-0.19) | 0.13 (0.12-0.14) | 0.63 (0.59-0.67) | 6.08 (5.42-6.73) |
| Armenia | 702349 (681902-726501) | 21189.16 (20548.25-21911.66) | 1394601 (1327669-1470780) | 43304.56 (41149.11-45630.72) | 16909 (10639-25103) | 488.61 (308.59-719.07) | 3 (2-4) | 0.11 (0.08-0.13) | 682224 (661820-705953) | 21936.16 (21250.02-22663.22) | 1468860 (1389691-1549029) | 44125.05 (41990.42-46374.25) | 14110 (9056-20184) | 509.17 (321.63-740.74) | 11 (8-15) | 0.27 (0.2-0.36) | 0.18 (0.14-0.21) | 0.1 (0.08-0.12) | 0.19 (0.17-0.21) | 4.78 (3.81-5.75) |
| Australia | 4608081 (4525915-4696530) | 26605.19 (26105.97-27131.35) | 10552420 (10076210-10994123) | 60206.03 (57624.33-62669.11) | 88442 (58173-126911) | 532.57 (348.85-760.88) | 129 (115-139) | 0.73 (0.65-0.78) | 8095762 (7971463-8219410) | 27982.11 (27485.31-28525.53) | 18601506 (17788443-19518056) | 61895.65 (59252.67-64392.89) | 149264 (100217-212221) | 568.13 (375.31-804.95) | 799 (677-890) | 1.5 (1.28-1.66) | 0.18 (0.17-0.2) | 0.11 (0.1-0.11) | 0.24 (0.22-0.25) | 2.36 (2.04-2.67) |
| Austria | 2243284 (2180975-2307987) | 27076.32 (26325.14-27845.6) | 4476724 (4217175-4725017) | 51001.18 (48392.59-53641.49) | 44926 (30145-63260) | 588.62 (389.83-827.3) | 31 (28-34) | 0.26 (0.23-0.28) | 2900242 (2825592-2980472) | 28557.87 (27800.91-29359.06) | 5865824 (5525283-6217434) | 52553.45 (49935.14-55184.65) | 55252 (37305-77745) | 619.4 (411.69-868.38) | 115 (96-127) | 0.5 (0.42-0.55) | 0.19 (0.17-0.2) | 0.11 (0.1-0.12) | 0.17 (0.17-0.18) | 2.27 (1.84-2.7) |
| Azerbaijan | 1530115 (1485538-1582549) | 21689.86 (21032.53-22413.75) | 2887353 (2749778-3044377) | 43169.03 (40980.44-45408.52) | 37789 (23708-56564) | 495.54 (313.07-730.86) | 4 (3-6) | 0.07 (0.04-0.1) | 2279185 (2209158-2355062) | 22353.15 (21680.47-23065.12) | 4566825 (4356494-4815456) | 43995.75 (41850.22-46241.55) | 49388 (32016-71264) | 506.05 (323.59-738.43) | 6 (4-9) | 0.06 (0.04-0.1) | 0.18 (0.12-0.24) | 0.11 (0.08-0.15) | 0.11 (0.08-0.13) | 0.41 (0.17-0.65) |
| Bahrain | 86035 (83421-88832) | 19326.43 (18837.13-19863.98) | 177507 (171068-184759) | 40449.92 (39000.59-41899.53) | 2392 (1761-3205) | 735.08 (598.41-918.35) | 21 (18-25) | 21.9 (18.2-27.82) | 289798 (281502-299333) | 20311.6 (19779.96-20881.11) | 583367 (560991-608451) | 41529.59 (40038.7-42931.37) | 7360 (5459-9816) | 672.57 (531.49-852.23) | 70 (56-89) | 18.72 (15.43-22.92) | 0.19 (0.18-0.2) | 0.12 (0.1-0.14) | -0.44 (-0.51--0.37) | -0.69 (-0.92--0.47) |
| Bangladesh | 22389413 (21264884-23539737) | 20904.74 (20101.43-21691.39) | 62108910 (59025066-65541207) | 60029.42 (57448.2-62538.56) | 525906 (341268-761330) | 446.54 (292.82-646.18) | 625 (465-778) | 1.82 (1.33-2.33) | 35259524 (34012861-36453598) | 21775.84 (21008.73-22509.14) | 99090494 (94971303-103276446) | 61877.97 (59412.44-64439.89) | 748741 (482098-1099632) | 464.48 (305.25-680.43) | 1509 (1161-1904) | 2.05 (1.55-2.62) | 0.13 (0.11-0.14) | 0.1 (0.09-0.11) | 0.1 (0.09-0.12) | -0.27 (-0.63-0.09) |
| Barbados | 67855 (65625-69972) | 26158.03 (25318.43-26981.29) | 155610 (147553-165156) | 59794.09 (56835.98-63309.91) | 2334 (1884-2931) | 869.11 (692.11-1103.84) | 62 (55-68) | 20.73 (18.32-22.72) | 87409 (84679-90469) | 26360.55 (25499.84-27177.3) | 205919 (193941-219119) | 59938.99 (57014.71-63649.94) | 3036 (2431-3802) | 848.28 (653.04-1094.04) | 88 (72-104) | 17.63 (14.4-20.72) | 0.02 (0.01-0.03) | 0.01 (0-0.01) | -0.08 (-0.15--0.01) | -0.42 (-0.6--0.24) |
| Belarus | 2109143 (2047187-2183342) | 19516.71 (18930.19-20208.51) | 5057848 (4822636-5315948) | 45826.7 (43768.63-48110.44) | 38609 (24955-55489) | 379.55 (246.47-546.92) | 16 (13-19) | 0.13 (0.1-0.15) | 2099093 (2034753-2171412) | 20246.24 (19636.43-20909.99) | 5120905 (4856740-5394982) | 46519.3 (44422.2-48848.53) | 35543 (23258-50989) | 398.19 (258.31-568.39) | 42 (35-51) | 0.28 (0.23-0.34) | 0.18 (0.14-0.21) | 0.08 (0.06-0.11) | 0.18 (0.16-0.2) | 2.14 (1.61-2.68) |
| Belgium | 2877319 (2795379-2964090) | 26971.85 (26210.93-27762.98) | 5453975 (5124186-5777253) | 48124.83 (45567.75-50756.26) | 62374 (43728-85832) | 617.35 (422.38-863.09) | 403 (351-441) | 2.6 (2.25-2.84) | 3706919 (3619874-3808151) | 28472.46 (27716.75-29233.71) | 7142761 (6731681-7594258) | 49681.91 (47127.92-52299.7) | 77182 (54411-105547) | 642.16 (438.48-893.21) | 609 (496-682) | 2.02 (1.69-2.23) | 0.19 (0.18-0.2) | 0.11 (0.1-0.12) | 0.13 (0.11-0.14) | -0.98 (-1.35--0.61) |
| Belize | 43663 (41983-45439) | 24931.22 (24062.35-25800.19) | 98079 (92524-104157) | 58311.9 (55357.25-62019.14) | 1000 (644-1452) | 519.99 (340.26-752) | 1 (1-2) | 1.42 (1.1-1.71) | 105792 (101904-109527) | 25740.66 (24886.52-26564.57) | 236346 (223843-250327) | 59075.46 (56200.36-62671.52) | 2328 (1534-3318) | 555.09 (369.85-789.02) | 6 (5-7) | 2.21 (1.94-2.47) | 0.09 (0.08-0.1) | 0.03 (0.03-0.04) | 0.19 (0.14-0.23) | 1.16 (0.64-1.68) |
| Benin | 1285274 (1173067-1412503) | 27339.34 (25657.47-29126.02) | 3655192 (3410886-3944746) | 80659.56 (76473.08-85068.65) | 19862 (12426-30452) | 385.3 (240.58-593.54) | 4 (3-4) | 0.08 (0.06-0.09) | 3525020 (3255031-3802064) | 27363.71 (25757.68-28964.71) | 10124049 (9488218-10829130) | 81362.61 (77243.77-85911.62) | 57371 (36497-86495) | 400.92 (251.87-611) | 20 (13-31) | 0.14 (0.09-0.21) | 0.01 (0-0.01) | 0.04 (0.03-0.05) | 0.14 (0.14-0.15) | 2.31 (1.99-2.63) |
| Bermuda | 15871 (15361-16397) | 26932.23 (26069.21-27810.7) | 35971 (34138-38114) | 60517.73 (57569.11-64078.46) | 312 (204-450) | 549.59 (359.59-793.89) | 1 (1-1) | 1.51 (1.28-1.73) | 20656 (20028-21351) | 27674.62 (26797.4-28524.35) | 48170 (45327-51317) | 61048.12 (58120.1-64524.14) | 384 (261-552) | 573.83 (378.01-828.71) | 3 (2-4) | 1.97 (1.62-2.39) | 0.11 (0.1-0.12) | 0.04 (0.03-0.05) | 0.15 (0.12-0.17) | 0.74 (0.23-1.26) |
| Bhutan | 128342 (122486-134481) | 20975.09 (20211.71-21752.82) | 354021 (337021-373902) | 60109.66 (57670.41-62636.85) | 3193 (2143-4502) | 472.78 (317.78-667.84) | 6 (4-8) | 1.61 (1.11-2.28) | 163085 (157466-168321) | 22244.87 (21492.93-22994.61) | 456666 (438382-475682) | 62551.49 (60140.92-65148.98) | 3491 (2329-5036) | 476.18 (318.04-687.2) | 7 (5-10) | 1.27 (0.85-1.66) | 0.2 (0.19-0.2) | 0.13 (0.13-0.14) | 0 (-0.01-0.01) | -0.95 (-1.02--0.87) |
| Bolivarian Republic of Venezuela | 4102337 (3968943-4239707) | 23233.68 (22565.89-23917.01) | 8365389 (7983322-8819105) | 49284.76 (46989.36-51722.28) | 95308 (61324-138259) | 493.62 (318.69-714.01) | 23 (21-25) | 0.25 (0.22-0.27) | 6307403 (6121455-6496770) | 23329.09 (22678.53-24017.96) | 13351935 (12699214-14043643) | 49001.83 (46604.7-51482.78) | 132276 (86286-190471) | 503.83 (329.67-727.33) | 93 (71-119) | 0.33 (0.25-0.42) | 0.05 (0.03-0.07) | 0.01 (0-0.02) | 0.08 (0.07-0.09) | 0.75 (0.53-0.97) |
| Bosnia and Herzegovina | 824378 (799006-853897) | 18777.09 (18205.35-19446.44) | 1871220 (1777888-1978372) | 42822.03 (40651.61-45106.6) | 17092 (11053-24586) | 392.77 (254.31-565.29) | 7 (5-11) | 0.19 (0.13-0.29) | 768867 (743136-797035) | 20527.98 (19939.96-21207.49) | 1797081 (1695118-1902436) | 44718.96 (42535.74-46997.71) | 13289 (8711-19178) | 419.01 (271.74-605.67) | 14 (10-19) | 0.23 (0.17-0.32) | 0.33 (0.31-0.36) | 0.16 (0.15-0.18) | 0.24 (0.23-0.26) | 1.11 (0.84-1.38) |
| Botswana | 301830 (286661-318163) | 24733.71 (23724.02-25855.74) | 908965 (865846-956526) | 76378.52 (72817.79-80047.86) | 5703 (3737-8327) | 430.67 (285.46-626.07) | 9 (6-14) | 2.18 (1.44-3.56) | 583151 (558145-610706) | 25932.3 (24931.04-27038.45) | 1738487 (1660043-1823595) | 78061.4 (74542.97-81732.23) | 10323 (6860-14971) | 455.24 (307.42-653.9) | 27 (20-39) | 2.58 (1.93-3.65) | 0.14 (0.13-0.15) | 0.06 (0.06-0.07) | 0.17 (0.14-0.21) | 0.48 (0.18-0.78) |
| Brazil | 38896099 (37448499-40427876) | 27667.85 (26777.73-28708.8) | 91354125 (86686169-97238990) | 67132.43 (63846.07-70933.78) | 891821 (574906-1298840) | 603.19 (392.91-879.05) | 1429 (1341-1496) | 1.77 (1.62-1.87) | 62908979 (60919339-65068881) | 28294.34 (27406.04-29286.72) | 152684032 (145071329-160913886) | 67936.41 (64656.8-71724.44) | 1430339 (978663-2036962) | 660.09 (446.97-942.04) | 9794 (8587-10887) | 4.07 (3.56-4.53) | 0.06 (0.03-0.09) | 0.01 (-0.03-0.05) | 0.3 (0.25-0.34) | 2.98 (2.72-3.24) |
| Brunei Darussalam | 63739 (61957-65743) | 26980 (26380.63-27664.48) | 92484 (87829-97373) | 42482.49 (40454.32-44666.07) | 1564 (1012-2260) | 609.89 (401.89-866.22) | 2 (1-2) | 2.16 (1.76-2.64) | 119157 (116430-122295) | 27834.48 (27230.71-28499.62) | 182172 (173568-191799) | 43246.19 (41201.21-45423.59) | 2647 (1765-3727) | 638.58 (429.26-895.63) | 6 (5-8) | 2.87 (2.37-3.46) | 0.12 (0.1-0.14) | 0.07 (0.06-0.09) | 0.22 (0.2-0.24) | 1.9 (1.56-2.23) |
| Bulgaria | 1869553 (1811450-1934668) | 20280.34 (19672.11-20964.1) | 4224410 (3995012-4466895) | 44511.89 (42267.36-46826.49) | 34711 (22479-49936) | 413.54 (268.53-597.65) | 25 (23-28) | 0.27 (0.25-0.29) | 1673225 (1621086-1732665) | 21085.24 (20524.26-21774.43) | 3913181 (3677871-4153262) | 45335.39 (43131.36-47712.97) | 28763 (19049-41014) | 434.52 (283.35-623.08) | 64 (53-77) | 0.5 (0.42-0.6) | 0.16 (0.13-0.18) | 0.08 (0.06-0.09) | 0.2 (0.17-0.23) | 3.38 (2.18-4.6) |
| Burkina Faso | 2455357 (2242026-2700608) | 26618.34 (25010.62-28296.48) | 7101205 (6661077-7584391) | 79774.87 (75615.53-84121.86) | 42856 (28510-63202) | 429.9 (285.9-629.18) | 101 (69-145) | 1.88 (1.15-2.89) | 5890648 (5454228-6392385) | 27046.14 (25426.96-28709.77) | 17043934 (16032356-18250572) | 80837.12 (76889.84-85237.18) | 103669 (68124-150995) | 444.64 (293.19-647.66) | 223 (126-387) | 1.88 (1.07-3.22) | 0.05 (0.04-0.06) | 0.05 (0.04-0.07) | 0.15 (0.13-0.17) | 0.19 (0.11-0.28) |
| Burundi | 1642222 (1504026-1797743) | 29545 (27824.63-31441.46) | 4424840 (4153824-4736974) | 82532.58 (78556.95-87126.77) | 33443 (23057-47647) | 572.6 (396.93-820.47) | 125 (88-184) | 4.06 (2.69-6.65) | 3793058 (3517770-4106514) | 28992.73 (27425.73-30768.97) | 10336460 (9687984-11047917) | 82160.07 (78094.14-86787.8) | 72884 (47923-108717) | 536.61 (359.16-795.06) | 163 (93-270) | 3.17 (1.92-5.18) | -0.07 (-0.07--0.06) | -0.03 (-0.04--0.02) | -0.24 (-0.25--0.22) | -1.07 (-1.17--0.97) |
| Cambodia | 2574236 (2455396-2701008) | 26660.9 (25501.66-27865.53) | 5493259 (5172489-5898034) | 58239.35 (55025.86-62213.28) | 60081 (40018-86579) | 580.27 (391.93-832.16) | 131 (99-166) | 3.01 (2.07-4.45) | 4608502 (4402190-4829843) | 27734.25 (26548.83-29000.38) | 9712569 (9198311-10376460) | 59162.86 (55912.2-63013.75) | 97787 (64251-142026) | 587.58 (390.3-850.8) | 250 (192-315) | 2.75 (2.13-3.54) | 0.14 (0.13-0.15) | 0.05 (0.05-0.06) | 0.04 (0.02-0.05) | -0.36 (-0.44--0.27) |
| Cameroon | 2579840 (2401416-2779454) | 26113.72 (24763.07-27518.35) | 7578655 (7150329-8061361) | 78936.17 (75160.26-83159.96) | 42806 (26884-64104) | 389.58 (244.21-592.89) | 7 (6-9) | 0.09 (0.08-0.12) | 7804012 (7484719-8138594) | 26034.79 (25044.7-27109.08) | 22948972 (21807776-24203919) | 79212.87 (75529.01-83176.59) | 136359 (86958-201114) | 407.21 (258.88-612.96) | 47 (31-74) | 0.16 (0.11-0.23) | 0.02 (0.01-0.04) | 0.03 (0.02-0.04) | 0.19 (0.18-0.21) | 2.19 (1.95-2.43) |
| Canada | 6450894 (6333374-6576332) | 23075.96 (22640.27-23518.75) | 9569781 (9215517-9909409) | 33402.11 (32208.78-34545.94) | 153505 (104019-215148) | 570.37 (385.57-800) | 188 (170-201) | 0.6 (0.54-0.64) | 10390750 (10172090-10566573) | 24457.95 (24022.64-24911.33) | 16024171 (15388828-16656611) | 34938.88 (33771.02-36148.8) | 230488 (158454-317142) | 598.63 (405.6-834.4) | 793 (675-871) | 0.99 (0.86-1.08) | 0.21 (0.2-0.22) | 0.16 (0.16-0.17) | 0.18 (0.17-0.19) | 1.92 (1.63-2.2) |
| Central African Republic | 754789 (664076-860053) | 26676.19 (24508.21-29164.78) | 2002291 (1869050-2169818) | 76400.23 (72452.33-81053.09) | 14820 (10531-21245) | 517.74 (373.93-725.8) | 64 (49-82) | 4.43 (3.51-5.57) | 1456397 (1295604-1631866) | 26003.59 (24009.24-28252.36) | 3957873 (3716895-4259069) | 76282.19 (72436.68-80841.4) | 27521 (18576-40162) | 498.76 (348.3-712.82) | 95 (63-134) | 3.98 (2.66-5.49) | -0.09 (-0.11--0.08) | 0 (-0.02-0.03) | -0.12 (-0.13--0.11) | -0.36 (-0.42--0.3) |
| Chad | 1565503 (1432139-1711989) | 26821.58 (25090.96-28493.03) | 4527142 (4251017-4851984) | 79978.06 (75921.42-84475.41) | 24145 (15177-36833) | 377.64 (236.2-581.89) | 4 (3-5) | 0.06 (0.05-0.08) | 4627737 (4247567-5039713) | 27192.15 (25606.8-28786.19) | 13222790 (12376145-14189595) | 80945.3 (76808.78-85329.12) | 75683 (47499-114713) | 393.19 (245.2-601.23) | 29 (19-42) | 0.13 (0.09-0.19) | 0.05 (0.04-0.06) | 0.05 (0.04-0.07) | 0.15 (0.14-0.15) | 2.73 (2.41-3.06) |
| Chile | 3061923 (2977674-3153741) | 23833.76 (23226.34-24498.08) | 5203112 (4964716-5465435) | 41941.74 (39906.1-44101.38) | 72090 (47459-102997) | 544.56 (360.25-773.42) | 108 (100-115) | 1.23 (1.13-1.31) | 5049136 (4931210-5177718) | 25596.8 (24991.27-26272.74) | 9063562 (8599040-9545115) | 43763.91 (41767.42-45964.1) | 112752 (78487-155386) | 602 (410.8-839.64) | 823 (715-912) | 3.14 (2.74-3.47) | 0.25 (0.24-0.26) | 0.14 (0.14-0.15) | 0.35 (0.31-0.4) | 3.47 (2.62-4.34) |
| China | 263028704 (254416357-271911261) | 22739.94 (22027.89-23456.26) | 551626774 (522171539-584510772) | 48417.32 (45903.55-51109.43) | 6220772 (4105723-8987533) | 530.28 (351.12-764.81) | 6511 (5623-7451) | 1.24 (1.06-1.45) | 365902580 (356230022-376032802) | 24852.41 (24125.76-25561.88) | 754334944 (714607164-792948185) | 50119.8 (47601.14-52795.98) | 7478377 (4833412-10992080) | 546.03 (353.11-798.16) | 6378 (5231-7666) | 0.4 (0.33-0.48) | 0.3 (0.3-0.31) | 0.11 (0.11-0.12) | 0.08 (0.07-0.09) | -4.44 (-4.84--4.05) |
| Colombia | 6773493 (6543911-6997694) | 22251.24 (21591.01-22921.9) | 14298385 (13662110-15062505) | 48596.36 (46310.79-51058.55) | 165785 (111421-235830) | 521.2 (356.75-734.1) | 437 (411-461) | 2.56 (2.39-2.71) | 11470540 (11130618-11789363) | 23050.65 (22384.85-23693.12) | 24648702 (23423021-25885578) | 49137.15 (46721.37-51571.28) | 258615 (178014-357840) | 533.57 (363.91-740.06) | 1364 (1089-1676) | 2.43 (1.95-2.98) | 0.12 (0.11-0.13) | 0.04 (0.03-0.05) | 0.21 (0.16-0.27) | 1.36 (0.6-2.13) |
| Commonwealth of the Bahamas | 65323 (63065-67667) | 27187.05 (26308.92-28050.55) | 142284 (134824-150858) | 61057.9 (57989.77-64666.28) | 1596 (1107-2215) | 672.83 (481.81-918.38) | 10 (9-11) | 7.05 (6.44-7.67) | 108072 (104532-111533) | 27612.16 (26714.68-28440.02) | 239141 (226616-253369) | 61327.41 (58414.53-64806) | 2685 (1905-3658) | 709.11 (502.03-963.58) | 29 (24-35) | 8.55 (7.07-10.28) | 0.06 (0.05-0.07) | 0.02 (0.01-0.03) | 0.17 (0.12-0.21) | 0.6 (0.42-0.8) |
| Comoros | 138592 (128269-151124) | 29988.73 (28378.06-31802.64) | 369808 (348650-395060) | 83290.33 (79383.04-87842.39) | 2814 (1914-4084) | 575.05 (398.14-823.61) | 10 (7-13) | 3.98 (2.73-5.78) | 218385 (205788-232482) | 30006.89 (28346.21-31694.89) | 596550 (565408-632540) | 83629.78 (79554.95-88235.97) | 4196 (2795-6157) | 570.93 (388.13-829.48) | 18 (12-25) | 3.86 (2.75-5.45) | -0.01 (-0.02-0) | 0 (-0.01-0.01) | -0.07 (-0.09--0.04) | -0.3 (-0.38--0.22) |
| Congo | 687164 (608879-772850) | 27542.71 (25343.58-29990.7) | 1791454 (1672505-1934365) | 77823.68 (73743.17-82484.15) | 12678 (8826-18420) | 527.83 (382.42-756.92) | 56 (44-70) | 5.02 (3.81-6.24) | 1406354 (1285671-1543483) | 26559.38 (24775.81-28622.6) | 3931373 (3722474-4196798) | 77665.75 (74082.1-81849.53) | 26188 (17811-37217) | 510.81 (356.78-715.81) | 111 (75-152) | 4.55 (2.95-6.06) | -0.14 (-0.15--0.13) | 0 (-0.02-0.02) | -0.16 (-0.19--0.13) | -0.5 (-0.6--0.39) |
| Cook Islands | 5052 (4865-5250) | 27186.28 (26264.32-28125.36) | 11583 (10935-12318) | 63368.41 (60051.38-67032.24) | 119 (76-177) | 616.14 (398.99-915.66) | 0 (0-0) | 1.04 (0.88-1.25) | 5225 (5060-5395) | 28225.18 (27333.87-29207.16) | 11955 (11309-12613) | 64324.16 (60860.65-68122.98) | 114 (75-167) | 644.83 (419-948.07) | 0 (0-0) | 1.35 (1.05-1.69) | 0.11 (0.11-0.12) | 0.04 (0.04-0.05) | 0.17 (0.15-0.18) | 1.43 (1.16-1.71) |
| Costa Rica | 639161 (618113-659303) | 22329.85 (21704.32-22996.12) | 1347472 (1284817-1417342) | 48490.71 (46160.24-50938.03) | 14938 (9736-21577) | 490.32 (323.01-701.53) | 22 (20-24) | 1.32 (1.17-1.46) | 1124526 (1091988-1156373) | 23271.98 (22594.16-23898.1) | 2404419 (2281904-2524872) | 49255.45 (46821.6-51664.86) | 24697 (16911-34587) | 526.54 (357.94-741.76) | 118 (101-133) | 2.11 (1.82-2.36) | 0.13 (0.13-0.14) | 0.05 (0.05-0.05) | 0.22 (0.19-0.25) | 1.39 (1.04-1.73) |
| Croatia | 1027195 (995938-1061160) | 20425.45 (19837.28-21104.21) | 2250969 (2137640-2375094) | 43734.61 (41540.66-46030.18) | 19124 (12310-27525) | 410.35 (264.47-588.2) | 8 (8-9) | 0.16 (0.14-0.17) | 1043549 (1010726-1079850) | 21112.13 (20535.48-21795.62) | 2388737 (2247242-2531545) | 44371.86 (42156.17-46679.26) | 17419 (11315-25272) | 426.01 (276.19-617.77) | 21 (18-23) | 0.23 (0.2-0.26) | 0.15 (0.13-0.16) | 0.07 (0.06-0.08) | 0.16 (0.14-0.17) | 2.83 (1.65-4.02) |
| Cuba | 2726009 (2625074-2819312) | 25361.28 (24465.26-26199.77) | 6245784 (5919687-6614815) | 58649.33 (55590.76-62160.1) | 56534 (36938-80884) | 530.72 (346.73-760.28) | 148 (134-162) | 1.58 (1.43-1.74) | 3202366 (3095263-3315931) | 25651.36 (24791.03-26507.57) | 7652212 (7230226-8154742) | 58798.57 (55958.86-62241.58) | 62367 (41787-89754) | 544.34 (357.87-781.64) | 353 (305-398) | 1.71 (1.48-1.93) | 0.07 (0.05-0.09) | 0.02 (0.01-0.03) | 0.08 (0.05-0.11) | 0.06 (-0.34-0.45) |
| Cyprus | 207136 (201304-213659) | 26501.2 (25771.62-27290.72) | 406358 (384507-427606) | 51766.55 (49119.26-54387.76) | 4688 (3262-6493) | 623.86 (439.01-860.89) | 22 (14-28) | 4.77 (3.03-6.19) | 405866 (395115-417977) | 28144.8 (27371.07-28959) | 814153 (770093-857807) | 53190.51 (50525.62-55801.78) | 8563 (5985-11823) | 639.41 (443.29-882.99) | 48 (39-59) | 3.18 (2.61-3.86) | 0.2 (0.18-0.22) | 0.09 (0.08-0.1) | 0.07 (0.05-0.09) | -1.43 (-1.74--1.11) |
| Czech Republic | 2252258 (2186076-2323472) | 20740.66 (20165.69-21402.84) | 5169149 (4904846-5441383) | 46408.4 (44204.14-48734.31) | 42654 (28033-60837) | 424.33 (278.93-605.85) | 68 (63-75) | 0.54 (0.5-0.59) | 2649569 (2568243-2739309) | 21525.25 (20949.16-22199.11) | 6274070 (5922515-6654056) | 47300.14 (45059.98-49691.7) | 47941 (32528-67866) | 448.85 (298.55-637.75) | 229 (195-262) | 1.06 (0.91-1.21) | 0.14 (0.13-0.15) | 0.07 (0.07-0.08) | 0.23 (0.19-0.27) | 4.46 (2.82-6.12) |
| Democratic People's Republic of Korea | 4533552 (4396423-4674241) | 22550.95 (21904.11-23217.03) | 9685264 (9226941-10210731) | 48384.04 (46144.01-50803.08) | 105898 (68497-153424) | 517.36 (334.32-749.57) | 89 (66-118) | 0.76 (0.55-1.01) | 6015746 (5829824-6205667) | 22693.55 (22008-23375.95) | 13132453 (12468859-13797277) | 48500.2 (46198.59-51035.2) | 131058 (83902-191243) | 521.1 (333.88-757.51) | 119 (88-165) | 0.48 (0.35-0.66) | 0.03 (0.02-0.04) | 0.01 (0-0.02) | 0.03 (0.02-0.04) | -1.56 (-1.61--1.51) |
| Democratic Republic of the Congo | 10437856 (9228402-11837118) | 26399.83 (24310.64-28718.48) | 27946443 (26027596-30342257) | 76287.33 (72463.94-80969.86) | 197242 (135093-280825) | 496.07 (347.6-698.87) | 742 (512-994) | 4.03 (2.79-5.5) | 22672116 (20452882-25094873) | 24907.79 (23231.59-26779.2) | 64208944 (60034640-69100576) | 75397.82 (71717.97-79668.71) | 419098 (282138-610459) | 467.11 (324.63-663.14) | 1183 (803-1744) | 3.38 (2.16-4.64) | -0.19 (-0.21--0.17) | -0.03 (-0.05--0.01) | -0.21 (-0.25--0.17) | -0.71 (-0.81--0.6) |
| Denmark | 1554631 (1515528-1598810) | 28338.57 (27537.94-29145.76) | 3146159 (2978935-3316255) | 53815.34 (51208.27-56479.63) | 32705 (21997-46023) | 651.99 (433-932) | 41 (38-44) | 0.48 (0.44-0.51) | 1949188 (1902185-2002810) | 29711.14 (28932.14-30526.79) | 3981887 (3766011-4215622) | 55410.03 (52799.03-58075.56) | 40550 (27616-56515) | 689.1 (463.43-977.67) | 177 (150-195) | 1.26 (1.08-1.39) | 0.17 (0.16-0.18) | 0.11 (0.1-0.12) | 0.19 (0.17-0.21) | 2.48 (2.05-2.9) |
| Djibouti | 120430 (111641-130500) | 29841.98 (28243.8-31682.37) | 323576 (303758-344180) | 83318.29 (79487.83-87762.06) | 2344 (1580-3456) | 544.25 (365.62-794.87) | 5 (4-8) | 3.07 (1.9-5.4) | 358056 (337315-380835) | 29965.03 (28434.91-31688.52) | 986130 (930898-1044212) | 83780.55 (79634.22-88442.59) | 6639 (4387-9740) | 551.37 (369.9-804.12) | 19 (12-29) | 3.42 (2.28-5.08) | 0.01 (0-0.01) | 0.01 (0-0.02) | 0.03 (0.01-0.05) | 0.34 (0.26-0.41) |
| Dominica | 18087 (17448-18715) | 25403.69 (24558.39-26290.34) | 41066 (39019-43544) | 58977.32 (56098.44-62689.18) | 464 (331-638) | 651.07 (469.64-890.59) | 4 (4-6) | 8.05 (6.39-10.57) | 18256 (17677-18851) | 26212.51 (25316.85-27040.61) | 41901 (39711-44510) | 59808.55 (56900.66-63277.31) | 479 (355-639) | 710.04 (521.27-946.91) | 7 (6-8) | 9.62 (7.87-11.59) | 0.1 (0.1-0.1) | 0.04 (0.04-0.05) | 0.27 (0.25-0.3) | 0.47 (0.36-0.59) |
| Dominican Republic | 1670886 (1608302-1734706) | 25069.57 (24201.32-25935.74) | 3756568 (3557691-3991026) | 58512.67 (55624.86-62231.06) | 36277 (22862-52834) | 495.66 (313.97-725.04) | 7 (4-12) | 0.2 (0.1-0.36) | 2837649 (2739796-2928198) | 26242.78 (25365.91-27074.64) | 6427889 (6113144-6811609) | 59737.74 (56808.24-63450.39) | 55966 (35849-81185) | 515.37 (329.48-749.13) | 18 (10-32) | 0.19 (0.11-0.33) | 0.16 (0.15-0.16) | 0.07 (0.07-0.07) | 0.14 (0.13-0.14) | 0.23 (-0.3-0.77) |
| Ecuador | 2720961 (2603488-2843262) | 28982.88 (27827.83-30203.96) | 5864258 (5525348-6254483) | 64481.7 (60840.31-68274.55) | 57400 (36668-83270) | 563.21 (358.44-819.55) | 6 (6-7) | 0.13 (0.12-0.14) | 5299143 (5097513-5515056) | 29687.61 (28576.22-30868.62) | 11499927 (10833791-12167270) | 64935.92 (61225.15-68664.2) | 111058 (73848-157263) | 620.17 (413.89-877.95) | 258 (202-331) | 1.76 (1.4-2.24) | 0.1 (0.09-0.1) | 0.04 (0.03-0.04) | 0.32 (0.27-0.38) | 8.35 (5.82-10.93) |
| Egypt | 9473072 (9207941-9777577) | 18302.67 (17818.22-18830.92) | 21096585 (20437808-21821851) | 41657.28 (40270.26-43078.95) | 209963 (140295-296301) | 376.56 (255.03-525.97) | 209 (144-375) | 1.27 (0.84-2.43) | 18948756 (18381760-19557193) | 19094.13 (18570.74-19640.69) | 40326846 (39091712-41773767) | 41534.93 (40208.15-42988.68) | 408089 (270920-577163) | 390.09 (260.64-549.56) | 443 (350-538) | 1.13 (0.91-1.34) | 0.16 (0.13-0.19) | 0.01 (-0.01-0.03) | 0.13 (0.1-0.16) | 0.02 (-0.12-0.16) |
| El Salvador | 1099137 (1059988-1137892) | 21661.3 (20992.29-22303.79) | 2339907 (2225890-2471767) | 47955.09 (45571.63-50357.75) | 26367 (17252-37781) | 482.33 (319.99-686.55) | 44 (38-54) | 1.39 (1.17-1.75) | 1448549 (1403800-1492014) | 22517.12 (21821.7-23172.36) | 3109948 (2956347-3269738) | 48483.94 (46103.53-50906.15) | 32752 (21953-46299) | 507.18 (340.63-716.81) | 114 (91-140) | 1.7 (1.34-2.09) | 0.13 (0.13-0.13) | 0.04 (0.03-0.04) | 0.18 (0.17-0.19) | 0.79 (0.65-0.94) |
| Equatorial Guinea | 118494 (104321-134328) | 26770.99 (24585.05-29194.43) | 313727 (292986-341019) | 76601.87 (72750.69-81419.45) | 2238 (1577-3272) | 501.51 (355.79-721.98) | 9 (7-12) | 4.02 (3.08-5.21) | 403919 (370385-441539) | 27377.94 (25777.89-29211.45) | 1100996 (1034180-1178053) | 78898.13 (75129.73-83123.68) | 7598 (5094-10893) | 528.31 (370.93-761.39) | 23 (14-35) | 4.64 (2.56-7.06) | 0.13 (0.09-0.18) | 0.14 (0.1-0.18) | 0.23 (0.21-0.26) | 0.7 (0.54-0.86) |
| Eritrea | 1007080 (920124-1101160) | 29614.99 (27896.92-31530.59) | 2680764 (2509371-2883274) | 82776 (78657.7-87512.66) | 20043 (13866-28766) | 568.51 (397.72-812.99) | 57 (41-81) | 4.12 (2.81-7.26) | 1930755 (1795115-2080908) | 30041.19 (28384.38-31814.13) | 5201618 (4890513-5535032) | 83783.42 (79658.41-88423.14) | 37283 (24785-54388) | 576.82 (394.18-829.41) | 113 (76-172) | 4.47 (3.23-6.57) | 0.04 (0.02-0.06) | 0.02 (0.01-0.03) | 0.05 (0.04-0.07) | 0.31 (0.23-0.38) |
| Estonia | 345142 (334934-357052) | 21342.66 (20713.67-22039.8) | 770790 (732301-811183) | 45668.85 (43525.2-47978.28) | 7465 (4937-10607) | 495.51 (323.99-712.83) | 15 (13-17) | 0.77 (0.66-0.87) | 333430 (324337-344142) | 22484.39 (21859.17-23169.66) | 780133 (737231-822942) | 46958.02 (44839.5-49323.58) | 6441 (4319-8931) | 516.56 (339.07-743.28) | 26 (22-29) | 1.01 (0.87-1.14) | 0.2 (0.19-0.21) | 0.12 (0.1-0.13) | 0.13 (0.12-0.15) | 0.81 (0.35-1.27) |
| Ethiopia | 21466848 (19450086-23710410) | 39258.39 (36548.97-41979.79) | 51315779 (47725803-55938372) | 99690.5 (94072.95-106271.43) | 370644 (248283-556252) | 682.33 (468.3-1010.85) | 1134 (603-1583) | 4.49 (2.7-7.04) | 41431282 (38188476-44876331) | 37380.8 (35113.17-39792.4) | 106278535 (99719895-113997726) | 99313.09 (94287.62-105322.89) | 712822 (456612-1068731) | 627.28 (409.79-934.57) | 1417 (985-2153) | 3 (2.12-4.47) | -0.21 (-0.22--0.19) | -0.09 (-0.12--0.06) | -0.39 (-0.44--0.34) | -1.65 (-1.79--1.51) |
| Federated States of Micronesia | 26331 (25235-27477) | 25990.71 (25069.51-26966.3) | 61169 (57283-65623) | 62263.3 (58890.85-66157.94) | 709 (473-1034) | 684.49 (472.86-967.68) | 2 (1-3) | 3.83 (2.73-5.4) | 26795 (25811-27876) | 26359.09 (25438.18-27312.95) | 62517 (59085-66588) | 62540.81 (59126.86-66115.8) | 710 (480-1006) | 696.55 (478.07-983.35) | 3 (2-4) | 3.92 (2.95-5.17) | 0.04 (0.04-0.04) | 0.01 (0.01-0.01) | 0.05 (0.04-0.06) | 0.06 (0.01-0.11) |
| Fiji | 200519 (192599-208643) | 27239.08 (26255.86-28197.62) | 472750 (444849-506897) | 65349.98 (61786.71-69308.95) | 5100 (3353-7537) | 671.04 (450.8-987.63) | 9 (7-12) | 2.63 (2.1-3.63) | 255023 (245977-264485) | 27988.86 (27000.62-28979.91) | 599170 (567101-637982) | 66167.78 (62618.43-70239.31) | 6411 (4325-9299) | 705.07 (480.45-1018.18) | 21 (17-27) | 3.51 (2.79-4.32) | 0.05 (-0.07-0.16) | -0.01 (-0.18-0.15) | 0.13 (-0.01-0.27) | 0.93 (0.74-1.13) |
| Finland | 1458501 (1418762-1500470) | 27846.47 (27097.38-28586.12) | 2863493 (2700172-3017285) | 51936.83 (49288.29-54554.78) | 30035 (20071-42217) | 615.29 (406.09-866.4) | 20 (18-22) | 0.29 (0.26-0.31) | 1878124 (1832051-1930316) | 29242.88 (28507.53-30048.66) | 3796973 (3576780-4034829) | 53275.87 (50734.92-56013.15) | 35751 (24101-51039) | 644.64 (431.16-908.77) | 74 (61-83) | 0.46 (0.38-0.51) | 0.18 (0.17-0.19) | 0.1 (0.09-0.1) | 0.16 (0.16-0.17) | 0.96 (0.74-1.18) |
| France | 17356262 (16884823-17870651) | 28476.11 (27655.29-29328.19) | 32327897 (30404588-34084537) | 50384.82 (47769.86-53041.03) | 403864 (276653-560793) | 693.07 (466.89-971.81) | 2244 (1969-2423) | 2.53 (2.23-2.73) | 22563548 (21985634-23170490) | 29832.7 (29017.61-30638.92) | 43406768 (40803089-46040258) | 51757.48 (49160.74-54441.29) | 485880 (336037-674200) | 709.57 (477.52-1008.31) | 2829 (2344-3169) | 1.4 (1.19-1.55) | 0.15 (0.14-0.16) | 0.09 (0.09-0.1) | 0.04 (0.02-0.06) | -2.49 (-2.82--2.16) |
| Gabon | 282839 (275269-290241) | 28173.7 (27428.51-28898.65) | 746502 (706200-799626) | 78451.13 (74774.33-82829.93) | 5235 (3599-7438) | 526.81 (373.1-737.43) | 24 (18-32) | 4.27 (3.16-5.54) | 487036 (472746-500459) | 27309.45 (26559.27-28032.59) | 1338154 (1275784-1415855) | 78004.44 (74698.88-81877.79) | 9455 (6425-13416) | 540.17 (380.92-758.8) | 43 (26-62) | 4.73 (2.68-6.62) | -0.12 (-0.14--0.09) | 0.02 (-0.01-0.05) | 0.06 (0.01-0.11) | 0.19 (-0.02-0.4) |
| Georgia | 1211543 (1173677-1252314) | 21765.18 (21096.35-22469.13) | 2315726 (2195024-2448722) | 40767.61 (38588.58-43005.54) | 26673 (16974-38870) | 497.59 (313.72-731.99) | 3 (2-3) | 0.04 (0.04-0.06) | 850545 (825477-879807) | 21930.77 (21268.45-22618.73) | 1731812 (1630651-1831480) | 40874.88 (38705.13-43185.04) | 17613 (11560-25219) | 515.41 (332.86-745.92) | 28 (23-32) | 0.49 (0.41-0.58) | 0.1 (0.05-0.15) | 0.06 (0.02-0.09) | 0.2 (0.16-0.24) | 11.19 (9.97-12.42) |
| Germany | 24913061 (24252907-25625858) | 29466.17 (28649.38-30304.72) | 51432782 (48685878-54207392) | 56743.67 (54111.49-59551.54) | 541659 (368510-760918) | 693.42 (467.51-972.19) | 536 (471-587) | 0.41 (0.36-0.45) | 30043648 (29343572-30868529) | 30844.25 (30056.69-31652.08) | 63632667 (60184243-67229207) | 58399.02 (55712.89-61166.86) | 640396 (446071-883787) | 734.79 (501.27-1020.79) | 2871 (2387-3175) | 1.2 (1.02-1.33) | 0.12 (0.1-0.14) | 0.07 (0.06-0.08) | 0.15 (0.13-0.17) | 4.63 (3.91-5.35) |
| Ghana | 3144987 (3045860-3247310) | 21592.71 (20982.86-22259.02) | 9696896 (9120151-10303124) | 69209.28 (66031.62-72650.68) | 58124 (39138-82938) | 371.22 (247.75-533.82) | 102 (76-137) | 1.23 (0.89-1.65) | 7352152 (7120760-7604965) | 22410.92 (21777.94-23111.44) | 22550105 (21450298-23730752) | 70694.98 (67655.33-74022.6) | 149090 (105842-208123) | 446 (320.35-618.48) | 599 (457-787) | 3.17 (2.37-4.13) | 0.15 (0.13-0.18) | 0.09 (0.07-0.11) | 0.87 (0.74-1) | 4.59 (3.8-5.38) |
| Greece | 2901242 (2815962-2995268) | 26150.94 (25357.84-26970.98) | 5466145 (5134101-5790100) | 47264.25 (44688.08-49913.23) | 57513 (38635-81829) | 557.87 (373.69-789.7) | 15 (14-17) | 0.11 (0.1-0.12) | 3304668 (3223212-3406013) | 27514.22 (26747.2-28369.05) | 6562438 (6169172-7014221) | 48531.17 (45976.42-51151.04) | 59757 (39866-85371) | 589.21 (391.87-827.15) | 74 (63-82) | 0.22 (0.19-0.24) | 0.18 (0.16-0.21) | 0.1 (0.08-0.11) | 0.19 (0.18-0.2) | 1.71 (1.41-2.01) |
| Greenland | 11988 (11751-12238) | 23248.84 (22825.87-23692.91) | 17013 (16386-17678) | 33883.36 (32639.06-35036.77) | 301 (203-421) | 570.03 (385.26-792.52) | 0 (0-0) | 1.14 (1-1.27) | 14485 (14186-14747) | 24966.97 (24507.45-25408.8) | 21015 (20262-21812) | 35687.02 (34507.43-36881.34) | 335 (229-463) | 602.98 (408.68-838.35) | 1 (1-1) | 1.46 (1.25-1.74) | 0.27 (0.26-0.28) | 0.2 (0.19-0.21) | 0.25 (0.23-0.27) | 1.72 (1.39-2.06) |
| Grenada | 21725 (20970-22508) | 25317.94 (24464.56-26197.49) | 49683 (47097-52814) | 58926.58 (56071.1-62530.7) | 571 (412-776) | 652.52 (481.43-880.64) | 7 (6-8) | 8.32 (7.29-9.29) | 27408 (26537-28326) | 26408.68 (25593.03-27244.86) | 62587 (59396-66412) | 60154.17 (57224.47-63760.94) | 793 (610-1035) | 790.78 (605.64-1027.38) | 13 (11-14) | 14.72 (12.91-16.18) | 0.14 (0.13-0.15) | 0.07 (0.07-0.07) | 0.58 (0.5-0.67) | 1.9 (1.63-2.16) |
| Guam | 36907 (35576-38319) | 28252.71 (27320.68-29207.47) | 84087 (79524-89596) | 64609.75 (61137.85-68362.15) | 887 (576-1282) | 665.02 (440.78-957.44) | 2 (1-2) | 2.39 (2.07-2.82) | 47085 (45650-48627) | 28762.9 (27858.95-29724.25) | 107559 (102130-113231) | 65249.82 (61827.32-68971.29) | 1136 (783-1609) | 709.39 (482.92-1011.24) | 6 (5-7) | 2.81 (2.4-3.27) | 0.07 (0.07-0.08) | 0.04 (0.03-0.05) | 0.35 (0.3-0.4) | 1.91 (1.44-2.38) |
| Guatemala | 1702721 (1639409-1766029) | 21774.5 (21082.98-22452.12) | 3612182 (3428006-3834753) | 48271.32 (45940.3-50682.1) | 41876 (27338-60373) | 485.61 (328.48-688.39) | 58 (52-65) | 2 (1.81-2.18) | 3425993 (3315137-3533932) | 22492.78 (21829.81-23167.34) | 7198423 (6853930-7579926) | 48699.24 (46357.24-51138.04) | 80146 (54100-113681) | 512.28 (350.21-721.53) | 218 (184-256) | 2.17 (1.85-2.52) | 0.11 (0.1-0.11) | 0.03 (0.03-0.04) | 0.15 (0.11-0.19) | -0.05 (-0.5-0.39) |
| Guinea | 1601529 (1469403-1758225) | 27450.6 (25722.14-29249.72) | 4592942 (4315817-4930900) | 80798.78 (76669.27-85492.7) | 24285 (15335-37454) | 386.18 (241.51-596.19) | 6 (4-9) | 0.09 (0.06-0.13) | 3569672 (3295211-3879537) | 27494.93 (25803.82-29203.63) | 10162342 (9558131-10853081) | 81368.92 (77313.72-85756.31) | 57438 (36251-86483) | 400.84 (250.97-611.26) | 21 (14-31) | 0.13 (0.09-0.2) | 0 (-0.01-0.01) | 0.02 (0.01-0.04) | 0.13 (0.12-0.14) | 1.66 (1.22-2.1) |
| Guinea-Bissau | 276205 (249359-305151) | 27929.78 (26127.59-29834.33) | 760495 (712193-816627) | 81106.25 (76863.37-85805.89) | 4208 (2634-6489) | 389.96 (243.71-603.72) | 1 (1-1) | 0.09 (0.07-0.12) | 552035 (506955-600535) | 27795.09 (26143.8-29525.91) | 1550494 (1458217-1666439) | 81705.7 (77723.1-86401.16) | 8826 (5538-13415) | 403.81 (252.05-620.05) | 3 (2-4) | 0.14 (0.1-0.19) | -0.03 (-0.04--0.02) | 0.02 (0.01-0.03) | 0.12 (0.11-0.13) | 1.8 (1.42-2.19) |
| Guyana | 179981 (173005-187184) | 24900.35 (23988.16-25740.33) | 405952 (384553-432425) | 58371.64 (55417.61-62120.06) | 3945 (2498-5763) | 497.81 (315.54-723.2) | 1 (1-1) | 0.38 (0.34-0.42) | 193722 (186968-200189) | 26106.06 (25187.42-26941.17) | 436742 (414336-462936) | 59611.55 (56682.39-63221.29) | 4189 (2787-5945) | 559.7 (375.22-792.88) | 11 (9-14) | 2.12 (1.68-2.59) | 0.14 (0.13-0.14) | 0.06 (0.06-0.06) | 0.33 (0.26-0.4) | 4.34 (2.66-6.06) |
| Haiti | 1456583 (1397625-1517116) | 24556.63 (23669.41-25403.68) | 3334353 (3149470-3547711) | 58175.41 (55096.14-61872.62) | 40084 (27767-55883) | 626.65 (448.12-855.58) | 180 (134-229) | 5.76 (4.05-8.86) | 2963386 (2844387-3082075) | 24714.57 (23845.39-25598.19) | 6768771 (6408175-7187817) | 58192.12 (55182.29-61774.67) | 77414 (54030-106592) | 633.07 (441.79-872.28) | 362 (250-515) | 5.75 (3.72-8.97) | 0.02 (0.02-0.03) | 0 (0-0) | 0.11 (0.09-0.13) | 0.27 (0.18-0.36) |
| Honduras | 954869 (921147-989880) | 21594.83 (20951.98-22229.03) | 2026933 (1922562-2146328) | 47983.93 (45660-50380.64) | 23083 (14933-33655) | 466.53 (308.86-673.1) | 18 (14-26) | 0.9 (0.61-1.4) | 2144348 (2070458-2218379) | 22241.16 (21551.01-22970.38) | 4511916 (4291148-4747729) | 48445.53 (46071.61-50848.02) | 49083 (32368-70064) | 488.23 (325.43-691.56) | 72 (59-90) | 1.37 (1.11-1.68) | 0.09 (0.09-0.1) | 0.03 (0.03-0.03) | 0.15 (0.15-0.16) | 1.28 (1.03-1.52) |
| Hungary | 2321260 (2250667-2401962) | 21125.28 (20532.23-21798.31) | 5121036 (4847686-5407094) | 44811.43 (42592.06-47216.24) | 45450 (29685-64350) | 455.63 (296.88-651.17) | 61 (57-65) | 0.48 (0.44-0.51) | 2426173 (2350740-2503665) | 22020.94 (21409.63-22699.23) | 5532069 (5215534-5843983) | 45790.67 (43619.03-48122.01) | 45162 (30363-64000) | 482.99 (320.13-684.9) | 180 (157-202) | 0.95 (0.82-1.07) | 0.15 (0.14-0.15) | 0.08 (0.08-0.08) | 0.22 (0.19-0.25) | 3.82 (2.56-5.08) |
| Iceland | 70961 (69087-73090) | 27420.81 (26663.09-28260.7) | 126585 (119724-133569) | 48095.07 (45538.82-50710.24) | 1527 (1012-2160) | 603.98 (400.08-864.47) | 1 (1-1) | 0.21 (0.19-0.23) | 109353 (106424-112472) | 28816.76 (28020.34-29629.04) | 202623 (191103-214784) | 49635.05 (47104.13-52232.33) | 2200 (1482-3096) | 634.43 (424.29-896.45) | 3 (2-4) | 0.44 (0.35-0.51) | 0.17 (0.16-0.18) | 0.11 (0.1-0.12) | 0.16 (0.15-0.17) | 2.21 (1.97-2.45) |
| India | 179014576 (170639113-187948134) | 21441.94 (20610.75-22304.22) | 504776832 (479679559-533578686) | 62038.97 (59347.71-64903.29) | 4471212 (3083518-6229429) | 507.76 (353.35-706.11) | 13808 (11827-15564) | 2.58 (2.15-2.95) | 315289548 (304307450-326102808) | 22731.05 (21948.06-23516.66) | 876367214 (840564839-913203710) | 63838.95 (61259.02-66515) | 6852183 (4653173-9729118) | 495.39 (336.83-701.94) | 20695 (18141-23586) | 1.91 (1.66-2.17) | 0.23 (0.18-0.28) | 0.14 (0.08-0.2) | -0.09 (-0.15--0.03) | -1.28 (-1.48--1.09) |
| Indonesia | 50358572 (48071561-52829649) | 28316.16 (27178.82-29570.92) | 106535646 (99946744-114533834) | 61373.44 (57852.21-65469.14) | 1109602 (723943-1602233) | 592.08 (388.52-855.53) | 1593 (1241-2124) | 1.6 (1.19-2.27) | 82134975 (78756100-85775077) | 29624.58 (28449.64-30857.78) | 172573674 (162493744-184511927) | 62599.76 (59126.52-66725.54) | 1702511 (1135024-2441915) | 627.67 (420.41-899.16) | 4194 (3466-4941) | 2.37 (1.98-2.72) | 0.14 (0.13-0.15) | 0.06 (0.05-0.06) | 0.22 (0.2-0.24) | 1.75 (1.52-1.97) |
| Iraq | 2963067 (2874116-3058601) | 17707.24 (17246.55-18191.03) | 5949329 (5748215-6164676) | 37264.28 (35830.12-38732.27) | 69293 (45537-97666) | 364.71 (241.86-515.31) | 28 (21-37) | 0.34 (0.25-0.48) | 7334149 (7114565-7551461) | 18876.21 (18373.36-19380.93) | 14304169 (13781518-14847254) | 38157.76 (36700.3-39610.58) | 163844 (109959-232150) | 395.73 (264.29-557.97) | 68 (52-84) | 0.4 (0.3-0.49) | 0.3 (0.24-0.35) | 0.13 (0.08-0.18) | 0.31 (0.26-0.37) | 0.41 (0-0.82) |
| Ireland | 989067 (960939-1019868) | 26653.46 (25911.75-27467.73) | 1803032 (1698613-1906228) | 48479.53 (45808.69-51037.03) | 21759 (14754-30557) | 593.81 (401.64-834.36) | 44 (40-46) | 1.16 (1.06-1.23) | 1547997 (1505844-1589603) | 28970.44 (28191.45-29752.76) | 2889474 (2733620-3056916) | 51010.09 (48488.21-53657.49) | 31472 (21651-44303) | 632.34 (428.17-892.8) | 98 (80-111) | 1.14 (0.94-1.29) | 0.28 (0.26-0.29) | 0.17 (0.15-0.18) | 0.2 (0.18-0.22) | -0.26 (-0.57-0.04) |
| Islamic Republic of Iran | 9790987 (9519991-10066934) | 18560.64 (18042.93-19045.14) | 18618224 (17938086-19384129) | 37313.63 (35818.91-38821.09) | 233887 (155103-329247) | 395.8 (264.12-559.58) | 181 (152-233) | 0.94 (0.76-1.31) | 16481207 (16039066-16923976) | 19815.15 (19313.42-20340.76) | 31944696 (30630142-33269393) | 38196.01 (36710.89-39697.64) | 346390 (230471-487854) | 422.77 (282.07-595.13) | 569 (493-635) | 0.88 (0.76-0.99) | 0.26 (0.24-0.29) | 0.11 (0.1-0.12) | 0.25 (0.24-0.27) | -0.13 (-0.28-0.02) |
| Israel | 1458053 (1421631-1501225) | 29412.41 (28707.5-30234.45) | 2646452 (2483925-2814037) | 53921.66 (50756.44-57375.08) | 30887 (21100-43372) | 619.53 (426.82-867.54) | 147 (130-161) | 3.51 (3.08-3.83) | 3068641 (3003167-3148533) | 30739.14 (30056.26-31560.49) | 5652155 (5321674-6014126) | 55467.72 (52275.93-58959.24) | 67222 (47777-91255) | 667.8 (466.88-918.53) | 737 (601-830) | 5.17 (4.28-5.79) | 0.15 (0.14-0.16) | 0.09 (0.09-0.1) | 0.29 (0.23-0.35) | 1.65 (1.04-2.27) |
| Italy | 16890034 (16410832-17390154) | 27667.45 (26954.24-28462.56) | 30291930 (28298352-32238266) | 46461.42 (43723.94-49215) | 334119 (222091-473503) | 602.82 (395.99-859) | 325 (288-344) | 0.39 (0.35-0.42) | 20288138 (19731874-20882615) | 28786.93 (28094.79-29606.57) | 38621021 (35952370-41316146) | 47460.71 (44727.53-50197.35) | 372523 (251173-527894) | 627.33 (414.04-893.2) | 1370 (1110-1518) | 0.71 (0.59-0.77) | 0.13 (0.12-0.15) | 0.07 (0.06-0.08) | 0.14 (0.13-0.14) | 2.25 (1.95-2.56) |
| Jamaica | 580841 (559441-601620) | 25198.87 (24336.17-26050.39) | 1313352 (1244744-1394893) | 58499.99 (55578.43-61991.5) | 13921 (9459-19773) | 591.65 (409.54-831.68) | 91 (79-101) | 4.87 (4.22-5.41) | 731756 (706066-756110) | 25771.26 (24863.42-26611.67) | 1685605 (1598486-1789823) | 59024.33 (56058.23-62521.44) | 17918 (12918-24177) | 638.86 (452.29-866.81) | 215 (168-264) | 6.27 (4.91-7.76) | 0.07 (0.06-0.07) | 0.02 (0.02-0.03) | 0.19 (0.13-0.24) | 0.57 (0.28-0.85) |
| Japan | 34721413 (33941566-35684798) | 26760.53 (26152.25-27438.81) | 56440508 (53402789-59808933) | 41321.2 (39237.5-43712.56) | 735376 (471408-1055545) | 622.18 (396.75-907.92) | 474 (421-502) | 0.34 (0.29-0.36) | 43180750 (42196641-44413530) | 28017.65 (27405.68-28680.7) | 80198798 (74786031-86100309) | 42615.61 (40499.54-44991.61) | 773815 (499931-1103820) | 643.67 (409.63-935.5) | 2598 (2075-2903) | 0.52 (0.44-0.57) | 0.15 (0.13-0.16) | 0.1 (0.09-0.1) | 0.1 (0.09-0.12) | 1.62 (1.17-2.08) |
| Jordan | 620002 (599743-643143) | 18036.68 (17541.18-18563.18) | 1244774 (1199557-1291249) | 38267.04 (36778.52-39694.26) | 14814 (9788-21206) | 386.55 (260.82-543.3) | 11 (9-14) | 1.14 (0.93-1.48) | 2233893 (2162959-2317311) | 18915.46 (18378.8-19492.98) | 4430408 (4265472-4608853) | 38827.09 (37385.89-40324.95) | 49733 (33234-69854) | 407.08 (273.47-572.18) | 56 (46-69) | 1.19 (0.95-1.44) | 0.2 (0.18-0.22) | 0.08 (0.07-0.09) | 0.2 (0.18-0.21) | 0.26 (0.05-0.48) |
| Kazakhstan | 3476311 (3376763-3591434) | 21901.57 (21255.9-22618.4) | 6648255 (6331626-7008719) | 43409.38 (41212.71-45681.58) | 83363 (53048-124483) | 498.35 (319.05-736.02) | 23 (18-27) | 0.18 (0.14-0.22) | 4229306 (4106157-4363877) | 22767.61 (22093.95-23493.54) | 8219068 (7822593-8646623) | 44399.07 (42181.43-46657.13) | 96674 (61681-141411) | 518.25 (330.7-759.14) | 44 (35-56) | 0.25 (0.2-0.31) | 0.18 (0.14-0.21) | 0.11 (0.08-0.13) | 0.15 (0.13-0.16) | 0.93 (0.48-1.37) |
| Kenya | 6148824 (5759225-6587884) | 26970.54 (25720.09-28316.16) | 18081774 (17095028-19277835) | 81541.01 (77831.57-85758.75) | 131853 (89811-192513) | 543.04 (372.63-784.08) | 353 (229-673) | 3.49 (2.07-7.52) | 13899108 (13135809-14784515) | 28140.06 (26903.43-29538.44) | 39756775 (37666321-42069590) | 83705.16 (79678.15-88070.23) | 282869 (189501-409341) | 567.32 (390.89-805.76) | 869 (613-1366) | 4.2 (3.01-6.54) | 0.11 (0.1-0.13) | 0.06 (0.04-0.08) | 0.18 (0.15-0.2) | 0.9 (0.8-1.01) |
| Kingdom of Eswatini | 180014 (170851-189440) | 24471.95 (23458.63-25568.99) | 548120 (522046-576176) | 76317.57 (72797.43-79932.54) | 3595 (2383-5206) | 465.23 (318.42-663.76) | 10 (7-14) | 4.13 (2.87-6.13) | 273745 (260807-288053) | 25440.24 (24409.27-26554.95) | 819174 (781653-862034) | 77946.15 (74575.74-81601.84) | 5410 (3654-7659) | 508.64 (358.24-712.8) | 23 (17-31) | 5.37 (4-7.08) | 0.12 (0.11-0.13) | 0.07 (0.06-0.07) | 0.29 (0.13-0.44) | 0.86 (0.2-1.53) |
| Kiribati | 18497 (17712-19334) | 25796.46 (24884.81-26767.42) | 43801 (41250-46787) | 62140.78 (58844.21-66022.45) | 639 (464-858) | 939.39 (667.6-1349.96) | 5 (3-9) | 12.94 (7.73-25.64) | 30802 (29587-32102) | 26029.27 (25116.14-27042.79) | 72543 (68341-77395) | 62360.3 (58958.6-66168.87) | 1024 (722-1417) | 917.18 (648.69-1285.36) | 8 (5-14) | 12.77 (7.84-23.24) | 0.03 (0.02-0.03) | 0.01 (0.01-0.01) | -0.13 (-0.19--0.07) | -0.14 (-0.29-0) |
| Kuwait | 303309 (294483-313588) | 19909.82 (19382.37-20464.87) | 608057 (585845-632558) | 40480.85 (38976.12-41891.34) | 6595 (4330-9516) | 393.5 (255.88-568.61) | 1 (1-1) | 0.15 (0.12-0.18) | 902463 (877155-930114) | 20905.08 (20363.54-21469.54) | 1799509 (1727982-1878519) | 41236.54 (39783.31-42735.3) | 18024 (11942-25475) | 424.01 (279.5-601.78) | 11 (8-13) | 0.5 (0.38-0.62) | 0.2 (0.18-0.22) | 0.08 (0.07-0.1) | 0.28 (0.26-0.29) | 4.58 (3.38-5.79) |
| Kyrgyzstan | 925777 (898369-958423) | 21284.29 (20638.43-22021.24) | 1852307 (1763567-1944817) | 45444.08 (43259.12-47723.67) | 23789 (14896-35626) | 492.88 (312.61-726.92) | 5 (4-5) | 0.13 (0.12-0.15) | 1406988 (1365037-1455606) | 21314.42 (20658.76-22047.42) | 2875843 (2747782-3025112) | 45488.02 (43279.63-47844.87) | 35859 (22737-52946) | 510.09 (327.77-746.98) | 25 (21-30) | 0.49 (0.4-0.57) | 0.02 (-0.02-0.06) | 0.02 (-0.01-0.05) | 0.16 (0.13-0.19) | 5.92 (5.08-6.76) |
| Lao People's Democratic Republic | 1058858 (1009331-1110733) | 26852.26 (25707.47-28091.24) | 2261573 (2135064-2430459) | 58619.63 (55384.84-62493.58) | 24766 (16153-35792) | 590.35 (393.04-850.53) | 62 (44-87) | 3.28 (2.13-5.45) | 2010007 (1917942-2109391) | 28212.54 (27030.8-29428.47) | 4208037 (3978730-4499180) | 59890.53 (56566.38-63528.42) | 43086 (28455-62411) | 598.14 (400.57-862.73) | 104 (72-148) | 2.73 (1.99-3.79) | 0.17 (0.16-0.18) | 0.07 (0.07-0.08) | 0.05 (0.04-0.07) | -0.62 (-0.64--0.59) |
| Latvia | 530316 (513710-549990) | 18747.35 (18156.5-19418.62) | 1305085 (1239357-1373579) | 45100.1 (42960.99-47365.47) | 9726 (6661-13578) | 364.54 (247.96-508.79) | 35 (33-38) | 1.05 (0.97-1.12) | 443259 (429036-458862) | 19687.1 (19053.04-20400.42) | 1112402 (1051151-1173501) | 46153.25 (44049.29-48502.75) | 8186 (5922-11231) | 403.22 (283.5-558.01) | 73 (64-81) | 1.96 (1.71-2.2) | 0.21 (0.19-0.24) | 0.11 (0.09-0.13) | 0.34 (0.29-0.39) | 2.01 (1.56-2.46) |
| Lebanon | 522225 (507104-538381) | 18218.48 (17724.14-18741.71) | 1087780 (1050279-1128026) | 38813.46 (37378.25-40229.24) | 11960 (8087-16682) | 405.27 (277.81-560.63) | 39 (30-50) | 2.34 (1.78-3.1) | 1069263 (1042549-1098090) | 19190.73 (18699.81-19751.85) | 2240704 (2155381-2328578) | 39479.92 (38094.06-40863) | 22976 (15701-31823) | 420.66 (285.51-585.34) | 137 (111-169) | 2.02 (1.63-2.49) | 0.22 (0.2-0.24) | 0.09 (0.08-0.1) | 0.17 (0.15-0.18) | -0.36 (-0.44--0.27) |
| Lesotho | 341107 (324056-359479) | 23576.66 (22580.81-24708.92) | 1060932 (1013591-1112152) | 74879.9 (71602.1-78402.66) | 6536 (4316-9520) | 421.96 (281.86-614.63) | 17 (12-27) | 2.28 (1.59-3.75) | 442015 (420602-466704) | 24750.22 (23695.53-25893.72) | 1336059 (1274062-1406450) | 76885.33 (73380.35-80708.5) | 8640 (5912-12387) | 484.85 (346.32-688.48) | 38 (27-50) | 4.58 (3.24-5.9) | 0.16 (0.16-0.17) | 0.09 (0.08-0.1) | 0.54 (0.46-0.62) | 2.78 (2.18-3.37) |
| Liberia | 653064 (594915-721061) | 27346.58 (25588.15-29304.09) | 1859271 (1743455-1990749) | 80528.91 (76575.6-84933.64) | 9923 (6242-15243) | 382.12 (238.03-586.51) | 2 (2-3) | 0.09 (0.07-0.11) | 1383946 (1284626-1488178) | 26572.05 (25074.39-28096.42) | 4024678 (3795155-4277165) | 80152.39 (76135.29-84507.37) | 22413 (14290-33569) | 391.41 (246.43-592.08) | 8 (5-13) | 0.15 (0.09-0.25) | -0.06 (-0.09--0.04) | 0.01 (-0.01-0.03) | 0.12 (0.1-0.14) | 2.22 (2-2.44) |
| Libya | 749041 (726956-773667) | 19275.28 (18773.17-19781.76) | 1495073 (1444216-1549117) | 40112.17 (38725.19-41569.21) | 16920 (11200-24061) | 389.96 (258.86-551.12) | 15 (10-28) | 0.88 (0.54-1.64) | 1258460 (1221648-1298509) | 18956.26 (18447.13-19468.4) | 2579606 (2484837-2685963) | 39223.89 (37792.43-40664.27) | 27143 (18191-37909) | 411.6 (276.71-576.58) | 50 (36-65) | 1.2 (0.88-1.6) | 0.02 (-0.02-0.07) | -0.02 (-0.05-0) | 0.25 (0.2-0.29) | 1.91 (1.57-2.25) |
| Lithuania | 748149 (724586-774100) | 19669.43 (19059.02-20361.17) | 1821378 (1734649-1909597) | 46881.84 (44725.78-49133.22) | 14366 (9543-20227) | 399.04 (263.85-561.43) | 27 (23-31) | 0.64 (0.54-0.72) | 669625 (649483-691452) | 20809.73 (20199.14-21503.57) | 1685593 (1595522-1773822) | 48264.52 (46156.72-50428.71) | 11861 (8185-16560) | 431.4 (293.4-609.33) | 59 (51-68) | 1.14 (0.98-1.31) | 0.24 (0.21-0.26) | 0.12 (0.11-0.14) | 0.31 (0.28-0.35) | 2.42 (1.94-2.91) |
| Luxembourg | 110569 (107579-113876) | 27663.82 (26915.72-28497.83) | 206350 (194133-217815) | 48812.85 (46175.45-51462.61) | 2236 (1511-3142) | 602.29 (402.65-853.39) | 3 (3-3) | 0.59 (0.54-0.62) | 204715 (199890-210326) | 29395.51 (28694.95-30208.09) | 379493 (357627-400263) | 50596.02 (47978.71-53246.83) | 4038 (2772-5695) | 635.71 (425.78-896.89) | 11 (9-12) | 0.85 (0.74-0.94) | 0.2 (0.19-0.22) | 0.12 (0.11-0.13) | 0.18 (0.17-0.19) | 1.07 (0.8-1.35) |
| Madagascar | 3171237 (3013645-3350818) | 27717.57 (26511.48-28977.21) | 9018273 (8522220-9520991) | 80238.03 (76516.73-84139.12) | 68714 (47683-98419) | 547.1 (376.77-784.1) | 234 (162-402) | 3.56 (2.24-7.18) | 7627600 (7267509-8023930) | 27830.97 (26767.56-29091.01) | 21417324 (20324327-22494646) | 80405.41 (76818.45-84299.48) | 155507 (104765-225713) | 536.42 (369.73-774.57) | 364 (224-612) | 3.15 (1.92-5.48) | 0.02 (0.01-0.03) | 0.01 (0-0.01) | -0.03 (-0.05-0) | -0.26 (-0.35--0.17) |
| Malawi | 2835091 (2606866-3081855) | 29179.38 (27581.29-31008.56) | 7718348 (7265514-8271041) | 82174.44 (78114.59-86713.18) | 62580 (45696-89409) | 581.61 (412.49-824.54) | 247 (177-318) | 4.1 (3-5.86) | 5678300 (5292959-6117704) | 29205.75 (27698.42-30879.66) | 15252542 (14371764-16230276) | 82546.56 (78629.85-86925.36) | 113487 (76374-166552) | 569.45 (392.32-817.39) | 331 (224-452) | 4.21 (3.11-5.54) | 0.01 (0-0.02) | 0.01 (0-0.02) | -0.05 (-0.08--0.03) | 0.13 (-0.05-0.32) |
| Malaysia | 4764312 (4556710-4991641) | 28406.3 (27234.55-29631.63) | 9930090 (9386636-10636833) | 60225.46 (56920.02-64213.35) | 105772 (69370-152367) | 619.68 (418.55-880.95) | 325 (265-407) | 3.74 (2.97-4.83) | 9396482 (9004010-9797075) | 29756.74 (28590.47-30971.54) | 19342875 (18278453-20614661) | 61555.23 (58245.69-65502.27) | 221592 (156502-308517) | 727.08 (522.45-1003.47) | 1991 (1705-2294) | 8.43 (7.15-9.81) | 0.15 (0.14-0.15) | 0.07 (0.06-0.07) | 0.71 (0.63-0.79) | 3.87 (3.34-4.41) |
| Maldives | 56859 (54302-59579) | 27636.49 (26451.08-28844.36) | 119543 (112727-128494) | 59525.73 (56351.46-63645.97) | 1310 (858-1896) | 602.21 (411.71-855.68) | 3 (2-4) | 3.98 (2.63-5.74) | 144589 (137468-152264) | 28959.88 (27795.75-30185.42) | 305164 (286967-326898) | 60597.64 (57362.18-64550.22) | 2849 (1870-4145) | 595.34 (395.16-862.72) | 7 (5-8) | 2.35 (1.88-2.89) | 0.16 (0.15-0.16) | 0.06 (0.06-0.06) | -0.02 (-0.04-0.01) | -1.64 (-1.93--1.34) |
| Mali | 3033185 (2741054-3327951) | 34477.37 (32191.62-36840.89) | 7241220 (6746939-7837855) | 87323.71 (82526.47-92941.44) | 40294 (24780-63679) | 434.54 (263.62-687.55) | 7 (5-10) | 0.08 (0.07-0.1) | 8566711 (7755263-9419032) | 34924.91 (32595.16-37244.67) | 20305098 (19003721-21994390) | 88813.89 (84154.25-94327.42) | 119094 (73904-189675) | 452.4 (275.03-713.98) | 37 (25-54) | 0.13 (0.09-0.2) | 0 (-0.01-0.02) | 0.05 (0.04-0.07) | 0.13 (0.12-0.14) | 1.98 (1.67-2.29) |
| Malta | 99245 (96379-102184) | 26337.51 (25589.89-27126.45) | 179998 (169686-190363) | 46797.25 (44217.45-49463.24) | 2291 (1594-3149) | 622.63 (430.21-860.68) | 12 (11-13) | 3.22 (2.88-3.47) | 144118 (140376-148321) | 28184.69 (27450.02-28962.18) | 278418 (261033-297354) | 48755.95 (46182.45-51437.95) | 3316 (2457-4402) | 672.51 (469.44-928.83) | 50 (40-56) | 4.32 (3.57-4.9) | 0.22 (0.21-0.23) | 0.13 (0.13-0.14) | 0.18 (0.13-0.22) | 0.08 (-0.38-0.54) |
| Marshall Islands | 11428 (10933-11970) | 25954.79 (25058.22-26923.89) | 26498 (24741-28351) | 62226.04 (58918.29-65849.18) | 303 (198-442) | 656.81 (446.58-948.1) | 1 (0-1) | 3.14 (2.21-4.85) | 14537 (14001-15139) | 26406.32 (25492.19-27405.58) | 33999 (32054-36259) | 62672.17 (59311.54-66351.96) | 376 (252-541) | 678.19 (458.93-967.9) | 1 (1-1) | 3.43 (2.61-4.7) | 0.06 (0.05-0.06) | 0.02 (0.02-0.02) | 0.1 (0.09-0.12) | 0.28 (0.19-0.37) |
| Mauritania | 558247 (513376-611126) | 27894.06 (26180.64-29705.12) | 1572399 (1477235-1689766) | 81483.19 (77398.4-86046.75) | 8495 (5289-13094) | 392.63 (243.45-605.42) | 1 (1-2) | 0.08 (0.06-0.1) | 1159372 (1081675-1246946) | 27370.22 (25894.34-28923.05) | 3332863 (3143753-3532139) | 81353.51 (77303.95-85830.44) | 18764 (11868-28509) | 404.64 (254.06-621.76) | 5 (3-8) | 0.12 (0.08-0.2) | -0.05 (-0.06--0.04) | 0.01 (0-0.03) | 0.11 (0.11-0.12) | 1.79 (1.55-2.03) |
| Mauritius | 297643 (284453-313000) | 28001.71 (26849.56-29243.02) | 623829 (589283-668170) | 59590.56 (56333.36-63556.48) | 5962 (3793-8764) | 544.47 (346.69-801.41) | 4 (3-4) | 0.59 (0.55-0.63) | 391167 (375097-406852) | 29294.36 (28052.1-30458.32) | 820420 (774560-873389) | 60810.87 (57543.39-64713.27) | 8069 (5566-11459) | 628.69 (423.58-898.9) | 56 (51-59) | 3.32 (3.02-3.55) | 0.14 (0.14-0.15) | 0.06 (0.06-0.07) | 0.5 (0.47-0.53) | 6.83 (6.22-7.44) |
| Mexico | 17871718 (17322349-18463092) | 22384.1 (21756.97-23008.36) | 36919223 (35242904-38911081) | 48135.22 (46018.97-50525.31) | 423182 (283543-602290) | 499.02 (338.23-703.51) | 929 (899-954) | 2.47 (2.36-2.54) | 30961555 (30090341-31810404) | 24022.44 (23337.82-24679.85) | 67152113 (63932156-70811162) | 52356.69 (49869.64-55139.42) | 703990 (488022-986684) | 551.76 (380.9-772.82) | 3652 (2869-4818) | 3.07 (2.44-4.02) | 0.27 (0.24-0.3) | 0.35 (0.3-0.41) | 0.49 (0.44-0.53) | 1.34 (0.58-2.1) |
| Mongolia | 434492 (421299-450669) | 20911.92 (20257.99-21654.68) | 789045 (749738-831404) | 42455.74 (40274.18-44749.57) | 12303 (7899-18178) | 515 (337.82-750.26) | 14 (10-18) | 0.97 (0.66-1.42) | 700104 (680068-723642) | 22078.91 (21420.58-22822.24) | 1331837 (1272084-1404084) | 43780.86 (41587.38-46087.65) | 18291 (12051-26375) | 545.97 (361.43-781.61) | 37 (28-49) | 1.47 (1.12-1.91) | 0.21 (0.19-0.23) | 0.12 (0.11-0.14) | 0.22 (0.19-0.25) | 1.49 (1.17-1.81) |
| Montenegro | 127055 (123269-131385) | 20349.15 (19743.27-21051.45) | 278395 (264721-293065) | 44586.49 (42357.65-46814.3) | 2513 (1618-3622) | 407.91 (262.37-587.79) | 0 (0-0) | 0.02 (0.01-0.02) | 140434 (136145-145139) | 20903.33 (20314.86-21590.55) | 317579 (299958-335700) | 45111.45 (42921.26-47443.76) | 2499 (1614-3646) | 420.34 (271.33-610.16) | 0 (0-0) | 0.02 (0.02-0.03) | 0.14 (0.11-0.17) | 0.07 (0.05-0.08) | 0.13 (0.11-0.14) | 1.2 (0.92-1.47) |
| Morocco | 4198749 (4072246-4338896) | 17570.68 (17069.69-18070.62) | 8837946 (8524415-9155779) | 38241.85 (36768.82-39733.36) | 96355 (63621-136990) | 371.09 (245.8-524.37) | 92 (65-140) | 0.71 (0.47-1.14) | 6822308 (6632656-7020626) | 18598.73 (18077.93-19124.34) | 14227124 (13697828-14777292) | 39055.84 (37617.24-40537.4) | 145977 (98324-204446) | 399.79 (269.86-560.2) | 339 (248-449) | 1.26 (0.93-1.67) | 0.2 (0.19-0.21) | 0.08 (0.08-0.09) | 0.27 (0.26-0.28) | 2.25 (2.1-2.4) |
| Mozambique | 3646904 (3482101-3813011) | 27736.89 (26667.02-28906.95) | 10314776 (9676808-10962105) | 80584.37 (76552.47-84657.24) | 71682 (49586-105019) | 476.76 (315.77-711.9) | 120 (85-161) | 0.87 (0.63-1.26) | 8745160 (8324091-9181226) | 28623.27 (27490.79-29816.05) | 24188191 (22802207-25667196) | 82092.43 (78109.63-86410.35) | 164275 (108511-241596) | 481.17 (314.61-719.45) | 150 (93-230) | 0.77 (0.5-1.21) | 0.14 (0.12-0.15) | 0.06 (0.05-0.08) | 0.08 (0.06-0.1) | 0.04 (-0.08-0.17) |
| Myanmar | 10369544 (9860090-10888382) | 26564.53 (25395.29-27819.56) | 22102548 (20861446-23656039) | 58120.7 (54958.92-61833.2) | 234995 (156448-342295) | 579.28 (384.77-836.12) | 574 (431-797) | 2.8 (2.01-4.45) | 15781857 (15104452-16502184) | 28177.94 (27027.76-29429.6) | 33123332 (31331570-35415948) | 59705.68 (56415.38-63575.74) | 328057 (217727-475576) | 588.78 (394.55-852.58) | 912 (722-1147) | 2.33 (1.85-2.95) | 0.21 (0.2-0.23) | 0.1 (0.09-0.1) | 0.05 (0.04-0.05) | -0.81 (-0.89--0.73) |
| Namibia | 322921 (306491-340568) | 24780.57 (23782.84-25897.65) | 975702 (927971-1025272) | 76537 (73185.38-80074.24) | 6099 (3999-8941) | 437.27 (297.36-632.45) | 12 (9-18) | 2.44 (1.77-3.8) | 583520 (557389-612923) | 25508.13 (24516.8-26640.86) | 1739618 (1662429-1827272) | 77428.09 (74030.34-81027.48) | 10844 (7150-15705) | 463.07 (313.52-665.14) | 32 (24-45) | 3.07 (2.27-4.4) | 0.11 (0.1-0.11) | 0.04 (0.04-0.05) | 0.15 (0.11-0.2) | 0.4 (0.02-0.78) |
| Nepal | 3996891 (3812923-4187755) | 20992 (20169.67-21755.13) | 11181062 (10658955-11753062) | 60349.63 (57752.03-63054.18) | 92548 (59085-135257) | 437.6 (280.92-637.27) | 8 (6-10) | 0.05 (0.04-0.06) | 6663676 (6417130-6902474) | 21843.06 (21083.87-22582.06) | 18722365 (17978488-19516876) | 62417.69 (59989.73-65001.35) | 148222 (95999-213679) | 467.61 (301.4-675.86) | 39 (30-55) | 0.13 (0.1-0.18) | 0.13 (0.12-0.14) | 0.11 (0.1-0.12) | 0.24 (0.23-0.25) | 4.74 (3.54-5.95) |
| Netherlands | 4187545 (4066420-4308497) | 26823.54 (26051.99-27636.69) | 7975550 (7517332-8424616) | 48934.52 (46333-51665.32) | 90153 (62516-124866) | 605.56 (413.71-847.65) | 444 (380-481) | 2.22 (1.9-2.4) | 5567025 (5421366-5731189) | 28352.62 (27622.99-29151.46) | 10838428 (10192116-11552841) | 50431.15 (47822.42-53061.65) | 115185 (81372-158470) | 635.89 (437.59-889.68) | 995 (815-1118) | 2.46 (2.03-2.75) | 0.19 (0.18-0.21) | 0.11 (0.1-0.12) | 0.15 (0.13-0.17) | -0.13 (-0.36-0.09) |
| New Zealand | 961155 (934141-990773) | 27502.81 (26753.03-28311.93) | 2075012 (1973779-2191359) | 58555.54 (55855.9-61715.18) | 19598 (12829-27990) | 581.89 (379.72-830.64) | 33 (30-36) | 0.9 (0.82-0.97) | 1618009 (1575001-1662713) | 28584.76 (27810.22-29402.19) | 3538606 (3362027-3746236) | 59695.59 (57036.84-62862.08) | 31528 (21001-44889) | 609.72 (400.44-864.36) | 153 (130-169) | 1.64 (1.41-1.81) | 0.14 (0.14-0.15) | 0.08 (0.07-0.09) | 0.18 (0.16-0.2) | 2.41 (1.89-2.94) |
| Nicaragua | 780439 (752251-810274) | 21482.52 (20811.27-22132.5) | 1647975 (1562152-1755892) | 47700.77 (45343.3-50184.96) | 19180 (12453-27735) | 467.11 (303.14-668.42) | 16 (14-18) | 0.89 (0.74-1.12) | 1416042 (1372113-1460317) | 22064.01 (21380.56-22717.01) | 3020048 (2876285-3180529) | 48042.34 (45678.29-50429.88) | 32066 (20966-45860) | 487.2 (320.74-694.8) | 53 (45-63) | 1.2 (1-1.43) | 0.1 (0.09-0.11) | 0.03 (0.03-0.04) | 0.17 (0.16-0.18) | 1.65 (1.39-1.91) |
| Niger | 2313858 (2193716-2433874) | 30010.09 (28660.64-31520.16) | 6305215 (5872431-6749090) | 84262.2 (79993.34-89010.08) | 33745 (21186-52354) | 397.09 (244.37-619.31) | 6 (5-9) | 0.08 (0.06-0.11) | 6947450 (6627399-7299013) | 29260.67 (27923.08-30716.13) | 19450231 (18265052-20651699) | 84013.68 (80117.74-88523.09) | 108630 (67798-165507) | 403.15 (248.35-623.88) | 35 (18-59) | 0.12 (0.06-0.19) | -0.11 (-0.13--0.08) | -0.01 (-0.03-0) | 0.04 (0.03-0.06) | 1.51 (1.24-1.77) |
| Nigeria | 28695255 (26561577-31059377) | 33375.27 (31400.55-35607.75) | 78488190 (73791260-83572135) | 92043.28 (87371.86-97264.81) | 435169 (273571-669486) | 471.96 (295.38-729.52) | 63 (52-85) | 0.07 (0.06-0.1) | 75866829 (70306832-82099734) | 34231.72 (32277.9-36475.51) | 202188900 (189818680-215602203) | 93310.57 (88592.83-98364.93) | 1205104 (768007-1833053) | 502.19 (317.19-775.26) | 303 (230-414) | 0.12 (0.09-0.17) | 0.08 (0.07-0.1) | 0.05 (0.04-0.07) | 0.24 (0.22-0.26) | 2.12 (1.68-2.55) |
| Northern Mariana Islands | 11984 (11527-12473) | 28305.23 (27386.54-29282.12) | 27482 (25889-29317) | 64837 (61409.19-68673.5) | 297 (198-432) | 705.54 (483.19-1015.03) | 1 (1-1) | 4.21 (3.34-5.28) | 13772 (13313-14251) | 28221.2 (27302.75-29172.38) | 31596 (29869-33473) | 64785.1 (61266.31-68501.57) | 369 (264-518) | 769.93 (544.95-1081.81) | 3 (2-3) | 6.88 (5.77-8.08) | -0.03 (-0.05--0.02) | -0.01 (-0.02--0.01) | 0.37 (0.31-0.42) | 2.13 (1.76-2.49) |
| Norway | 1255007 (1220766-1291344) | 27380.64 (26633.91-28212.55) | 2497276 (2348453-2643818) | 51244.44 (48518.33-53973.33) | 24091 (15901-34243) | 574.96 (376.71-817.47) | 40 (35-43) | 0.52 (0.45-0.55) | 1739717 (1694125-1788507) | 28958.51 (28215.51-29797.38) | 3444527 (3244364-3638241) | 52827.56 (50053.18-55621.74) | 34113 (23366-47628) | 617.8 (412.17-868.71) | 263 (216-291) | 2.02 (1.68-2.22) | 0.21 (0.19-0.22) | 0.1 (0.1-0.11) | 0.28 (0.26-0.3) | 5.4 (4.94-5.86) |
| Oman | 331190 (321439-342008) | 18965.97 (18461.79-19511.74) | 681432 (659082-707068) | 39829.26 (38373.98-41253.14) | 7829 (5311-11031) | 419.65 (297.83-579.2) | 15 (11-22) | 2.55 (1.73-3.85) | 847214 (820855-874645) | 20251.53 (19722.21-20842.56) | 1712028 (1646139-1786481) | 40830.03 (39410.89-42321.04) | 19090 (12875-26698) | 467.31 (326.04-642.62) | 49 (39-61) | 3.61 (2.6-4.78) | 0.26 (0.24-0.29) | 0.12 (0.1-0.13) | 0.5 (0.45-0.54) | 2 (1.71-2.28) |
| Pakistan | 23479479 (22452024-24569944) | 21547.36 (20795.32-22350.26) | 65859750 (62651297-69292162) | 62014.95 (59395.94-64795.71) | 549200 (360099-783806) | 456.59 (301.04-653.69) | 614 (472-808) | 0.88 (0.63-1.24) | 51060957 (49131134-53129011) | 22513.63 (21778.36-23293.78) | 142404015 (136318141-149200961) | 64210.51 (61686.8-66989.34) | 1136351 (734306-1639314) | 467.85 (304.68-675.86) | 871 (599-1140) | 0.73 (0.49-0.97) | 0.14 (0.14-0.15) | 0.11 (0.11-0.12) | 0.08 (0.07-0.09) | -0.81 (-0.91--0.71) |
| Palestine | 325104 (314619-336088) | 17201.36 (16714.34-17733.08) | 678045 (655046-702743) | 37657.16 (36207.3-39117.65) | 7901 (5187-11279) | 370.54 (247.22-521.78) | 7 (5-10) | 0.94 (0.63-1.46) | 883091 (853231-916755) | 18209.42 (17681.12-18781.52) | 1780169 (1715043-1848268) | 38411.04 (36955.27-39809.82) | 20520 (13594-29173) | 393.78 (261.31-554.92) | 16 (14-19) | 0.97 (0.81-1.15) | 0.19 (0.19-0.2) | 0.07 (0.07-0.08) | 0.22 (0.2-0.23) | 0.18 (-0.07-0.44) |
| Panama | 511467 (494264-528417) | 22426.84 (21765.59-23093.47) | 1074167 (1022114-1132320) | 48635.98 (46367.4-51158.09) | 11902 (7766-17031) | 494.45 (325.23-705.21) | 18 (16-20) | 1.22 (1.07-1.37) | 1025021 (995424-1054096) | 23741.5 (23055.11-24409.9) | 2153838 (2047817-2262078) | 49841.39 (47436.08-52302.1) | 23427 (16186-32601) | 545.54 (376.07-760.23) | 122 (94-146) | 2.66 (2.06-3.19) | 0.19 (0.18-0.2) | 0.09 (0.08-0.09) | 0.32 (0.3-0.34) | 2.61 (2.45-2.77) |
| Papua New Guinea | 1014262 (972182-1060279) | 25667.33 (24772.26-26666.38) | 2391412 (2245394-2557978) | 61881.34 (58354.76-65666.79) | 26909 (17755-39095) | 645.81 (431.36-923.56) | 56 (31-103) | 2.81 (1.32-5.8) | 2635539 (2537065-2749374) | 26228.12 (25322.82-27193.62) | 6198950 (5852180-6623188) | 62583.15 (59181.17-66210.66) | 68731 (46002-99237) | 656.95 (447.39-943.73) | 149 (80-257) | 2.7 (1.38-5.12) | 0.06 (0.06-0.07) | 0.03 (0.03-0.04) | 0.05 (0.05-0.06) | -0.12 (-0.18--0.07) |
| Paraguay | 1035113 (995309-1079065) | 26915.29 (26000.41-27919.82) | 2403791 (2280755-2567404) | 64395.83 (61224.76-68436.8) | 24039 (15311-35402) | 572.27 (367-839.57) | 23 (17-39) | 1.01 (0.76-1.75) | 1922507 (1855370-1996924) | 27672.91 (26760.99-28679.31) | 4450836 (4234190-4736843) | 65103.37 (62018.23-69181.46) | 41840 (26900-60644) | 596.49 (384.82-865.9) | 90 (69-111) | 1.64 (1.26-2.02) | 0.09 (0.08-0.09) | 0.03 (0.03-0.04) | 0.17 (0.15-0.18) | 2.36 (2.09-2.63) |
| Peru | 6023285 (5773473-6299649) | 29302.44 (28154.46-30505.42) | 12913719 (12148588-13727100) | 64915.03 (61259.41-68996.77) | 129701 (84706-186597) | 589.46 (386.45-850.45) | 129 (108-152) | 1.05 (0.88-1.27) | 10741801 (10325875-11165307) | 30035.17 (28914.83-31195.29) | 23349831 (22075802-24764235) | 65453.82 (61825.62-69460.3) | 216963 (141468-311872) | 607.17 (396.34-873.77) | 315 (234-423) | 0.93 (0.69-1.26) | 0.09 (0.07-0.1) | 0.03 (0.02-0.04) | 0.04 (0-0.09) | -1.96 (-3.11--0.79) |
| Philippines | 16808591 (16050213-17605560) | 28090.96 (26968.37-29348.43) | 36249369 (34126215-38772729) | 62114.22 (58782.65-66172.56) | 388086 (259256-556787) | 619.18 (422.12-883.37) | 872 (747-1087) | 3.45 (2.95-4.36) | 32191691 (30890221-33669102) | 29016.1 (27886.04-30256.1) | 68843669 (64945344-73402715) | 62955.46 (59637.04-66971.39) | 708930 (476197-1014738) | 642.48 (438.94-913.41) | 2537 (2176-2882) | 3.92 (3.36-4.5) | 0.1 (0.09-0.11) | 0.04 (0.04-0.04) | 0.15 (0.13-0.17) | 0.74 (0.63-0.85) |
| Plurinational State of Bolivia | 1686782 (1614230-1764957) | 28325.68 (27227.84-29583.76) | 3704352 (3482505-3950565) | 63960.81 (60257.91-67680.59) | 36467 (23543-52410) | 559.8 (366.46-807.34) | 26 (18-32) | 0.83 (0.62-1.11) | 3331014 (3199043-3475948) | 29240.68 (28130.61-30430.51) | 7265121 (6854738-7686829) | 64683.81 (61023.74-68393.77) | 68737 (44952-98530) | 590.82 (388.02-848.63) | 84 (61-110) | 1.12 (0.83-1.48) | 0.11 (0.1-0.11) | 0.04 (0.04-0.04) | 0.18 (0.18-0.19) | 1.16 (1.09-1.23) |
| Poland | 8040897 (7797173-8291123) | 20676.33 (20065.51-21328.57) | 17541470 (16582508-18536431) | 44538.31 (42193.57-46917.52) | 164783 (108160-235964) | 439.61 (289.07-631.04) | 177 (169-183) | 0.43 (0.41-0.45) | 9447331 (9168371-9763846) | 21783.4 (21192.33-22371.22) | 21262377 (19992073-22519413) | 45541.35 (43211.35-47876.31) | 172240 (115149-245638) | 458.49 (301.94-651.17) | 481 (429-530) | 0.68 (0.61-0.75) | 0.19 (0.18-0.19) | 0.08 (0.08-0.09) | 0.16 (0.12-0.2) | 1.96 (-0.63-4.61) |
| Portugal | 2859073 (2776267-2951437) | 26692.89 (25920.56-27533.18) | 5245183 (4932328-5563406) | 47411.08 (44806.22-50038.1) | 58572 (39382-82423) | 578.45 (387.39-808.07) | 77 (71-82) | 0.65 (0.6-0.7) | 3503692 (3410428-3610284) | 28260.57 (27477.54-29086.61) | 6872317 (6442895-7353307) | 48927.01 (46413.35-51543.68) | 66247 (45039-93147) | 616.3 (411.65-867.83) | 301 (254-329) | 0.95 (0.82-1.03) | 0.17 (0.15-0.19) | 0.1 (0.08-0.11) | 0.16 (0.13-0.19) | -0.04 (-0.89-0.82) |
| Principality of Monaco | 10390 (10120-10693) | 29187.91 (28458.13-29959.75) | 20555 (19318-21914) | 51523.51 (48874.92-54125.43) | 185 (125-265) | 615.4 (409.62-877.85) | 0 (0-0) | 0.23 (0.18-0.29) | 14139 (13798-14510) | 30826.93 (30065.2-31621.9) | 27557 (25911-29408) | 53339.91 (50717.33-55991.01) | 246 (163-353) | 641.09 (425.81-905.67) | 0 (0-0) | 0.26 (0.2-0.35) | 0.19 (0.18-0.19) | 0.12 (0.12-0.12) | 0.14 (0.14-0.15) | 0.47 (0.41-0.53) |
| Puerto Rico | 958552 (927834-987754) | 26403.74 (25545.89-27197.07) | 2160908 (2054895-2290281) | 59864.56 (57009.56-63426.62) | 20311 (13402-29040) | 563.18 (373.1-803.85) | 83 (77-88) | 2.62 (2.41-2.78) | 1060250 (1028177-1096814) | 27199.89 (26347.71-28047.26) | 2493118 (2339009-2661183) | 60606.99 (57665.16-64175.02) | 22875 (16732-31578) | 615.55 (424.81-861.18) | 329 (266-388) | 3.84 (3.15-4.5) | 0.09 (0.09-0.1) | 0.04 (0.03-0.04) | 0.28 (0.2-0.37) | 1.17 (0.5-1.84) |
| Qatar | 76939 (74455-79307) | 20143.07 (19615.92-20639.52) | 156970 (150544-163827) | 41113.62 (39558.12-42592.56) | 1643 (1079-2362) | 425.11 (291.62-592.27) | 1 (1-2) | 2.67 (1.97-3.81) | 553431 (536242-570403) | 21431.5 (20869.98-21999.96) | 1109504 (1059212-1164645) | 42145.39 (40618.26-43634.6) | 11297 (7608-15816) | 476.83 (340.81-657.89) | 15 (11-20) | 4.22 (3.27-5.34) | 0.27 (0.24-0.29) | 0.12 (0.11-0.14) | 0.47 (0.38-0.55) | 2.08 (1.34-2.82) |
| Republic of Cabo Verde | 92523 (86110-99615) | 26573.76 (25065.81-28115.93) | 271059 (255336-288958) | 79766.85 (75680.03-84145.71) | 1442 (899-2219) | 383.83 (236.98-588.38) | 0 (0-0) | 0.06 (0.04-0.07) | 148639 (140051-157018) | 27317.85 (25875.2-28855.17) | 437756 (415713-461591) | 81092.04 (76931.35-85372) | 2265 (1437-3465) | 410.73 (260.43-630.51) | 2 (1-2) | 0.33 (0.23-0.46) | 0.09 (0.08-0.09) | 0.06 (0.05-0.07) | 0.24 (0.23-0.26) | 6.86 (5.82-7.92) |
| Republic of C么te d'Ivoire | 3135293 (2990694-3275112) | 27336.78 (26262.73-28507.33) | 9004282 (8450402-9588717) | 80952.44 (76922.76-85131.87) | 49128 (31084-74659) | 384.68 (239.8-594.79) | 7 (5-10) | 0.08 (0.06-0.1) | 7353190 (7036575-7695159) | 27787.23 (26722.56-28948.44) | 21122240 (19972897-22499333) | 82197.15 (77934.74-86774.4) | 117275 (74654-178479) | 404.68 (253.17-625.84) | 37 (25-53) | 0.13 (0.09-0.18) | -0.02 (-0.05-0.01) | 0.02 (0.01-0.03) | 0.14 (0.12-0.15) | 2.04 (1.83-2.24) |
| Republic of Korea | 10617126 (10301314-10955979) | 24961.62 (24290.96-25667.76) | 15933925 (15150117-16809916) | 39533.62 (37439.14-41756.94) | 253019 (164465-367277) | 582.97 (380.79-840.54) | 226 (182-271) | 1.22 (0.98-1.54) | 15097229 (14727179-15478647) | 27023.47 (26391.59-27729.55) | 25994720 (24448982-27571257) | 41708.38 (39681.37-43859.63) | 281173 (183847-403593) | 602.81 (388.15-868.9) | 725 (585-851) | 0.82 (0.66-0.95) | 0.29 (0.27-0.31) | 0.19 (0.17-0.2) | 0.15 (0.14-0.17) | -1.3 (-1.53--1.07) |
| Republic of Moldova | 829816 (803536-861101) | 18879.14 (18293.05-19570.86) | 1975885 (1885888-2077047) | 45098.03 (43048.55-47303.43) | 17246 (11469-24345) | 391.39 (259.74-552.9) | 29 (26-32) | 0.66 (0.6-0.72) | 755378 (731677-783043) | 19267.34 (18652.27-19970.84) | 1900195 (1798217-2004662) | 45404.36 (43287.19-47717.81) | 14269 (9755-19734) | 414.05 (278.17-574.15) | 58 (52-65) | 1.04 (0.92-1.18) | 0.1 (0.06-0.15) | 0.05 (0.02-0.07) | 0.23 (0.18-0.27) | 1.8 (1.37-2.23) |
| Republic of Nauru | 2660 (2556-2779) | 27434.66 (26496.73-28425.96) | 6122 (5756-6553) | 64069.86 (60711.93-67812.79) | 70 (47-101) | 710.22 (495.39-1005.64) | 0 (0-0) | 4.4 (3.48-5.45) | 2928 (2819-3043) | 27403.78 (26499.4-28347.46) | 6699 (6318-7148) | 63817.84 (60414.96-67483.22) | 78 (52-112) | 721.75 (492.88-1014.89) | 0 (0-0) | 4.54 (3.03-5.93) | 0 (-0.04-0.04) | -0.01 (-0.03-0.01) | 0.03 (0-0.05) | -0.02 (-0.11-0.07) |
| Republic of Niue | 621 (599-645) | 26672.29 (25775.25-27645.19) | 1450 (1370-1544) | 62880.86 (59517-66706.17) | 16 (11-23) | 663.76 (452.75-952.34) | 0 (0-0) | 3.08 (2.3-4.03) | 471 (455-486) | 27430.32 (26497.58-28373.58) | 1094 (1034-1155) | 63661.87 (60113.53-67475.31) | 12 (9-17) | 722 (508.28-1005.83) | 0 (0-0) | 4.11 (3.32-5.03) | 0.11 (0.1-0.11) | 0.05 (0.04-0.05) | 0.21 (0.18-0.23) | 0.81 (0.75-0.88) |
| Republic of Palau | 4033 (3880-4187) | 27093.14 (26190.09-28027.59) | 9299 (8774-9927) | 63285.89 (59882.41-67117.43) | 94 (60-140) | 607.64 (391.99-912.06) | 0 (0-0) | 0.59 (0.44-0.83) | 5046 (4882-5213) | 27643.05 (26703.11-28593.06) | 11777 (11125-12456) | 63914.37 (60377.7-67636.64) | 107 (70-160) | 621.53 (401.25-919.18) | 0 (0-0) | 0.59 (0.47-0.73) | 0.06 (0.06-0.07) | 0.03 (0.03-0.03) | 0.07 (0.07-0.07) | 0.04 (0-0.08) |
| Republic of San Marino | 7070 (6875-7275) | 27756.78 (26985.5-28564.87) | 13271 (12487-14047) | 49634.94 (47045.87-52348.17) | 141 (95-202) | 600.8 (403.41-852.5) | 0 (0-0) | 0.15 (0.12-0.18) | 11080 (10815-11412) | 28888.79 (28153.2-29695.23) | 21753 (20464-23159) | 50746.85 (48187.74-53401.48) | 202 (136-288) | 621.33 (416.96-874.2) | 0 (0-0) | 0.09 (0.06-0.12) | 0.15 (0.12-0.18) | 0.08 (0.06-0.1) | 0.12 (0.1-0.14) | -0.61 (-1--0.21) |
| Republic of the Gambia | 252624 (231510-275221) | 27108.15 (25439.36-28733.51) | 725775 (679107-777318) | 80475.74 (76439.25-85042.52) | 3951 (2474-6091) | 383.17 (238.13-589.38) | 0 (0-1) | 0.07 (0.05-0.08) | 623692 (580377-673672) | 27183.09 (25622.22-28785.11) | 1789530 (1685101-1904857) | 80999.51 (77029.04-85382.95) | 10079 (6385-15221) | 398.82 (250.36-608.47) | 3 (2-5) | 0.13 (0.09-0.2) | 0.01 (0-0.01) | 0.03 (0.02-0.04) | 0.14 (0.14-0.15) | 2.7 (2.44-2.96) |
| Romania | 4712951 (4573187-4882037) | 19506.88 (18934.75-20191.31) | 11241418 (10677246-11806894) | 46231.12 (44037.51-48498.89) | 87305 (57092-125254) | 378.56 (250-541.11) | 44 (39-50) | 0.2 (0.17-0.24) | 4545015 (4403106-4694751) | 20600.16 (20014.92-21260.72) | 11161644 (10548107-11785743) | 47631.04 (45409.92-49965.02) | 76194 (50183-109954) | 402.15 (265.03-577.95) | 166 (146-188) | 0.48 (0.42-0.54) | 0.22 (0.2-0.25) | 0.12 (0.11-0.13) | 0.26 (0.24-0.29) | 4.21 (3.42-5.01) |
| Russian Federation | 33022975 (32167966-34048719) | 21554.68 (21019.22-22207.48) | 74885201 (71325732-78614924) | 47719.2 (45578.28-49883.9) | 681892 (451758-963181) | 469.89 (309.5-668.92) | 1016 (990-1035) | 0.59 (0.58-0.61) | 34678226 (33786500-35737802) | 22177.22 (21636.6-22820.85) | 81340569 (77322159-85610030) | 48393.15 (46245.65-50538.39) | 722586 (502230-993055) | 516.03 (351.27-720.07) | 3776 (3506-4063) | 1.68 (1.57-1.81) | 0.15 (0.11-0.19) | 0.08 (0.06-0.11) | 0.3 (0.27-0.33) | 2.53 (2.07-2.99) |
| Rwanda | 2534890 (2344369-2685895) | 32845.68 (31203.55-34417.97) | 5852779 (5453478-6345908) | 82985.85 (78459.58-88113.36) | 43928 (30109-65671) | 572.54 (396.38-838.75) | 154 (119-201) | 4.32 (3.3-6.46) | 4050225 (3880003-4226237) | 30673.05 (29516.09-31963.99) | 10453639 (9795493-11225320) | 82782 (78135.61-88332.47) | 70499 (46392-104946) | 527.25 (354.88-782.37) | 199 (137-303) | 3.39 (2.43-5.06) | -0.28 (-0.31--0.25) | -0.04 (-0.06--0.02) | -0.4 (-0.45--0.34) | -1.33 (-1.55--1.1) |
| Saint Kitts and Nevis | 10730 (10341-11105) | 26018.54 (25098.88-26900.97) | 24177 (22903-25686) | 59771.86 (56716.5-63387.95) | 275 (197-376) | 668.91 (486.25-905.73) | 3 (3-3) | 8.53 (7.61-9.34) | 16113 (15609-16634) | 26997.64 (26111.06-27824.43) | 36586 (34685-38844) | 60712.4 (57808.45-64170.27) | 394 (288-532) | 701.75 (509.48-942.46) | 5 (4-5) | 9.17 (7.95-10.3) | 0.12 (0.11-0.13) | 0.05 (0.05-0.05) | 0.11 (0.05-0.17) | 0.12 (-0.1-0.35) |
| Saint Lucia | 33245 (32048-34476) | 25483.08 (24624.96-26306.55) | 74423 (70526-79030) | 59032.72 (56119.29-62550.57) | 784 (529-1108) | 588.93 (408.34-819.6) | 4 (3-4) | 5.24 (4.82-5.72) | 48824 (47215-50479) | 26247.27 (25356.68-27097.66) | 113839 (107813-120918) | 59862.98 (56858.57-63359.78) | 1217 (899-1615) | 670.64 (484.03-901.62) | 18 (15-21) | 8.16 (6.8-9.58) | 0.09 (0.09-0.09) | 0.04 (0.04-0.04) | 0.29 (0.16-0.42) | 0.58 (-0.05-1.22) |
| Saint Vincent and the Grenadines | 26371 (25378-27376) | 25034.72 (24152.91-25931.22) | 59278 (56144-63068) | 58463.47 (55565.91-62143.57) | 589 (380-849) | 527.05 (345.75-757.61) | 1 (1-1) | 1.75 (1.61-1.87) | 30749 (29789-31750) | 25964.05 (25111.79-26773.92) | 71242 (67476-75827) | 59529.64 (56590.15-63113.49) | 803 (596-1067) | 690.77 (505.18-922.44) | 11 (10-12) | 8.87 (7.79-10.12) | 0.12 (0.11-0.12) | 0.06 (0.06-0.06) | 0.73 (0.52-0.93) | 4.31 (2.87-5.78) |
| Samoa | 43392 (41545-45441) | 26176.94 (25275.2-27159.06) | 100325 (94137-107069) | 62334.32 (58852.8-66028.67) | 1132 (744-1627) | 659.85 (450.55-944.35) | 3 (2-4) | 3.05 (2.36-4.22) | 56011 (53964-58206) | 26734.46 (25837.53-27689.9) | 129587 (122312-137609) | 62877.66 (59443.89-66656.07) | 1454 (975-2092) | 681.27 (460.65-974.91) | 5 (3-6) | 3.33 (2.57-4.36) | 0.08 (0.08-0.09) | 0.04 (0.03-0.04) | 0.1 (0.09-0.12) | 0.27 (0.17-0.36) |
| Sao Tome and Principe | 32588 (29979-35737) | 27250.56 (25616.17-29095.27) | 92415 (86843-98717) | 80546.2 (76593.37-85136.45) | 510 (320-781) | 390.86 (242.51-605.45) | 0 (0-0) | 0.06 (0.05-0.09) | 56825 (53049-60678) | 27281.75 (25783.26-28872.23) | 163538 (154729-173527) | 81029.58 (76933.1-85452.03) | 901 (570-1371) | 401.08 (250.57-616.5) | 0 (0-0) | 0.05 (0.03-0.07) | 0.02 (0.01-0.03) | 0.03 (0.02-0.05) | 0.11 (0.1-0.12) | -1.09 (-1.22--0.96) |
| Saudi Arabia | 2783930 (2695088-2872625) | 19505.89 (18983.14-20034.83) | 5571409 (5375361-5772929) | 40376.17 (38879.53-41859.07) | 67820 (46815-94990) | 463.64 (331.37-629.38) | 220 (149-336) | 4.67 (3.06-7.33) | 7111418 (6894022-7336854) | 20648.14 (20092.26-21229.28) | 14110172 (13571481-14725977) | 41082.34 (39619.27-42563) | 161048 (113138-223043) | 498.7 (363.18-675.21) | 591 (469-730) | 5.27 (4.35-6.54) | 0.22 (0.21-0.23) | 0.08 (0.07-0.08) | 0.26 (0.23-0.3) | 0.37 (0.19-0.55) |
| Senegal | 2021814 (1848672-2217690) | 27420.49 (25782.74-29237.52) | 5737544 (5380449-6159391) | 80822.3 (76667.51-85408.7) | 31282 (19648-48204) | 385.93 (241.28-590.19) | 5 (4-6) | 0.08 (0.06-0.1) | 4207906 (3916234-4526552) | 27530.96 (25977.27-29245.48) | 12058647 (11390014-12784590) | 81586.94 (77455.46-86000.58) | 66927 (42703-101916) | 401.88 (254.77-615.59) | 20 (12-30) | 0.13 (0.09-0.19) | 0.02 (0.01-0.04) | 0.04 (0.03-0.06) | 0.15 (0.14-0.16) | 2.16 (1.89-2.43) |
| Serbia | 1980775 (1920937-2046184) | 20175.81 (19607.38-20852.7) | 4274427 (4046808-4511314) | 43017.97 (40816.82-45320.33) | 38542 (25005-55210) | 414.3 (269.44-594.98) | 33 (29-40) | 0.38 (0.33-0.45) | 2093881 (2028774-2164372) | 20759.81 (20162.85-21434.27) | 4713148 (4430331-5008232) | 43562.59 (41315.98-45888.52) | 37297 (24747-53200) | 430.36 (280.75-612.59) | 88 (72-109) | 0.54 (0.45-0.66) | 0.14 (0.11-0.17) | 0.07 (0.05-0.09) | 0.18 (0.15-0.2) | 2.1 (1.66-2.55) |
| Seychelles | 20431 (19538-21334) | 28585.37 (27407.89-29793.03) | 42455 (40117-45136) | 60297.12 (57099.19-64021.42) | 422 (272-622) | 571.92 (369.36-840.32) | 1 (1-1) | 1.3 (1.07-1.61) | 31529 (30265-32836) | 29526.35 (28387.99-30710.53) | 65673 (62000-69893) | 61119.48 (57864.48-64926.9) | 618 (407-898) | 599 (393.1-872.89) | 2 (2-2) | 1.75 (1.53-2) | 0.1 (0.09-0.11) | 0.04 (0.04-0.05) | 0.2 (0.18-0.21) | 1.91 (1.55-2.28) |
| Sierra Leone | 1084071 (997384-1180392) | 27198.63 (25535.2-28846.29) | 3133006 (2941144-3342528) | 80355.1 (76295.28-84924.96) | 16607 (10426-25364) | 384.42 (239.08-592.34) | 4 (3-5) | 0.08 (0.06-0.09) | 2292729 (2133059-2483803) | 27016.71 (25406.63-28730.9) | 6631351 (6246763-7073102) | 80666.83 (76492.42-85274.57) | 37080 (23604-56216) | 397.57 (249.39-608.32) | 13 (9-20) | 0.13 (0.08-0.19) | -0.01 (-0.03-0) | 0.02 (0.01-0.04) | 0.13 (0.12-0.14) | 1.94 (1.55-2.32) |
| Singapore | 728426 (708171-748937) | 25117.42 (24438.82-25777.69) | 1142972 (1086384-1207188) | 40430.73 (38340.14-42584.51) | 16469 (11037-23280) | 578.18 (387.12-815.34) | 37 (35-39) | 2.13 (1.95-2.26) | 1630744 (1591568-1669553) | 27295.62 (26636.01-28015) | 2778071 (2627992-2934593) | 42856.19 (40852.8-45062.03) | 31949 (21781-44906) | 602.84 (401.64-860.87) | 173 (147-193) | 2.12 (1.79-2.37) | 0.28 (0.27-0.3) | 0.2 (0.19-0.21) | 0.17 (0.16-0.19) | 0.7 (0.43-0.96) |
| Slovakia | 1102141 (1068473-1139461) | 20468.64 (19873.28-21142.29) | 2501534 (2376182-2628021) | 46007 (43803.65-48317.38) | 21599 (14065-31063) | 415.94 (270.95-599.34) | 18 (16-20) | 0.32 (0.28-0.36) | 1294855 (1256788-1337467) | 21406.71 (20833.31-22062.55) | 3020758 (2860327-3191214) | 46972.71 (44784.2-49320.74) | 22562 (14774-32410) | 432.86 (281.81-618.41) | 33 (28-41) | 0.38 (0.31-0.46) | 0.18 (0.16-0.19) | 0.09 (0.08-0.09) | 0.17 (0.15-0.18) | 1.63 (1.05-2.21) |
| Slovenia | 425408 (412600-439708) | 20781.2 (20204.48-21470.3) | 939504 (891153-989009) | 44739.75 (42519.93-46952.96) | 8087 (5256-11565) | 424.08 (276.91-608.66) | 10 (9-11) | 0.44 (0.4-0.47) | 523599 (506886-540515) | 21496.94 (20896.85-22169.69) | 1207710 (1135496-1276312) | 45446.86 (43233.84-47775.91) | 9082 (6051-12958) | 441.21 (288.84-628.34) | 32 (27-36) | 0.68 (0.58-0.77) | 0.13 (0.12-0.14) | 0.07 (0.06-0.07) | 0.16 (0.14-0.19) | 2.59 (1.52-3.67) |
| Socialist Republic of Viet Nam | 17562545 (16753633-18444237) | 26777.78 (25627-28016.97) | 38099249 (36001949-40784109) | 59578.5 (56310.11-63472.27) | 390569 (256821-565881) | 568.11 (379.33-816.43) | 848 (590-1375) | 2.3 (1.58-3.82) | 28247655 (26990347-29489233) | 28274.45 (27071.45-29486.74) | 61056196 (57732233-64895610) | 60908.48 (57584.55-64637.95) | 574618 (377162-839660) | 588.97 (386.43-859.42) | 1694 (1286-2142) | 2.11 (1.64-2.67) | 0.18 (0.18-0.19) | 0.08 (0.07-0.08) | 0.14 (0.13-0.15) | -0.14 (-0.19--0.09) |
| Solomon Islands | 83661 (79990-87657) | 25478.78 (24524.33-26420.81) | 196579 (184285-210557) | 61728.62 (58381.38-65665.08) | 2237 (1466-3244) | 649.2 (442.99-924.61) | 4 (2-7) | 3.08 (1.64-5.43) | 171856 (165122-179322) | 25857.86 (24978.91-26823.55) | 404414 (380761-431715) | 62109.13 (58675.03-65841.08) | 4583 (3019-6579) | 673.87 (454.07-961.86) | 12 (8-20) | 3.45 (2.13-5.53) | 0.04 (0.03-0.05) | 0.01 (0.01-0.02) | 0.12 (0.1-0.14) | 0.4 (0.29-0.51) |
| Somalia | 2227008 (2041134-2453526) | 28146.14 (26513.74-30055.37) | 6134405 (5730950-6585069) | 81080.06 (77005.91-85653.07) | 46814 (32503-67134) | 561.88 (389.34-806.49) | 154 (93-252) | 4.38 (2.4-8.52) | 5940611 (5464718-6502609) | 27684 (26096.61-29427.9) | 16619486 (15577644-17809077) | 81053.63 (77165.64-85540.72) | 119022 (80481-175917) | 534.96 (364.76-776.12) | 288 (170-513) | 3.71 (2.2-7.04) | -0.05 (-0.05--0.04) | -0.01 (-0.02-0) | -0.15 (-0.16--0.14) | -0.5 (-0.55--0.44) |
| South Africa | 8855278 (8447448-9265314) | 25472.52 (24454.58-26464.13) | 26854906 (25618778-28141339) | 78481.75 (74957.53-82081.96) | 167328 (111551-240718) | 458.85 (311.26-659.54) | 510 (411-661) | 2.75 (2.14-3.69) | 14322720 (13736599-14892123) | 26042.15 (25039.49-26999.56) | 43399992 (41514349-45384932) | 79242.98 (75784.62-82679.79) | 256633 (173300-370246) | 471.88 (321.52-678.58) | 1132 (1023-1269) | 3.12 (2.81-3.48) | 0.08 (0.07-0.09) | 0.03 (0.03-0.04) | 0.08 (0.03-0.13) | 0.2 (-0.18-0.58) |
| South Sudan | 1751108 (1618382-1897665) | 30102.33 (28540.92-31945.26) | 4707689 (4418918-4997405) | 83582.9 (79316.34-87785.09) | 36484 (25325-52294) | 587.6 (408.99-844.41) | 149 (101-228) | 4.51 (2.7-8.01) | 2791688 (2609827-3011197) | 29115.74 (27667.71-30911.24) | 7617581 (7174043-8133725) | 82697.87 (78545.49-87343.95) | 59596 (41028-85445) | 601.02 (418.9-855.58) | 239 (155-364) | 5.41 (3.52-8.58) | -0.08 (-0.1--0.06) | -0.03 (-0.04--0.01) | 0.1 (0.06-0.15) | 0.61 (0.49-0.72) |
| Spain | 10278182 (10019042-10563701) | 25008.05 (24391.12-25719.37) | 18388235 (17455213-19314973) | 42788.97 (40802.16-44838.33) | 216837 (145525-306214) | 561.71 (374.04-792.9) | 474 (423-509) | 0.92 (0.82-0.99) | 13755855 (13429760-14124509) | 26539.47 (25892.21-27260.62) | 26383485 (24977451-27862837) | 44410.46 (42447.55-46454.43) | 270295 (185933-377616) | 590.33 (395.48-831.39) | 1871 (1487-2082) | 1.36 (1.11-1.5) | 0.2 (0.19-0.22) | 0.13 (0.12-0.15) | 0.17 (0.15-0.19) | 1.52 (1.17-1.87) |
| Sri Lanka | 4899360 (4701013-5071026) | 29185.35 (28130.73-30099.73) | 9899339 (9317919-10567446) | 60332.03 (57073.65-64001.9) | 95490 (60836-140625) | 550.79 (351.62-811.51) | 59 (48-75) | 0.66 (0.55-0.85) | 6955589 (6700052-7159810) | 30459.02 (29359.21-31385.05) | 14069800 (13293252-14898190) | 61475.5 (58175.9-65157.05) | 128544 (82941-187510) | 579.43 (372.71-846) | 212 (153-276) | 0.92 (0.66-1.18) | 0.31 (0.25-0.36) | 0.4 (0.28-0.52) | 0.51 (0.39-0.63) | 2.43 (1.89-2.97) |
| Sudan | 3138186 (3048923-3246715) | 16918.07 (16469.71-17403.82) | 6680290 (6458584-6924541) | 37546.37 (36160.75-39038.73) | 75126 (50199-106195) | 362.25 (242.15-511.89) | 63 (43-99) | 0.71 (0.41-1.29) | 7386723 (7151420-7659145) | 18098.39 (17601.36-18649.78) | 14944338 (14434659-15472532) | 38484.84 (37053.12-39931.44) | 175962 (116990-248889) | 400.9 (274.67-562.26) | 213 (134-375) | 1.33 (0.81-2.4) | 0.27 (0.25-0.28) | 0.11 (0.1-0.12) | 0.38 (0.36-0.4) | 2.54 (2.35-2.73) |
| Suriname | 94513 (91105-97849) | 25687.05 (24823.27-26558.2) | 212400 (201512-224930) | 59368.43 (56520.38-62885.33) | 2429 (1724-3375) | 658.51 (476.78-897.18) | 17 (14-20) | 6.82 (5.69-8.33) | 154564 (149473-159657) | 26321.14 (25451.06-27185.8) | 353729 (335815-374933) | 60062.1 (57123.44-63538.32) | 3991 (2965-5363) | 690.97 (509.66-928.71) | 44 (33-56) | 7.62 (5.67-9.72) | 0.1 (0.09-0.11) | 0.05 (0.04-0.06) | 0.25 (0.2-0.3) | 0.86 (0.66-1.06) |
| Sweden | 2577377 (2508598-2652600) | 27466.98 (26754.13-28272.65) | 5135457 (4827017-5442084) | 50669.62 (48001.51-53378.09) | 51050 (34081-72387) | 599.47 (394.38-845.58) | 147 (132-158) | 0.87 (0.78-0.94) | 3393852 (3305957-3489305) | 28786.2 (28078.15-29609.05) | 6765474 (6356531-7175793) | 52102.2 (49358.86-54853.8) | 67106 (45827-94172) | 627.13 (417.79-882.08) | 467 (387-527) | 1.63 (1.37-1.84) | 0.17 (0.16-0.18) | 0.1 (0.1-0.11) | 0.16 (0.15-0.17) | 2.23 (1.95-2.51) |
| Switzerland | 2022022 (1971241-2080785) | 27695.43 (26964.28-28498.5) | 3894284 (3665668-4108887) | 50066.15 (47478.97-52676.46) | 40772 (27670-57248) | 606.23 (408.83-848.87) | 42 (37-46) | 0.38 (0.34-0.41) | 2918297 (2846909-3001403) | 28949.47 (28224.91-29738.92) | 5717077 (5390444-6066287) | 51403.65 (48824.75-53999.37) | 56668 (38631-79239) | 633.4 (428.7-893.09) | 183 (150-207) | 0.75 (0.62-0.85) | 0.16 (0.15-0.16) | 0.1 (0.09-0.1) | 0.15 (0.14-0.16) | 2.06 (1.6-2.53) |
| Syrian Arab Republic | 2047484 (1982302-2117145) | 17421.23 (16941.29-17936.16) | 4245194 (4100751-4399331) | 38024.84 (36616.29-39474.21) | 48057 (31217-68750) | 355.11 (233.67-509.25) | 2 (1-2) | 0.04 (0.03-0.05) | 2631696 (2543899-2729753) | 18259.38 (17746.31-18814.66) | 5330484 (5135308-5542039) | 38477.47 (37048.2-39919.25) | 55244 (36429-78005) | 380.33 (248.77-538.73) | 3 (2-4) | 0.04 (0.03-0.04) | 0.19 (0.17-0.2) | 0.06 (0.05-0.07) | 0.25 (0.23-0.26) | -0.5 (-0.73--0.27) |
| Taiwan (Province of China) | 4958074 (4811504-5104528) | 24737.09 (24060.7-25399.93) | 10434726 (9977129-10902360) | 52725.78 (50490.91-55100) | 125000 (84214-177886) | 630.63 (428.41-888.41) | 505 (476-531) | 4.2 (3.89-4.45) | 6666735 (6501004-6827035) | 26566.56 (25804.1-27269.42) | 14394781 (13823803-14984407) | 55211.6 (52981.07-57511.97) | 144086 (97814-203808) | 624.32 (410.73-897.31) | 1037 (885-1163) | 2.38 (2.04-2.66) | 0.25 (0.25-0.26) | 0.19 (0.17-0.2) | -0.07 (-0.1--0.04) | -2.52 (-2.86--2.18) |
| Tajikistan | 1095461 (1063668-1134734) | 21109.32 (20463.75-21837.65) | 1980290 (1890512-2083223) | 42597.72 (40381.21-44839.72) | 29484 (18113-44738) | 483.81 (303.02-718.58) | 1 (1-1) | 0.02 (0.01-0.03) | 2025346 (1964284-2097638) | 20991.96 (20341.64-21713.3) | 3818593 (3641179-4023757) | 42481.84 (40311.85-44727.02) | 51999 (32475-77660) | 488.25 (306.56-721.1) | 1 (1-2) | 0.02 (0.01-0.03) | 0.02 (-0.03-0.07) | 0.02 (-0.01-0.05) | 0.05 (0.03-0.08) | -0.33 (-0.47--0.19) |
| Thailand | 15259198 (14543216-16010896) | 27589.91 (26456.57-28863.68) | 32066187 (30250509-34358956) | 59203.94 (55964.41-63090.92) | 340415 (225716-487694) | 612.14 (412.76-875.32) | 1148 (846-1731) | 3.62 (2.7-5.37) | 20532472 (19710467-21406799) | 28764.85 (27605.23-29983.13) | 43853807 (41383592-46636986) | 60273.99 (56925.2-64055.66) | 459419 (329430-638330) | 658.91 (457.37-919.87) | 4945 (3817-6394) | 4.7 (3.63-6.08) | 0.13 (0.13-0.14) | 0.05 (0.05-0.06) | 0.21 (0.16-0.26) | 0.76 (0.43-1.1) |
| The former Yugoslav Republic of Macedonia | 392200 (380978-405889) | 19977.32 (19399.95-20646.56) | 862433 (818827-909620) | 44212.23 (41956.49-46583.86) | 7897 (5101-11423) | 403.6 (260.9-583.77) | 1 (1-1) | 0.07 (0.06-0.09) | 480590 (465603-497138) | 20645.08 (20044.69-21318.58) | 1095370 (1033806-1157437) | 44842.74 (42583.79-47138.52) | 8463 (5463-12272) | 416.36 (267.89-601.88) | 2 (2-3) | 0.08 (0.06-0.1) | 0.13 (0.12-0.15) | 0.06 (0.05-0.07) | 0.13 (0.12-0.14) | 1.11 (0.67-1.56) |
| Timor-Leste | 200093 (190590-210205) | 27588.71 (26384.94-28797.14) | 433088 (408220-466491) | 60518.09 (57193.16-64617.28) | 4570 (2976-6654) | 583.17 (383.77-842.87) | 7 (5-10) | 2.33 (1.43-3.92) | 390324 (372788-408322) | 28712.41 (27492.11-29912.97) | 814466 (768407-871962) | 61461.72 (58108.88-65423.87) | 8610 (5599-12606) | 603.74 (398.79-872.89) | 16 (10-24) | 2.15 (1.41-3.25) | 0.22 (0.09-0.36) | 0.16 (-0.05-0.38) | 0.25 (0.05-0.45) | -0.15 (-0.33-0.02) |
| Togo | 955176 (867898-1054290) | 27194.67 (25513.02-28894.05) | 2700484 (2528889-2904359) | 80395.42 (76228.95-84859.29) | 14944 (9394-22859) | 384.6 (240.58-592.36) | 2 (1-3) | 0.06 (0.05-0.08) | 2182635 (2027977-2356168) | 27221.22 (25592.09-28964.44) | 6284120 (5931414-6700595) | 80981.86 (77029.84-85415.23) | 34652 (21917-52404) | 398.56 (249.84-610.28) | 9 (5-14) | 0.11 (0.07-0.17) | -0.01 (-0.02-0) | 0.03 (0.02-0.04) | 0.12 (0.11-0.12) | 2.1 (1.8-2.41) |
| Tokelau | 415 (399-431) | 26091.13 (25200.85-27023.33) | 971 (916-1028) | 62242.51 (58923.24-65697.76) | 11 (7-15) | 651.33 (441.29-939.3) | 0 (0-0) | 2.92 (1.94-4.35) | 376 (363-389) | 26906.05 (26008.37-27863.64) | 875 (827-926) | 63020.34 (59608.76-66573.02) | 10 (7-14) | 707.78 (490.36-1004.23) | 0 (0-0) | 3.53 (2.59-4.79) | 0.11 (0.1-0.12) | 0.05 (0.04-0.05) | 0.17 (0.13-0.22) | 0.37 (0.25-0.5) |
| Tonga | 25368 (24311-26488) | 26118.5 (25213.71-27064.79) | 58774 (55229-62662) | 62186.14 (58790.76-65723.73) | 655 (425-960) | 646.98 (427.56-932.65) | 1 (1-2) | 2.52 (1.67-3.72) | 28154 (27138-29297) | 26781.64 (25900.23-27732.33) | 65197 (61517-69343) | 62891.47 (59461.5-66603.43) | 721 (484-1029) | 672.15 (455.04-951) | 2 (2-3) | 2.98 (2.17-4.11) | 0.07 (0.07-0.08) | 0.03 (0.03-0.03) | 0.12 (0.1-0.13) | 0.55 (0.43-0.68) |
| Trinidad and Tobago | 299810 (288991-310236) | 26063.18 (25174.45-26923.61) | 670808 (636122-713646) | 59694.25 (56736.03-63398.61) | 6901 (4593-9825) | 593.73 (406.46-834.72) | 29 (27-32) | 4.44 (4.07-4.88) | 400442 (388573-412917) | 27197.53 (26338.11-28011.89) | 915024 (867553-970910) | 60895.04 (57960.55-64349.44) | 8394 (5814-11734) | 598.24 (407.7-841.21) | 57 (45-70) | 3.18 (2.53-3.89) | 0.18 (0.17-0.2) | 0.09 (0.08-0.1) | 0.02 (0-0.03) | -1.16 (-1.32--1) |
| Tunisia | 1411893 (1370221-1456099) | 17817.77 (17355.06-18315.1) | 2932628 (2829206-3038180) | 38344.91 (36911.69-39794.74) | 31988 (21069-45785) | 374.19 (246.18-534.75) | 24 (17-34) | 0.62 (0.44-0.89) | 2274543 (2213109-2342044) | 19039.19 (18526.69-19617.22) | 4743784 (4565076-4928894) | 39329.44 (37921.39-40761.54) | 47364 (32703-65668) | 409.86 (281.61-569.91) | 144 (75-284) | 1.29 (0.69-2.51) | 0.23 (0.22-0.24) | 0.1 (0.09-0.1) | 0.34 (0.32-0.35) | 2.95 (2.73-3.17) |
| Turkey | 10588618 (10279016-10930580) | 19300.6 (18811.21-19823.86) | 23071087 (22309816-23845370) | 43349.27 (41801.13-44789.54) | 247232 (163452-351977) | 419.23 (275.76-594.14) | 129 (103-171) | 0.43 (0.34-0.59) | 17548445 (17081580-18018572) | 20710.61 (20149.93-21282.91) | 37985268 (36669194-39300292) | 44479.42 (42956.35-45920.56) | 371785 (245414-523021) | 454.37 (299.25-641.41) | 421 (340-508) | 0.52 (0.41-0.63) | 0.23 (0.22-0.24) | 0.1 (0.09-0.1) | 0.27 (0.26-0.28) | 0.92 (0.43-1.41) |
| Turkmenistan | 764044 (742218-790209) | 21569.85 (20895.86-22299.6) | 1380784 (1313052-1453888) | 43054.39 (40853.42-45360.13) | 20159 (12562-30410) | 492.84 (313-722.27) | 2 (2-2) | 0.07 (0.06-0.07) | 1119791 (1087582-1154634) | 22525.01 (21867.5-23251.87) | 2139022 (2043719-2250194) | 44223.38 (42016.72-46462.06) | 27123 (17649-39528) | 527.55 (344.41-764.51) | 28 (22-36) | 0.64 (0.51-0.81) | 0.19 (0.15-0.24) | 0.12 (0.09-0.15) | 0.27 (0.23-0.31) | 9.54 (8.65-10.43) |
| Tuvalu | 2366 (2276-2461) | 25743.25 (24823.23-26655.31) | 5642 (5333-6007) | 61876.71 (58463.42-65599) | 62 (42-90) | 657.86 (444.52-950.61) | 0 (0-0) | 3.07 (2.21-4.5) | 3244 (3130-3373) | 26421.55 (25538.38-27434.59) | 7600 (7199-8055) | 62569.46 (59246.66-66216.38) | 83 (56-121) | 676.58 (460.56-978.3) | 0 (0-0) | 3.23 (2.52-4.31) | 0.07 (0.07-0.08) | 0.03 (0.03-0.03) | 0.1 (0.1-0.1) | 0.2 (0.17-0.24) |
| Uganda | 4916980 (4543192-5352530) | 28747.77 (27225.74-30518.89) | 13500798 (12697113-14391108) | 81510.94 (77605.18-85854.2) | 102424 (70163-147333) | 547.56 (368.81-797.32) | 302 (192-543) | 3.3 (1.81-7.08) | 12753284 (11862132-13773960) | 29649.88 (28159.3-31360.17) | 34153765 (32018590-36409695) | 82991.55 (78846.54-87581.59) | 254636 (168676-377025) | 562.37 (378.65-820) | 560 (358-916) | 3.29 (2.19-5.29) | 0.1 (0.09-0.11) | 0.05 (0.04-0.06) | 0.06 (0.04-0.08) | -0.25 (-0.38--0.12) |
| Ukraine | 11062785 (10718066-11465812) | 19904.34 (19259.25-20595.16) | 26742051 (25385034-28130898) | 46772.07 (44547-49022.6) | 201838 (130577-287951) | 391.55 (255.01-559.8) | 161 (139-184) | 0.22 (0.19-0.25) | 9665562 (9360465-10026662) | 19975.03 (19397.46-20694.68) | 24229029 (22871554-25608446) | 46722.13 (44587.11-48995.69) | 175169 (119760-245044) | 420.12 (283.09-591.14) | 542 (403-699) | 0.79 (0.59-1.02) | 0.06 (0.02-0.1) | 0.03 (0-0.05) | 0.25 (0.2-0.29) | 3.68 (2.77-4.6) |
| United Arab Emirates | 331230 (321002-341788) | 20767.65 (20200.26-21306.27) | 667927 (642839-696523) | 41820.7 (40322.62-43268.79) | 7059 (4635-10077) | 421.61 (287.01-598.87) | 5 (4-7) | 1.79 (1.26-2.68) | 1817527 (1757354-1881485) | 21021.57 (20471.09-21575.03) | 3660547 (3494646-3861079) | 41650.79 (40116.16-43094.83) | 35575 (23910-50177) | 446.91 (308.6-624.65) | 27 (21-35) | 2.08 (1.62-2.6) | 0.04 (0.03-0.06) | 0 (-0.01-0.01) | 0.37 (0.3-0.45) | 3.05 (2.14-3.97) |
| United Kingdom of Great Britain and Northern Ireland | 16587665 (16130114-17077208) | 27042.16 (26317.82-27823.98) | 32600485 (30669392-34474366) | 49842.99 (47205.02-52472.14) | 336005 (226786-471441) | 593.09 (394.51-839.03) | 897 (818-939) | 0.97 (0.88-1.01) | 21551598 (21002410-22148162) | 28464.69 (27748-29259.61) | 42758274 (40219565-45249968) | 51501.07 (48801.49-54173.57) | 438454 (303788-607568) | 632.91 (426.87-890.38) | 2495 (2147-2687) | 1.65 (1.44-1.77) | 0.17 (0.16-0.18) | 0.13 (0.12-0.14) | 0.22 (0.2-0.24) | 2.12 (1.7-2.55) |
| United Republic of Tanzania | 7979701 (7669451-8391865) | 30851.69 (29777.29-31999.26) | 22123015 (20800849-23592644) | 87796.4 (83868.87-92164.77) | 184003 (126283-263104) | 654.65 (452.64-952.46) | 623 (445-955) | 4.32 (2.85-7.8) | 18146383 (17491525-18843139) | 31172.66 (30124.82-32310.87) | 48397998 (45567575-51549913) | 86264.78 (81990.93-90966.81) | 367946 (247415-536696) | 608.77 (408.88-890.02) | 1012 (666-1748) | 3.65 (2.48-6.12) | -0.02 (-0.06-0.02) | -0.1 (-0.16--0.05) | -0.24 (-0.31--0.17) | -0.61 (-0.65--0.56) |
| United States of America | 64964770 (64015597-66000833) | 24732.37 (24359.49-25165.03) | 98997043 (95744121-102390164) | 36571.54 (35430.05-37825.77) | 1614091 (1102415-2243045) | 636.05 (431.22-890.91) | 2949 (2637-3120) | 0.89 (0.8-0.94) | 94589888 (93568200-95632652) | 25620.6 (25338.78-25921.25) | 144553118 (140794732-148048606) | 36869.46 (35992.27-37765.06) | 2181781 (1515228-2966464) | 638.7 (436.92-885.1) | 6874 (5890-7806) | 1.16 (1-1.31) | 0.12 (0.11-0.13) | 0.1 (0.07-0.14) | 0.07 (0.05-0.09) | 1.55 (1.18-1.92) |
| United States Virgin Islands | 27353 (26452-28250) | 26634.27 (25814.99-27487.66) | 61049 (57956-64743) | 60242.37 (57318.26-63740.69) | 604 (408-859) | 586.32 (401.41-829.45) | 2 (2-3) | 3.34 (2.73-4.12) | 28058 (27217-29033) | 27667.07 (26791.81-28501.13) | 65305 (61272-69628) | 61407.92 (58460.77-64966.97) | 565 (396-794) | 613.46 (417.81-864.2) | 5 (4-7) | 3.48 (2.68-4.56) | 0.12 (0.1-0.15) | 0.06 (0.05-0.08) | 0.21 (0.15-0.27) | 0.48 (-0.02-0.98) |
| Uruguay | 780832 (761464-802390) | 24012.74 (23392.77-24706.04) | 1481126 (1407728-1559454) | 44588.26 (42636.83-46781.58) | 17868 (12016-25052) | 562.81 (375.51-793.18) | 82 (74-91) | 2.16 (1.94-2.4) | 947631 (926020-971906) | 25507.39 (24890.2-26185.05) | 1838125 (1743944-1935854) | 46299.5 (44276.65-48499.83) | 22536 (16262-30176) | 624.92 (433.01-855.86) | 277 (246-301) | 4.26 (3.83-4.59) | 0.2 (0.19-0.21) | 0.13 (0.12-0.13) | 0.35 (0.31-0.39) | 2.22 (1.7-2.74) |
| Uzbekistan | 4273950 (4149174-4430601) | 21074.03 (20408.13-21809.28) | 7832590 (7449332-8234763) | 42500.9 (40299.87-44737.51) | 112889 (69590-171105) | 484.18 (301.84-715.31) | 6 (4-7) | 0.04 (0.03-0.06) | 7149855 (6931347-7392841) | 21841.94 (21176.12-22582.65) | 13861320 (13236197-14635313) | 43388.57 (41207.29-45577.51) | 170548 (108614-250276) | 502.52 (321.14-733.54) | 36 (29-43) | 0.13 (0.11-0.15) | 0.14 (0.12-0.17) | 0.09 (0.07-0.11) | 0.14 (0.13-0.16) | 4.06 (3.17-4.96) |
| Vanuatu | 36511 (35090-38107) | 25140.42 (24314.57-26023.44) | 84788 (79883-90358) | 59915.33 (56697.44-63329.15) | 963 (631-1390) | 636.91 (427.5-899.29) | 2 (1-4) | 3.33 (1.84-6.05) | 77389 (74396-80496) | 25454.57 (24602.11-26352.84) | 178922 (168578-190449) | 60063.96 (56815.79-63597.56) | 2050 (1354-2952) | 664.37 (453.71-933.83) | 6 (4-11) | 3.85 (2.43-6.3) | 0.05 (0.05-0.05) | 0.02 (0.01-0.02) | 0.15 (0.14-0.16) | 0.52 (0.45-0.59) |
| Yemen | 2091802 (2028407-2159580) | 17001.94 (16543.19-17490.73) | 4418112 (4268635-4585325) | 37646.55 (36207.78-39146.94) | 50949 (33820-73350) | 357.04 (238.94-507.65) | 31 (20-51) | 0.66 (0.36-1.44) | 5547047 (5370827-5744469) | 17589.33 (17101.14-18141.52) | 11332219 (10937400-11762334) | 37962.26 (36545.07-39396.04) | 131824 (87645-187411) | 380.56 (255.83-537.16) | 88 (58-136) | 0.8 (0.5-1.3) | 0.16 (0.14-0.19) | 0.06 (0.04-0.08) | 0.24 (0.22-0.26) | 0.68 (0.57-0.79) |
| Zambia | 2395999 (2198752-2617985) | 30069.06 (28339.75-32015.66) | 6325778 (5933467-6783584) | 83155.93 (79179.96-87798.71) | 52080 (37521-75190) | 608.4 (431.38-857.15) | 207 (166-250) | 4.83 (3.93-5.96) | 5822758 (5396324-6252010) | 30345.62 (28744.84-32086.33) | 15488539 (14575684-16469417) | 84154.69 (80083.21-88692.05) | 116103 (77575-168296) | 590.77 (409.51-841.57) | 347 (251-464) | 4.36 (3.23-5.79) | 0.05 (0.04-0.06) | 0.04 (0.03-0.05) | -0.11 (-0.12--0.09) | -0.44 (-0.56--0.32) |
| Zimbabwe | 2314240 (2196282-2442152) | 24185.65 (23224.63-25331.36) | 7041477 (6712864-7425032) | 75728.99 (72322.35-79356.61) | 53357 (37371-73135) | 577.58 (425.5-785.47) | 326 (250-464) | 9.82 (7.44-14.83) | 3532072 (3351692-3727938) | 24258.78 (23234.33-25386.2) | 10769159 (10247691-11321286) | 76085.55 (72669.59-79539.7) | 85175 (62080-115232) | 637.47 (480.98-836.3) | 637 (476-817) | 11.69 (8.64-15.59) | -0.01 (-0.02-0) | 0.01 (0-0.01) | 0.44 (0.3-0.58) | 1.03 (0.68-1.38) |

No., number; SSDs: skin and subcutaneous diseases; DALYs, disability-adjusted-life-years; ASIR, age-standardized incidence rate; ASPR, age-standardized prevalence rate; ASDR, age-standardized DALY rate; ASMR, age-standardized mortality rate; UI: uncertainty intervals; CI: confidence interval; SDI: socio-demographic index; GBD: global burden of diseases; EAPC: estimated annual percentage change.
